# Relationship between Nonhepatic Serum Ammonia Levels and Sepsis-Associated Encephalopathy: A Retrospective Cohort Study

**DOI:** 10.1155/2023/6676033

**Published:** 2023-10-12

**Authors:** Pei Wang, Jia Yan, Qiqing Shi, Fei Yang, Xuguang Li, Yuehao Shen, Haiying Liu, Keliang Xie, Lina Zhao

**Affiliations:** ^1^Department of Critical Care Medicine, Tianjin Medical University General Hospital, Tianjin 300052, China; ^2^Department of Anesthesiology, Minhang Hospital, Fudan University, Shanghai 201199, China; ^3^Department of Critical Care Medicine, Chifeng Municipal Hospital, Chifeng Clinical Medical College of Inner Mongolia Medical University, Chifeng 024000, China; ^4^Department of Anesthesiology, Tianjin Institute of Anesthesiology, Tianjin Medical University General Hospital, Tianjin 300052, China

## Abstract

**Objectives:**

Nonhepatic hyperammonemia often occurs in patients with sepsis. Ammonia plays an essential role in the occurrence of hepatic encephalopathy. However, the relationship between nonhepatic serum ammonia levels and sepsis-associated encephalopathy (SAE) remains unclear. Thus, we aimed to evaluate the association between serum ammonia levels and patients with SAE.

**Methods:**

Data of critically ill adults with sepsis who were admitted to the intensive care unit were retrieved from the Medical Information Mart for Intensive Care IV (MIMIC IV) between 2008 and 2019 and retrospectively analyzed. Data of patients with sepsis patients and serum ammonia not related to acute or chronic liver disease were not included.

**Results:**

Data from 720 patients with sepsis were included. SAE was found to have a high incidence (64.6%). After adjusting for other risk factors, a serum ammonia level of ≥45 *μ*mol/L (odds ratio (OR): 3.508, 95% confidence interval (CI): 2.336–5.269, *p* < 0.001) was found to be an independent risk factor for patients with SAE; moreover, as the serum ammonia level increased, the hospital mortality of SAE gradually increased in a certain range (serum ammonia <150 *μ*mol/L). Serum ammonia levels of ≥45 *μ*mol/L were associated with higher Simplified Acute Physiology Score II and Sequential Organ Failure Assessment (SOFA) scores in patients with SAE. Besides, our study found that patients with SAE used opioid analgesics (OR:3.433, 95% CI: 1.360–8.669, *p* = 0.009) and the SOFA scores of patients with SAE (OR: 1.126, 95% CI: 1.062–1.194, *p* < 0.001) were significantly higher than those without SAE.

**Conclusions:**

Nonhepatic serum ammonia levels of ≥45 *μ*mol/L evidently increased the incidence of SAE. Serum ammonia levels should be closely monitored in patients with sepsis.

## 1. Introduction

Sepsis, a life-threatening organ dysfunction caused by a dysregulated host response to infection [1], can cause multiple organ dysfunction syndromes (MODS). In sepsis, brain dysfunctions occur easily and early on [2], and brain dysfunction in sepsis without direct infection of the nerve is called sepsis-associated encephalopathy (SAE) [3]. The latest research shows that SAE has a high incidence of 49.6% [4] and a mortality of 50.3% [5]. Moreover, 45% of sepsis survivors have long-term cognitive dysfunction [6]. Since there is no effective treatment for patients with SAE patients, it is essential to control the modifiable factors to prevent SAE.

High serum ammonia levels play an essential role in the pathogenesis of hepatic encephalopathy [7]. Ammonia crosses the blood-brain barrier where it is further metabolized to glutamine; consequently, high glutamine levels in the brain induce brain edema and increase cerebral volume, and ammonia decreases excitatory neurotransmission. Patients often show consciousness changes and delirium and even go into coma [8, 9]. In recent years, nonhepatic hyperammonemia has gradually gained attention, especially in patients with sepsis. In our previous study, we found a 40.3% incidence of nonhepatic hyperammonemia in sepsis. In addition, we found that the incidence of encephalopathy was significantly higher in the nonhepatic hyperammonemia group (37.3%) than in the nonhepatic nonhyperammonemia group (19.6%), and the nonhepatic hyperammonemia group had higher short-term (hospital mortality: 59.8%; 30-day mortality: 47.7%) and long-term mortality (90-day mortality: 61.7%; 1-year mortality: 67.7%) than the nonhepatic nonhyperammonemia group [10]. Yazan et al. found that elevated ammonia levels can be a novel biomarker for sepsis [11].

Serum ammonia is an important neurotoxic molecule, and elevated nonhepatic serum ammonia levels are commonly seen in patients with sepsis. Our previous research found that the serum ammonia level was associated with higher SOFA scores and lactate levels in patients with SAE [12]. However, the relationship between nonhepatic serum ammonia levels and incidence of SAE remains unclear. Therefore, we aimed to evaluate the association of nonhepatic serum ammonia levels with SAE.

## 2. Materials and Methods

### 2.1. Setting

This study was a retrospective cohort study based on data from the Medical Information Mart for Intensive Care IV (MIMIC IV 0.4) from 2008 to 2019 [13]. MIMIC IV is a publicly available database of patients admitted to the Beth Israel Deaconess Medical Center in Boston, MA, USA. The use of data from the MIMIC IV database was approved by the Institutional Review Boards of the Massachusetts Institute of Technology and Beth Israel Deaconess Medical Center [13]. The requirement for individual patient consent was waived because the study did not impact clinical care, and all protected health information was deidentified. We included data from 69,619 ICU patients, which included 19,658 patients with sepsis, among whom 1,377 patients with sepsis had their serum ammonia levels evaluated.

### 2.2. Patients

First, the database was searched for patients with sepsis. Sepsis was defined as a suspected infection combined with an acute increase in the Sequential Organ Failure Assessment (SOFA) score of ≥2 [1]. Patients with an infection site or prescriptions for antibiotics and those who underwent sampling of bodily fluids for a microbiological culture were considered to have a suspected infection. When the antibiotic was administered before sampling, we assumed the microbiological sample must have been collected within 24 h; when microbiological sampling was conducted before administering antibiotics, we assumed the antibiotic must have been administered within 72 h [14]. Second, we searched for patients with sepsis who underwent serum ammonia level testing. We excluded patients diagnosed with acute and chronic liver disease and diseases that affect consciousness. We divided the patients into an SAE group and a non-SAE group. SAE in the study was defined as a Glasgow Coma Scale (GCS) score of <15, diagnosed delirium, or usage of haloperidol in patients with sepsis [4, 5, 15].

The inclusion criteria were as follows: (1) patients who fulfilled the definition of sepsis 3.0, (2) patients >18 years, and (3) an ICU stay of >24 h.

The exclusion criteria included the following [4, 5]: (1) patients with brain injuries, such as traumatic brain injury, meningitis, encephalitis intracerebral hemorrhage, cerebral embolism, ischemic stroke, epilepsy, or intracranial infection and other cerebrovascular diseases (Supplementary materials 1–Supplementary materials 5); (2) mental disorders and neurological diseases (Supplementary materials 6); (3) chronic alcohol or drug abuse (Supplementary materials 7); (4) metabolic encephalopathy, hepatic encephalopathy, hypertensive encephalopathy, hypoglycemic coma, and other liver or kidney diseases affecting consciousness (Supplementary materials 8); (5) severe electrolyte imbalances or glycemic disturbances, including hyponatremia (<120 mmol/l), hyperglycemia (>180 mg/dl), or hypoglycemia (<54 mg/dl); (6) a partial pressure of carbon dioxide (PaCO_2_) ≥80 mmHg; (7) patients with acute and chronic liver diseases [16, 17], including hepatitis, hepatic cirrhosis, hepatic encephalopathy, hepatorenal syndrome, hepatic injury, or other chronic liver diseases (Supplementary materials 9); and (8) missing serum ammonia values.

### 2.3. Data Collection

Patients' baseline variables, including age, sex, type of admission, ethnicity, coexisting illness (Supplementary materials 10–14), site of infection, microbiology type, length of hospital stays, and hospital mortality were extracted from the database. The patient's vital signs, including heart rate, systolic blood pressure, diastolic blood pressure, mean arterial pressure, respiratory rate, temperature, and arterial oxygen saturation (SpO_2_), were extracted. The patients' laboratory parameters, including alanine aminotransferase, aspartate aminotransferase, albumin, ammonia, lactate, bilirubin, creatinine, blood urea nitrogen, glucose, and hemoglobin levels along with platelet count, partial thrombin time, international normalized ratio, prothrombin time, white blood cell count, neutrophil count, lymphocyte count, partial pressure of carbon dioxide (PaCO_2_), and partial pressure of oxygen (PaO_2_), were extracted. The patient's disease severity score, including Sequential Organ Failure Assessment (SOFA) score, Simplified Acute Physiology Score II (SAPS), and Glasgow Coma Scale (GCS) score in the first 24 h of the patient's admission to the ICU, was extracted. The analgesic and sedative drugs used, including midazolam, propofol, and/or opioids, were extracted. The use of vasopressors, mechanical ventilation, and renal replacement therapy was extracted. The worst laboratory parameters and the mean value of vital signs during the first 24 h of ICU admission were used in the analysis in this study. R statistical software (R Foundation for Statistical Computing, Vienna, Austria) was used to extract the patient's indexes.

### 2.4. Statistical Analysis

All continuous and nonnormally distributed variables were assessed using the Shapiro–Wilk test. Age, Charlson's comorbidity index, vital signs, laboratory parameters, SAPS II score, SOFA score, GCS score, and length of hospital stays were expressed as the medians (P25, P75) (interquartile range, IQR). Categorical variables, including sex, type of admission, ethnicity, coexisting illness, site of infection, microbiology type, length of hospital stays, hospital mortality, use of analgesic and sedative drugs, vasopressors, and mechanical ventilation, were expressed as numbers and proportions. Continuous variables were examined using the Mann–Whitney *U* test in the SAE group and the non-SAE group; categorical variables were compared using Pearson's exact test.

Serum ammonia in the SAE group was significantly higher than that in the non-SAE group as shown in Table 1. We applied propensity score matching to match the covariates with in-between differences as shown in Supplementary Material 15. We considered that it may be related to the occurrence of SAE. Our data were all nonnormally distributed. We used generalized additive models [18] to further determine the relationship between serum ammonia levels and SAE. Our cohort study found that serum ammonia levels of ≥45 *μ*mol/L can increase the incidence of SAE. For further verification, we used the multivariate logistic regression analysis. After adjusting for other risk factors, serum ammonia levels of ≥45 mmol/l were identified as an independent risk factor for SAE. We used the survive package to observe the trend of changes between serum ammonia levels and the hospital mortality of SAE. Data were analyzed using R software.

## 3. Results

### 3.1. Baseline Characteristics

**Figure 1:**
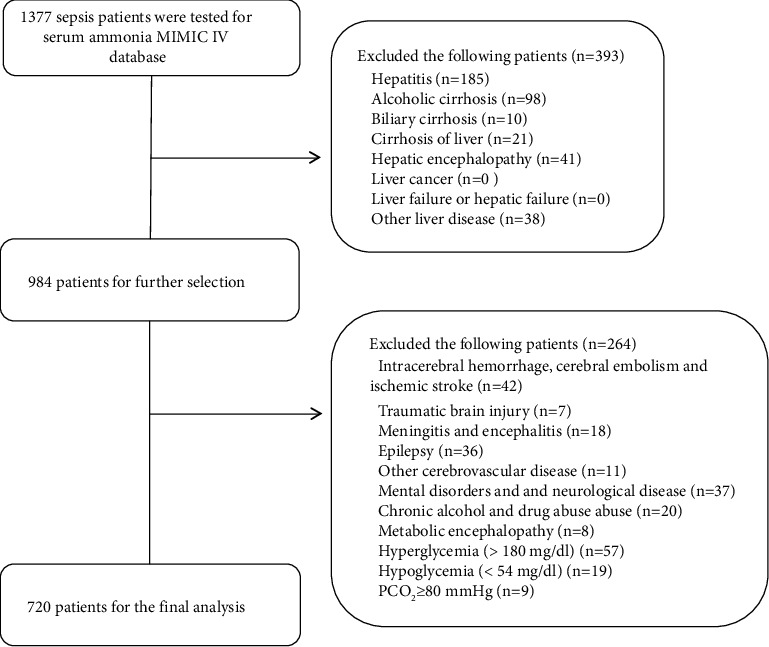
Flowchart of the study's selection process.

As shown in Figure 1, 1,377 patients with sepsis had data on their serum ammonia level in the MIMIC IV database. In total, 393 patients were diagnosed with acute and chronic liver diseases, 264 patients were diagnosed with traumatic brain injury, and other diseases were excluded. Finally, 720 patients were included in the final cohort.

Table 1 describes the characteristics and outcomes of patients with SAE in the original cohort. Compared with patients in the non-SAE group, patients in the SAE group were younger (64 (55–72) vs. 68 (58–75), *p*=0.002) and had lower platelet levels (140 (82–206) vs. 173 (98–242), *p*=0.001), higher serum ammonia levels (41 (31–63) vs. 21 (16–31), *p* < 0.001), and higher SOFA scores (6 (4–10) vs. 5 (3–8), *p* < 0.001). In addition, compared to the non-SAE group, more patients in the SAE group used opioids (7.5% vs. 3.1%, *p*=0.017), underwent mechanical ventilation (53.8% vs. 41.2%, *p*=0.017), had a longer hospital stay (4.6 (1.9–11.7) vs. 2.6 (1.3–6.2), *p* < 0.001), and had higher hospital mortality (20.6% vs. 1.2%, *p* < 0.001). To verify the accuracy of our results, propensity score matching analysis was performed on the original cohort; patients were matched for age, urinary infection, lung infection, catheter infection, abdominal cavity infection, *Klebsiella* infection, *Escherichia coli* infection, *Pseudomonas aeruginosa* infection, *Staphylococcus aureus* infection, platelet counts, and use of propofol and morphine drugs in the original cohort in a 1 : 1 match. The standardized mean differences (SMD) of the original cohort when compared with those of the IPW cohorts showed that almost all variables had SMD values of <10%, thereby indicating a good matching effect (Supplementary material 15). After propensity score matching, our cohort still showed that serum ammonia levels were significantly higher in the SAE group than in the non-SAE group (*p* < 0.001).

### 3.2. Relationship between Serum Ammonia Levels and SAE Incidence

**Figure 2:**
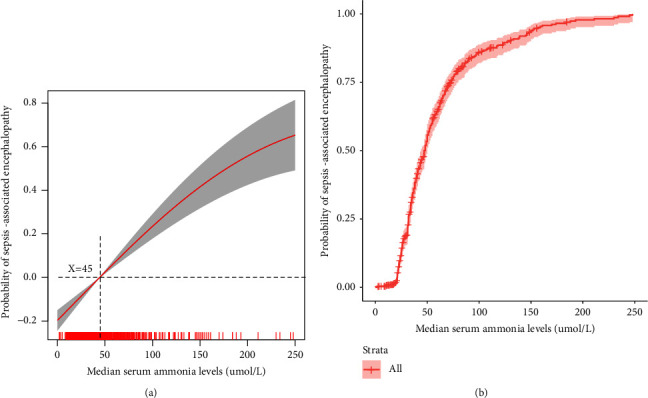
Association between serum ammonia levels and SAE incidence.

A generalized linear model was used to explore the relationship between serum ammonia levels and SAE incidence (Figure 2(a)). Serum ammonia ≥45 *μ*mol/L can increase the incidence of SAE. Furthermore, Figure 2(b) shows that as serum ammonia levels increase, the incidence of SAE increases.

### 3.3. Multivariate Logistic Analysis of Risk Factors for SAE

After adjusting for other risk factors, serum ammonia levels of ≥45 *μ*mol/L (odds ratio (OR): 3.501, 95% confidence interval (CI): 2.326–5.269, *p* < 0.001), use of opioids (OR: 3.433, 95% CI: 1.360–8.669, *p*=0.009), and SOFA scores (OR: 1.126, 95% CI: 1.062–1.194, *p* < 0.001) were risk factors for the occurrence of SAE (Table 2).

### 3.4. Relationship between Serum Ammonia Levels and SAE Hospital Mortality

**Figure 3:**
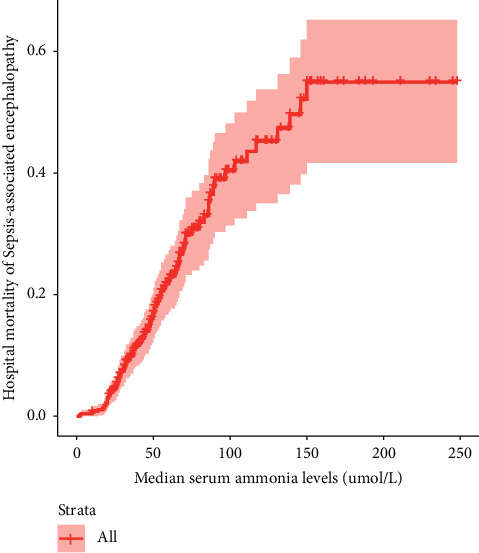
Relationship between serum ammonia levels and hospital mortality of patients with SAE.

The survival package of R Studio was used to observe the relationship between serum ammonia levels and the hospital mortality of patients with SAE.  Figure 3 demonstrates that, when serum ammonia levels were <150 *μ*mol/L, an increase in the serum ammonia level was associated with a gradual increase in the hospital mortality of patients with SAE.

### 3.5. The Effect of Serum Ammonia Level ≥45 μmol/L on Prognosis of SAE Patients

Patients with SAE who have serum ammonia levels of ≥45 *μ*mol/L had higher SOFA scores (7 (5–10) vs. 4 (6–9), *p*=0.022) than those with serum ammonia levels of <45 *μ*mol/L; however, there was no difference in length of hospital stays and hospital mortality between the two groups (Table 3).

## 4. Discussion

In the present cohort study, we demonstrated an association between serum ammonia levels and the incidence of SAE. The incidence of SAE in this cohort study was 64.4%. Patients with SAE have a worse prognosis in terms of longer hospital stays and higher hospital mortality than those without SAE. Furthermore, among patients with sepsis, those with a serum ammonia level ≥45 *μ*mol/L were 3.508 times more likely to develop SAE than those with serum ammonia levels of <45 *μ*mol/L. Patients with SAE with serum ammonia levels of ≥45 *μ*mol/l had higher SAPS II and SOFA scores than those with serum ammonia levels of <45 *μ*mol/L. Besides, we found that the use of opioids and high SOFA scores were independent risk factors for SAE.

SAE has a high incidence and poor outcome. Sonneville et al. conducted a multicenter study and demonstrated that the incidence of SAE was 53% [5]. Tomonori Yamamoto et al.'s multicenter, randomized, controlled trial found that patients with SAE had a longer ICU stay than those without SAE [19]. Feng et al. found that patients with SAE presented with high APACHE II and SOFA scores and high short-term and long-term mortality [20]. The incidence of SAE in this study was 64%. Moreover, we found that patients with SAE had significantly longer hospital stays and higher hospital mortality (20.6%) than those without SAE (1.2%). Our study results are consistent with those of previous studies. Their study results further support our outcome. SAE still has a high incidence and poor clinical outcomes. Its pathogenesis remains unclear, and effective interventions are lacking. Therefore, finding potentially modifiable factors is important.

Our study found that serum ammonia levels, use of opioids, and high SOFA scores were potentially modifiable factors for the incidence of SAE.

Ammonia is a potential neurotoxin, and elevated serum ammonia levels can cause neurological symptoms and changes in the patient's consciousness. Elevated serum ammonia levels can be due to hepatic hyperammonemia [21, 22] and nonhepatic hyperammonemia [16, 23, 24]. Elevated nonhepatic serum ammonia levels need attention. Nonhepatic hyperammonemia had a high incidence and was associated with a poor prognosis in critically ill patients. Yao et al. found that the incidence of nonhepatic hyperammonemia was as high as 75.98%. Patients with severe nonhepatic hyperammonemia had longer ICU stays and higher acute physiology assessment, chronic health assessment, and SOFA scores [23]. Stergachis et al. [24] reported that 57% of patients with severe nonhepatic hyperammonemia survived after having reversible neurological changes and 4% survived with irreversible neurological changes. Our previous study demonstrated that the incidence of nonhepatic hyperammonemia was 42.2% in critical care patients. Patients with sepsis accounted for 26.2% of the patients with nonhepatic hyperammonemia [25]. Among patients with sepsis, those with nonhepatic hyperammonemia had a significantly higher incidence of encephalopathy (37.4%) and delirium (15.9%) than those with normal serum ammonia levels [10]. Sakusic et al. [16] reported that 71% of patients with nonhepatic hyperammonemia presented with acute encephalopathy. Our cohort study found that the incidence of SAE was 64.4% and that nonhepatic serum ammonia levels in the SAE group were significantly higher than those in the non-SAE group (Table 1); moreover, patients with serum ammonia levels of ≥45 *μ*mol/L had 3.501 times higher incidence of SAE than those with serum ammonia levels of <45 *μ*mol/L (Figure 2, Table 2). Our study results further confirm those of previous research. The neurotoxicity caused by nonhepatic blood ammonia may be attributed to serum ammonia-induced TNF-*α* in glial cells; TNFR1 activated by released TNF-*α*, TNFR1 leads to nuclear translocation of NF-*κ*B and an increase of TNF-*α*. [26]. In addition, the logistic analysis showed that *Escherichia coli* infection was more frequent in patients with SAE patients than in those without SAE, thereby indicating that the occurrence of nonhepatic hyperammonemia may be related to *Escherichia coli* infection and increased ammonia production [27]. Serum ammonia is a fat-soluble substance, and increased serum ammonia can enter the brain through the blood-brain barrier and affect the activities of glutaminase, glutamine synthetase, and glutamate dehydrogenase, which are important enzymes for nervous system function [28, 29]. Nonhepatic serum ammonia is a risk factor for SAE, especially serum ammonia levels of ≥45 *μ*mol/L, although these levels do not affect the mortality in patients with SAE (Table 3); as serum ammonia levels increase, the mortality rate (serum ammonia <150 *μ*mol/L) and incidence of SAE increase (Figure 3); patients with serum ammonia levels of ≥45 *μ*mol/L had higher SOFA and SAPS II scores than those with serum ammonia levels of <45 *μ*mol/L (Table 3). We recommend that the serum ammonia levels should be closely monitored in patients with sepsis, and in patients with serum ammonia levels of ≥45 *μ*mol/L, timely and effective interventions should be taken to reduce the incidence and improve the prognosis of SAE. The mechanism of nonhepatic blood ammonia levels leading to SAE needs to be studied in the future.

Sonneville et al. found that the rate of use of opioids in patients with and without SAE was 43.7% and 25.9%, respectively [5]. Our results show that the incidence of SAE in patients with sepsis who used opioids was 3.433 times that of those who did not use opioids. The use of opioids is an independent risk factor for SAE. Our study results are consistent with those of Sonneville et al. Opioid-induced delirium has been confirmed in other patients, except for those with SAE. Pavone et al. found that among critically ill patients, those exposed to opioids were 2.5 times more likely to develop delirium than those not exposed to it [30]. The odds of postoperative day 2 delirium increased significantly with the increase in intraoperative opioid use in patients with chronic kidney disease (OR = 1.6) [31]. Future research should focus on the optimal dose and timing of administration of the opioid and pain management approaches to prevent delirium.

Leite et al. found that the SOFA score was a risk factor for the incidence of delirium in patients weaned from mechanical ventilation [32]. A previous study found that patients with SAE patients had higher SOFA scores [20], and a kidney SOFA score of >2 was associated with a 1.41 times higher incidence of SAE [5]. Our study found that the SOFA score was an independent risk factor for SAE (OR: 1.126). Our study results support those of previous studies. Thus, the more severe the organ dysfunction in patients with sepsis, the more likely SAE is to occur.

### 4.1. Limitations

This study had some limitations. First, our definition of SAE and nonhepatic serum ammonia were different from those used in previous studies. According to the reference literature retrieved from retrospective studies, the definition of SAE is based on the GCS score, the diagnosis of delirium, or the use of haloperidol drugs, and the exclusion of cerebral hemorrhage, brain trauma, and epilepsy, severe liver and kidney disease caused by consciousness disorder, or mental disorders. The definition of nonhepatic serum ammonia is based on the diagnostic code to exclude acute and chronic liver disease. They all lack imaging data, such as computed tomography, magnetic resonance imaging, electroencephalogram, and ultrasound data. Hence, there may be information bias in the SAE cohort. Second, the results of this study demonstrated that patients with sepsis who had serum ammonia levels of ≥45 *μ*mol/L had a 3.508 times higher chance of developing SAE than those with serum ammonia levels of <45 *μ*mol/L; however, a causal association with serum ammonia level ≥45 *μ*mol/L and the incidence of SAE could not be proved due to the retrospective nature of our study. Its causality should be assessed in future studies using a prospective study design. The consciousness of patients with SAE could not be observed through our intervention of serum ammonia levels. Finally, critically ill patients often have various other comorbidities. Due to the interaction between the diseases, some confounding factors still cannot be ruled out and can cover up or exaggerate the relationship between the risk factors and SAE. The small sample size of this study may lead to bias in the study results and require larger sample sizes to validate in the future.

## 5. Conclusions

Serum ammonia levels of ≥45 *μ*mol/L are associated with the incidence of SAE. These levels can provide a reference for clinicians to intervene in patients with SAE. Serum ammonia levels of ≥45 *μ*mol/L provide a reference target for future prospective research.

## Figures and Tables

**Table 1:** Baseline characteristics and outcome.

Baseline variables	Original cohort			Propensity score matching		
	SAE patients *n* = 465	Non-SAE patients *n* = 255	*p*	SAE patients *n* = 232	Non-SAE patients *n* = 232	*p*
Age	64 (55–72)	68 (58–75)	0.002	66 (60–74)	68 (58–75)	0.843
Sex (female) (*n* (%))	193 (41.5)	97 (38)	0.364	127 (54.7)	146 (62.9)	0.09
Coexisting illness (*n* (%))						
Charlson's comorbidity index	5 (3–8)	6 (4–8)	0.113	6 (4–8)	6 (4–8)	0.747
Hypertension	57 (12.3)	70 (27.5)	<0.001	41 (17.7)	53 (22.8)	0.204
Diabetes	134 (28.8)	73 (28.6)	0.957	77 (33.2)	64 (27.6)	0.226
Respiration	91 (19.6)	37 (14.5)	0.089	53 (22.8)	34 (14.7)	0.032
Cardiovascular	130 (28.0)	72 (28)	0.937	73 (31.5)	68 (29.3)	0.686
Renal	96 (20.6)	71 (27.8)	0.029	52 (22.4)	63 (27.2)	0.282
Site of infection (*n* (%))						
Intestinal	14 (3.0)	11 (4.3)	0.361	9 (3.9)	5 (2.2)	0.416
Urinary	24 (5.2)	31 (12.2)	<0.001	19 (8.2)	20 (8.6)	1.000
Lung	19 (4.1)	27 (10.6)	0.001	15 (6.5)	15 (6.5)	1.000
Catheter	7 (1.5)	12 (4.7)	0.010	6 (2.6)	7 (3.0)	1.000
Skin and soft tissue	25 (5.4)	20 (7.8)	0.191	16 (6.9)	13 (5.6)	0.701
Abdominal cavity	15 (3.2)	18 (7.1)	0.019	12 (5.2)	11 (4.7)	1.000
Microbiology type (*n* (%))						
*Klebsiella*	51 (11.0)	49 (19.2)	0.002	34 (14.7)	37 (15.9)	0.796
*Acinetobacter baumannii*	4 (0.9)	1 (0.4)	0.469	2 (0.9)	1 (0.4)	1.000
*Escherichia coli*	88 (18.9)	74 (29.0)	0.002	61 (26.3)	60 (25.9)	1.000
*Pseudomonas aeruginosa*	32 (6.9)	34 (13.3)	0.004	25 (10.8)	27 (11.6)	0.883
*Staphylococcus aureus*	8 (1.7)	14 (5.5)	0.005	8 (3.4)	7 (3.0)	1.000
*Enterococcus*	180 (38.7)	84 (32.9)	0.124	88 (37.9)	72 (31.0)	0.143
Vital signs, median (IQR)						
Heart rate (bmp)	105 (90–117)	103 (91–118)	0.635	104.5 (90–117)	103.0 (90–118)	0.83
Systolic blood pressure (mmHg)	90 (80–100)	88 (77–101)	0.273	90 (79–100)	88 (77–100)	0.553
Diastolic blood pressure (mmHg)	45 (38–53.5)	45 (37.5–51)	0.321	44 (37–52)	45 (37.9–51)	0.496
Mean arterial pressure (mmHg)	58 (50–66)	56 (49–65)	0.131	57 (50–65)	56 (49–64.3)	0.606
Respiratory rate (bmp)	28 (24–32)	28 (24–32)	0.951	27.5 (24–32)	28 (24–32)	0.932
Temperature (°C)	37.2 (36.9–37.7)	37.2 (36.9–37.7)	0.431	37.2 (36.9–37.7)	37.2 (37–37.7)	0.274
S_P_O_2_ (%)	93 (90–95)	93 (91–95)	0.725	93 (90–95)	93.00 (91–95)	0.889
Laboratory parameters, median (IQR)						
Alanine aminotransferase (IU/L)	40 (20–44)	34 (18–40)	0.057	35 (18.8–41.5)	32.5 (18–40)	0.358
Aspartate aminotransferase (IU/L)	48 (26–55.5)	42 (23–51)	0.114	43 (24.8–54)	41 (23–53)	0.58
Albumin (g/dL)	3.2 (2.6–3.7)	3.2 (2.7–3.8)	0.168	3.2 (2.7–3.7)	3.2 (2.7–3.8)	0.707
Bilirubin (mg/dL)	1.4 (0.5–1.9)	1.3 (0.5–2.1)	0.505	1.3 (0.5–1.9)	1.2 (0.5–2.10)	0.845
Creatinine (mg/dL)	1.3 (0.8–2.1)	1.3 (0.9–2.3)	0.111	1.4 (0.8–2.1)	1.3 (0.9–2.2)	0.472
Blood urea nitrogen (mg/dL)	27 (16–46)	26 (18–40)	0.658	29.5 (18–49)	26.5 (18–40)	0.225
Glucose (mg/dL)	103 (87–126)	107 (93–126)	0.105	102 (87–126)	106 (93.8–126)	0.207
Hemoglobin (g/dL)	9.0 (7.8–10.7)	9.0 (7.9–10.6)	0.934	8.9 (7.8–10.5)	9.0 (7.9–10.7)	0.776
Platelet (×10^9^/L)	140 (82–206)	173 (98–242)	0.001	166 (88.5–230.5)	168 (96.5–237.3)	0.580
Partial thrombin time (s)	37.2 (30.3–48.0)	36.5 (30.4–47.7)	0.822	37.4 (31–47.9)	36.3 (30.5–47.7)	0.287
International normalized ratio	1.6 (1.2–1.9)	1.5 (1.2–1.9)	0.236	1.6 (1.2–1.9)	1.5 (1.2–1.9)	0.418
Prothrombin time (s)	15.3 (12.8–18.1)	16.3 (13.4–22.9)	0.290	17.0 (13.6–22.4)	16.0 (13.4–22.4)	0.394
White blood cell (×10^9^/L)	11.0 (7.7–15.6)	11.0 (7.6–16.2)	0.782	11.3 (7.6–16.1)	11.0 (7.9–16.2)	0.829
Neutrophils (%)	71.6 (66.4–80.7)	71.6 (68–80.9)	0.591	71.6 (65.9–81.4)	71.8 (68.2–81.4)	0.412
Lymphocyte (%)	17.2 (10–20.2)	16.1 (10.1–20)	0.629	17.2 (9–21)	16.1 (10–20)	0.823
Ammonia (*μ*mol/L)	41 (31–63)	21 (16–31)	<0.001	56.5 (40–79.3)	21.5 (17–32)	<0.001
Lactates (mmol/l)	1.8 (1.3–2.5)	1.6 (1.3–2.3)	0.202	1.9 (1.3–2.5)	1.6 (1.3–2.3)	0.167
PaO_2_ (mmHg)	123 (88–132.5)	125 (90–133)	0.525	123 (87–132.8)	125 (90.8–130.3)	0.608
PaCO_2_ (mmHg)	44 (39–46)	45 (41–46)	0.059	44 (39–46)	45 (42–46)	0.042
Analgesic and sedative drugs (*n* (%))						
Midazolam	12 (2.6)	9 (3.5)	0.469	10 (4.3)	7 (3.0)	0.621
Propofol	11 (2.4)	15 (5.9)	0.016	11 (4.7)	10 (4.3)	1.000
Opioids	35 (7.5)	8 (3.1)	0.017	7 (3.0)	8 (3.4)	1.000
Mechanical ventilation (*n* (%))	250 (53.8)	105 (41.2)	0.001	125 (53.9)	95 (40.9)	0.007
Renal replacement therapy (*n* (%))	41 (8.8)	27 (10.6)	0.437	17 (7.3)	21 (9.1)	0.612
Score system						
SAPS II	38 (30–47)	37 (29–45)	0.144	41 (32–49.3)	37 (28–45)	0.001
SOFA	6 (4–10)	5 (3–8)	<0.001	6 (4–10)	5 (3–7)	<0.001
GCS	10 (7–13)	15 (15–15)	<0.001	10 (6–13)	15 (15–15)	<0.001
Use of vasopressors (*n* (%))	182 (39.1)	101 (39.6)	0.902	90 (38.8)	93 (40.1)	0.849
Length of hospital stays, days	4.6 (1.9–11.7)	2.6 (1.3–6.2)	<0.001	4.2 (1.8–10.8)	2.8 (1.3–6.0)	<0.001
Hospital mortality (*n* (%))	96 (20.6)	3 (1.2)	<0.001	42 (18.1)	2 (0.9)	<0.001

PaCO_2_: partial pressure of carbon dioxide; SpO_2_: arterial oxygen saturation; PaO_2_: partial pressure of oxygen; GCS: Glasgow Coma Scale; SAPS II: Simplified Acute Physiology Score; SOFA: Sequential Organ Failure Assessment.

**Table 2:** Multivariate logistic analysis of risk factors in patients with SAE.

	*p*	OR	95.0% CI	
			Lower	Upper
Age	0.154	0.990	0.997	1.004
Coexisting illness (*n* (%))				
Hypertension	0.044	0.581	0.343	0.985
Renal	0.241	0.780	0.514	1.182
Site of infection (*n* (%))				
Urinary	0.357	1.429	0.668	3.056
Lung	0.316	0.645	0.274	1.520
Catheter	0.411	0.609	0.187	1.987
Abdominal cavity	0.609	0.796	0.331	1.911
Microbiology type (*n* (%))				
*Klebsiella*	0.076	0.635	0.385	1.048
*Escherichia coli*	0.014	0.587	0.384	0.897
*Pseudomonas aeruginosa*	0.108	0.625	0.352	1.109
*Staphylococcus aureus*	0.02	0.290	0.102	0.824
Laboratory parameters				
Platelet (×10^9^/L)	0.967	1.000	0.998	1.002
Serum ammonia level ≥45 (*μ*mol/L)	<0.001	3.508	2.336	5.269
Analgesic and sedative drugs (*n* (%))				
Propofol	0.022	0.348	0.141	0.859
Opioids	0.009	3.433	1.360	8.669
Mechanical ventilation (*n* (%))	0.129	1.320	0.922	1.889
Score system				
SOFA	<0.001	1.126	1.062	1.194

SOFA: Sequential Organ Failure Assessment.

**Table 3:** The effect of serum ammonia on the prognosis of patients with SAE.

	≥45 *μ*mol/L serum ammonia level	<45 *μ*mol/L serum ammonia level	*p*
	*n* = 216	*n* = 249	
Mechanical ventilation (*n* (%))	121 (56.8)	129 (51.8)	0.364
Renal replacement therapy (*n* (%))	20 (9.2)	21 (8.4)	0.754
Score system			
SAPS II	40 (32–48)	37 (29–46)	0.095
SOFA	7 (5–10)	4 (6–9)	0.022
GCS	11 (7–13)	10 (6.5–13)	0.551
Length of hospital stays, days	4.2 (2.0–11.6)	4.8 (1.9–12.4)	0.696
Hospital mortality (*n* (%))	45 (20.8)	51 (20.5)	0.926

GCS: Glasgow Coma Scale; SAPS II: Simplified Acute Physiology Score; SOFA: Sequential Organ Failure Assessment.

## Data Availability

The MIMIC IV database (version 0.4) is publically available from https://mimic-iv.mit.edu/. Any researcher who adheres to the data use requirements is permitted access to the database.

## Abbreviations

SAE: Sepsis-associated encephalopathy
PaCO_2_: Partial pressure of carbon dioxide
PaO_2_: Partial pressure of oxygen
SpO_2_: Arterial oxygen saturation
SAPS II: Simplified Acute Physiology Score
SOFA: Sequential Organ Failure Assessment
GCS: Glasgow Coma Scale
ICU: Intensive care unit
MIMIC IV: Medical Information Mart for Intensive Care IV.

## Additional Points

The codes used in the manuscript are available from https://github.com/MIT-LCP/mimic-IV.

## Supplementary Materials

Supplementary Materials Supplementary materials 1: exclude patients with trauma of the skull from the MIMIC IV database according to ICD codes. Supplementary materials 2: exclude patients with intracerebral hemorrhage, cerebral embolism, and ischemic stroke disease from the MIMIC IV database according to ICD codes. Supplementary materials 3: exclude patients with meningitis and encephalitis disease from the MIMIC IV database according to ICD codes. Supplementary materials 4: exclude patients with epilepsy disease from the MIMIC IV database according to ICD codes. Supplementary materials 5: exclude patients with other cerebrovascular disease from the MIMIC IV database according to ICD codes. Supplementary materials 6: exclude patients with mental disorders and neurological disease from the MIMIC IV database according to ICD codes. Supplementary materials 7: exclude patients with alcoholic intoxication or drug abuse from the MIMIC IV database according to ICD codes. Supplementary materials 8: exclude patients with metabolic encephalopathy, hepatic encephalopathy, hypertensive encephalopathy, diabetes with coma, disorders of urea cycle, hypernatremia, and Wernicke's encephalopathy from the MIMIC IV database according to ICD codes. Supplementary materials 9: exclude patients with acute and chronic liver disease. Supplementary materials 10: hypertension disease and ICD codes. Supplementary materials 11: diabetes disease and ICD codes. Supplementary materials 12: lung disease and ICD codes. Supplementary materials 13: cardiovascular diseases and ICD codes. Supplementary materials 14: renal disease from the MIMIC IV database according to ICD codes. Supplementary materials 15: the standardized mean differences of the original cohort were compared with those of the IPW cohorts in sepsis patients. SMD: standardized mean differences.
