# Supplementary material for: Relationship between Nonhepatic Serum Ammonia Levels and Sepsis-Associated Encephalopathy: A Retrospective Cohort Study
Source: Emerg Med Int. 2023 Oct 12;2023:6676033. doi: 10.1155/2023/6676033 (PMC10590267; doi:10.1155/2023/6676033)
Supplement: Supplementary Materials — 1: exclude patients with trauma of the skull from the MIMIC IV database according to ICD codes. Supplementary materials 2: exclude patients with intracerebral hemorrhage, cerebral embolism, and ischemic stroke disease from the MIMIC IV database according to ICD codes. Supplementary materials 3: exclude patients with meningitis and encephalitis disease from the MIMIC IV database according to ICD codes. Supplementary materials 4: exclude patients with epilepsy disease from the MIMIC IV database according to ICD codes. Supplementary materials 5: exclude patients with other cerebrovascular disease from the MIMIC IV database according to ICD codes. Supplementary materials 6: exclude patients with mental disorders and neurological disease from the MIMIC IV database according to ICD codes. Supplementary materials 7: exclude patients with alcoholic intoxication or drug abuse from the MIMIC IV database according to ICD codes. Supplementary materials 8: exclude patients with metabolic encephalopathy, hepatic encephalopathy, hypertensive encephalopathy, diabetes with coma, disorders of urea cycle, hypernatremia, and Wernicke's encephalopathy from the MIMIC IV database according to ICD codes. Supplementary materials 9: exclude patients with acute and chronic liver disease. Supplementary materials 10: hypertension disease and ICD codes. Supplementary materials 11: diabetes disease and ICD codes. Supplementary materials 12: lung disease and ICD codes. Supplementary materials 13: cardiovascular diseases and ICD codes. Supplementary materials 14: renal disease from the MIMIC IV database according to ICD codes. Supplementary materials 15: the standardized mean differences of the original cohort were compared with those of the IPW cohorts in sepsis patients. SMD: standardized mean differences. [file 6676033.f1.zip › Supplementary materials.1.docx]

|  | **Supplementary materials1** Exclude patients with trauma of skull from the MIMIC IV database according to ICD-codes | | | |
| --- | --- | --- | --- | --- |
| ICD-code | | ICD |  | Description |
| 80016 | | ICD9 |  | Closed fracture of vault of skull with cerebral laceration and contusion, with loss of consciousness of unspecified duration |
| 80019 | | ICD9 |  | Closed fracture of vault of skull with cerebral laceration and contusion, with concussion, unspecified |
| 80020 | | ICD9 |  | Closed fracture of vault of skull with subarachnoid, subdural, and extradural hemorrhage, unspecified state of consciousness |
| 80021 | | ICD9 |  | Closed fracture of vault of skull with subarachnoid, subdural, and extradural hemorrhage, with no loss of consciousness |
| 80022 | | ICD9 |  | Closed fracture of vault of skull with subarachnoid, subdural, and extradural hemorrhage, with brief [less than one hour] loss of consciousness |
| 80023 | | ICD9 |  | Closed fracture of vault of skull with subarachnoid, subdural, and extradural hemorrhage, with moderate [1-24 hours] loss of consciousness |
| 80024 | | ICD9 |  | Closed fracture of vault of skull with subarachnoid, subdural, and extradural hemorrhage, with prolonged [more than 24 hours] loss of consciousness and return to pre-existing conscious level |
| 80025 | | ICD9 |  | Closed fracture of vault of skull with subarachnoid, subdural, and extradural hemorrhage, with prolonged [more than 24 hours] loss of consciousness, without return to pre-existing conscious level |
| 80026 | | ICD9 |  | Closed fracture of vault of skull with subarachnoid, subdural, and extradural hemorrhage, with loss of consciousness of unspecified duration |
| 80029 | | ICD9 |  | Closed fracture of vault of skull with subarachnoid, subdural, and extradural hemorrhage, with concussion, unspecified |
| 80030 | | ICD9 |  | Closed fracture of vault of skull with other and unspecified intracranial hemorrhage, unspecified state of consciousness |
| 80031 | | ICD9 |  | Closed fracture of vault of skull with other and unspecified intracranial hemorrhage, with no loss of consciousness |
| 80032 | | ICD9 |  | Closed fracture of vault of skull with other and unspecified intracranial hemorrhage, with brief [less than one hour] loss of consciousness |
| 80033 | | ICD9 |  | Closed fracture of vault of skull with other and unspecified intracranial hemorrhage, with moderate [1-24 hours] loss of consciousness |
| 80034 | | ICD9 |  | Closed fracture of vault of skull with other and unspecified intracranial hemorrhage, with prolonged [more than 24 hours] loss of consciousness and return to pre-existing conscious level |
| 80035 | | ICD9 |  | Closed fracture of vault of skull with other and unspecified intracranial hemorrhage, with prolonged [more than 24 hours] loss of consciousness, without return to pre-existing conscious level |
| 80036 | | ICD9 |  | Closed fracture of vault of skull with other and unspecified intracranial hemorrhage, with loss of consciousness of unspecified duration |
| 80129 | | ICD9 |  | Closed fracture of base of skull with subarachnoid, subdural, and extradural hemorrhage, with concussion, unspecified |
| 80130 | | ICD9 |  | Closed fracture of base of skull with other and unspecified intracranial hemorrhage, unspecified state of consciousness |
| 80131 | | ICD9 |  | Closed fracture of base of skull with other and unspecified intracranial hemorrhage, with no loss of consciousness |
| 80132 | | ICD9 |  | Closed fracture of base of skull with other and unspecified intracranial hemorrhage, with brief [less than one hour] loss of consciousness |
| 80133 | | ICD9 |  | Closed fracture of base of skull with other and unspecified intracranial hemorrhage, with moderate [1-24 hours] loss of consciousness |
| 80134 | | ICD9 |  | Closed fracture of base of skull with other and unspecified intracranial hemorrhage, with prolonged [more than 24 hours] loss of consciousness and return to pre-existing conscious level |
| 80135 | | ICD9 |  | Closed fracture of base of skull with other and unspecified intracranial hemorrhage, with prolonged [more than 24 hours] loss of consciousness, without return to pre-existing conscious level |
| 80136 | | ICD9 |  | Closed fracture of base of skull with other and unspecified intracranial hemorrhage, with loss of consciousness of unspecified duration |
| 80139 | | ICD9 |  | Closed fracture of base of skull with other and unspecified intracranial hemorrhage, with concussion, unspecified |
| 80140 | | ICD9 |  | Closed fracture of base of skull with intracranial injury of other and unspecified nature, unspecified state of consciousness |
| 80141 | | ICD9 |  | Closed fracture of base of skull with intracranial injury of other and unspecified nature, with no loss of consciousness |
| 80142 | | ICD9 |  | Closed fracture of base of skull with intracranial injury of other and unspecified nature, with brief [less than one hour] loss of consciousness |
| 80143 | | ICD9 |  | Closed fracture of base of skull with intracranial injury of other and unspecified nature, with moderate [1-24 hours] loss of consciousness |
| 80144 | | ICD9 |  | Closed fracture of base of skull with intracranial injury of other and unspecified nature, with prolonged [more than 24 hours) loss of consciousness and return to pre-existing conscious level |
| 80145 | | ICD9 |  | Closed fracture of base of skull with intracranial injury of other and unspecified nature, with prolonged [more than 24 hours] loss of consciousness, without return to pre-existing conscious level |
| 80146 | | ICD9 |  | Closed fracture of base of skull with intracranial injury of other and unspecified nature, with loss of consciousness of unspecified duration |
| 80149 | | ICD9 |  | Closed fracture of base of skull with intracranial injury of other and unspecified nature, with concussion, unspecified |
| 80150 | | ICD9 |  | Open fracture of base of skull without mention of intracranial injury, unspecified state of consciousness |
| 80151 | | ICD9 |  | Open fracture of base of skull without mention of intracranial injury, with no loss of consciousness |
| 80152 | | ICD9 |  | Open fracture of base of skull without mention of intracranial injury, with brief [less than one hour] loss of consciousness |
| 80153 | | ICD9 |  | Open fracture of base of skull without mention of intracranial injury, with moderate [1-24 hours] loss of consciousness |
| 80154 | | ICD9 |  | Open fracture of base of skull without mention of intracranial injury, with prolonged [more than 24 hours] loss of consciousness and return to pre-existing conscious level |
| 80155 | | ICD9 |  | Open fracture of base of skull without mention of intracranial injury, with prolonged [more than 24 hours] loss of consciousness, without return to pre-existing conscious level |
| 80156 | | ICD9 |  | Open fracture of base of skull without mention of intracranial injury, with loss of consciousness of unspecified duration |
| 80159 | | ICD9 |  | Open fracture of base of skull without mention of intracranial injury, with concussion, unspecified |
| 80160 | | ICD9 |  | Open fracture of base of skull with cerebral laceration and contusion, unspecified state of consciousness |
| 80161 | | ICD9 |  | Open fracture of base of skull with cerebral laceration and contusion, with no loss of consciousness |
| 80162 | | ICD9 |  | Open fracture of base of skull with cerebral laceration and contusion, with brief [less than one hour] loss of consciousness |
| 80163 | | ICD9 |  | Open fracture of base of skull with cerebral laceration and contusion, with moderate [1-24 hours] loss of consciousness |
| 80164 | | ICD9 |  | Open fracture of base of skull with cerebral laceration and contusion, with prolonged [more than 24 hours] loss of consciousness and return to pre-existing conscious level |
| 80165 | | ICD9 |  | Open fracture of base of skull with cerebral laceration and contusion, with prolonged [more than 24 hours] loss of consciousness, without return to pre-existing conscious level |
| 80166 | | ICD9 |  | Open fracture of base of skull with cerebral laceration and contusion, with loss of consciousness of unspecified duration |
| 80169 | | ICD9 |  | Open fracture of base of skull with cerebral laceration and contusion, with concussion, unspecified |
| 80170 | | ICD9 |  | Open fracture of base of skull with subarachnoid, subdural, and extradural hemorrhage, unspecified state of consciousness |
| 80171 | | ICD9 |  | Open fracture of base of skull with subarachnoid, subdural, and extradural hemorrhage, with no loss of consciousness |
| 80172 | | ICD9 |  | Open fracture of base of skull with subarachnoid, subdural, and extradural hemorrhage, with brief [less than one hour] loss of consciousness |
| 80173 | | ICD9 |  | Open fracture of base of skull with subarachnoid, subdural, and extradural hemorrhage, with moderate [1-24 hours] loss of consciousness |
| 85154 | | ICD9 |  | Cerebellar or brain stem contusion with open intracranial wound, with prolonged [more than 24 hours] loss of consciousness and return |
| 85155 | | ICD9 |  | Cerebellar or brain stem contusion with open intracranial wound, with prolonged [more than 24 hours] loss of consciousness without return to pre-existing conscious level |
| 85156 | | ICD9 |  | Cerebellar or brain stem contusion with open intracranial wound, with loss of consciousness of unspecified duration |
| 85159 | | ICD9 |  | Cerebellar or brain stem contusion with open intracranial wound, with concussion, unspecified |
| 85160 | | ICD9 |  | Cerebellar or brain stem laceration without mention of open intracranial wound, unspecified state of consciousness |
| 85161 | | ICD9 |  | Cerebellar or brain stem laceration without mention of open intracranial wound, with no loss of consciousness |
| 85162 | | ICD9 |  | Cerebellar or brain stem laceration without mention of open intracranial wound, with brief [less than 1 hour] loss of consciousness |
| 85163 | | ICD9 |  | Cerebellar or brain stem laceration without mention of open intracranial wound, with moderate [1-24 hours] loss of consciousness |
| 85164 | | ICD9 |  | Cerebellar or brain stem laceration without mention of open intracranial wound, with prolonged [more than 24 hours] loss of consciousness and return to pre-existing conscious level |
| 85165 | | ICD9 |  | Cerebellar or brain stem laceration without mention of open intracranial wound, with prolonged [more than 24 hours] loss of consciousness without return to pre-existing conscious level |
| 85166 | | ICD9 |  | Cerebellar or brain stem laceration without mention of open intracranial wound, with loss of consciousness of unspecified duration |
| 85169 | | ICD9 |  | Cerebellar or brain stem laceration without mention of open intracranial wound, with concussion, unspecified |
| 85170 | | ICD9 |  | Cerebellar or brain stem laceration with open intracranial wound, unspecified state of consciousness |
| 85171 | | ICD9 |  | Cerebellar or brain stem laceration with open intracranial wound, with no loss of consciousness |
| 85172 | | ICD9 |  | Cerebellar or brain stem laceration with open intracranial wound, with brief [less than one hour] loss of consciousness |
| 85173 | | ICD9 |  | Cerebellar or brain stem laceration with open intracranial wound, with moderate [1-24 hours] loss of consciousness |
| 85174 | | ICD9 |  | Cerebellar or brain stem laceration with open intracranial wound, with prolonged [more than 24 hours] loss of consciousness and return to pre-existing conscious level |
| 85175 | | ICD9 |  | Cerebellar or brain stem laceration with open intracranial wound, with prolonged [more than 24 hours] loss of consciousness without return to pre-existing conscious level |
| 85176 | | ICD9 |  | Cerebellar or brain stem laceration with open intracranial wound, with loss of consciousness of unspecified duration |
| 85179 | | ICD9 |  | Cerebellar or brain stem laceration with open intracranial wound, with concussion, unspecified |
| 85180 | | ICD9 |  | Other and unspecified cerebral laceration and contusion, without mention of open intracranial wound, unspecified state of consciousness |
| 85181 | | ICD9 |  | Other and unspecified cerebral laceration and contusion, without mention of open intracranial wound, with no loss of consciousness |
| 85182 | | ICD9 |  | Other and unspecified cerebral laceration and contusion, without mention of open intracranial wound, with brief [less than one hour] loss of consciousness |
| 85183 | | ICD9 |  | Other and unspecified cere85174bral laceration and contusion, without mention of open intracranial wound, with moderate [1-24 hours] loss of consciousness |
| 85184 | | ICD9 |  | Other and unspecified cerebral laceration and contusion, without mention of open intracranial wound, with prolonged [more than 24 hours] loss of consciousness and return to pre- existing conscious level |
| 85185 | | ICD9 |  | Other and unspecified cerebral laceration and contusion, without mention of open intracranial wound, with prolonged [more than 24 hours] loss of consciousness without return to pre-existing conscious level |
| 85186 | | ICD9 |  | Other and unspecified cerebral laceration and contusion, without mention of open intracranial wound, with loss of consciousness of unspecified duration |
| 85189 | | ICD9 |  | Other and unspecified cerebral laceration and contusion, without mention of open intracranial wound, with concussion, unspecified |
| 85190 | | ICD9 |  | Other and unspecified cerebral laceration and contusion, with open intracranial wound, unspecified state of consciousness |
| 85191 | | ICD9 |  | Other and unspecified cerebral laceration and contusion, with open intracranial wound, with no loss of consciousness |
| 85192 | | ICD9 |  | Other and unspecified cerebral laceration and contusion, with open intracranial wound, with brief [less than one hour] loss of consciousness |
| 80039 | | ICD9 |  | Closed fracture of vault of skull with other and unspecified intracranial hemorrhage, with concussion, unspecified |
| 80040 | | ICD9 |  | Closed fracture of vault of skull with intracranial injury of other and unspecified nature, unspecified state of consciousness |
| 80041 | | ICD9 |  | Closed fracture of vault of skull with intracranial injury of other and unspecified nature, with no loss of consciousness |
| 80042 | | ICD9 |  | Closed fracture of vault of skull with intracranial injury of other and unspecified nature, with brief [less than one hour] loss of consciousness |
| 80043 | | ICD9 |  | Closed fracture of vault of skull with intracranial injury of other and unspecified nature, with moderate [1-24 hours] loss of consciousness |
| 80044 | | ICD9 |  | Closed fracture of vault of skull with intracranial injury of other and unspecified nature, with prolonged [more than 24 hours] loss of consciousness and return to pre-existing conscious level |
| 80045 | | ICD9 |  | Closed fracture of vault of skull with intracranial injury of other and unspecified nature, with prolonged [more than 24 hours] loss of consciousness, without return to pre-existing conscious level |
| 80046 | | ICD9 |  | Closed fracture of vault of skull with intracranial injury of other and unspecified nature, with loss of consciousness of unspecified duration |
| 80049 | | ICD9 |  | Closed fracture of vault of skull with intracranial injury of other and unspecified nature, with concussion, unspecified |
| 80050 | | ICD9 |  | Open fracture of vault of skull without mention of intracranial injury, unspecified state of consciousness |
| 80051 | | ICD9 |  | Open fracture of vault of skull without mention of intracranial injury, with no loss of consciousness |
| 80052 | | ICD9 |  | Open fracture of vault of skull without mention of intracranial injury, with brief [less than one hour] loss of consciousness |
| 80053 | | ICD9 |  | Open fracture of vault of skull without mention of intracranial injury, with moderate [1-24 hours] loss of consciousness |
| 80054 | | ICD9 |  | Open fracture of vault of skull without mention of intracranial injury, with prolonged [more than 24 hours] loss of consciousness and return to pre-existing conscious level |
| 80055 | | ICD9 |  | Open fracture of vault of skull without mention of intracranial injury, with prolonged [more than 24 hours] loss of consciousness, without return to pre-existing conscious level |
| 80056 | | ICD9 |  | Open fracture of vault of skull without mention of intracranial injury, with loss of consciousness of unspecified duration |
| 80059 | | ICD9 |  | Open fracture of vault of skull without mention of intracranial injury, with concussion, unspecified |
| 80060 | | ICD9 |  | Open fracture of vault of skull with cerebral laceration and contusion, unspecified state of consciousness |
| 80061 | | ICD9 |  | Open fracture of vault of skull with cerebral laceration and contusion, with no loss of consciousness |
| 80062 | | ICD9 |  | Open fracture of vault of skull with cerebral laceration and contusion, with brief [less than one hour] loss of consciousness |
| 80063 | | ICD9 |  | Open fracture of vault of skull with cerebral laceration and contusion, with moderate [1-24 hours] loss of consciousness |
| 80064 | | ICD9 |  | Open fracture of vault of skull with cerebral laceration and contusion, with prolonged [more than 24 hours] loss of consciousness and return to pre-existing conscious level |
| 80065 | | ICD9 |  | Open fracture of vault of skull with cerebral laceration and contusion, with prolonged [more than 24 hours] loss of consciousness, without return to pre-existing conscious level |
| 80066 | | ICD9 |  | Open fracture of vault of skull with cerebral laceration and contusion, with loss of consciousness of unspecified duration |
| 80069 | | ICD9 |  | Open fracture of vault of skull with cerebral laceration and contusion, with concussion, unspecified |
| 80070 | | ICD9 |  | Open fracture of vault of skull with subarachnoid, subdural, and extradural hemorrhage, unspecified state of consciousness |
| 80071 | | ICD9 |  | Open fracture of vault of skull with subarachnoid, subdural, and extradural hemorrhage, with no loss of consciousness |
| 80072 | | ICD9 |  | Open fracture of vault of skull with subarachnoid, subdural, and extradural hemorrhage, with brief [less than one hour] loss of consciousness |
| 80073 | | ICD9 |  | Open fracture of vault of skull with subarachnoid, subdural, and extradural hemorrhage, with moderate [1-24 hours] loss of consciousness |
| 80074 | | ICD9 |  | Open fracture of vault of skull with subarachnoid, subdural, and extradural hemorrhage, with moderate [1-24 hours] loss of consciousness |
| 80075 | | ICD9 |  | Open fracture of vault of skull with subarachnoid, subdural, and extradural hemorrhage, with prolonged [more than 24 hours] loss of consciousness, without return to pre-existing conscious level |
| 80076 | | ICD9 |  | Open fracture of vault of skull with subarachnoid, subdural, and extradural hemorrhage, with loss of consciousness of unspecified duration |
| 80079 | | ICD9 |  | Open fracture of vault of skull with subarachnoid, subdural, and extradural hemorrhage, with concussion, unspecified |
| 80080 | | ICD9 |  | Open fracture of vault of skull with other and unspecified intracranial hemorrhage, unspecified state of consciousness |
| 80081 | | ICD9 |  | Open fracture of vault of skull with other and unspecified intracranial hemorrhage, with no loss of consciousness |
| 80082 | | ICD9 |  | Open fracture of vault of skull with other and unspecified intracranial hemorrhage, with brief [less than one hour] loss of |
| 80083 | | ICD9 |  | Open fracture of vault of skull with other and unspecified intracranial hemorrhage, with moderate [1-24 hours] loss of consciousness |
| 80084 | | ICD9 |  | Open fracture of vault of skull with other and unspecified intracranial hemorrhage, with prolonged [more than 24 hours] loss of consciousness and return to pre-existing conscious level |
| 80085 | | ICD9 |  | Open fracture of vault of skull with other and unspecified intracranial hemorrhage, with prolonged [more than 24 hours] loss of consciousness, without return to pre-existing conscious level |
| 80086 | | ICD9 |  | Open fracture of vault of skull with other and unspecified intracranial hemorrhage, with loss of consciousness of unspecified duration |
| 80089 | | ICD9 |  | Open fracture of vault of skull with other and unspecified intracranial hemorrhage, with concussion, unspecified |
| 80090 | | ICD9 |  | Open fracture of vault of skull with intracranial injury of other and unspecified nature, unspecified state of consciousness |
| 80091 | | ICD9 |  | Open fracture of vault of skull with intracranial injury of other and unspecified nature, with no loss of consciousness |
| 80092 | | ICD9 |  | Open fracture of vault of skull with intracranial injury of other and unspecified nature, with brief [less than one hour] loss of consciousness |
| 80093 | | ICD9 |  | Open fracture of vault of skull with intracranial injury of other and unspecified nature, with moderate [1-24 hours] loss of consciousness |
| 80094 | | ICD9 |  | Open fracture of vault of skull with intracranial injury of other and unspecified nature, with prolonged [more than 24 hours] loss of consciousness and return to pre-existing conscious level |
| 80095 | | ICD9 |  | Open fracture of vault of skull with intracranial injury of other and unspecified nature, with prolonged [more than 24 hours] loss of consciousness, without return to pre-existing conscious level |
| 80096 | | ICD9 |  | Open fracture of vault of skull with intracranial injury of other and unspecified nature, with loss of consciousness of unspecified duration |
| 80099 | | ICD9 |  | Open fracture of vault of skull with intracranial injury of other and unspecified nature, with concussion, unspecified |
| 80100 | | ICD9 |  | Closed fracture of base of skull without mention of intra cranial injury, unspecified state of consciousness |
| 80101 | | ICD9 |  | Closed fracture of base of skull without mention of intra cranial injury, with no loss of consciousness |
| 80102 | | ICD9 |  | Closed fracture of base of skull without mention of intra cranial injury, with brief [less than one hour] loss of consciousness |
| 80103 | | ICD9 |  | Closed fracture of base of skull without mention of intra cranial injury, with moderate [1-24 hours] loss of consciousness |
| 80104 | | ICD9 |  | Closed fracture of base of skull without mention of intra cranial injury, with prolonged [more than 24 hours] loss of consciousness and return to pre-existing conscious level |
| 80105 | | ICD9 |  | Closed fracture of base of skull without mention of intra cranial injury, with prolonged [more than 24 hours] loss of consciousness, without return to pre-existing conscious level |
| 80106 | | ICD9 |  | Closed fracture of base of skull without mention of intra cranial injury, with loss of consciousness of unspecified duration |
| 80109 | | ICD9 |  | Closed fracture of base of skull without mention of intra cranial injury, with concussion, unspecified |
| 80110 | | ICD9 |  | Closed fracture of base of skull with cerebral laceration and contusion, unspecified state of consciousness |
| 80111 | | ICD9 |  | Closed fracture of base of skull with cerebral laceration and contusion, with no loss of consciousness |
| 80112 | | ICD9 |  | Closed fracture of base of skull with cerebral laceration and contusion, with brief [less than one hour] loss of consciousness |
| 80113 | | ICD9 |  | Closed fracture of base of skull with cerebral laceration and contusion, with moderate [1-24 hours] loss of consciousness |
| 80114 | | ICD9 |  | Closed fracture of base of skull with cerebral laceration and contusion, with prolonged [more than 24 hours] loss of consciousness and return to pre-existing conscious level |
| 80115 | | ICD9 |  | Closed fracture of base of skull with cerebral laceration and contusion, with prolonged [more than 24 hours] loss of consciousness, without return to pre-existing conscious level |
| 80116 | | ICD9 |  | Closed fracture of base of skull with cerebral laceration and contusion, with loss of consciousness of unspecified duration |
| 80119 | | ICD9 |  | Closed fracture of base of skull with cerebral laceration and contusion, with concussion, unspecified |
| 80120 | | ICD9 |  | Closed fracture of base of skull with subarachnoid, subdural, and extradural hemorrhage, unspecified state of consciousness |
| 80121 | | ICD9 |  | Closed fracture of base of skull with subarachnoid, subdural, and extradural hemorrhage, with no loss of consciousness |
| 80122 | | ICD9 |  | Closed fracture of base of skull with subarachnoid, subdural, and extradural hemorrhage, with brief [less than one hour] loss of consciousness |
| 80123 | | ICD9 |  | Closed fracture of base of skull with subarachnoid, subdural, and extradural hemorrhage, with moderate [1-24 hours] loss of consciousness |
| 80124 | | ICD9 |  | Closed fracture of base of skull with subarachnoid, subdural, and extradural hemorrhage, with prolonged [more than 24 hours] loss of consciousness and return to pre-existing conscious level |
| 80300 | | ICD9 |  | Other closed skull fracture without mention of intracranial injury, unspecified state of consciousness |
| 80301 | | ICD9 |  | Other closed skull fracture without mention of intracranial injury, with no loss of consciousness |
| 80302 | | ICD9 |  | Other closed skull fracture without mention of intracranial injury, with brief [less than one hour] loss of consciousness |
| 80303 | | ICD9 |  | Other closed skull fracture without mention of intracranial injury, with moderate [1-24 hours] loss of consciousness |
| 80304 | | ICD9 |  | Other closed skull fracture without mention of intracranial injury, with prolonged [more than 24 hours] loss of consciousness and return to pre-existing conscious level |
| 80305 | | ICD9 |  | Other closed skull fracture without mention of intracranial injury, with prolonged [more than 24 hours] loss of consciousness, without return to pre-existing conscious level |
| 80306 | | ICD9 |  | Other closed skull fracture without mention of intracranial injury, with loss of consciousness of unspecified duration |
| 80309 | | ICD9 |  | Other closed skull fracture without mention of intracranial injury, with concussion, unspecified |
| 80310 | | ICD9 |  | Other closed skull fracture with cerebral laceration and contusion, unspecified state of consciousness |
| 80311 | | ICD9 |  | Other closed skull fracture with cerebral laceration and contusion, with no loss of consciousness |
| 80312 | | ICD9 |  | Other closed skull fracture with cerebral laceration and contusion, with brief [less than one hour] loss of consciousness |
| 80313 | | ICD9 |  | Other closed skull fracture with cerebral laceration and contusion, with moderate [1-24 hours] loss of consciousness |
| 80314 | | ICD9 |  | Other closed skull fracture with cerebral laceration and contusion, with prolonged [more than 24 hours] loss of consciousness and return to pre-existing conscious level |
| 80315 | | ICD9 |  | Other closed skull fracture with cerebral laceration and contusion, with prolonged [more than 24 hours] loss of consciousness, without return to pre-existing conscious level |
| 80316 | | ICD9 |  | Other closed skull fracture with cerebral laceration and contusion, with loss of consciousness of unspecified duration |
| 80319 | | ICD9 |  | Other closed skull fracture with cerebral laceration and contusion, with concussion, unspecified |
| 80320 | | ICD9 |  | Other closed skull fracture with subarachnoid, subdural, and extradural hemorrhage, unspecified state of consciousness |
| 80321 | | ICD9 |  | Other closed skull fracture with subarachnoid, subdural, and extradural hemorrhage, with no loss of consciousness |
| 80322 | | ICD9 |  | Other closed skull fracture with subarachnoid, subdural, and extradural hemorrhage, with brief [less than one hour] loss of consciousness |
| 80323 | | ICD9 |  | Other closed skull fracture with subarachnoid, subdural, and extradural hemorrhage, with moderate [1-24 hours] loss of consciousness |
| 80324 | | ICD9 |  | Other closed skull fracture with subarachnoid, subdural, and extradural hemorrhage, with prolonged [more than 24 hours] loss of consciousness and return to pre-existing conscious level |
| 80325 | | ICD9 |  | Other closed skull fracture with subarachnoid, subdural, and extradural hemorrhage, with prolonged [more than 24 hours] loss of consciousness, without return to pre-existing conscious level |
| 80326 | | ICD9 |  | Other closed skull fracture with subarachnoid, subdural, and extradural hemorrhage, with loss of consciousness of unspecified duration |
| 80329 | | ICD9 |  | Other closed skull fracture with subarachnoid, subdural, and extradural hemorrhage, with concussion, unspecified |
| 80330 | | ICD9 |  | Other closed skull fracture with other and unspecified intracranial hemorrhage, unspecified state of unconsciousness |
| 80331 | | ICD9 |  | Other closed skull fracture with other and unspecified intracranial hemorrhage, with no loss of consciousness |
| 80332 | | ICD9 |  | Other closed skull fracture with other and unspecified intracranial hemorrhage, with brief [less than one hour] loss of consciousness |
| 80333 | | ICD9 |  | Other closed skull fracture with other and unspecified intracranial hemorrhage, with moderate [1-24 hours] loss of consciousness |
| 80334 | | ICD9 |  | Other closed skull fracture with other and unspecified intracranial hemorrhage, with prolonged [more than 24 hours] loss of consciousness and return to pre-existing conscious level |
| 80335 | | ICD9 |  | Other closed skull fracture with other and unspecified intracranial hemorrhage, with prolonged [more than 24 hours] loss of consciousness, without return to pre-existing conscious level |
| 80336 | | ICD9 |  | Other closed skull fracture with other and unspecified intracranial hemorrhage, with loss of consciousness of unspecified duration |
| 80339 | | ICD9 |  | Other closed skull fracture with other and unspecified intracranial hemorrhage, with concussion, unspecified |
| 80340 | | ICD9 |  | Other closed skull fracture with intracranial injury of other and unspecified nature, unspecified state of consciousness |
| 80341 | | ICD9 |  | Other closed skull fracture with intracranial injury of other and unspecified nature, with no loss of consciousness |
| 80342 | | ICD9 |  | Other closed skull fracture with intracranial injury of other and unspecified nature, with brief [less than one hour] loss of consciousness |
| 80343 | | ICD9 |  | Other closed skull fracture with intracranial injury of other and unspecified nature, with moderate [1-24 hours] loss of consciousness |
| 80344 | | ICD9 |  | Other closed skull fracture with intracranial injury of other and unspecified nature, with prolonged [more than 24 hours] loss of consciousness and return to pre-existing conscious level |
| 80345 | | ICD9 |  | Other closed skull fracture with intracranial injury of other and unspecified nature, with prolonged [more than 24 hours] loss of consciousness, without return to pre-existing conscious level |
| 8503 | | ICD9 |  | Concussion with prolonged loss of consciousness and return to pre-existing conscious level |
| 85193 | | ICD9 |  | Other and unspecified cerebral laceration and contusion, with open intracranial wound, with moderate [1-24 hours] loss of consciousness |
| 85194 | | ICD9 |  | Other and unspecified cerebral laceration and contusion, with open intracranial wound, with prolonged [more than 24 hours] loss of consciousness and return to pre-existing conscious level |
| 85195 | | ICD9 |  | Other and unspecified cerebral laceration and contusion, with open intracranial wound, with prolonged [more than 24 hours] loss of consciousness without return to pre-existing conscious level |
| 85196 | | ICD9 |  | Other and unspecified cerebral laceration and contusion, with open intracranial wound, with loss of consciousness of unspecified duration |
| 85199 | | ICD9 |  | Other and unspecified cerebral laceration and contusion, with open intracranial wound, with concussion, unspecified |
| 85200 | | ICD9 |  | Subarachnoid hemorrhage following injury without mention of open intracranial wound, unspecified state of consciousness |
| 85201 | | ICD9 |  | Subarachnoid hemorrhage following injury without mention of open intracranial wound, with no loss of consciousness |
| 85412 | | ICD9 |  | Intracranial injury of other and unspecified nature with open intracranial wound, with brief [less than one hour] loss of consciousness |
| 85413 | | ICD9 |  | Intracranial injury of other and unspecified nature with open intracranial wound, with moderate [1-24 hours] loss of consciousness |
| 85414 | | ICD9 |  | Intracranial injury of other and unspecified nature with open intracranial wound, with prolonged [more than 24 hours] loss of consciousness and return to pre-existing conscious level |
| 85415 | | ICD9 |  | Intracranial injury of other and unspecified nature with open intracranial wound, with prolonged [more than 24 hours] loss of consciousness without return to pre-existing conscious level |
| 85416 | | ICD9 |  | Intracranial injury of other and unspecified nature with open intracranial wound, with loss of consciousness of unspecified duration |
| 85419 | | ICD9 |  | Intracranial injury of other and unspecified nature with open intracranial wound, with concussion, unspecified |
| 80411 | | ICD9 |  | Closed fractures involving skull or face with other bones, with cerebral laceration and contusion, with no loss of consciousness |
| 80420 | | ICD9 |  | Closed fractures involving skull or face with other bones with subarachnoid, subdural, and extradural hemorrhage, unspecified state of consciousness |
| 80421 | | ICD9 |  | Closed fractures involving skull or face with other bones with subarachnoid, subdural, and extradural hemorrhage, with no loss of consciousness |
| 80422 | | ICD9 |  | Closed fractures involving skull or face with other bones with subarachnoid, subdural, and extradural hemorrhage, with brief [less than one hour] loss of consciousness |
| 80423 | | ICD9 |  | Closed fractures involving skull or face with other bones with subarachnoid, subdural, and extradural hemorrhage, with moderate [1-24 hours] loss of consciousness |
| 80424 | | ICD9 |  | Closed fractures involving skull or face with other bones with subarachnoid, subdural, and extradural hemorrhage, with prolonged [more than 24 hours] loss of consciousness and return to pre-existing conscious level |
| 80425 | | ICD9 |  | Closed fractures involving skull or face with other bones with subarachnoid, subdural, and extradural hemorrhage, with prolonged [more than 24 hours] loss of consciousness, without return to pre-existing conscious level |
| 80426 | | ICD9 |  | Closed fractures involving skull or face with other bones with subarachnoid, subdural, and extradural hemorrhage, with loss of consciousness of unspecified duration |
| 80429 | | ICD9 |  | Closed fractures involving skull or face with other bones with subarachnoid, subdural, and extradural hemorrhage, with concussion, unspecified |
| 80430 | | ICD9 |  | Closed fractures involving skull or face with other bones, with other and unspecified intracranial hemorrhage, unspecified state of consciousness |
| 80431 | | ICD9 |  | Closed fractures involving skull or face with other bones, with other and unspecified intracranial hemorrhage, with no loss of consciousness |
| 80432 | | ICD9 |  | Closed fractures involving skull or face with other bones, with other and unspecified intracranial hemorrhage, with brief [less than one hour] loss of consciousness |
| 80433 | | ICD9 |  | Closed fractures involving skull or face with other bones, with other and unspecified intracranial hemorrhage, with moderate [1-24 hours] loss of consciousness |
| 85250 | | ICD9 |  | Extradural hemorrhage following injury with open intracranial wound, unspecified state of consciousness |
| 85251 | | ICD9 |  | Extradural hemorrhage following injury with open intracranial wound, with no loss of consciousness |
| 85252 | | ICD9 |  | Extradural hemorrhage following injury with open intracranial wound, with brief [less than one hour] loss of consciousness |
| 85253 | | ICD9 |  | Extradural hemorrhage following injury with open intracranial wound, with moderate [1-24 hours] loss of consciousness |
| 85254 | | ICD9 |  | Extradural hemorrhage following injury with open intracranial wound, with prolonged [more than 24 hours] loss of consciousness and return to pre-existing conscious level |
| 85255 | | ICD9 |  | Extradural hemorrhage following injury with open intracranial wound, with prolonged [more than 24 hours] loss of consciousness without return to pre-existing conscious level |
| 85256 | | ICD9 |  | Extradural hemorrhage following injury with open intracranial wound, with loss of consciousness of unspecified duration |
| 85259 | | ICD9 |  | Extradural hemorrhage following injury with open intracranial wound, with concussion, unspecified |
| 85300 | | ICD9 |  | Other and unspecified intracranial hemorrhage following injury without mention of open intracranial wound, unspecified state of consciousness |
| 85301 | | ICD9 |  | Other and unspecified intracranial hemorrhage following injury without mention of open intracranial wound, with no loss of consciousness |
| 85302 | | ICD9 |  | Other and unspecified intracranial hemorrhage following injury without mention of open intracranial wound, with brief [less than one hour] loss of consciousness |
| 85303 | | ICD9 |  | Other and unspecified intracranial hemorrhage following injury without mention of open intracranial wound, with moderate [1-24 hours] loss of consciousness |
| 85304 | | ICD9 |  | Other and unspecified intracranial hemorrhage following injury without mention of open intracranial wound, with prolonged [more than 24 hours] loss of consciousness and return to pre- existing conscious level |
| 85305 | | ICD9 |  | Other and unspecified intracranial hemorrhage following injury without mention of open intracranial wound, with prolonged [more than 24 hours] loss of consciousness without return to pre-existing conscious level |
| 85306 | | ICD9 |  | Other and unspecified intracranial hemorrhage following injury without mention of open intracranial wound, with loss of consciousness of unspecified duration |
| 85309 | | ICD9 |  | Other and unspecified intracranial hemorrhage following injury without mention of open intracranial wound, with concussion, unspecified |
| 85310 | | ICD9 |  | Other and unspecified intracranial hemorrhage following injury with open intracranial wound, unspecified state of consciousness |
| 85311 | | ICD9 |  | Other and unspecified intracranial hemorrhage following injury with open intracranial wound, with no loss of consciousness |
| 85312 | | ICD9 |  | Other and unspecified intracranial hemorrhage following injury with open intracranial wound, with brief [less than one hour] loss of consciousness |
| 85313 | | ICD9 |  | Other and unspecified intracranial hemorrhage following injury with open intracranial wound, with moderate [1-24 hours] loss of consciousness |
| 85314 | | ICD9 |  | Other and unspecified intracranial hemorrhage following injury with open intracranial wound, with prolonged [more than 24 hours] loss of consciousness and return to pre-existing conscious level |
| 85315 | | ICD9 |  | Other and unspecified intracranial hemorrhage following injury with open intracranial wound, with prolonged [more than 24 hours] loss of consciousness without return to pre-existing conscious level |
| 85316 | | ICD9 |  | Other and unspecified intracranial hemorrhage following injury with open intracranial wound, with loss of consciousness of unspecified duration |
| 85319 | | ICD9 |  | Other and unspecified intracranial hemorrhage following injury with open intracranial wound, with concussion, unspecified |
| 85400 | | ICD9 |  | Intracranial injury of other and unspecified nature without mention of open intracranial wound, unspecified state of consciousness |
| 85401 | | ICD9 |  | Intracranial injury of other and unspecified nature without mention of open intracranial wound, with no loss of consciousness |
| 85402 | | ICD9 |  | Intracranial injury of other and unspecified nature without mention of open intracranial wound, with brief [less than one hour] loss of consciousness |
| 85403 | | ICD9 |  | Intracranial injury of other and unspecified nature without mention of open intracranial wound, with moderate [1-24 hours] loss of consciousness |
| 85404 | | ICD9 |  | Intracranial injury of other and unspecified nature without mention of open intracranial wound, with prolonged [more than 24 hours] loss of consciousness and return to pre-existing conscious level |
| 85405 | | ICD9 |  | Intracranial injury of other and unspecified nature without mention of open intracranial wound, with prolonged [more than 24 hours] loss of consciousness without return to pre-existing conscious level |
| 85406 | | ICD9 |  | Intracranial injury of other and unspecified nature without mention of open intracranial wound, with loss of consciousness of unspecified duration |
| 85409 | | ICD9 |  | Intracranial injury of other and unspecified nature without mention of open intracranial wound, with concussion, unspecified |
| 85410 | | ICD9 |  | Intracranial injury of other and unspecified nature with open intracranial wound, unspecified state of consciousness |
| 85411 | | ICD9 |  | Intracranial injury of other and unspecified nature with open intracranial wound, with no loss of consciousness |
| 85412 | | ICD9 |  | Intracranial injury of other and unspecified nature with open intracranial wound, with brief [less than one hour] loss of consciousness |
| 85413 | | ICD9 |  | Intracranial injury of other and unspecified nature with open intracranial wound, with moderate [1-24 hours] loss of consciousness |
| 85414 | | ICD9 |  | Intracranial injury of other and unspecified nature with open intracranial wound, with prolonged [more than 24 hours] loss of consciousness and return to pre-existing conscious level |
| 85415 | | ICD9 |  | Intracranial injury of other and unspecified nature with open intracranial wound, with prolonged [more than 24 hours] loss of consciousness without return to pre-existing conscious level |
| 85416 | | ICD9 |  | Intracranial injury of other and unspecified nature with open intracranial wound, with loss of consciousness of unspecified duration |
| 85419 | | ICD9 |  | Intracranial injury of other and unspecified nature with open intracranial wound, with concussion, unspecified |
| 80434 | | ICD9 |  | Closed fractures involving skull or face with other bones, with other and unspecified intracranial hemorrhage, with prolonged [more than 24 hours] loss of consciousness and return to pre- existing conscious level |
| 80435 | | ICD9 |  | Closed fractures involving skull or face with other bones, with other and unspecified intracranial hemorrhage, with prolonged [more than 24 hours] loss of consciousness, without return to pre-existing conscious level |
| 80436 | | ICD9 |  | Closed fractures involving skull or face with other bones, with other and unspecified intracranial hemorrhage, with loss of consciousness of unspecified duration |
| 80439 | | ICD9 |  | Closed fractures involving skull or face with other bones, with other and unspecified intracranial hemorrhage, with concussion, unspecified |
| 80440 | | ICD9 |  | Closed fractures involving skull or face with other bones, with intracranial injury of other and unspecified nature, unspecified state of consciousness |
| 80441 | | ICD9 |  | Closed fractures involving skull or face with other bones, with intracranial injury of other and unspecified nature, with no loss of consciousness |
| 80442 | | ICD9 |  | Closed fractures involving skull or face with other bones, with intracranial injury of other and unspecified nature, with brief [less than one hour] loss of consciousness |
| 80443 | | ICD9 |  | Closed fractures involving skull or face with other bones, with intracranial injury of other and unspecified nature, with moderate [1-24 hours] loss of consciousness |
| 80444 | | ICD9 |  | Closed fractures involving skull or face with other bones, with intracranial injury of other and unspecified nature, with prolonged [more than 24 hours] loss of consciousness and return to pre-existing conscious level |
| 80445 | | ICD9 |  | Closed fractures involving skull or face with other bones, with intracranial injury of other and unspecified nature, with prolonged [more than 24 hours] loss of consciousness, without return to pre-existing conscious level |
| 80446 | | ICD9 |  | Closed fractures involving skull or face with other bones, with intracranial injury of other and unspecified nature, with loss of consciousness of unspecified duration |
| 80449 | | ICD9 |  | Closed fractures involving skull or face with other bones, with intracranial injury of other and unspecified nature, with concussion, unspecified |
| 80450 | | ICD9 |  | Open fractures involving skull or face with other bones, without mention of intracranial injury, unspecified state of consciousness |
| 80451 | | ICD9 |  | Open fractures involving skull or face with other bones, without mention of intracranial injury, with no loss of consciousness |
| 80452 | | ICD9 |  | Open fractures involving skull or face with other bones, without mention of intracranial injury, with brief [less than one hour] loss of consciousness |
| 80453 | | ICD9 |  | Open fractures involving skull or face with other bones, without mention of intracranial injury, with moderate [1-24 hours] loss of consciousness |
| 80454 | | ICD9 |  | Open fractures involving skull or face with other bones, without mention of intracranial injury, with prolonged [more than 24 hours] loss of consciousness and return to pre-existing conscious level |
| 80455 | | ICD9 |  | Open fractures involving skull or face with other bones, without mention of intracranial injury, with prolonged [more than 24 hours] loss of consciousness, without return to pre-existing conscious level |
| 80456 | | ICD9 |  | Open fractures involving skull or face with other bones, without mention of intracranial injury, with loss of consciousness of unspecified duration |
| 80459 | | ICD9 |  | Open fractures involving skull or face with other bones, without mention of intracranial injury, with concussion, unspecified |
| 80460 | | ICD9 |  | Open fractures involving skull or face with other bones, with cerebral laceration and contusion, unspecified state of consciousness |
| 80461 | | ICD9 |  | Open fractures involving skull or face with other bones, with cerebral laceration and contusion, with no loss of consciousness |
| 80462 | | ICD9 |  | Open fractures involving skull or face with other bones, with cerebral laceration and contusion, with brief [less than one hour] loss of consciousness |
| 80463 | | ICD9 |  | Open fractures involving skull or face with other bones, with cerebral laceration and contusion, with moderate [1-24 hours] loss of consciousness |
| 80464 | | ICD9 |  | Open fractures involving skull or face with other bones, with cerebral laceration and contusion, with prolonged [more than 24 hours] loss of consciousness and return to pre-existing conscious level |
| 80465 | | ICD9 |  | Open fractures involving skull or face with other bones, with cerebral laceration and contusion, with prolonged [more than 24 hours] loss of consciousness, without return to pre-existing conscious level |
| 80466 | | ICD9 |  | Open fractures involving skull or face with other bones, with cerebral laceration and contusion, with loss of consciousness of unspecified duration |
| 80469 | | ICD9 |  | Open fractures involving skull or face with other bones, with cerebral laceration and contusion, with concussion, unspecified |
| 80470 | | ICD9 |  | Open fractures involving skull or face with other bones with subarachnoid, subdural, and extradural hemorrhage, unspecified state of consciousness |
| 80471 | | ICD9 |  | Open fractures involving skull or face with other bones with subarachnoid, subdural, and extradural hemorrhage, with no loss of consciousness |
| 80472 | | ICD9 |  | Open fractures involving skull or face with other bones with subarachnoid, subdural, and extradural hemorrhage, with brief [less than one hour] loss of consciousness |
| 80473 | | ICD9 |  | Open fractures involving skull or face with other bones with subarachnoid, subdural, and extradural hemorrhage, with moderate [1-24 hours] loss of consciousness |
| 85103 | | ICD9 |  | Cortex (cerebral) contusion without mention of open intracranial wound, with moderate [1-24 hours] loss of consciousness |
| 85104 | | ICD9 |  | Cortex (cerebral) contusion without mention of open intracranial wound, with prolonged [more than 24 hours] loss of consciousness and return to pre-existing conscious level |
| 85105 | | ICD9 |  | Cortex (cerebral) contusion without mention of open intracranial wound, with prolonged [more than 24 hours] loss of consciousness without return to pre-existing conscious level |
| 85106 | | ICD9 |  | Cortex (cerebral) contusion without mention of open intracranial wound, with loss of consciousness of unspecified duration |
| 80346 | | ICD9 |  | Other closed skull fracture with intracranial injury of other and unspecified nature, with loss of consciousness of unspecified duration |
| 80349 | | ICD9 |  | Other closed skull fracture with intracranial injury of other and unspecified nature, with concussion, unspecified |
| 80350 | | ICD9 |  | Other open skull fracture without mention of injury, unspecified state of consciousness |
| 80351 | | ICD9 |  | Other open skull fracture without mention of intracranial injury, with no loss of consciousness |
| 80352 | | ICD9 |  | Other open skull fracture without mention of intracranial injury, with brief [less than one hour] loss of consciousness |
| 80353 | | ICD9 |  | Other open skull fracture without mention of intracranial injury, with moderate [1-24 hours] loss of consciousness |
| 80354 | | ICD9 |  | Other open skull fracture without mention of intracranial injury, with prolonged [more than 24 hours] loss of consciousness and return to pre-existing conscious level |
| 80355 | | ICD9 |  | Other open skull fracture without mention of intracranial injury, with prolonged [more than 24 hours] loss of consciousness, without return to pre-existing conscious level |
| 80356 | | ICD9 |  | Other open skull fracture without mention of intracranial injury, with loss of consciousness of unspecified duration |
| 80359 | | ICD9 |  | Other open skull fracture without mention of intracranial injury, with concussion, unspecified |
| 80360 | | ICD9 |  | Other open skull fracture with cerebral laceration and contusion, unspecified state of consciousness |
| 80361 | | ICD9 |  | Other open skull fracture with cerebral laceration and contusion, with no loss of consciousness |
| 80362 | | ICD9 |  | Other open skull fracture with cerebral laceration and contusion, with brief [less than one hour] loss of consciousness |
| 80363 | | ICD9 |  | Other open skull fracture with cerebral laceration and contusion, with moderate [1-24 hours] loss of consciousness |
| 80364 | | ICD9 |  | Other open skull fracture with cerebral laceration and contusion, with prolonged [more than 24 hours] loss of consciousness and return to pre-existing conscious level |
| 80365 | | ICD9 |  | Other open skull fracture with cerebral laceration and contusion, with prolonged [more than 24 hours] loss of consciousness, without return to pre-existing conscious level |
| 80366 | | ICD9 |  | Other open skull fracture with cerebral laceration and contusion, with loss of consciousness of unspecified duration |
| 80369 | | ICD9 |  | Other open skull fracture with cerebral laceration and contusion, with concussion, unspecified |
| 80370 | | ICD9 |  | Other open skull fracture with subarachnoid, subdural, and extradural hemorrhage, unspecified state of consciousness |
| 80371 | | ICD9 |  | Other open skull fracture with subarachnoid, subdural, and extradural hemorrhage, with no loss of consciousness |
| 80372 | | ICD9 |  | Other open skull fracture with subarachnoid, subdural, and extradural hemorrhage, with brief [less than one hour] loss of consciousness |
| 80373 | | ICD9 |  | Other open skull fracture with subarachnoid, subdural, and extradural hemorrhage, with moderate [1-24 hours] loss of consciousness |
| 80374 | | ICD9 |  | Other open skull fracture with subarachnoid, subdural, and extradural hemorrhage, with prolonged [more than 24 hours] loss of consciousness and return to pre-existing conscious level |
| 80375 | | ICD9 |  | Other open skull fracture with subarachnoid, subdural, and extradural hemorrhage, with prolonged [more than 24 hours] loss of consciousness, without return to pre-existing conscious level |
| 80376 | | ICD9 |  | Other open skull fracture with subarachnoid, subdural, and extradural hemorrhage, with loss of consciousness of unspecified duration |
| 80379 | | ICD9 |  | Other open skull fracture with subarachnoid, subdural, and extradural hemorrhage, with concussion, unspecified |
| 80380 | | ICD9 |  | Other open skull fracture with other and unspecified intracranial hemorrhage, unspecified state of consciousness |
| 80381 | | ICD9 |  | Other open skull fracture with other and unspecified intracranial hemorrhage, with no loss of consciousness |
| 80382 | | ICD9 |  | Other open skull fracture with other and unspecified intracranial hemorrhage, with brief [less than one hour] loss of consciousness |
| 80383 | | ICD9 |  | Other open skull fracture with other and unspecified intracranial hemorrhage, with moderate [1-24 hours] loss of consciousness |
| 80384 | | ICD9 |  | Other open skull fracture with other and unspecified intracranial hemorrhage, with prolonged [more than 24 hours] loss of consciousness and return to pre-existing conscious level |
| 80385 | | ICD9 |  | Other open skull fracture with other and unspecified intracranial hemorrhage, with prolonged [more than 24 hours] loss of consciousness, without return to pre-existing conscious level |
| 80386 | | ICD9 |  | Other open skull fracture with other and unspecified intracranial hemorrhage, with loss of consciousness of unspecified duration |
| 80389 | | ICD9 |  | Other open skull fracture with other and unspecified intracranial hemorrhage, with concussion, unspecified |
| 80390 | | ICD9 |  | Other open skull fracture with intracranial injury of other and unspecified nature, unspecified state of consciousness |
| 80391 | | ICD9 |  | Other open skull fracture with intracranial injury of other and unspecified nature, with no loss of consciousness |
| 80392 | | ICD9 |  | Other open skull fracture with intracranial injury of other and unspecified nature, with brief [less than one hour] loss of consciousness |
| 80393 | | ICD9 |  | Other open skull fracture with intracranial injury of other and unspecified nature, with moderate [1-24 hours] loss of consciousness |
| 80394 | | ICD9 |  | Other open skull fracture with intracranial injury of other and unspecified nature, with prolonged [more than 24 hours] loss of consciousness and return to pre-existing conscious level |
| 80395 | | ICD9 |  | Other open skull fracture with intracranial injury of other and unspecified nature, with prolonged [more than 24 hours] loss of consciousness, without return to pre-existing conscious level |
| 80396 | | ICD9 |  | Other open skull fracture with intracranial injury of other and unspecified nature, with loss of consciousness of unspecified duration |
| 80399 | | ICD9 |  | Other open skull fracture with intracranial injury of other and unspecified nature, with concussion, unspecified |
| 80400 | | ICD9 |  | Closed fractures involving skull or face with other bones, without mention of intracranial injury, unspecified state of consciousness |
| 80401 | | ICD9 |  | Closed fractures involving skull or face with other bones, without mention of intracranial injury, with no loss of consciousness |
| 80402 | | ICD9 |  | Closed fractures involving skull or face with other bones, without mention of intracranial injury, with brief [less than one hour] loss of consciousness |
| 80403 | | ICD9 |  | Closed fractures involving skull or face with other bones, without mention of intracranial injury, with moderate [1-24 hours] loss of consciousness |
| 80404 | | ICD9 |  | Closed fractures involving skull or face with other bones, without mention or intracranial injury, with prolonged [more than 24 hours] loss of consciousness and return to pre-existing conscious level |
| 80405 | | ICD9 |  | Closed fractures involving skull of face with other bones, without mention of intracranial injury, with prolonged [more than 24 hours] loss of consciousness, without return to pre-existing conscious level |
| 80406 | | ICD9 |  | Closed fractures involving skull of face with other bones, without mention of intracranial injury, with loss of consciousness of unspecified duration |
| 80409 | | ICD9 |  | Closed fractures involving skull of face with other bones, without mention of intracranial injury, with concussion, unspecified |
| 80410 | | ICD9 |  | Closed fractures involving skull or face with other bones, with cerebral laceration and contusion, unspecified state of consciousness |
| 80474 | | ICD9 |  | Open fractures involving skull or face with other bones with subarachnoid, subdural, and extradural hemorrhage, with prolonged [more than 24 hours] loss of consciousness and return to pre-existing conscious level |
| 80475 | | ICD9 |  | Open fractures involving skull or face with other bones with subarachnoid, subdural, and extradural hemorrhage, with prolonged [more than 24 hours] loss of consciousness, without return to pre-existing conscious level |
| 80476 | | ICD9 |  | Open fractures involving skull or face with other bones with subarachnoid, subdural, and extradural hemorrhage, with loss of consciousness of unspecified duration |
| 80479 | | ICD9 |  | Open fractures involving skull or face with other bones with subarachnoid, subdural, and extradural hemorrhage, with concussion, unspecified |
| 80480 | | ICD9 |  | Open fractures involving skull or face with other bones, with other and unspecified intracranial hemorrhage, unspecified state of consciousness |
| 80481 | | ICD9 |  | Open fractures involving skull or face with other bones, with other and unspecified intracranial hemorrhage, with no loss of consciousness |
| 80482 | | ICD9 |  | Open fractures involving skull or face with other bones, with other and unspecified intracranial hemorrhage, with brief [less than one hour] loss of consciousness |
| 80483 | | ICD9 |  | Open fractures involving skull or face with other bones, with other and unspecified intracranial hemorrhage, with moderate [1-24 hours] loss of consciousness |
| 80484 | | ICD9 |  | Open fractures involving skull or face with other bones, with other and unspecified intracranial hemorrhage, with prolonged [more than 24 hours] loss of consciousness and return to pre-existing conscious level |
| 80485 | | ICD9 |  | Open fractures involving skull or face with other bones, with other and unspecified intracranial hemorrhage, with prolonged [more than 24 hours] loss consciousness, without return to pre-existing conscious level |
| 80486 | | ICD9 |  | Open fractures involving skull or face with other bones, with other and unspecified intracranial hemorrhage, with loss of consciousness of unspecified duration |
| 80489 | | ICD9 |  | Open fractures involving skull or face with other bones, with other and unspecified intracranial hemorrhage, with concussion, unspecified |
| 80490 | | ICD9 |  | Open fractures involving skull or face with other bones, with intracranial injury of other and unspecified nature, unspecified state of consciousness |
| 80491 | | ICD9 |  | Open fractures involving skull or face with other bones, with intracranial injury of other and unspecified nature, with no loss of consciousness |
| 80492 | | ICD9 |  | Open fractures involving skull or face with other bones, with intracranial injury of other and unspecified nature, with brief [less than one hour] loss of consciousness |
| 80493 | | ICD9 |  | Open fractures involving skull or face with other bones, with intracranial injury of other and unspecified nature, with moderate [1-24 hours] loss of consciousness |
| 80494 | | ICD9 |  | Open fractures involving skull or face with other bones, with intracranial injury of other and unspecified nature, with prolonged [more than 24 hours] loss of consciousness and return to pre-existing conscious level |
| 80495 | | ICD9 |  | Open fractures involving skull or face with other bones, with intracranial injury of other and unspecified nature, with prolonged [more than 24 hours] loss of consciousness without return to pre-existing conscious level |
| 80496 | | ICD9 |  | Open fractures involving skull or face with other bones, with intracranial injury of other and unspecified nature, with loss of consciousness of unspecified duration |
| 80499 | | ICD9 |  | Open fractures involving skull or face with other bones, with intracranial injury of other and unspecified nature, with concussion, unspecified |
| 85109 | | ICD9 |  | Cortex (cerebral) contusion without mention of open intracranial wound, with concussion, unspecified |
| 85110 | | ICD9 |  | Cortex (cerebral) contusion with open intracranial wound, unspecified state of consciousness |
| 85111 | | ICD9 |  | Cortex (cerebral) contusion with open intracranial wound, with no loss of consciousness |
| 85112 | | ICD9 |  | Cortex (cerebral) contusion with open intracranial wound, with brief [less than one hour] loss of consciousness |
| 85113 | | ICD9 |  | Cortex (cerebral) contusion with open intracranial wound, with moderate [1-24 hours] loss of consciousness |
| 85114 | | ICD9 |  | Cortex (cerebral) contusion with open intracranial wound, with prolonged [more than 24 hours] loss of consciousness and return to pre-existing conscious level |
| 85115 | | ICD9 |  | Cortex (cerebral) contusion with open intracranial wound, with prolonged [more than 24 hours] loss of consciousness without return to pre-existing conscious level |
| 85116 | | ICD9 |  | Cortex (cerebral) contusion with open intracranial wound, with loss of consciousness of unspecified duration |
| 85119 | | ICD9 |  | Cortex (cerebral) contusion with open intracranial wound, with concussion, unspecified |
| 85120 | | ICD9 |  | Cortex (cerebral) laceration without mention of open intracranial wound, unspecified state of consciousness |
| 85121 | | ICD9 |  | Cortex (cerebral) laceration without mention of open intracranial wound, with no loss of consciousness |
| 85122 | | ICD9 |  | Cortex (cerebral) laceration without mention of open intracranial wound, with brief [less than one hour] loss of consciousness |
| 85123 | | ICD9 |  | Cortex (cerebral) laceration without mention of open intracranial wound, with moderate [1-24 hours] loss of consciousness |
| 85124 | | ICD9 |  | Cortex (cerebral) laceration without mention of open intracranial wound, with prolonged [more than 24 hours] loss of consciousness and return to pre-existing conscious level |
| 85125 | | ICD9 |  | Cortex (cerebral) laceration without mention of open intracranial wound, with prolonged [more than 24 hours] loss of consciousness |
| 85126 | | ICD9 |  | Cortex (cerebral) laceration without mention of open intracranial wound, with loss of consciousness of unspecified duration |
| 85129 | | ICD9 |  | Cortex (cerebral) laceration without mention of open intracranial wound, with concussion, unspecified |
| 85130 | | ICD9 |  | Cortex (cerebral) laceration with open intracranial wound, unspecified state of consciousness |
| 85131 | | ICD9 |  | Cortex (cerebral) laceration with open intracranial wound, with no loss of consciousness |
| 85132 | | ICD9 |  | Cortex (cerebral) laceration with open intracranial wound, with brief [less than one hour] loss of consciousness |
| 85133 | | ICD9 |  | Cortex (cerebral) laceration with open intracranial wound, with moderate [1-24 hours] loss of consciousness |
| 85134 | | ICD9 |  | Cortex (cerebral) laceration with open intracranial wound, with prolonged [more than 24 hours] loss of consciousness and return to pre-existing conscious level |
| 85135 | | ICD9 |  | Cortex (cerebral) laceration with open intracranial wound, with prolonged [more than 24 hours] loss of consciousness without return to pre-existing conscious level |
| 85136 | | ICD9 |  | Cortex (cerebral) laceration with open intracranial wound, with loss of consciousness of unspecified duration |
| 85139 | | ICD9 |  | Cortex (cerebral) laceration with open intracranial wound, with concussion, unspecified |
| 85140 | | ICD9 |  | Cerebellar or brain stem contusion without mention of open intracranial wound, unspecified state of consciousness |
| 85141 | | ICD9 |  | Cerebellar or brain stem contusion without mention of open intracranial wound, with no loss of consciousness |
| 85142 | | ICD9 |  | Cerebellar or brain stem contusion without mention of open intracranial wound, with brief [less than one hour] loss of consciousness |
| 85143 | | ICD9 |  | Cerebellar or brain stem contusion without mention of open intracranial wound, with moderate [1-24 hours] loss of consciousness |
| 85144 | | ICD9 |  | Cerebellar or brain stem contusion without mention of open intracranial wound, with prolonged [more than 24 hours] loss consciousness and return to pre-existing conscious level |
| 85145 | | ICD9 |  | Cerebellar or brain stem contusion without mention of open intracranial wound, with prolonged [more than 24 hours] loss of consciousness without return to pre-existing conscious level |
| 85146 | | ICD9 |  | Cerebellar or brain stem contusion without mention of open intracranial wound, with loss of consciousness of unspecified duration |
| 85149 | | ICD9 |  | Cerebellar or brain stem contusion without mention of open intracranial wound, with concussion, unspecified |
| 85150 | | ICD9 |  | Cerebellar or brain stem contusion with open intracranial wound, unspecified state of consciousness |
| 85151 | | ICD9 |  | Cerebellar or brain stem contusion with open intracranial wound, with no loss of consciousness |
| 85152 | | ICD9 |  | Cerebellar or brain stem contusion with open intracranial wound, with brief [less than one hour] loss of consciousness |
| 85153 | | ICD9 |  | Cerebellar or brain stem contusion with open intracranial wound, with moderate [1-24 hours] loss of consciousness |
| P108 | | ICD10 |  | Other intracranial lacerations and hemorrhages due to birth injury |
| P109 | | ICD10 |  | Unspecified intracranial laceration and hemorrhage due to birth injury |
| P258 | | ICD10 |  | Other intracranial (nontraumatic) hemorrhages of newborn |
| S06810A | | ICD10 |  | Injury of right internal carotid artery, intracranial portion, not elsewhere classified without loss of consciousness, initial encounter |
| S06810D | | ICD10 |  | Injury of right internal carotid artery, intracranial portion, not elsewhere classified without loss of consciousness, subsequent encounter |
| S06810S | | ICD10 |  | Injury of right internal carotid artery, intracranial portion, not elsewhere classified without loss of consciousness, sequela |
| S06811A | | ICD10 |  | Injury of right internal carotid artery, intracranial portion, not elsewhere classified with loss of consciousness of 30 minutes or less, initial encounter |
| S06811D | | ICD10 |  | Injury of right internal carotid artery, intracranial portion, not elsewhere classified with loss of consciousness of 30 minutes or less, subsequent encounter |
| S06811S | | ICD10 |  | Injury of right internal carotid artery, intracranial portion, not elsewhere classified with loss of consciousness of 30 minutes or less, sequela |
| S06812A | | ICD10 |  | Injury of right internal carotid artery, intracranial portion, not elsewhere classified with loss of consciousness of 31 minutes to 59 minutes, initial encounter |
| S06812D | | ICD10 |  | Injury of right internal carotid artery, intracranial portion, not elsewhere classified with loss of consciousness of 31 minutes to 59 minutes, subsequent encounter |
| S06812D | | ICD10 |  | Injury of right internal carotid artery, intracranial portion, not elsewhere classified with loss of consciousness of 31 minutes to 59 minutes, subsequent encounter |
| S06812S | | ICD10 |  | Injury of right internal carotid artery, intracranial portion, not elsewhere classified with loss of consciousness of 31 minutes to 59 minutes, sequela |
| S06813A | | ICD10 |  | Injury of right internal carotid artery, intracranial portion, not elsewhere classified with loss of consciousness of 1 hour to 5 hours 59 minutes, initial encounter |
| S06813D | | ICD10 |  | Injury of right internal carotid artery, intracranial portion, not elsewhere classified with loss of consciousness of 1 hour to 5 hours 59 minutes, subsequent encounter |
| S06813S | | ICD10 |  | Injury of right internal carotid artery, intracranial portion, not elsewhere classified with loss of consciousness of 1 hour to 5 hours 59 minutes, sequela |
| S06814A | | ICD10 |  | Injury of right internal carotid artery, intracranial portion, not elsewhere classified with loss of consciousness of 6 hours to 24 hours, initial encounter |
| S06814D | | ICD10 |  | Injury of right internal carotid artery, intracranial portion, not elsewhere classified with loss of consciousness of 6 hours to 24 hours, subsequent encounter |
| S06814S | | ICD10 |  | Injury of right internal carotid artery, intracranial portion, not elsewhere classified with loss of consciousness of 6 hours to 24 hours, sequela |
| S06815A | | ICD10 |  | Injury of right internal carotid artery, intracranial portion, not elsewhere classified with loss of consciousness greater than 24 hours with return to pre-existing conscious level, initial encounter |
| S06815D | | ICD10 |  | Injury of right internal carotid artery, intracranial portion, not elsewhere classified with loss of consciousness greater than 24 hours with return to pre-existing conscious level, subsequent encounter |
| S06815S | | ICD10 |  | Injury of right internal carotid artery, intracranial portion, not elsewhere classified with loss of consciousness greater than 24 hours with return to pre-existing conscious level, sequela |
| S06816A | | ICD10 |  | Injury of right internal carotid artery, intracranial portion, not elsewhere classified with loss of consciousness greater than 24 hours without return to pre-existing conscious level with patient surviving, initial encounter |
| S079XXA | | ICD10 |  | Crushing injury of head, part unspecified, initial encounter |
| S06816D | | ICD10 |  | Injury of right internal carotid artery, intracranial portion, not elsewhere classified with loss of consciousness greater than 24 hours without return to pre-existing conscious level with patient surviving, subsequent encounter |
| S06816S | | ICD10 |  | Injury of right internal carotid artery, intracranial portion, not elsewhere classified with loss of consciousness greater than 24 hours without return to pre-existing conscious level with patient surviving, sequela |
| S06817A | | ICD10 |  | Injury of right internal carotid artery, intracranial portion, not elsewhere classified with loss of consciousness of any duration with death due to brain injury prior to regaining consciousness, initial encounter |
| S06818A | | ICD10 |  | Injury of right internal carotid artery, intracranial portion, not elsewhere classified with loss of consciousness of any duration with death due to other cause prior to regaining consciousness, initial encounter |
| S06819A | | ICD10 |  | Injury of right internal carotid artery, intracranial portion, not elsewhere classified with loss of consciousness of unspecified duration, initial encounter |
| S06819D | | ICD10 |  | Injury of right internal carotid artery, intracranial portion, not elsewhere classified with loss of consciousness of unspecified duration, subsequent encounter |
| S06819S | | ICD10 |  | Injury of right internal carotid artery, intracranial portion, not elsewhere classified with loss of consciousness of unspecified duration, sequela |
| S06820A | | ICD10 |  | Injury of left internal carotid artery, intracranial portion, not elsewhere classified without loss of consciousness, initial encounter |
| S06820D | | ICD10 |  | Injury of left internal carotid artery, intracranial portion, not elsewhere classified without loss of consciousness, subsequent encounter |
| S06820S | | ICD10 |  | Injury of left internal carotid artery, intracranial portion, not elsewhere classified without loss of consciousness, sequela |
| S06821A | | ICD10 |  | Injury of left internal carotid artery, intracranial portion, not elsewhere classified with loss of consciousness of 30 minutes or less, initial encounter |
| S06821D | | ICD10 |  | Injury of left internal carotid artery, intracranial portion, not elsewhere classified with loss of consciousness of 30 minutes or less, subsequent encounter |
| S06821S | | ICD10 |  | Injury of left internal carotid artery, intracranial portion, not elsewhere classified with loss of consciousness of 30 minutes or less, sequela |
| S06822A | | ICD10 |  | Injury of left internal carotid artery, intracranial portion, not elsewhere classified with loss of consciousness of 31 minutes to 59 minutes, initial encounter |
| S06822D | | ICD10 |  | Injury of left internal carotid artery, intracranial portion, not elsewhere classified with loss of consciousness of 31 minutes to 59 minutes, subsequent encounter |
| S06822S | | ICD10 |  | Injury of left internal carotid artery, intracranial portion, not elsewhere classified with loss of consciousness of 31 minutes to 59 minutes, sequela |
| S06823A | | ICD10 |  | Injury of left internal carotid artery, intracranial portion, not elsewhere classified with loss of consciousness of 1 hour to 5 hours 59 minutes, initial encounter |
| S06823D | | ICD10 |  | Injury of left internal carotid artery, intracranial portion, not elsewhere classified with loss of consciousness of 1 hour to 5 hours 59 minutes, subsequent encounter |
| S06823S | | ICD10 |  | Injury of left internal carotid artery, intracranial portion, not elsewhere classified with loss of consciousness of 1 hour to 5 hours 59 minutes, sequela |
| S06824A | | ICD10 |  | Injury of left internal carotid artery, intracranial portion, not elsewhere classified with loss of consciousness of 6 hours to 24 hours, initial encounter |
| S06824D | | ICD10 |  | Injury of left internal carotid artery, intracranial portion, not elsewhere classified with loss of consciousness of 6 hours to 24 hours, subsequent encounter |
| S06824S | | ICD10 |  | Injury of left internal carotid artery, intracranial portion, not elsewhere classified with loss of consciousness of 6 hours to 24 hours, sequela |
| S06825A | | ICD10 |  | Injury of left internal carotid artery, intracranial portion, not elsewhere classified with loss of consciousness greater than 24 hours with return to pre-existing conscious level, initial encounter |
| S06825D | | ICD10 |  | Injury of left internal carotid artery, intracranial portion, not elsewhere classified with loss of consciousness greater than 24 hours with return to pre-existing conscious level, subsequent encounter |
| S06825S | | ICD10 |  | Injury of left internal carotid artery, intracranial portion, not elsewhere classified with loss of consciousness greater than 24 hours with return to pre-existing conscious level, sequela |
| S06826A | | ICD10 |  | Injury of left internal carotid artery, intracranial portion, not elsewhere classified with loss of consciousness greater than 24 hours without return to pre-existing conscious level with patient surviving, initial encounter |
| S06826D | | ICD10 |  | Injury of left internal carotid artery, intracranial portion, not elsewhere classified with loss of consciousness greater than 24 hours without return to pre-existing conscious level with patient surviving, subsequent encounter |
| S06826S | | ICD10 |  | Injury of left internal carotid artery, intracranial portion, not elsewhere classified with loss of consciousness greater than 24 hours without return to pre-existing conscious level with patient surviving, sequela |
| S06827A | | ICD10 |  | Injury of left internal carotid artery, intracranial portion, not elsewhere classified with loss of consciousness of any duration with death due to brain injury prior to regaining consciousness, initial encounter |
| S06828A | | ICD10 |  | Injury of left internal carotid artery, intracranial portion, not elsewhere classified with loss of consciousness of any duration with death due to other cause prior to regaining consciousness, initial encounter |
| S06829A | | ICD10 |  | Injury of left internal carotid artery, intracranial portion, not elsewhere classified with loss of consciousness of unspecified duration, initial encounter |
| S06829D | | ICD10 |  | Injury of left internal carotid artery, intracranial portion, not elsewhere classified with loss of consciousness of unspecified duration, subsequent encounter |
| S06829S | | ICD10 |  | Injury of left internal carotid artery, intracranial portion, not elsewhere classified with loss of consciousness of unspecified duration, sequela |
| S06890A | | ICD10 |  | Other specified intracranial injury without loss of consciousness, initial encounter |
| S06890D | | ICD10 |  | Other specified intracranial injury without loss of consciousness, subsequent encounter |
| S06890S | | ICD10 |  | Other specified intracranial injury without loss of consciousness, sequela |
| S06891A | | ICD10 |  | Other specified intracranial injury with loss of consciousness of 30 minutes or less, initial encounter |
| S06891D | | ICD10 |  | Other specified intracranial injury with loss of consciousness of 30 minutes or less, subsequent encounter |
| S06891S | | ICD10 |  | Other specified intracranial injury with loss of consciousness of 30 minutes or less, sequela |
| W880XXS | | ICD10 |  | Exposure to X-rays, sequela |
| S06892A | | ICD10 |  | Other specified intracranial injury with loss of consciousness of 31 minutes to 59 minutes, initial encounter |
| S06892D | | ICD10 |  | Other specified intracranial injury with loss of consciousness of 31 minutes to 59 minutes, subsequent encounter |
| S06892S | | ICD10 |  | Other specified intracranial injury with loss of consciousness of 31 minutes to 59 minutes, sequela |
| S06893A | | ICD10 |  | Other specified intracranial injury with loss of consciousness of 1 hour to 5 hours 59 minutes, initial encounter |
| S06893D | | ICD10 |  | Other specified intracranial injury with loss of consciousness of 1 hour to 5 hours 59 minutes, subsequent encounter |
| S06893S | | ICD10 |  | Other specified intracranial injury with loss of consciousness of 1 hour to 5 hours 59 minutes, sequela |
| S06894A | | ICD10 |  | Other specified intracranial injury with loss of consciousness of 6 hours to 24 hours, initial encounter |
| S06894D | | ICD10 |  | Other specified intracranial injury with loss of consciousness of 6 hours to 24 hours, subsequent encounter |
| S06894S | | ICD10 |  | Other specified intracranial injury with loss of consciousness of 6 hours to 24 hours, sequela |
| S06895A | | ICD10 |  | Other specified intracranial injury with loss of consciousness greater than 24 hours with return to pre-existing conscious level, initial encounter |
| S06895D | | ICD10 |  | Other specified intracranial injury with loss of consciousness greater than 24 hours with return to pre-existing conscious level, subsequent encounter |
| S06895S | | ICD10 |  | Other specified intracranial injury with loss of consciousness greater than 24 hours with return to pre-existing conscious level, sequela |
| S06896A | | ICD10 |  | Other specified intracranial injury with loss of consciousness greater than 24 hours without return to pre-existing conscious level with patient surviving, initial encounter |
| S06896D | | ICD10 |  | Other specified intracranial injury with loss of consciousness greater than 24 hours without return to pre-existing conscious level with patient surviving, subsequent encounter |
| S06896S | | ICD10 |  | Other specified intracranial injury with loss of consciousness greater than 24 hours without return to pre-existing conscious level with patient surviving, sequela |
| S06897A | | ICD10 |  | Other specified intracranial injury with loss of consciousness of any duration with death due to brain injury prior to regaining consciousness, initial encounter |
| S06898A | | ICD10 |  | Other specified intracranial injury with loss of consciousness of any duration with death due to other cause prior to regaining consciousness, initial encounter |
| S06899A | | ICD10 |  | Other specified intracranial injury with loss of consciousness of unspecified duration, initial encounter |
| S06899D | | ICD10 |  | Other specified intracranial injury with loss of consciousness of unspecified duration, subsequent encounter |
| S06899S | | ICD10 |  | Other specified intracranial injury with loss of consciousness of unspecified duration, sequela |
| S069X0A | | ICD10 |  | Unspecified intracranial injury without loss of consciousness, initial encounter |
| S069X0D | | ICD10 |  | Unspecified intracranial injury without loss of consciousness, subsequent encounter |
| S069X0S | | ICD10 |  | Unspecified intracranial injury without loss of consciousness, sequela |
| S069X1A | | ICD10 |  | Unspecified intracranial injury with loss of consciousness of 30 minutes or less, initial encounter |
| S069X1D | | ICD10 |  | Unspecified intracranial injury with loss of consciousness of 30 minutes or less, subsequent encounter |
| S069X1S | | ICD10 |  | Unspecified intracranial injury with loss of consciousness of 30 minutes or less, sequela |
| S069X2A | | ICD10 |  | Unspecified intracranial injury with loss of consciousness of 31 minutes to 59 minutes, initial encounter |
| S069X2D | | ICD10 |  | Unspecified intracranial injury with loss of consciousness of 31 minutes to 59 minutes, subsequent encounter |
| S069X2S | | ICD10 |  | Unspecified intracranial injury with loss of consciousness of 31 minutes to 59 minutes, sequela |
| S069X3A | | ICD10 |  | Unspecified intracranial injury with loss of consciousness of 1 hour to 5 hours 59 minutes, initial encounter |
| S069X3D | | ICD10 |  | Unspecified intracranial injury with loss of consciousness of 1 hour to 5 hours 59 minutes, subsequent encounter |
| S069X3S | | ICD10 |  | Unspecified intracranial injury with loss of consciousness of 1 hour to 5 hours 59 minutes, sequela |
| S069X4A | | ICD10 |  | Unspecified intracranial injury with loss of consciousness of 6 hours to 24 hours, initial encounter |
| S069X4D | | ICD10 |  | Unspecified intracranial injury with loss of consciousness of 6 hours to 24 hours, subsequent encounter |
| S069X4S | | ICD10 |  | Unspecified intracranial injury with loss of consciousness of 6 hours to 24 hours, sequela |
| S069X5A | | ICD10 |  | Unspecified intracranial injury with loss of consciousness greater than 24 hours with return to pre-existing conscious level, initial encounter |
| S069X5D | | ICD10 |  | Unspecified intracranial injury with loss of consciousness greater than 24 hours with return to pre-existing conscious level, subsequent encounter |
| S069X5S | | ICD10 |  | Unspecified intracranial injury with loss of consciousness greater than 24 hours with return to pre-existing conscious level, sequela |
| S069X6A | | ICD10 |  | Unspecified intracranial injury with loss of consciousness greater than 24 hours without return to pre-existing conscious level with patient surviving, initial encounter |
| S069X6D | | ICD10 |  | Unspecified intracranial injury with loss of consciousness greater than 24 hours without return to pre-existing conscious level with patient surviving, subsequent encounter |
| S069X6S | | ICD10 |  | Unspecified intracranial injury with loss of consciousness greater than 24 hours without return to pre-existing conscious level with patient surviving, sequela |
| S069X7A | | ICD10 |  | Unspecified intracranial injury with loss of consciousness of any duration with death due to brain injury prior to regaining consciousness, initial encounter |
| S069X8A | | ICD10 |  | Unspecified intracranial injury with loss of consciousness of any duration with death due to other cause prior to regaining consciousness, initial encounter |
| S069X9A | | ICD10 |  | Unspecified intracranial injury with loss of consciousness of unspecified duration, initial encounter |
| S069X9D | | ICD10 |  | Unspecified intracranial injury with loss of consciousness of unspecified duration, subsequent encounter |
| S069X9S | | ICD10 |  | Unspecified intracranial injury with loss of consciousness of unspecified duration, sequela |
| S070XXA | | ICD10 |  | Crushing injury of face, initial encounter |
| S070XXD | | ICD10 |  | Crushing injury of face, subsequent encounter |
| S070XXS | | ICD10 |  | Crushing injury of face, sequela |
| S071XXA | | ICD10 |  | Crushing injury of skull, initial encounter |
| S071XXD | | ICD10 |  | Crushing injury of skull, subsequent encounter |
| S071XXS | | ICD10 |  | Crushing injury of skull, sequela |
| S078XXA | | ICD10 |  | Crushing injury of other parts of head, initial encounter |
| S079XXD | | ICD10 |  | Crushing injury of head, part unspecified, subsequent encounter |
| S079XXS | | ICD10 |  | Crushing injury of head, part unspecified, sequela |
| S080XXA | | ICD10 |  | Avulsion of scalp, initial encounter |
| S080XXD | | ICD10 |  | Avulsion of scalp, subsequent encounter |
| S080XXS | | ICD10 |  | Avulsion of scalp, sequela |
| S08111A | | ICD10 |  | Complete traumatic amputation of right ear, initial encounter |
| S08111D | | ICD10 |  | Complete traumatic amputation of right ear, subsequent encounter |
| S08111S | | ICD10 |  | Complete traumatic amputation of right ear, sequela |
| S08112A | | ICD10 |  | Complete traumatic amputation of left ear, initial encounter |
| S08112D | | ICD10 |  | Complete traumatic amputation of left ear, subsequent encounter |
| S08112S | | ICD10 |  | Complete traumatic amputation of left ear, sequela |
| S08119A | | ICD10 |  | Complete traumatic amputation of unspecified ear, initial encounter |
| S08119D | | ICD10 |  | Complete traumatic amputation of unspecified ear, subsequent encounter |
| S08119S | | ICD10 |  | Complete traumatic amputation of unspecified ear, sequela |
| S08121A | | ICD10 |  | Partial traumatic amputation of right ear, initial encounter |
| S08121D | | ICD10 |  | Partial traumatic amputation of right ear, subsequent encounter |
| S08121S | | ICD10 |  | Partial traumatic amputation of right ear, sequela |
| S08122A | | ICD10 |  | Partial traumatic amputation of left ear, initial encounter |
| S08122D | | ICD10 |  | Partial traumatic amputation of left ear, subsequent encounter |
| S08122S | | ICD10 |  | Partial traumatic amputation of left ear, sequela |
| S08129A | | ICD10 |  | Partial traumatic amputation of unspecified ear, initial encounter |
| S08129D | | ICD10 |  | Partial traumatic amputation of unspecified ear, subsequent encounter |
| S08129S | | ICD10 |  | Partial traumatic amputation of unspecified ear, sequela |
| S08811A | | ICD10 |  | Complete traumatic amputation of nose, initial encounter |
| S08811D | | ICD10 |  | Complete traumatic amputation of nose, subsequent encounter |
| S08811S | | ICD10 |  | Complete traumatic amputation of nose, sequela |
| S08812A | | ICD10 |  | Partial traumatic amputation of nose, initial encounter |
| S08812D | | ICD10 |  | Partial traumatic amputation of nose, subsequent encounter |
| S08812S | | ICD10 |  | Partial traumatic amputation of nose, sequela |
| S0889XA | | ICD10 |  | Traumatic amputation of other parts of head, initial encounter |
| S0889XD | | ICD10 |  | Traumatic amputation of other parts of head, subsequent encounter |
| S0889XS | | ICD10 |  | Traumatic amputation of other parts of head, sequela |
| S090XXA | | ICD10 |  | Injury of blood vessels of head, not elsewhere classified, initial encounter |
| S090XXD | | ICD10 |  | Injury of blood vessels of head, not elsewhere classified, subsequent encounter |
| S090XXS | | ICD10 |  | Injury of blood vessels of head, not elsewhere classified, sequela |
| S0280XA | | ICD10 |  | Fracture of other specified skull and facial bones, unspecified side, initial encounter for closed fracture |
| S0280XB | | ICD10 |  | Fracture of other specified skull and facial bones, unspecified side, initial encounter for open fracture |
| S0280XD | | ICD10 |  | Fracture of other specified skull and facial bones, unspecified side, subsequent encounter for fracture with routine healing |
| S0280XG | | ICD10 |  | Fracture of other specified skull and facial bones, unspecified side, subsequent encounter for fracture with delayed healing |
| S0280XK | | ICD10 |  | Fracture of other specified skull and facial bones, unspecified side, subsequent encounter for fracture with nonunion |
| S0280XS | | ICD10 |  | Fracture of other specified skull and facial bones, unspecified side, sequela |
| S0281XA | | ICD10 |  | Fracture of other specified skull and facial bones, right side, initial encounter for closed fracture |
| S0281XB | | ICD10 |  | Fracture of other specified skull and facial bones, right side, initial encounter for open fracture |
| S0291XA | | ICD10 |  | Unspecified fracture of skull, initial encounter for closed fracture |
| S0291XB | | ICD10 |  | Unspecified fracture of skull, initial encounter for open fracture |
| S0291XD | | ICD10 |  | Unspecified fracture of skull, subsequent encounter for fracture with routine healing |
| S0291XG | | ICD10 |  | Unspecified fracture of skull, subsequent encounter for fracture with delayed healing |
| S0291XK | | ICD10 |  | Unspecified fracture of skull, subsequent encounter for fracture with nonunion |
| S0291XS | | ICD10 |  | Unspecified fracture of skull, sequela |
| S060X1S | | ICD10 |  | Concussion with loss of consciousness of 30 minutes or less, sequela |
| S060X9A | | ICD10 |  | Concussion with loss of consciousness of unspecified duration, initial encounter |
| S060X9D | | ICD10 |  | Concussion with loss of consciousness of unspecified duration, subsequent encounter |
| S060X9S | | ICD10 |  | Concussion with loss of consciousness of unspecified duration, sequela |
| S061X0A | | ICD10 |  | Traumatic cerebral edema without loss of consciousness, initial encounter |
| S061X0D | | ICD10 |  | Traumatic cerebral edema without loss of consciousness, subsequent encounter |
| S061X0S | | ICD10 |  | Traumatic cerebral edema without loss of consciousness, sequela |
| S061X1A | | ICD10 |  | Traumatic cerebral edema with loss of consciousness of 30 minutes or less, initial encounter |
| S061X1D | | ICD10 |  | Traumatic cerebral edema with loss of consciousness of 30 minutes or less, subsequent encounter |
| S061X1S | | ICD10 |  | Traumatic cerebral edema with loss of consciousness of 30 minutes or less, sequela |
| S061X2A | | ICD10 |  | Traumatic cerebral edema with loss of consciousness of 31 minutes to 59 minutes, initial encounter |
| S061X2D | | ICD10 |  | Traumatic cerebral edema with loss of consciousness of 31 minutes to 59 minutes, subsequent encounter |
| S061X2S | | ICD10 |  | Traumatic cerebral edema with loss of consciousness of 31 minutes to 59 minutes, sequela |
| S061X3A | | ICD10 |  | Traumatic cerebral edema with loss of consciousness of 1 hour to 5 hours 59 minutes, initial encounter |
| S061X3D | | ICD10 |  | Traumatic cerebral edema with loss of consciousness of 1 hour to 5 hours 59 minutes, subsequent encounter |
| S061X3S | | ICD10 |  | Traumatic cerebral edema with loss of consciousness of 1 hour to 5 hours 59 minutes, sequela |
| S061X4A | | ICD10 |  | Traumatic cerebral edema with loss of consciousness of 6 hours to 24 hours, initial encounter |
| S061X4D | | ICD10 |  | Traumatic cerebral edema with loss of consciousness of 6 hours to 24 hours, subsequent encounter |
| S061X4S | | ICD10 |  | Traumatic cerebral edema with loss of consciousness of 6 hours to 24 hours, sequela |
| S061X5A | | ICD10 |  | Traumatic cerebral edema with loss of consciousness greater than 24 hours with return to pre-existing conscious level, initial encounter |
| S061X5D | | ICD10 |  | Traumatic cerebral edema with loss of consciousness greater than 24 hours with return to pre-existing conscious level, subsequent encounter |
| S061X5S | | ICD10 |  | Traumatic cerebral edema with loss of consciousness greater than 24 hours with return to pre-existing conscious level, sequela |
| S061X6A | | ICD10 |  | Traumatic cerebral edema with loss of consciousness greater than 24 hours without return to pre-existing conscious level with patient surviving, initial encounter |
| S061X6D | | ICD10 |  | Traumatic cerebral edema with loss of consciousness greater than 24 hours without return to pre-existing conscious level with patient surviving, subsequent encounter |
| S061X6S | | ICD10 |  | Traumatic cerebral edema with loss of consciousness greater than 24 hours without return to pre-existing conscious level with patient surviving, sequela |
| S061X7A | | ICD10 |  | Traumatic cerebral edema with loss of consciousness of any duration with death due to brain injury prior to regaining consciousness, initial encounter |
| S061X8A | | ICD10 |  | Traumatic cerebral edema with loss of consciousness of any duration with death due to other cause prior to regaining consciousness, initial encounter |
| S061X9A | | ICD10 |  | Traumatic cerebral edema with loss of consciousness of unspecified duration, initial encounter |
| S061X9D | | ICD10 |  | Traumatic cerebral edema with loss of consciousness of unspecified duration, subsequent encounter |
| S061X9S | | ICD10 |  | Traumatic cerebral edema with loss of consciousness of unspecified duration, sequela |
| S062X0A | | ICD10 |  | Diffuse traumatic brain injury without loss of consciousness, initial encounter |
| S062X0D | | ICD10 |  | Diffuse traumatic brain injury without loss of consciousness, subsequent encounter |
| S062X0S | | ICD10 |  | Diffuse traumatic brain injury without loss of consciousness, sequela |
| S062X1A | | ICD10 |  | Diffuse traumatic brain injury with loss of consciousness of 30 minutes or less, initial encounter |
| S062X1D | | ICD10 |  | Diffuse traumatic brain injury with loss of consciousness of 30 minutes or less, subsequent encounter |
| S062X1S | | ICD10 |  | Diffuse traumatic brain injury with loss of consciousness of 30 minutes or less, sequela |
| S062X2A | | ICD10 |  | Diffuse traumatic brain injury with loss of consciousness of 31 minutes to 59 minutes, initial encounter |
| S062X2D | | ICD10 |  | Diffuse traumatic brain injury with loss of consciousness of 31 minutes to 59 minutes, subsequent encounter |
| S062X2S | | ICD10 |  | Diffuse traumatic brain injury with loss of consciousness of 31 minutes to 59 minutes, sequela |
| S062X3A | | ICD10 |  | Diffuse traumatic brain injury with loss of consciousness of 1 hour to 5 hours 59 minutes, initial encounter |
| S062X3D | | ICD10 |  | Diffuse traumatic brain injury with loss of consciousness of 1 hour to 5 hours 59 minutes, subsequent encounter |
| S062X3S | | ICD10 |  | Diffuse traumatic brain injury with loss of consciousness of 1 hour to 5 hours 59 minutes, sequela |
| S062X4A | | ICD10 |  | Diffuse traumatic brain injury with loss of consciousness of 6 hours to 24 hours, initial encounter |
| S062X4D | | ICD10 |  | Diffuse traumatic brain injury with loss of consciousness of 6 hours to 24 hours, subsequent encounter |
| S062X4S | | ICD10 |  | Diffuse traumatic brain injury with loss of consciousness of 6 hours to 24 hours, sequela |
| S062X5A | | ICD10 |  | Diffuse traumatic brain injury with loss of consciousness greater than 24 hours with return to pre-existing conscious levels, initial encounter |
| S062X5D | | ICD10 |  | Diffuse traumatic brain injury with loss of consciousness greater than 24 hours with return to pre-existing conscious levels, subsequent encounter |
| S062X5S | | ICD10 |  | Diffuse traumatic brain injury with loss of consciousness greater than 24 hours with return to pre-existing conscious levels, sequela |
| S062X6A | | ICD10 |  | Diffuse traumatic brain injury with loss of consciousness greater than 24 hours without return to pre-existing conscious level with patient surviving, initial encounter |
| S062X6D | | ICD10 |  | Diffuse traumatic brain injury with loss of consciousness greater than 24 hours without return to pre-existing conscious level with patient surviving, subsequent encounter |
| S062X6S | | ICD10 |  | Diffuse traumatic brain injury with loss of consciousness greater than 24 hours without return to pre-existing conscious level with patient surviving, sequela |
| S062X7A | | ICD10 |  | Diffuse traumatic brain injury with loss of consciousness of any duration with death due to brain injury prior to regaining consciousness, initial encounter |
| S062X8A | | ICD10 |  | Diffuse traumatic brain injury with loss of consciousness of any duration with death due to other cause prior to regaining consciousness, initial encounter |
| V1542 | | ICD10 |  | History of emotional abuse |
| S062X9A | | ICD10 |  | Diffuse traumatic brain injury with loss of consciousness of unspecified duration, initial encounter |
| S062X9D | | ICD10 |  | Diffuse traumatic brain injury with loss of consciousness of unspecified duration, subsequent encounter |
| S062X9S | | ICD10 |  | Diffuse traumatic brain injury with loss of consciousness of unspecified duration, sequela |
| S06300A | | ICD10 |  | Unspecified focal traumatic brain injury without loss of consciousness, initial encounter |
| S06300D | | ICD10 |  | Unspecified focal traumatic brain injury without loss of consciousness, subsequent encounter |
| S06300S | | ICD10 |  | Unspecified focal traumatic brain injury without loss of consciousness, sequela |
| S06301A | | ICD10 |  | Unspecified focal traumatic brain injury with loss of consciousness of 30 minutes or less, initial encounter |
| S06301D | | ICD10 |  | Unspecified focal traumatic brain injury with loss of consciousness of 30 minutes or less, subsequent encounter |
| S06301S | | ICD10 |  | Unspecified focal traumatic brain injury with loss of consciousness of 30 minutes or less, sequela |
| S06302A | | ICD10 |  | Unspecified focal traumatic brain injury with loss of consciousness of 31 minutes to 59 minutes, initial encounter |
| S06302D | | ICD10 |  | Unspecified focal traumatic brain injury with loss of consciousness of 31 minutes to 59 minutes, subsequent encounter |
| S06302S | | ICD10 |  | Unspecified focal traumatic brain injury with loss of consciousness of 31 minutes to 59 minutes, sequela |
| S06303A | | ICD10 |  | Unspecified focal traumatic brain injury with loss of consciousness of 1 hour to 5 hours 59 minutes, initial encounter |
| S06303D | | ICD10 |  | Unspecified focal traumatic brain injury with loss of consciousness of 1 hour to 5 hours 59 minutes, subsequent encounter |
| S06303S | | ICD10 |  | Unspecified focal traumatic brain injury with loss of consciousness of 1 hour to 5 hours 59 minutes, sequela |
| S06304A | | ICD10 |  | Unspecified focal traumatic brain injury with loss of consciousness of 6 hours to 24 hours, initial encounter |
| S06304D | | ICD10 |  | Unspecified focal traumatic brain injury with loss of consciousness of 6 hours to 24 hours, subsequent encounter |
| S06304S | | ICD10 |  | Unspecified focal traumatic brain injury with loss of consciousness of 6 hours to 24 hours, sequela |
| S06305A | | ICD10 |  | Unspecified focal traumatic brain injury with loss of consciousness greater than 24 hours with return to pre-existing conscious level, initial encounter |
| S06305D | | ICD10 |  | Unspecified focal traumatic brain injury with loss of consciousness greater than 24 hours with return to pre-existing conscious level, subsequent encounter |
| S06305S | | ICD10 |  | Unspecified focal traumatic brain injury with loss of consciousness greater than 24 hours with return to pre-existing conscious level, sequela |
| S06306A | | ICD10 |  | Unspecified focal traumatic brain injury with loss of consciousness greater than 24 hours without return to pre-existing conscious level with patient surviving, initial encounter |
| S06306D | | ICD10 |  | Unspecified focal traumatic brain injury with loss of consciousness greater than 24 hours without return to pre-existing conscious level with patient surviving, subsequent encounter |
| S06306S | | ICD10 |  | Unspecified focal traumatic brain injury with loss of consciousness greater than 24 hours without return to pre-existing conscious level with patient surviving, sequela |
| S06307A | | ICD10 |  | Unspecified focal traumatic brain injury with loss of consciousness of any duration with death due to brain injury prior to regaining consciousness, initial encounter |
| S06308A | | ICD10 |  | Unspecified focal traumatic brain injury with loss of consciousness of any duration with death due to other cause prior to regaining consciousness, initial encounter |
| S06309A | | ICD10 |  | Unspecified focal traumatic brain injury with loss of consciousness of unspecified duration, initial encounter |
| S06309D | | ICD10 |  | Unspecified focal traumatic brain injury with loss of consciousness of unspecified duration, subsequent encounter |
| S06309S | | ICD10 |  | Unspecified focal traumatic brain injury with loss of consciousness of unspecified duration, sequela |
| S06310A | | ICD10 |  | Contusion and laceration of right cerebrum without loss of consciousness, initial encounter |
| S06310D | | ICD10 |  | Contusion and laceration of right cerebrum without loss of consciousness, subsequent encounter |
| S06310S | | ICD10 |  | Contusion and laceration of right cerebrum without loss of consciousness, sequela |
| S06311A | | ICD10 |  | Contusion and laceration of right cerebrum with loss of consciousness of 30 minutes or less, initial encounter |
| S06311D | | ICD10 |  | Contusion and laceration of right cerebrum with loss of consciousness of 30 minutes or less, subsequent encounter |
| S06311S | | ICD10 |  | Contusion and laceration of right cerebrum with loss of consciousness of 30 minutes or less, sequela |
| S06312A | | ICD10 |  | Contusion and laceration of right cerebrum with loss of consciousness of 31 minutes to 59 minutes, initial encounter |
| S06312D | | ICD10 |  | Contusion and laceration of right cerebrum with loss of consciousness of 31 minutes to 59 minutes, subsequent encounter |
| S06312S | | ICD10 |  | Contusion and laceration of right cerebrum with loss of consciousness of 31 minutes to 59 minutes, sequela |
| S06313A | | ICD10 |  | Contusion and laceration of right cerebrum with loss of consciousness of 1 hour to 5 hours 59 minutes, initial encounter |
| S06313D | | ICD10 |  | Contusion and laceration of right cerebrum with loss of consciousness of 1 hour to 5 hours 59 minutes, subsequent encounter |
| S06313S | | ICD10 |  | Contusion and laceration of right cerebrum with loss of consciousness of 1 hour to 5 hours 59 minutes, sequela |
| S06314A | | ICD10 |  | Contusion and laceration of right cerebrum with loss of consciousness of 6 hours to 24 hours, initial encounter |
| S06314D | | ICD10 |  | Contusion and laceration of right cerebrum with loss of consciousness of 6 hours to 24 hours, subsequent encounter |
| S06314S | | ICD10 |  | Contusion and laceration of right cerebrum with loss of consciousness of 6 hours to 24 hours, sequela |
| S06315A | | ICD10 |  | Contusion and laceration of right cerebrum with loss of consciousness greater than 24 hours with return to pre-existing conscious level, initial encounter |
| S06315D | | ICD10 |  | Contusion and laceration of right cerebrum with loss of consciousness greater than 24 hours with return to pre-existing conscious level, subsequent encounter |
| S06315S | | ICD10 |  | Contusion and laceration of right cerebrum with loss of consciousness greater than 24 hours with return to pre-existing conscious level, sequela |
| S06316A | | ICD10 |  | Contusion and laceration of right cerebrum with loss of consciousness greater than 24 hours without return to pre-existing conscious level with patient surviving, initial encounter |
| S06316D | | ICD10 |  | Contusion and laceration of right cerebrum with loss of consciousness greater than 24 hours without return to pre-existing conscious level with patient surviving, subsequent encounter |
| S078XXD | | ICD10 |  | Crushing injury of other parts of head, subsequent encounter |
| S06316S | | ICD10 |  | Contusion and laceration of right cerebrum with loss of consciousness greater than 24 hours without return to pre-existing conscious level with patient surviving, sequela |
| S06317A | | ICD10 |  | Contusion and laceration of right cerebrum with loss of consciousness of any duration with death due to brain injury prior to regaining consciousness, initial encounter |
| S06318A | | ICD10 |  | Contusion and laceration of right cerebrum with loss of consciousness of any duration with death due to other cause prior to regaining consciousness, initial encounter |
| S06319A | | ICD10 |  | Contusion and laceration of right cerebrum with loss of consciousness of unspecified duration, initial encounter |
| S06319D | | ICD10 |  | Contusion and laceration of right cerebrum with loss of consciousness of unspecified duration, subsequent encounter |
| S06319S | | ICD10 |  | Contusion and laceration of right cerebrum with loss of consciousness of unspecified duration, sequela |
| S06320A | | ICD10 |  | Contusion and laceration of left cerebrum without loss of consciousness, initial encounter |
| S06320D | | ICD10 |  | Contusion and laceration of left cerebrum without loss of consciousness, subsequent encounter |
| S06320S | | ICD10 |  | Contusion and laceration of left cerebrum without loss of consciousness, sequela |
| S06321A | | ICD10 |  | Contusion and laceration of left cerebrum with loss of consciousness of 30 minutes or less, initial encounter |
| S06321D | | ICD10 |  | Contusion and laceration of left cerebrum with loss of consciousness of 30 minutes or less, subsequent encounter |
| S06321S | | ICD10 |  | Contusion and laceration of left cerebrum with loss of consciousness of 30 minutes or less, sequela |
| S06322A | | ICD10 |  | Contusion and laceration of left cerebrum with loss of consciousness of 31 minutes to 59 minutes, initial encounter |
| S06322D | | ICD10 |  | Contusion and laceration of left cerebrum with loss of consciousness of 31 minutes to 59 minutes, subsequent encounter |
| S06322S | | ICD10 |  | Contusion and laceration of left cerebrum with loss of consciousness of 31 minutes to 59 minutes, sequela |
| S06323A | | ICD10 |  | Contusion and laceration of left cerebrum with loss of consciousness of 1 hour to 5 hours 59 minutes, initial encounter |
| S06323D | | ICD10 |  | Contusion and laceration of left cerebrum with loss of consciousness of 1 hour to 5 hours 59 minutes, subsequent encounter |
| S06323S | | ICD10 |  | Contusion and laceration of left cerebrum with loss of consciousness of 1 hour to 5 hours 59 minutes, sequela |
| S06324A | | ICD10 |  | Contusion and laceration of left cerebrum with loss of consciousness of 6 hours to 24 hours, initial encounter |
| S06324D | | ICD10 |  | Contusion and laceration of left cerebrum with loss of consciousness of 6 hours to 24 hours, subsequent encounter |
| S06324S | | ICD10 |  | Contusion and laceration of left cerebrum with loss of consciousness of 6 hours to 24 hours, sequela |
| S06325A | | ICD10 |  | Contusion and laceration of left cerebrum with loss of consciousness greater than 24 hours with return to pre-existing conscious level, initial encounter |
| S06325D | | ICD10 |  | Contusion and laceration of left cerebrum with loss of consciousness greater than 24 hours with return to pre-existing conscious level, subsequent encounter |
| S06325S | | ICD10 |  | Contusion and laceration of left cerebrum with loss of consciousness greater than 24 hours with return to pre-existing conscious level, sequela |
| S06326A | | ICD10 |  | Contusion and laceration of left cerebrum with loss of consciousness greater than 24 hours without return to pre-existing conscious level with patient surviving, initial encounter |
| S06326D | | ICD10 |  | Contusion and laceration of left cerebrum with loss of consciousness greater than 24 hours without return to pre-existing conscious level with patient surviving, subsequent encounter |
| S06326S | | ICD10 |  | Contusion and laceration of left cerebrum with loss of consciousness greater than 24 hours without return to pre-existing conscious level with patient surviving, sequela |
| S06327A | | ICD10 |  | Contusion and laceration of left cerebrum with loss of consciousness of any duration with death due to brain injury prior to regaining consciousness, initial encounter |
| S06328A | | ICD10 |  | Contusion and laceration of left cerebrum with loss of consciousness of any duration with death due to other cause prior to regaining consciousness, initial encounter |
| S06329A | | ICD10 |  | Contusion and laceration of left cerebrum with loss of consciousness of unspecified duration, initial encounter |
| S06329D | | ICD10 |  | Contusion and laceration of left cerebrum with loss of consciousness of unspecified duration, subsequent encounter |
| S06329S | | ICD10 |  | Contusion and laceration of left cerebrum with loss of consciousness of unspecified duration, sequela |
| S06330A | | ICD10 |  | Contusion and laceration of cerebrum, unspecified, without loss of consciousness, initial encounter |
| S06330D | | ICD10 |  | Contusion and laceration of cerebrum, unspecified, without loss of consciousness, subsequent encounter |
| S06330S | | ICD10 |  | Contusion and laceration of cerebrum, unspecified, without loss of consciousness, sequela |
| S06331A | | ICD10 |  | Contusion and laceration of cerebrum, unspecified, with loss of consciousness of 30 minutes or less, initial encounter |
| S06331D | | ICD10 |  | Contusion and laceration of cerebrum, unspecified, with loss of consciousness of 30 minutes or less, subsequent encounter |
| S06331S | | ICD10 |  | Contusion and laceration of cerebrum, unspecified, with loss of consciousness of 30 minutes or less, sequela |
| S06332A | | ICD10 |  | Contusion and laceration of cerebrum, unspecified, with loss of consciousness of 31 minutes to 59 minutes, initial encounter |
| S06332D | | ICD10 |  | Contusion and laceration of cerebrum, unspecified, with loss of consciousness of 31 minutes to 59 minutes, subsequent encounter |
| S06332S | | ICD10 |  | Contusion and laceration of cerebrum, unspecified, with loss of consciousness of 31 minutes to 59 minutes, sequela |
| S06333A | | ICD10 |  | Contusion and laceration of cerebrum, unspecified, with loss of consciousness of 1 hour to 5 hours 59 minutes, initial encounter |
| S06333D | | ICD10 |  | Contusion and laceration of cerebrum, unspecified, with loss of consciousness of 1 hour to 5 hours 59 minutes, subsequent encounter |
| S06334A | | ICD10 |  | Contusion and laceration of cerebrum, unspecified, with loss of consciousness of 6 hours to 24 hours, initial encounter |
| S06334D | | ICD10 |  | Contusion and laceration of cerebrum, unspecified, with loss of consciousness of 6 hours to 24 hours, subsequent encounter |
| S06334S | | ICD10 |  | Contusion and laceration of cerebrum, unspecified, with loss of consciousness of 6 hours to 24 hours, sequela |
| S06335A | | ICD10 |  | Contusion and laceration of cerebrum, unspecified, with loss of consciousness greater than 24 hours with return to pre-existing conscious level, initial encounter |
| S06335D | | ICD10 |  | Contusion and laceration of cerebrum, unspecified, with loss of consciousness greater than 24 hours with return to pre-existing conscious level, subsequent encounter |
| S06335S | | ICD10 |  | Contusion and laceration of cerebrum, unspecified, with loss of consciousness greater than 24 hours with return to pre-existing conscious level, sequela |
| S06336A | | ICD10 |  | Contusion and laceration of cerebrum, unspecified, with loss of consciousness greater than 24 hours without return to pre-existing conscious level with patient surviving, initial encounter |
| S06336D | | ICD10 |  | Contusion and laceration of cerebrum, unspecified, with loss of consciousness greater than 24 hours without return to pre-existing conscious level with patient surviving, subsequent encounter |
| S06336S | | ICD10 |  | Contusion and laceration of cerebrum, unspecified, with loss of consciousness greater than 24 hours without return to pre-existing conscious level with patient surviving, sequela |
| S06337A | | ICD10 |  | Contusion and laceration of cerebrum, unspecified, with loss of consciousness of any duration with death due to brain injury prior to regaining consciousness, initial encounter |
| S06338A | | ICD10 |  | Contusion and laceration of cerebrum, unspecified, with loss of consciousness of any duration with death due to other cause prior to regaining consciousness, initial encounter |
| S06339A | | ICD10 |  | Contusion and laceration of cerebrum, unspecified, with loss of consciousness of unspecified duration, initial encounter |
| S06339D | | ICD10 |  | Contusion and laceration of cerebrum, unspecified, with loss of consciousness of unspecified duration, subsequent encounter |
| S06339S | | ICD10 |  | Contusion and laceration of cerebrum, unspecified, with loss of consciousness of unspecified duration, sequela |
| S06340A | | ICD10 |  | Traumatic hemorrhage of right cerebrum without loss of consciousness, initial encounter |
| S06340D | | ICD10 |  | Traumatic hemorrhage of right cerebrum without loss of consciousness, subsequent encounter |
| S06340S | | ICD10 |  | Traumatic hemorrhage of right cerebrum without loss of consciousness, sequela |
| S06341A | | ICD10 |  | Traumatic hemorrhage of right cerebrum with loss of consciousness of 30 minutes or less, initial encounter |
| S06341D | | ICD10 |  | Traumatic hemorrhage of right cerebrum with loss of consciousness of 30 minutes or less, subsequent encounter |
| S06341S | | ICD10 |  | Traumatic hemorrhage of right cerebrum with loss of consciousness of 30 minutes or less, sequela |
| S06342A | | ICD10 |  | Traumatic hemorrhage of right cerebrum with loss of consciousness of 31 minutes to 59 minutes, initial encounter |
| S06342D | | ICD10 |  | Traumatic hemorrhage of right cerebrum with loss of consciousness of 31 minutes to 59 minutes, subsequent encounter |
| S06342S | | ICD10 |  | Traumatic hemorrhage of right cerebrum with loss of consciousness of 31 minutes to 59 minutes, sequela |
| S06343A | | ICD10 |  | Traumatic hemorrhage of right cerebrum with loss of consciousness of 1 hours to 5 hours 59 minutes, initial encounter |
| S06343D | | ICD10 |  | Traumatic hemorrhage of right cerebrum with loss of consciousness of 1 hours to 5 hours 59 minutes, subsequent encounter |
| S06343S | | ICD10 |  | Traumatic hemorrhage of right cerebrum with loss of consciousness of 1 hours to 5 hours 59 minutes, sequela |
| S06344A | | ICD10 |  | Traumatic hemorrhage of right cerebrum with loss of consciousness of 6 hours to 24 hours, initial encounter |
| S06344D | | ICD10 |  | Traumatic hemorrhage of right cerebrum with loss of consciousness of 6 hours to 24 hours, subsequent encounter |
| S06344S | | ICD10 |  | Traumatic hemorrhage of right cerebrum with loss of consciousness of 6 hours to 24 hours, sequela |
| S06345A | | ICD10 |  | Traumatic hemorrhage of right cerebrum with loss of consciousness greater than 24 hours with return to pre-existing conscious level, initial encounter |
| S06345D | | ICD10 |  | Traumatic hemorrhage of right cerebrum with loss of consciousness greater than 24 hours with return to pre-existing conscious level, subsequent encounter |
| S06345S | | ICD10 |  | Traumatic hemorrhage of right cerebrum with loss of consciousness greater than 24 hours with return to pre-existing conscious level, sequela |
| S06346A | | ICD10 |  | Traumatic hemorrhage of right cerebrum with loss of consciousness greater than 24 hours without return to pre-existing conscious level with patient surviving, initial encounter |
| S06346D | | ICD10 |  | Traumatic hemorrhage of right cerebrum with loss of consciousness greater than 24 hours without return to pre-existing conscious level with patient surviving, subsequent encounter |
| S06346S | | ICD10 |  | Traumatic hemorrhage of right cerebrum with loss of consciousness greater than 24 hours without return to pre-existing conscious level with patient surviving, sequela |
| S06347A | | ICD10 |  | Traumatic hemorrhage of right cerebrum with loss of consciousness of any duration with death due to brain injury prior to regaining consciousness, initial encounter |
| S06348A | | ICD10 |  | Traumatic hemorrhage of right cerebrum with loss of consciousness of any duration with death due to other cause prior to regaining consciousness, initial encounter |
| S06349A | | ICD10 |  | Traumatic hemorrhage of right cerebrum with loss of consciousness of unspecified duration, initial encounter |
| S06349D | | ICD10 |  | Traumatic hemorrhage of right cerebrum with loss of consciousness of unspecified duration, subsequent encounter |
| S06349S | | ICD10 |  | Traumatic hemorrhage of right cerebrum with loss of consciousness of unspecified duration, sequela |
| S06350A | | ICD10 |  | Traumatic hemorrhage of left cerebrum without loss of consciousness, initial encounter |
| S06350D | | ICD10 |  | Traumatic hemorrhage of left cerebrum without loss of consciousness, subsequent encounter |
| S06350S | | ICD10 |  | Traumatic hemorrhage of left cerebrum without loss of consciousness, sequela |
| S06351A | | ICD10 |  | Traumatic hemorrhage of left cerebrum with loss of consciousness of 30 minutes or less, initial encounter |
| S06351D | | ICD10 |  | Traumatic hemorrhage of left cerebrum with loss of consciousness of 30 minutes or less, subsequent encounter |
| S06351S | | ICD10 |  | Traumatic hemorrhage of left cerebrum with loss of consciousness of 30 minutes or less, sequela |
| S06352A | | ICD10 |  | Traumatic hemorrhage of left cerebrum with loss of consciousness of 31 minutes to 59 minutes, initial encounter |
| S06352D | | ICD10 |  | Traumatic hemorrhage of left cerebrum with loss of consciousness of 31 minutes to 59 minutes, subsequent encounter |
| S06352S | | ICD10 |  | Traumatic hemorrhage of left cerebrum with loss of consciousness of 31 minutes to 59 minutes, sequela |
| S06353A | | ICD10 |  | Traumatic hemorrhage of left cerebrum with loss of consciousness of 1 hours to 5 hours 59 minutes, initial encounter |
| S06353D | | ICD10 |  | Traumatic hemorrhage of left cerebrum with loss of consciousness of 1 hours to 5 hours 59 minutes, subsequent encounter |
| S06353S | | ICD10 |  | Traumatic hemorrhage of left cerebrum with loss of consciousness of 1 hours to 5 hours 59 minutes, sequela |
| V425 | | ICD10 |  | Cornea replaced by transplant |
| S06354A | | ICD10 |  | Traumatic hemorrhage of left cerebrum with loss of consciousness of 6 hours to 24 hours, initial encounter |
| S06354D | | ICD10 |  | Traumatic hemorrhage of left cerebrum with loss of consciousness of 6 hours to 24 hours, subsequent encounter |
| S06354S | | ICD10 |  | Traumatic hemorrhage of left cerebrum with loss of consciousness of 6 hours to 24 hours, sequela |
| S06355A | | ICD10 |  | Traumatic hemorrhage of left cerebrum with loss of consciousness greater than 24 hours with return to pre-existing conscious level, initial encounter |
| S06355D | | ICD10 |  | Traumatic hemorrhage of left cerebrum with loss of consciousness greater than 24 hours with return to pre-existing conscious level, subsequent encounter |
| S06355S | | ICD10 |  | Traumatic hemorrhage of left cerebrum with loss of consciousness greater than 24 hours with return to pre-existing conscious level, sequela |
| S06356A | | ICD10 |  | Traumatic hemorrhage of left cerebrum with loss of consciousness greater than 24 hours without return to pre-existing conscious level with patient surviving, initial encounter |
| S06356D | | ICD10 |  | Traumatic hemorrhage of left cerebrum with loss of consciousness greater than 24 hours without return to pre-existing conscious level with patient surviving, subsequent encounter |
| S06356S | | ICD10 |  | Traumatic hemorrhage of left cerebrum with loss of consciousness greater than 24 hours without return to pre-existing conscious level with patient surviving, sequela |
| S06357A | | ICD10 |  | Traumatic hemorrhage of left cerebrum with loss of consciousness of any duration with death due to brain injury prior to regaining consciousness, initial encounter |
| S06358A | | ICD10 |  | Traumatic hemorrhage of left cerebrum with loss of consciousness of any duration with death due to other cause prior to regaining consciousness, initial encounter |
| S06359A | | ICD10 |  | Traumatic hemorrhage of left cerebrum with loss of consciousness of unspecified duration, initial encounter |
| S06359D | | ICD10 |  | Traumatic hemorrhage of left cerebrum with loss of consciousness of unspecified duration, subsequent encounter |
| S06359S | | ICD10 |  | Traumatic hemorrhage of left cerebrum with loss of consciousness of unspecified duration, sequela |
| S06360A | | ICD10 |  | Traumatic hemorrhage of cerebrum, unspecified, without loss of consciousness, initial encounter |
| S06360D | | ICD10 |  | Traumatic hemorrhage of cerebrum, unspecified, without loss of consciousness, subsequent encounter |
| S06360S | | ICD10 |  | Traumatic hemorrhage of cerebrum, unspecified, without loss of consciousness, sequela |
| S06361A | | ICD10 |  | Traumatic hemorrhage of cerebrum, unspecified, with loss of consciousness of 30 minutes or less, initial encounter |
| S06361D | | ICD10 |  | Traumatic hemorrhage of cerebrum, unspecified, with loss of consciousness of 30 minutes or less, subsequent encounter |
| S06361S | | ICD10 |  | Traumatic hemorrhage of cerebrum, unspecified, with loss of consciousness of 30 minutes or less, sequela |
| S06362A | | ICD10 |  | Traumatic hemorrhage of cerebrum, unspecified, with loss of consciousness of 31 minutes to 59 minutes, initial encounter |
| S06362D | | ICD10 |  | Traumatic hemorrhage of cerebrum, unspecified, with loss of consciousness of 31 minutes to 59 minutes, subsequent encounter |
| S06362S | | ICD10 |  | Traumatic hemorrhage of cerebrum, unspecified, with loss of consciousness of 31 minutes to 59 minutes, sequela |
| S06363A | | ICD10 |  | Traumatic hemorrhage of cerebrum, unspecified, with loss of consciousness of 1 hours to 5 hours 59 minutes, initial encounter |
| S06363D | | ICD10 |  | Traumatic hemorrhage of cerebrum, unspecified, with loss of consciousness of 1 hours to 5 hours 59 minutes, subsequent encounter |
| S06363S | | ICD10 |  | Traumatic hemorrhage of cerebrum, unspecified, with loss of consciousness of 1 hours to 5 hours 59 minutes, sequela |
| S06364A | | ICD10 |  | Traumatic hemorrhage of cerebrum, unspecified, with loss of consciousness of 6 hours to 24 hours, initial encounter |
| S06364D | | ICD10 |  | Traumatic hemorrhage of cerebrum, unspecified, with loss of consciousness of 6 hours to 24 hours, subsequent encounter |
| S06364S | | ICD10 |  | Traumatic hemorrhage of cerebrum, unspecified, with loss of consciousness of 6 hours to 24 hours, sequela |
| S06365A | | ICD10 |  | Traumatic hemorrhage of cerebrum, unspecified, with loss of consciousness greater than 24 hours with return to pre-existing conscious level, initial encounter |
| S06365D | | ICD10 |  | Traumatic hemorrhage of cerebrum, unspecified, with loss of consciousness greater than 24 hours with return to pre-existing conscious level, subsequent encounter |
| S06365S | | ICD10 |  | Traumatic hemorrhage of cerebrum, unspecified, with loss of consciousness greater than 24 hours with return to pre-existing conscious level, sequela |
| S06366A | | ICD10 |  | Traumatic hemorrhage of cerebrum, unspecified, with loss of consciousness greater than 24 hours without return to pre-existing conscious level with patient surviving, initial encounter |
| S06366D | | ICD10 |  | Traumatic hemorrhage of cerebrum, unspecified, with loss of consciousness greater than 24 hours without return to pre-existing conscious level with patient surviving, subsequent encounter |
| S06366S | | ICD10 |  | Traumatic hemorrhage of cerebrum, unspecified, with loss of consciousness greater than 24 hours without return to pre-existing conscious level with patient surviving, sequela |
| S06367A | | ICD10 |  | Traumatic hemorrhage of cerebrum, unspecified, with loss of consciousness of any duration with death due to brain injury prior to regaining consciousness, initial encounter |
| S06368A | | ICD10 |  | Traumatic hemorrhage of cerebrum, unspecified, with loss of consciousness of any duration with death due to other cause prior to regaining consciousness, initial encounter |
| S06369A | | ICD10 |  | Traumatic hemorrhage of cerebrum, unspecified, with loss of consciousness of unspecified duration, initial encounter |
| S06369D | | ICD10 |  | Traumatic hemorrhage of cerebrum, unspecified, with loss of consciousness of unspecified duration, subsequent encounter |
| S06369S | | ICD10 |  | Traumatic hemorrhage of cerebrum, unspecified, with loss of consciousness of unspecified duration, sequela |
| S06370A | | ICD10 |  | Contusion, laceration, and hemorrhage of cerebellum without loss of consciousness, initial encounter |
| S06370D | | ICD10 |  | Contusion, laceration, and hemorrhage of cerebellum without loss of consciousness, subsequent encounter |
| S06370S | | ICD10 |  | Contusion, laceration, and hemorrhage of cerebellum without loss of consciousness, sequela |
| S06371A | | ICD10 |  | Contusion, laceration, and hemorrhage of cerebellum with loss of consciousness of 30 minutes or less, initial encounter |
| S06371D | | ICD10 |  | Contusion, laceration, and hemorrhage of cerebellum with loss of consciousness of 30 minutes or less, subsequent encounter |
| S06371S | | ICD10 |  | Contusion, laceration, and hemorrhage of cerebellum with loss of consciousness of 30 minutes or less, sequela |
| S06372A | | ICD10 |  | Contusion, laceration, and hemorrhage of cerebellum with loss of consciousness of 31 minutes to 59 minutes, initial encounter |
| S06372D | | ICD10 |  | Contusion, laceration, and hemorrhage of cerebellum with loss of consciousness of 31 minutes to 59 minutes, subsequent encounter |
| S06372S | | ICD10 |  | Contusion, laceration, and hemorrhage of cerebellum with loss of consciousness of 31 minutes to 59 minutes, sequela |
| S06373A | | ICD10 |  | Contusion, laceration, and hemorrhage of cerebellum with loss of consciousness of 1 hour to 5 hours 59 minutes, initial encounter |
| S06373D | | ICD10 |  | Contusion, laceration, and hemorrhage of cerebellum with loss of consciousness of 1 hour to 5 hours 59 minutes, subsequent encounter |
| S06373S | | ICD10 |  | Contusion, laceration, and hemorrhage of cerebellum with loss of consciousness of 1 hour to 5 hours 59 minutes, sequela |
| S06374A | | ICD10 |  | Contusion, laceration, and hemorrhage of cerebellum with loss of consciousness of 6 hours to 24 hours, initial encounter |
| S06374D | | ICD10 |  | Contusion, laceration, and hemorrhage of cerebellum with loss of consciousness of 6 hours to 24 hours, subsequent encounter |
| S06374S | | ICD10 |  | Contusion, laceration, and hemorrhage of cerebellum with loss of consciousness of 6 hours to 24 hours, sequela |
| S06375A | | ICD10 |  | Contusion, laceration, and hemorrhage of cerebellum with loss of consciousness greater than 24 hours with return to pre-existing conscious level, initial encounter |
| S06375D | | ICD10 |  | Contusion, laceration, and hemorrhage of cerebellum with loss of consciousness greater than 24 hours with return to pre-existing conscious level, subsequent encounter |
| S06375S | | ICD10 |  | Contusion, laceration, and hemorrhage of cerebellum with loss of consciousness greater than 24 hours with return to pre-existing conscious level, sequela |
| S06376A | | ICD10 |  | Contusion, laceration, and hemorrhage of cerebellum with loss of consciousness greater than 24 hours without return to pre-existing conscious level with patient surviving, initial encounter |
| S06376D | | ICD10 |  | Contusion, laceration, and hemorrhage of cerebellum with loss of consciousness greater than 24 hours without return to pre-existing conscious level with patient surviving, subsequent encounter |
| S06376S | | ICD10 |  | Contusion, laceration, and hemorrhage of cerebellum with loss of consciousness greater than 24 hours without return to pre-existing conscious level with patient surviving, sequela |
| S06377A | | ICD10 |  | Contusion, laceration, and hemorrhage of cerebellum with loss of consciousness of any duration with death due to brain injury prior to regaining consciousness, initial encounter |
| S06378A | | ICD10 |  | Contusion, laceration, and hemorrhage of cerebellum with loss of consciousness of any duration with death due to other cause prior to regaining consciousness, initial encounter |
| S06379A | | ICD10 |  | Contusion, laceration, and hemorrhage of cerebellum with loss of consciousness of unspecified duration, initial encounter |
| S06379D | | ICD10 |  | Contusion, laceration, and hemorrhage of cerebellum with loss of consciousness of unspecified duration, subsequent encounter |
| S06379S | | ICD10 |  | Contusion, laceration, and hemorrhage of cerebellum with loss of consciousness of unspecified duration, sequela |
| S06380A | | ICD10 |  | Contusion, laceration, and hemorrhage of brainstem without loss of consciousness, initial encounter |
| S06380D | | ICD10 |  | Contusion, laceration, and hemorrhage of brainstem without loss of consciousness, subsequent encounter |
| S06380S | | ICD10 |  | Contusion, laceration, and hemorrhage of brainstem without loss of consciousness, sequela |
| S06381A | | ICD10 |  | Contusion, laceration, and hemorrhage of brainstem with loss of consciousness of 30 minutes or less, initial encounter |
| S06381D | | ICD10 |  | Contusion, laceration, and hemorrhage of brainstem with loss of consciousness of 30 minutes or less, subsequent encounter |
| S06381S | | ICD10 |  | Contusion, laceration, and hemorrhage of brainstem with loss of consciousness of 30 minutes or less, sequela |
| S06382A | | ICD10 |  | Contusion, laceration, and hemorrhage of brainstem with loss of consciousness of 31 minutes to 59 minutes, initial encounter |
| S06382D | | ICD10 |  | Contusion, laceration, and hemorrhage of brainstem with loss of consciousness of 31 minutes to 59 minutes, subsequent encounter |
| S06382S | | ICD10 |  | Contusion, laceration, and hemorrhage of brainstem with loss of consciousness of 31 minutes to 59 minutes, sequela |
| S06383A | | ICD10 |  | Contusion, laceration, and hemorrhage of brainstem with loss of consciousness of 1 hour to 5 hours 59 minutes, initial encounter |
| S06383D | | ICD10 |  | Contusion, laceration, and hemorrhage of brainstem with loss of consciousness of 1 hour to 5 hours 59 minutes, subsequent encounter |
| S06383S | | ICD10 |  | Contusion, laceration, and hemorrhage of brainstem with loss of consciousness of 1 hour to 5 hours 59 minutes, sequela |
| S06384A | | ICD10 |  | Contusion, laceration, and hemorrhage of brainstem with loss of consciousness of 6 hours to 24 hours, initial encounter |
| S06384D | | ICD10 |  | Contusion, laceration, and hemorrhage of brainstem with loss of consciousness of 6 hours to 24 hours, subsequent encounter |
| S06384S | | ICD10 |  | Contusion, laceration, and hemorrhage of brainstem with loss of consciousness of 6 hours to 24 hours, sequela |
| S06385A | | ICD10 |  | Contusion, laceration, and hemorrhage of brainstem with loss of consciousness greater than 24 hours with return to pre-existing conscious level, initial encounter |
| S06385D | | ICD10 |  | Contusion, laceration, and hemorrhage of brainstem with loss of consciousness greater than 24 hours with return to pre-existing conscious level, subsequent encounter |
| S06385S | | ICD10 |  | Contusion, laceration, and hemorrhage of brainstem with loss of consciousness greater than 24 hours with return to pre-existing conscious level, sequela |
| S06386A | | ICD10 |  | Contusion, laceration, and hemorrhage of brainstem with loss of consciousness greater than 24 hours without return to pre-existing conscious level with patient surviving, initial encounter |
| S06386D | | ICD10 |  | Contusion, laceration, and hemorrhage of brainstem with loss of consciousness greater than 24 hours without return to pre-existing conscious level with patient surviving, subsequent encounter |
| S06386S | | ICD10 |  | Contusion, laceration, and hemorrhage of brainstem with loss of consciousness greater than 24 hours without return to pre-existing conscious level with patient surviving, sequela |
| S06387A | | ICD10 |  | Contusion, laceration, and hemorrhage of brainstem with loss of consciousness of any duration with death due to brain injury prior to regaining consciousness, initial encounter |
| S06388A | | ICD10 |  | Contusion, laceration, and hemorrhage of brainstem with loss of consciousness of any duration with death due to other cause prior to regaining consciousness, initial encounter |
| S06389A | | ICD10 |  | Contusion, laceration, and hemorrhage of brainstem with loss of consciousness of unspecified duration, initial encounter |
| S06389D | | ICD10 |  | Contusion, laceration, and hemorrhage of brainstem with loss of consciousness of unspecified duration, subsequent encounter |
| S078XXS | | ICD10 |  | Crushing injury of other parts of head, sequela |
| S06389S | | ICD10 |  | Contusion, laceration, and hemorrhage of brainstem with loss of consciousness of unspecified duration, sequela |
| S06335D | | ICD10 |  | Contusion and laceration of cerebrum, unspecified, with loss of consciousness greater than 24 hours with return to pre-existing conscious level, subsequent encounter |
| S06335S | | ICD10 |  | Contusion and laceration of cerebrum, unspecified, with loss of consciousness greater than 24 hours with return to pre-existing conscious level, sequela |
| S06355A | | ICD10 |  | Traumatic hemorrhage of left cerebrum with loss of consciousness greater than 24 hours with return to pre-existing conscious level, initial encounter |
| S06355D | | ICD10 |  | Traumatic hemorrhage of left cerebrum with loss of consciousness greater than 24 hours with return to pre-existing conscious level, subsequent encounter |
| S064X0A | | ICD10 |  | Epidural hemorrhage without loss of consciousness, initial encounter |
| S064X0D | | ICD10 |  | Epidural hemorrhage without loss of consciousness, subsequent encounter |
| S064X0S | | ICD10 |  | Epidural hemorrhage without loss of consciousness, sequela |
| S064X1A | | ICD10 |  | Epidural hemorrhage with loss of consciousness of 30 minutes or less, initial encounter |
| S064X1D | | ICD10 |  | Epidural hemorrhage with loss of consciousness of 30 minutes or less, subsequent encounter |
| S064X1S | | ICD10 |  | Epidural hemorrhage with loss of consciousness of 30 minutes or less, sequela |
| S064X2A | | ICD10 |  | Epidural hemorrhage with loss of consciousness of 31 minutes to 59 minutes, initial encounter |
| S064X2D | | ICD10 |  | Epidural hemorrhage with loss of consciousness of 31 minutes to 59 minutes, subsequent encounter |
| S064X2S | | ICD10 |  | Epidural hemorrhage with loss of consciousness of 31 minutes to 59 minutes, sequela |
| S064X3A | | ICD10 |  | Epidural hemorrhage with loss of consciousness of 1 hour to 5 hours 59 minutes, initial encounter |
| S064X3D | | ICD10 |  | Epidural hemorrhage with loss of consciousness of 1 hour to 5 hours 59 minutes, subsequent encounter |
| S064X3S | | ICD10 |  | Epidural hemorrhage with loss of consciousness of 1 hour to 5 hours 59 minutes, sequela |
| S064X4A | | ICD10 |  | Epidural hemorrhage with loss of consciousness of 6 hours to 24 hours, initial encounter |
| S064X4D | | ICD10 |  | Epidural hemorrhage with loss of consciousness of 6 hours to 24 hours, subsequent encounter |
| S064X4S | | ICD10 |  | Epidural hemorrhage with loss of consciousness of 6 hours to 24 hours, sequela |
| S064X5A | | ICD10 |  | Epidural hemorrhage with loss of consciousness greater than 24 hours with return to pre-existing conscious level, initial encounter |
| S064X5D | | ICD10 |  | Epidural hemorrhage with loss of consciousness greater than 24 hours with return to pre-existing conscious level, subsequent encounter |
| S064X5S | | ICD10 |  | Epidural hemorrhage with loss of consciousness greater than 24 hours with return to pre-existing conscious level, sequela |
| S064X6A | | ICD10 |  | Epidural hemorrhage with loss of consciousness greater than 24 hours without return to pre-existing conscious level with patient surviving, initial encounter |
| S064X6D | | ICD10 |  | Epidural hemorrhage with loss of consciousness greater than 24 hours without return to pre-existing conscious level with patient surviving, subsequent encounter |
| S064X6S | | ICD10 |  | Epidural hemorrhage with loss of consciousness greater than 24 hours without return to pre-existing conscious level with patient surviving, sequela |
| S064X7A | | ICD10 |  | Epidural hemorrhage with loss of consciousness of any duration with death due to brain injury prior to regaining consciousness, initial encounter |
| S064X8A | | ICD10 |  | Epidural hemorrhage with loss of consciousness of any duration with death due to other causes prior to regaining consciousness, initial encounter |
| S064X9A | | ICD10 |  | Epidural hemorrhage with loss of consciousness of unspecified duration, initial encounter |
| S064X9D | | ICD10 |  | Epidural hemorrhage with loss of consciousness of unspecified duration, subsequent encounter |
| S064X9S | | ICD10 |  | Epidural hemorrhage with loss of consciousness of unspecified duration, sequela |
| S065X0A | | ICD10 |  | Traumatic subdural hemorrhage without loss of consciousness, initial encounter |
| S065X0D | | ICD10 |  | Traumatic subdural hemorrhage without loss of consciousness, subsequent encounter |
| S065X0S | | ICD10 |  | Traumatic subdural hemorrhage without loss of consciousness, sequela |
| S065X1A | | ICD10 |  | Traumatic subdural hemorrhage with loss of consciousness of 30 minutes or less, initial encounter |
| S065X1D | | ICD10 |  | Traumatic subdural hemorrhage with loss of consciousness of 30 minutes or less, subsequent encounter |
| S065X1S | | ICD10 |  | Traumatic subdural hemorrhage with loss of consciousness of 30 minutes or less, sequela |
| S065X2A | | ICD10 |  | Traumatic subdural hemorrhage with loss of consciousness of 31 minutes to 59 minutes, initial encounter |
| S065X2D | | ICD10 |  | Traumatic subdural hemorrhage with loss of consciousness of 31 minutes to 59 minutes, subsequent encounter |
| S065X2S | | ICD10 |  | Traumatic subdural hemorrhage with loss of consciousness of 31 minutes to 59 minutes, sequela |
| S065X3A | | ICD10 |  | Traumatic subdural hemorrhage with loss of consciousness of 1 hour to 5 hours 59 minutes, initial encounter |
| S065X3D | | ICD10 |  | Traumatic subdural hemorrhage with loss of consciousness of 1 hour to 5 hours 59 minutes, subsequent encounter |
| S065X3S | | ICD10 |  | Traumatic subdural hemorrhage with loss of consciousness of 1 hour to 5 hours 59 minutes, sequela |
| S065X4A | | ICD10 |  | Traumatic subdural hemorrhage with loss of consciousness of 6 hours to 24 hours, initial encounter |
| S065X4D | | ICD10 |  | Traumatic subdural hemorrhage with loss of consciousness of 6 hours to 24 hours, subsequent encounter |
| S065X4S | | ICD10 |  | Traumatic subdural hemorrhage with loss of consciousness of 6 hours to 24 hours, sequela |
| S065X5A | | ICD10 |  | Traumatic subdural hemorrhage with loss of consciousness greater than 24 hours with return to pre-existing conscious level, initial encounter |
| S065X5D | | ICD10 |  | Traumatic subdural hemorrhage with loss of consciousness greater than 24 hours with return to pre-existing conscious level, subsequent encounter |
| S065X5S | | ICD10 |  | Traumatic subdural hemorrhage with loss of consciousness greater than 24 hours with return to pre-existing conscious level, sequela |
| S065X6A | | ICD10 |  | Traumatic subdural hemorrhage with loss of consciousness greater than 24 hours without return to pre-existing conscious level with patient surviving, initial encounter |
| S065X6D | | ICD10 |  | Traumatic subdural hemorrhage with loss of consciousness greater than 24 hours without return to pre-existing conscious level with patient surviving, subsequent encounter |
| S065X6S | | ICD10 |  | Traumatic subdural hemorrhage with loss of consciousness greater than 24 hours without return to pre-existing conscious level with patient surviving, sequela |
| S065X7A | | ICD10 |  | Traumatic subdural hemorrhage with loss of consciousness of any duration with death due to brain injury before regaining consciousness, initial encounter |
| S065X8A | | ICD10 |  | Traumatic subdural hemorrhage with loss of consciousness of any duration with death due to other cause before regaining consciousness, initial encounter |
| S065X9A | | ICD10 |  | Traumatic subdural hemorrhage with loss of consciousness of unspecified duration, initial encounter |
| S065X9D | | ICD10 |  | Traumatic subdural hemorrhage with loss of consciousness of unspecified duration, subsequent encounter |
| S065X9S | | ICD10 |  | Traumatic subdural hemorrhage with loss of consciousness of unspecified duration, sequela |
| S066X0A | | ICD10 |  | Traumatic subarachnoid hemorrhage without loss of consciousness, initial encounter |
| S066X0D | | ICD10 |  | Traumatic subarachnoid hemorrhage without loss of consciousness, subsequent encounter |
| S066X0S | | ICD10 |  | Traumatic subarachnoid hemorrhage without loss of consciousness, sequela |
| S066X1A | | ICD10 |  | Traumatic subarachnoid hemorrhage with loss of consciousness of 30 minutes or less, initial encounter |
| S066X1D | | ICD10 |  | Traumatic subarachnoid hemorrhage with loss of consciousness of 30 minutes or less, subsequent encounter |
| S066X1S | | ICD10 |  | Traumatic subarachnoid hemorrhage with loss of consciousness of 30 minutes or less, sequela |
| S066X2A | | ICD10 |  | Traumatic subarachnoid hemorrhage with loss of consciousness of 31 minutes to 59 minutes, initial encounter |
| S066X2D | | ICD10 |  | Traumatic subarachnoid hemorrhage with loss of consciousness of 31 minutes to 59 minutes, subsequent encounter |
| S066X2S | | ICD10 |  | Traumatic subarachnoid hemorrhage with loss of consciousness of 31 minutes to 59 minutes, sequela |
| S066X3A | | ICD10 |  | Traumatic subarachnoid hemorrhage with loss of consciousness of 1 hour to 5 hours 59 minutes, initial encounter |
| S066X3D | | ICD10 |  | Traumatic subarachnoid hemorrhage with loss of consciousness of 1 hour to 5 hours 59 minutes, subsequent encounter |
| S066X3S | | ICD10 |  | Traumatic subarachnoid hemorrhage with loss of consciousness of 1 hour to 5 hours 59 minutes, sequela |
| S066X4A | | ICD10 |  | Traumatic subarachnoid hemorrhage with loss of consciousness of 6 hours to 24 hours, initial encounter |
| S066X4D | | ICD10 |  | Traumatic subarachnoid hemorrhage with loss of consciousness of 6 hours to 24 hours, subsequent encounter |
| S066X4S | | ICD10 |  | Traumatic subarachnoid hemorrhage with loss of consciousness of 6 hours to 24 hours, sequela |
| S066X5A | | ICD10 |  | Traumatic subarachnoid hemorrhage with loss of consciousness greater than 24 hours with return to pre-existing conscious level, initial encounter |
| S066X5D | | ICD10 |  | Traumatic subarachnoid hemorrhage with loss of consciousness greater than 24 hours with return to pre-existing conscious level, subsequent encounter |
| S066X5S | | ICD10 |  | Traumatic subarachnoid hemorrhage with loss of consciousness greater than 24 hours with return to pre-existing conscious level, sequela |
| S066X6A | | ICD10 |  | Traumatic subarachnoid hemorrhage with loss of consciousness greater than 24 hours without return to pre-existing conscious level with patient surviving, initial encounter |
| S066X6D | | ICD10 |  | Traumatic subarachnoid hemorrhage with loss of consciousness greater than 24 hours without return to pre-existing conscious level with patient surviving, subsequent encounter |
| S066X6S | | ICD10 |  | Traumatic subarachnoid hemorrhage with loss of consciousness greater than 24 hours without return to pre-existing conscious level with patient surviving, sequela |
| S066X7A | | ICD10 |  | Traumatic subarachnoid hemorrhage with loss of consciousness of any duration with death due to brain injury prior to regaining consciousness, initial encounter |
| S066X8A | | ICD10 |  | Traumatic subarachnoid hemorrhage with loss of consciousness of any duration with death due to other cause prior to regaining consciousness, initial encounter |
| S066X9A | | ICD10 |  | Traumatic subarachnoid hemorrhage with loss of consciousness of unspecified duration, initial encounter |
| S066X9D | | ICD10 |  | Traumatic subarachnoid hemorrhage with loss of consciousness of unspecified duration, subsequent encounter |
| S066X9S | | ICD10 |  | Traumatic subarachnoid hemorrhage with loss of consciousness of unspecified duration, sequela |
| S06810A | | ICD10 |  | Injury of right internal carotid artery, intracranial portion, not elsewhere classified without loss of consciousness, initial encounter |
| S06810D | | ICD10 |  | Injury of right internal carotid artery, intracranial portion, not elsewhere classified without loss of consciousness, subsequent encounter |
| S06810S | | ICD10 |  | Injury of right internal carotid artery, intracranial portion, not elsewhere classified without loss of consciousness, sequela |
| S06811A | | ICD10 |  | Injury of right internal carotid artery, intracranial portion, not elsewhere classified with loss of consciousness of 30 minutes or less, initial encounter |
| S06811D | | ICD10 |  | Injury of right internal carotid artery, intracranial portion, not elsewhere classified with loss of consciousness of 30 minutes or less, subsequent encounter |
| S06811S | | ICD10 |  | Injury of right internal carotid artery, intracranial portion, not elsewhere classified with loss of consciousness of 30 minutes or less, sequela |
| S06812A | | ICD10 |  | Injury of right internal carotid artery, intracranial portion, not elsewhere classified with loss of consciousness of 31 minutes to 59 minutes, initial encounter |
| S06812D | | ICD10 |  | Injury of right internal carotid artery, intracranial portion, not elsewhere classified with loss of consciousness of 31 minutes to 59 minutes, subsequent encounter |
| S06812S | | ICD10 |  | Injury of right internal carotid artery, intracranial portion, not elsewhere classified with loss of consciousness of 31 minutes to 59 minutes, sequela |
| S06813A | | ICD10 |  | Injury of right internal carotid artery, intracranial portion, not elsewhere classified with loss of consciousness of 1 hour to 5 hours 59 minutes, initial encounter |
| S06813D | | ICD10 |  | Injury of right internal carotid artery, intracranial portion, not elsewhere classified with loss of consciousness of 1 hour to 5 hours 59 minutes, subsequent encounter |
| S06813S | | ICD10 |  | Injury of right internal carotid artery, intracranial portion, not elsewhere classified with loss of consciousness of 1 hour to 5 hours 59 minutes, sequela |
| S06814A | | ICD10 |  | Injury of right internal carotid artery, intracranial portion, not elsewhere classified with loss of consciousness of 6 hours to 24 hours, initial encounter |
| S06814D | | ICD10 |  | Injury of right internal carotid artery, intracranial portion, not elsewhere classified with loss of consciousness of 6 hours to 24 hours, subsequent encounter |
| S06814S | | ICD10 |  | Injury of right internal carotid artery, intracranial portion, not elsewhere classified with loss of consciousness of 6 hours to 24 hours, sequela |
| S06815A | | ICD10 |  | Injury of right internal carotid artery, intracranial portion, not elsewhere classified with loss of consciousness greater than 24 hours with return to pre-existing conscious level, initial encounter |
| S06815D | | ICD10 |  | Injury of right internal carotid artery, intracranial portion, not elsewhere classified with loss of consciousness greater than 24 hours with return to pre-existing conscious level, subsequent encounter |
| S06815S | | ICD10 |  | Injury of right internal carotid artery, intracranial portion, not elsewhere classified with loss of consciousness greater than 24 hours with return to pre-existing conscious level, sequela |
| S06816A | | ICD10 |  | Injury of right internal carotid artery, intracranial portion, not elsewhere classified with loss of consciousness greater than 24 hours without return to pre-existing conscious level with patient surviving, initial encounter |
| S079XXA | | ICD10 |  | Crushing injury of head, part unspecified, initial encounter |
| S06816D | | ICD10 |  | Injury of right internal carotid artery, intracranial portion, not elsewhere classified with loss of consciousness greater than 24 hours without return to pre-existing conscious level with patient surviving, subsequent encounter |
| S06816S | | ICD10 |  | Injury of right internal carotid artery, intracranial portion, not elsewhere classified with loss of consciousness greater than 24 hours without return to pre-existing conscious level with patient surviving, sequela |
| S06817A | | ICD10 |  | Injury of right internal carotid artery, intracranial portion, not elsewhere classified with loss of consciousness of any duration with death due to brain injury prior to regaining consciousness, initial encounter |
| S06818A | | ICD10 |  | Injury of right internal carotid artery, intracranial portion, not elsewhere classified with loss of consciousness of any duration with death due to other cause prior to regaining consciousness, initial encounter |
| S06819A | | ICD10 |  | Injury of right internal carotid artery, intracranial portion, not elsewhere classified with loss of consciousness of unspecified duration, initial encounter |
| S06819D | | ICD10 |  | Injury of right internal carotid artery, intracranial portion, not elsewhere classified with loss of consciousness of unspecified duration, subsequent encounter |
| S06819S | | ICD10 |  | Injury of right internal carotid artery, intracranial portion, not elsewhere classified with loss of consciousness of unspecified duration, sequela |
| S06820A | | ICD10 |  | Injury of left internal carotid artery, intracranial portion, not elsewhere classified without loss of consciousness, initial encounter |
| S06820D | | ICD10 |  | Injury of left internal carotid artery, intracranial portion, not elsewhere classified without loss of consciousness, subsequent encounter |
| S06820S | | ICD10 |  | Injury of left internal carotid artery, intracranial portion, not elsewhere classified without loss of consciousness, sequela |
| S06821A | | ICD10 |  | Injury of left internal carotid artery, intracranial portion, not elsewhere classified with loss of consciousness of 30 minutes or less, initial encounter |
| S06821D | | ICD10 |  | Injury of left internal carotid artery, intracranial portion, not elsewhere classified with loss of consciousness of 30 minutes or less, subsequent encounter |
| S06821S | | ICD10 |  | Injury of left internal carotid artery, intracranial portion, not elsewhere classified with loss of consciousness of 30 minutes or less, sequela |
| S06822A | | ICD10 |  | Injury of left internal carotid artery, intracranial portion, not elsewhere classified with loss of consciousness of 31 minutes to 59 minutes, initial encounter |
| S06822D | | ICD10 |  | Injury of left internal carotid artery, intracranial portion, not elsewhere classified with loss of consciousness of 31 minutes to 59 minutes, subsequent encounter |
| S06822S | | ICD10 |  | Injury of left internal carotid artery, intracranial portion, not elsewhere classified with loss of consciousness of 31 minutes to 59 minutes, sequela |
| S06823A | | ICD10 |  | Injury of left internal carotid artery, intracranial portion, not elsewhere classified with loss of consciousness of 1 hour to 5 hours 59 minutes, initial encounter |
| S06823D | | ICD10 |  | Injury of left internal carotid artery, intracranial portion, not elsewhere classified with loss of consciousness of 1 hour to 5 hours 59 minutes, subsequent encounter |
| S06823S | | ICD10 |  | Injury of left internal carotid artery, intracranial portion, not elsewhere classified with loss of consciousness of 1 hour to 5 hours 59 minutes, sequela |
| S06824A | | ICD10 |  | Injury of left internal carotid artery, intracranial portion, not elsewhere classified with loss of consciousness of 6 hours to 24 hours, initial encounter |
| S06824D | | ICD10 |  | Injury of left internal carotid artery, intracranial portion, not elsewhere classified with loss of consciousness of 6 hours to 24 hours, subsequent encounter |
| S06824S | | ICD10 |  | Injury of left internal carotid artery, intracranial portion, not elsewhere classified with loss of consciousness of 6 hours to 24 hours, sequela |
| S06825A | | ICD10 |  | Injury of left internal carotid artery, intracranial portion, not elsewhere classified with loss of consciousness greater than 24 hours with return to pre-existing conscious level, initial encounter |
| S06825D | | ICD10 |  | Injury of left internal carotid artery, intracranial portion, not elsewhere classified with loss of consciousness greater than 24 hours with return to pre-existing conscious level, subsequent encounter |
| S06825S | | ICD10 |  | Injury of left internal carotid artery, intracranial portion, not elsewhere classified with loss of consciousness greater than 24 hours with return to pre-existing conscious level, sequela |
| S06826A | | ICD10 |  | Injury of left internal carotid artery, intracranial portion, not elsewhere classified with loss of consciousness greater than 24 hours without return to pre-existing conscious level with patient surviving, initial encounter |
| S06826D | | ICD10 |  | Injury of left internal carotid artery, intracranial portion, not elsewhere classified with loss of consciousness greater than 24 hours without return to pre-existing conscious level with patient surviving, subsequent encounter |
| S06826S | | ICD10 |  | Injury of left internal carotid artery, intracranial portion, not elsewhere classified with loss of consciousness greater than 24 hours without return to pre-existing conscious level with patient surviving, sequela |
| S06827A | | ICD10 |  | Injury of left internal carotid artery, intracranial portion, not elsewhere classified with loss of consciousness of any duration with death due to brain injury prior to regaining consciousness, initial encounter |
| S06828A | | ICD10 |  | Injury of left internal carotid artery, intracranial portion, not elsewhere classified with loss of consciousness of any duration with death due to other cause prior to regaining consciousness, initial encounter |
| S06829A | | ICD10 |  | Injury of left internal carotid artery, intracranial portion, not elsewhere classified with loss of consciousness of unspecified duration, initial encounter |
| S06829D | | ICD10 |  | Injury of left internal carotid artery, intracranial portion, not elsewhere classified with loss of consciousness of unspecified duration, subsequent encounter |
| S06829S | | ICD10 |  | Injury of left internal carotid artery, intracranial portion, not elsewhere classified with loss of consciousness of unspecified duration, sequela |
| S06890A | | ICD10 |  | Other specified intracranial injury without loss of consciousness, initial encounter |
| S06890D | | ICD10 |  | Other specified intracranial injury without loss of consciousness, subsequent encounter |
| S06890S | | ICD10 |  | Other specified intracranial injury without loss of consciousness, sequela |
| S06891A | | ICD10 |  | Other specified intracranial injury with loss of consciousness of 30 minutes or less, initial encounter |
| S06891D | | ICD10 |  | Other specified intracranial injury with loss of consciousness of 30 minutes or less, subsequent encounter |
| S06891S | | ICD10 |  | Other specified intracranial injury with loss of consciousness of 30 minutes or less, sequela |
| W880XXS | | ICD10 |  | Exposure to X-rays, sequela |
| S06892A | | ICD10 |  | Other specified intracranial injury with loss of consciousness of 31 minutes to 59 minutes, initial encounter |
| S06892D | | ICD10 |  | Other specified intracranial injury with loss of consciousness of 31 minutes to 59 minutes, subsequent encounter |
| S06892S | | ICD10 |  | Other specified intracranial injury with loss of consciousness of 31 minutes to 59 minutes, sequela |
| S06893A | | ICD10 |  | Other specified intracranial injury with loss of consciousness of 1 hour to 5 hours 59 minutes, initial encounter |
| S06893D | | ICD10 |  | Other specified intracranial injury with loss of consciousness of 1 hour to 5 hours 59 minutes, subsequent encounter |
| S06893S | | ICD10 |  | Other specified intracranial injury with loss of consciousness of 1 hour to 5 hours 59 minutes, sequela |
| S06894A | | ICD10 |  | Other specified intracranial injury with loss of consciousness of 6 hours to 24 hours, initial encounter |
| S06894D | | ICD10 |  | Other specified intracranial injury with loss of consciousness of 6 hours to 24 hours, subsequent encounter |
| S06894S | | ICD10 |  | Other specified intracranial injury with loss of consciousness of 6 hours to 24 hours, sequela |
| S06895A | | ICD10 |  | Other specified intracranial injury with loss of consciousness greater than 24 hours with return to pre-existing conscious level, initial encounter |
| S06895D | | ICD10 |  | Other specified intracranial injury with loss of consciousness greater than 24 hours with return to pre-existing conscious level, subsequent encounter |
| S06895S | | ICD10 |  | Other specified intracranial injury with loss of consciousness greater than 24 hours with return to pre-existing conscious level, sequela |
| S06896A | | ICD10 |  | Other specified intracranial injury with loss of consciousness greater than 24 hours without return to pre-existing conscious level with patient surviving, initial encounter |
| S06896D | | ICD10 |  | Other specified intracranial injury with loss of consciousness greater than 24 hours without return to pre-existing conscious level with patient surviving, subsequent encounter |
| S06896S | | ICD10 |  | Other specified intracranial injury with loss of consciousness greater than 24 hours without return to pre-existing conscious level with patient surviving, sequela |
| S06897A | | ICD10 |  | Other specified intracranial injury with loss of consciousness of any duration with death due to brain injury prior to regaining consciousness, initial encounter |
| S06898A | | ICD10 |  | Other specified intracranial injury with loss of consciousness of any duration with death due to other cause prior to regaining consciousness, initial encounter |
| S06899A | | ICD10 |  | Other specified intracranial injury with loss of consciousness of unspecified duration, initial encounter |
| S06899D | | ICD10 |  | Other specified intracranial injury with loss of consciousness of unspecified duration, subsequent encounter |
| S06899S | | ICD10 |  | Other specified intracranial injury with loss of consciousness of unspecified duration, sequela |
| S069X0A | | ICD10 |  | Unspecified intracranial injury without loss of consciousness, initial encounter |
| S069X0D | | ICD10 |  | Unspecified intracranial injury without loss of consciousness, subsequent encounter |
| S069X0S | | ICD10 |  | Unspecified intracranial injury without loss of consciousness, sequela |
| S069X1A | | ICD10 |  | Unspecified intracranial injury with loss of consciousness of 30 minutes or less, initial encounter |
| S069X1D | | ICD10 |  | Unspecified intracranial injury with loss of consciousness of 30 minutes or less, subsequent encounter |
| S069X1S | | ICD10 |  | Unspecified intracranial injury with loss of consciousness of 30 minutes or less, sequela |
| S069X2A | | ICD10 |  | Unspecified intracranial injury with loss of consciousness of 31 minutes to 59 minutes, initial encounter |
| S069X2D | | ICD10 |  | Unspecified intracranial injury with loss of consciousness of 31 minutes to 59 minutes, subsequent encounter |
| S069X2S | | ICD10 |  | Unspecified intracranial injury with loss of consciousness of 31 minutes to 59 minutes, sequela |
| S069X3A | | ICD10 |  | Unspecified intracranial injury with loss of consciousness of 1 hour to 5 hours 59 minutes, initial encounter |
| S069X3D | | ICD10 |  | Unspecified intracranial injury with loss of consciousness of 1 hour to 5 hours 59 minutes, subsequent encounter |
| S069X3S | | ICD10 |  | Unspecified intracranial injury with loss of consciousness of 1 hour to 5 hours 59 minutes, sequela |
| S069X4A | | ICD10 |  | Unspecified intracranial injury with loss of consciousness of 6 hours to 24 hours, initial encounter |
| S069X4D | | ICD10 |  | Unspecified intracranial injury with loss of consciousness of 6 hours to 24 hours, subsequent encounter |
| S069X4S | | ICD10 |  | Unspecified intracranial injury with loss of consciousness of 6 hours to 24 hours, sequela |
| S069X5A | | ICD10 |  | Unspecified intracranial injury with loss of consciousness greater than 24 hours with return to pre-existing conscious level, initial encounter |
| S069X5D | | ICD10 |  | Unspecified intracranial injury with loss of consciousness greater than 24 hours with return to pre-existing conscious level, subsequent encounter |
| S069X5S | | ICD10 |  | Unspecified intracranial injury with loss of consciousness greater than 24 hours with return to pre-existing conscious level, sequela |
| S069X6A | | ICD10 |  | Unspecified intracranial injury with loss of consciousness greater than 24 hours without return to pre-existing conscious level with patient surviving, initial encounter |
| S069X6D | | ICD10 |  | Unspecified intracranial injury with loss of consciousness greater than 24 hours without return to pre-existing conscious level with patient surviving, subsequent encounter |
| S069X6S | | ICD10 |  | Unspecified intracranial injury with loss of consciousness greater than 24 hours without return to pre-existing conscious level with patient surviving, sequela |
| S069X7A | | ICD10 |  | Unspecified intracranial injury with loss of consciousness of any duration with death due to brain injury prior to regaining consciousness, initial encounter |
| S069X8A | | ICD10 |  | Unspecified intracranial injury with loss of consciousness of any duration with death due to other cause prior to regaining consciousness, initial encounter |
| S069X9A | | ICD10 |  | Unspecified intracranial injury with loss of consciousness of unspecified duration, initial encounter |
| S069X9D | | ICD10 |  | Unspecified intracranial injury with loss of consciousness of unspecified duration, subsequent encounter |
| S069X9S | | ICD10 |  | Unspecified intracranial injury with loss of consciousness of unspecified duration, sequela |
|  | |  |  |  |
