# Supplementary material for: Relationship between Nonhepatic Serum Ammonia Levels and Sepsis-Associated Encephalopathy: A Retrospective Cohort Study
Source: Emerg Med Int. 2023 Oct 12;2023:6676033. doi: 10.1155/2023/6676033 (PMC10590267; doi:10.1155/2023/6676033)
Supplement: Supplementary Materials — 1: exclude patients with trauma of the skull from the MIMIC IV database according to ICD codes. Supplementary materials 2: exclude patients with intracerebral hemorrhage, cerebral embolism, and ischemic stroke disease from the MIMIC IV database according to ICD codes. Supplementary materials 3: exclude patients with meningitis and encephalitis disease from the MIMIC IV database according to ICD codes. Supplementary materials 4: exclude patients with epilepsy disease from the MIMIC IV database according to ICD codes. Supplementary materials 5: exclude patients with other cerebrovascular disease from the MIMIC IV database according to ICD codes. Supplementary materials 6: exclude patients with mental disorders and neurological disease from the MIMIC IV database according to ICD codes. Supplementary materials 7: exclude patients with alcoholic intoxication or drug abuse from the MIMIC IV database according to ICD codes. Supplementary materials 8: exclude patients with metabolic encephalopathy, hepatic encephalopathy, hypertensive encephalopathy, diabetes with coma, disorders of urea cycle, hypernatremia, and Wernicke's encephalopathy from the MIMIC IV database according to ICD codes. Supplementary materials 9: exclude patients with acute and chronic liver disease. Supplementary materials 10: hypertension disease and ICD codes. Supplementary materials 11: diabetes disease and ICD codes. Supplementary materials 12: lung disease and ICD codes. Supplementary materials 13: cardiovascular diseases and ICD codes. Supplementary materials 14: renal disease from the MIMIC IV database according to ICD codes. Supplementary materials 15: the standardized mean differences of the original cohort were compared with those of the IPW cohorts in sepsis patients. SMD: standardized mean differences. [file 6676033.f1.zip › Supplementary materials.10.docx]

| **Supplementary materials.10** Hypertension disease and ICD-codes | | |  |  |  |  |  |  |  |  |  |
| --- | --- | --- | --- | --- | --- | --- | --- | --- | --- | --- | --- |
| Disease | ICD-Code | Description |  |  |  |  |  |  |  |  |  |
| Hypertension |  |  |  |  |  |  |  |  |  |  |  |
|  | 64222 | Other pre-existing hypertension, complicating pregnancy, childbirth, and the puerperium, delivered, with mention of postpartum complication |  |  |  |  |  |  |  |  |  |
|  | 64200 | Benign essential hypertension complicating pregnancy, childbirth, and the puerperium, unspecified as to episode of care or not applicable |  |  |  |  |  |  |  |  |  |
|  | 64201 | Benign essential hypertension complicating pregnancy, childbirth, and the puerperium, delivered, with or without mention of antepartum condition |  |  |  |  |  |  |  |  |  |
|  | 64202 | Benign essential hypertension, complicating pregnancy, childbirth, and the puerperium, delivered, with mention of postpartum complication |  |  |  |  |  |  |  |  |  |
|  | 64203 | Benign essential hypertension complicating pregnancy, childbirth, and the puerperium, antepartum condition or complication |  |  |  |  |  |  |  |  |  |
|  | 64204 | Benign essential hypertension complicating pregnancy, childbirth, and the puerperium, postpartum condition or complication |  |  |  |  |  |  |  |  |  |
|  | 64210 | Hypertension secondary to renal disease, complicating pregnancy, childbirth, and the puerperium, unspecified as to episode of care or not applicable |  |  |  |  |  |  |  |  |  |
|  | 64211 | Hypertension secondary to renal disease, complicating pregnancy, childbirth, and the puerperium, delivered, with or without mention of antepartum condition |  |  |  |  |  |  |  |  |  |
|  | 64212 | Hypertension secondary to renal disease, complicating pregnancy, childbirth, and the puerperium, delivered, with mention of postpartum complication |  |  |  |  |  |  |  |  |  |
|  | 64213 | Hypertension secondary to renal disease, complicating pregnancy, childbirth, and the puerperium, antepartum condition or complication |  |  |  |  |  |  |  |  |  |
|  | 64214 | Hypertension secondary to renal disease, complicating pregnancy, childbirth, and the puerperium, postpartum condition or complication |  |  |  |  |  |  |  |  |  |
|  | 64220 | Other pre-existing hypertension complicating pregnancy, childbirth, and the puerperium, unspecified as to episode of care or not applicable |  |  |  |  |  |  |  |  |  |
|  | 64221 | Other pre-existing hypertension, complicating pregnancy, childbirth, and the puerperium, delivered, with or without mention of antepartum condition |  |  |  |  |  |  |  |  |  |
|  | 64223 | Other pre-existing hypertension, complicating pregnancy, childbirth, and the puerperium, antepartum condition or complication |  |  |  |  |  |  |  |  |  |
|  | 64224 | Other pre-existing hypertension,complicating pregnancy, childbirth, and the puerperium, , postpartum condition or complication |  |  |  |  |  |  |  |  |  |
|  | 64270 | Pre-eclampsia or eclampsia superimposed on pre-existing hypertension, unspecified as to episode of care or not applicable |  |  |  |  |  |  |  |  |  |
|  | 64271 | Pre-eclampsia or eclampsia superimposed on pre-existing hypertension, delivered, with or without mention of antepartum condition |  |  |  |  |  |  |  |  |  |
|  | 64272 | Pre-eclampsia or eclampsia superimposed on pre-existing hypertension, delivered, with mention of postpartum complication |  |  |  |  |  |  |  |  |  |
|  | 64273 | Pre-eclampsia or eclampsia superimposed on pre-existing hypertension, antepartum condition or complication |  |  |  |  |  |  |  |  |  |
|  | 64274 | Pre-eclampsia or eclampsia superimposed on pre-existing hypertension, postpartum condition or complication |  |  |  |  |  |  |  |  |  |
|  | 64290 | Unspecified hypertension complicating pregnancy, childbirth, or the puerperium, unspecified as to episode of care or not applicable |  |  |  |  |  |  |  |  |  |
|  | 7600 | Maternal hypertensive disorders affecting fetus or newborn |  |  |  |  |  |  |  |  |  |
|  | 9726 | Poisoning by other antihypertensive agents |  |  |  |  |  |  |  |  |  |
|  | 99791 | Complications affecting other specified body systems, not elsewhere classified, hypertension |  |  |  |  |  |  |  |  |  |
|  | E9426 | Other antihypertensive agents causing adverse effects in therapeutic use |  |  |  |  |  |  |  |  |  |
|  | 36042 | Blind hypertensive eye |  |  |  |  |  |  |  |  |  |
|  | 36211 | Hypertensive retinopathy |  |  |  |  |  |  |  |  |  |
|  | 4010 | Malignant essential hypertension |  |  |  |  |  |  |  |  |  |
|  | 4011 | Benign essential hypertension |  |  |  |  |  |  |  |  |  |
|  | 4019 | Unspecified essential hypertension |  |  |  |  |  |  |  |  |  |
|  | 40200 | Malignant hypertensive heart disease without heart failure |  |  |  |  |  |  |  |  |  |
|  | 40201 | Malignant hypertensive heart disease with heart failure |  |  |  |  |  |  |  |  |  |
|  | 40210 | Benign hypertensive heart disease without heart failure |  |  |  |  |  |  |  |  |  |
|  | 40211 | Benign hypertensive heart disease with heart failure |  |  |  |  |  |  |  |  |  |
|  | 40290 | Unspecified hypertensive heart disease without heart failure |  |  |  |  |  |  |  |  |  |
|  | 40291 | Unspecified hypertensive heart disease with heart failure |  |  |  |  |  |  |  |  |  |
|  | 40300 | Hypertensive chronic kidney disease, malignant, with chronic kidney disease stage I through stage IV, or unspecified |  |  |  |  |  |  |  |  |  |
|  | 40301 | Hypertensive chronic kidney disease, malignant, with chronic kidney disease stage V or end stage renal disease |  |  |  |  |  |  |  |  |  |
|  | 40310 | Hypertensive chronic kidney disease, benign, with chronic kidney disease stage I through stage IV, or unspecified |  |  |  |  |  |  |  |  |  |
|  | 40311 | Hypertensive chronic kidney disease, benign, with chronic kidney disease stage V or end stage renal disease |  |  |  |  |  |  |  |  |  |
|  | 40390 | Hypertensive chronic kidney disease, unspecified, with chronic kidney disease stage I through stage IV, or unspecified |  |  |  |  |  |  |  |  |  |
|  | 40391 | Hypertensive chronic kidney disease, unspecified, with chronic kidney disease stage V or end stage renal disease |  |  |  |  |  |  |  |  |  |
|  | 40400 | Hypertensive heart and chronic kidney disease, malignant, without heart failure and with chronic kidney disease stage I through stage IV, or unspecified |  |  |  |  |  |  |  |  |  |
|  | 40401 | Hypertensive heart and chronic kidney disease, malignant, with heart failure and with chronic kidney disease stage I through stage IV, or unspecified |  |  |  |  |  |  |  |  |  |
|  | 40402 | Hypertensive heart and chronic kidney disease, malignant, without heart failure and with chronic kidney disease stage V or end stage renal disease |  |  |  |  |  |  |  |  |  |
|  | 40403 | Hypertensive heart and chronic kidney disease, malignant, with heart failure and with chronic kidney disease stage V or end stage renal disease |  |  |  |  |  |  |  |  |  |
|  | 40410 | Hypertensive heart and chronic kidney disease, benign, without heart failure and with chronic kidney disease stage I through stage IV, or unspecified |  |  |  |  |  |  |  |  |  |
|  | 40411 | Hypertensive heart and chronic kidney disease, benign, with heart failure and with chronic kidney disease stage I through stage IV, or unspecified |  |  |  |  |  |  |  |  |  |
|  | 40412 | Hypertensive heart and chronic kidney disease, benign, without heart failure and with chronic kidney disease stage V or end stage renal disease |  |  |  |  |  |  |  |  |  |
|  | 40413 | Hypertensive heart and chronic kidney disease, benign, with heart failure and chronic kidney disease stage V or end stage renal disease |  |  |  |  |  |  |  |  |  |
|  | 40490 | Hypertensive heart and chronic kidney disease, unspecified, without heart failure and with chronic kidney disease stage I through stage IV, or unspecified |  |  |  |  |  |  |  |  |  |
|  | 40491 | Hypertensive heart and chronic kidney disease, unspecified, with heart failure and with chronic kidney disease stage I through stage IV, or unspecified |  |  |  |  |  |  |  |  |  |
|  | 40492 | Hypertensive heart and chronic kidney disease, unspecified, without heart failure and with chronic kidney disease stage V or end stage renal disease |  |  |  |  |  |  |  |  |  |
|  | 40493 | Hypertensive heart and chronic kidney disease, unspecified, with heart failure and chronic kidney disease stage V or end stage renal disease |  |  |  |  |  |  |  |  |  |
|  | 40501 | Malignant renovascular hypertension |  |  |  |  |  |  |  |  |  |
|  | 40509 | Other malignant secondary hypertension |  |  |  |  |  |  |  |  |  |
|  | 40511 | Benign renovascular hypertension |  |  |  |  |  |  |  |  |  |
|  | 40519 | Other benign secondary hypertension |  |  |  |  |  |  |  |  |  |
|  | 40599 | Other unspecified secondary hypertension |  |  |  |  |  |  |  |  |  |
|  | 40591 | Unspecified renovascular hypertension |  |  |  |  |  |  |  |  |  |
|  | 45930 | Chronic venous hypertension without complications |  |  |  |  |  |  |  |  |  |
|  | 45931 | Chronic venous hypertension with ulcer |  |  |  |  |  |  |  |  |  |
|  | 45932 | Chronic venous hypertension with inflammation |  |  |  |  |  |  |  |  |  |
|  | 45933 | Chronic venous hypertension with ulcer and inflammation |  |  |  |  |  |  |  |  |  |
|  | 45939 | Chronic venous hypertension with other complication |  |  |  |  |  |  |  |  |  |
|  | H40051 | Ocular hypertension, right eye |  |  |  |  |  |  |  |  |  |
|  | H40052 | Ocular hypertension, left eye |  |  |  |  |  |  |  |  |  |
|  | H40053 | Ocular hypertension, bilateral |  |  |  |  |  |  |  |  |  |
|  | H40059 | Ocular hypertension, unspecified eye |  |  |  |  |  |  |  |  |  |
|  | I10 | Essential (primary) hypertension |  |  |  |  |  |  |  |  |  |
|  | I110 | Hypertensive heart disease with heart failure |  |  |  |  |  |  |  |  |  |
|  | I119 | Hypertensive heart disease without heart failure |  |  |  |  |  |  |  |  |  |
|  | I120 | Hypertensive chronic kidney disease with stage 5 chronic kidney disease or end stage renal disease |  |  |  |  |  |  |  |  |  |
|  | I129 | Hypertensive chronic kidney disease with stage 1 through stage 4 chronic kidney disease, or unspecified chronic kidney disease |  |  |  |  |  |  |  |  |  |
|  | I130 | Hypertensive heart and chronic kidney disease with heart failure and stage 1 through stage 4 chronic kidney disease, or unspecified chronic kidney disease |  |  |  |  |  |  |  |  |  |
|  | I1310 | Hypertensive heart and chronic kidney disease without heart failure, with stage 1 through stage 4 chronic kidney disease, or unspecified chronic kidney disease |  |  |  |  |  |  |  |  |  |
|  | I1311 | Hypertensive heart and chronic kidney disease without heart failure, with stage 5 chronic kidney disease, or end stage renal disease |  |  |  |  |  |  |  |  |  |
|  | I132 | Hypertensive heart and chronic kidney disease with heart failure and with stage 5 chronic kidney disease, or end stage renal disease |  |  |  |  |  |  |  |  |  |
|  | I150 | Renovascular hypertension |  |  |  |  |  |  |  |  |  |
|  | I151 | Hypertension secondary to other renal disorders |  |  |  |  |  |  |  |  |  |
|  | I152 | Hypertension secondary to endocrine disorders |  |  |  |  |  |  |  |  |  |
|  | I158 | Other secondary hypertension |  |  |  |  |  |  |  |  |  |
|  | I159 | Secondary hypertension, unspecified |  |  |  |  |  |  |  |  |  |
|  | I160 | Hypertensive urgency |  |  |  |  |  |  |  |  |  |
|  | I161 | Hypertensive emergency |  |  |  |  |  |  |  |  |  |
|  | I169 | Hypertensive crisis, unspecified |  |  |  |  |  |  |  |  |  |
|  | I270 | Primary pulmonary hypertension |  |  |  |  |  |  |  |  |  |
|  | I2720 | Pulmonary hypertension, unspecified |  |  |  |  |  |  |  |  |  |
|  | I2721 | Secondary pulmonary arterial hypertension |  |  |  |  |  |  |  |  |  |
|  | I2722 | Pulmonary hypertension due to left heart disease |  |  |  |  |  |  |  |  |  |
|  | I2723 | Pulmonary hypertension due to lung diseases and hypoxia |  |  |  |  |  |  |  |  |  |
|  | I2724 | Chronic thromboembolic pulmonary hypertension |  |  |  |  |  |  |  |  |  |
|  | I2729 | Other secondary pulmonary hypertension |  |  |  |  |  |  |  |  |  |
|  | I87301 | Chronic venous hypertension (idiopathic) without complications of right lower extremity |  |  |  |  |  |  |  |  |  |
|  | I87302 | Chronic venous hypertension (idiopathic) without complications of left lower extremity |  |  |  |  |  |  |  |  |  |
|  | I87303 | Chronic venous hypertension (idiopathic) without complications of bilateral lower extremity |  |  |  |  |  |  |  |  |  |
|  | I87309 | Chronic venous hypertension (idiopathic) without complications of unspecified lower extremity |  |  |  |  |  |  |  |  |  |
|  | I87311 | Chronic venous hypertension (idiopathic) with ulcer of right lower extremity |  |  |  |  |  |  |  |  |  |
|  | I87312 | Chronic venous hypertension (idiopathic) with ulcer of left lower extremity |  |  |  |  |  |  |  |  |  |
|  | I87313 | Chronic venous hypertension (idiopathic) with ulcer of bilateral lower extremity |  |  |  |  |  |  |  |  |  |
|  | I87319 | Chronic venous hypertension (idiopathic) with ulcer of unspecified lower extremity |  |  |  |  |  |  |  |  |  |
|  | I87321 | Chronic venous hypertension (idiopathic) with inflammation of right lower extremity |  |  |  |  |  |  |  |  |  |
|  | I87322 | Chronic venous hypertension (idiopathic) with inflammation of left lower extremity |  |  |  |  |  |  |  |  |  |
|  | I87323 | Chronic venous hypertension (idiopathic) with inflammation of bilateral lower extremity |  |  |  |  |  |  |  |  |  |
|  | I87329 | Chronic venous hypertension (idiopathic) with inflammation of unspecified lower extremity |  |  |  |  |  |  |  |  |  |
|  | I87331 | Chronic venous hypertension (idiopathic) with ulcer and inflammation of right lower extremity |  |  |  |  |  |  |  |  |  |
|  | I87332 | Chronic venous hypertension (idiopathic) with ulcer and inflammation of left lower extremity |  |  |  |  |  |  |  |  |  |
|  | I87333 | Chronic venous hypertension (idiopathic) with ulcer and inflammation of bilateral lower extremity |  |  |  |  |  |  |  |  |  |
|  | I87339 | Chronic venous hypertension (idiopathic) with ulcer and inflammation of unspecified lower extremity |  |  |  |  |  |  |  |  |  |
|  | I87391 | Chronic venous hypertension (idiopathic) with other complications of right lower extremity |  |  |  |  |  |  |  |  |  |
|  | I87392 | Chronic venous hypertension (idiopathic) with other complications of left lower extremity |  |  |  |  |  |  |  |  |  |
|  | I87393 | Chronic venous hypertension (idiopathic) with other complications of bilateral lower extremity |  |  |  |  |  |  |  |  |  |
|  | I87399 | Chronic venous hypertension (idiopathic) with other complications of unspecified lower extremity |  |  |  |  |  |  |  |  |  |
|  | I973 | Postprocedural hypertension |  |  |  |  |  |  |  |  |  |
|  | K766 | Portal hypertension |  |  |  |  |  |  |  |  |  |
|  | K767 | Hepatorenal syndrome |  |  |  |  |  |  |  |  |  |
|  | O10011 | Pre-existing essential hypertension complicating pregnancy, first trimester |  |  |  |  |  |  |  |  |  |
|  | O10012 | Pre-existing essential hypertension complicating pregnancy, second trimester |  |  |  |  |  |  |  |  |  |
|  | O10013 | Pre-existing essential hypertension complicating pregnancy, third trimester |  |  |  |  |  |  |  |  |  |
|  | O10019 | Pre-existing essential hypertension complicating pregnancy, unspecified trimester |  |  |  |  |  |  |  |  |  |
|  | O1002 | Pre-existing essential hypertension complicating childbirth |  |  |  |  |  |  |  |  |  |
|  | O1003 | Pre-existing essential hypertension complicating the puerperium |  |  |  |  |  |  |  |  |  |
|  | O10111 | Pre-existing hypertensive heart disease complicating pregnancy, first trimester |  |  |  |  |  |  |  |  |  |
|  | O10112 | Pre-existing hypertensive heart disease complicating pregnancy, second trimester |  |  |  |  |  |  |  |  |  |
|  | O10113 | Pre-existing hypertensive heart disease complicating pregnancy, third trimester |  |  |  |  |  |  |  |  |  |
|  | O10119 | Pre-existing hypertensive heart disease complicating pregnancy, unspecified trimester |  |  |  |  |  |  |  |  |  |
|  | O1012 | Pre-existing hypertensive heart disease complicating childbirth |  |  |  |  |  |  |  |  |  |
|  | O1013 | Pre-existing hypertensive heart disease complicating the puerperium |  |  |  |  |  |  |  |  |  |
|  | O10211 | Pre-existing hypertensive chronic kidney disease complicating pregnancy, first trimester |  |  |  |  |  |  |  |  |  |
|  | O10212 | Pre-existing hypertensive chronic kidney disease complicating pregnancy, second trimester |  |  |  |  |  |  |  |  |  |
|  | O10213 | Pre-existing hypertensive chronic kidney disease complicating pregnancy, third trimester |  |  |  |  |  |  |  |  |  |
|  | O10219 | Pre-existing hypertensive chronic kidney disease complicating pregnancy, unspecified trimester |  |  |  |  |  |  |  |  |  |
|  | O1022 | Pre-existing hypertensive chronic kidney disease complicating childbirth |  |  |  |  |  |  |  |  |  |
|  | O1023 | Pre-existing hypertensive chronic kidney disease complicating the puerperium |  |  |  |  |  |  |  |  |  |
|  | O10311 | Pre-existing hypertensive heart and chronic kidney disease complicating pregnancy, first trimester |  |  |  |  |  |  |  |  |  |
|  | O10312 | Pre-existing hypertensive heart and chronic kidney disease complicating pregnancy, second trimester |  |  |  |  |  |  |  |  |  |
|  | O10313 | Pre-existing hypertensive heart and chronic kidney disease complicating pregnancy, third trimester |  |  |  |  |  |  |  |  |  |
|  | O10319 | Pre-existing hypertensive heart and chronic kidney disease complicating pregnancy, unspecified trimester |  |  |  |  |  |  |  |  |  |
|  | O1032 | Pre-existing hypertensive heart and chronic kidney disease complicating childbirth |  |  |  |  |  |  |  |  |  |
|  | O1033 | Pre-existing hypertensive heart and chronic kidney disease complicating the puerperium |  |  |  |  |  |  |  |  |  |
|  | O10411 | Pre-existing secondary hypertension complicating pregnancy, first trimester |  |  |  |  |  |  |  |  |  |
|  | O10412 | Pre-existing secondary hypertension complicating pregnancy, second trimester |  |  |  |  |  |  |  |  |  |
|  | O10413 | Pre-existing secondary hypertension complicating pregnancy, third trimester |  |  |  |  |  |  |  |  |  |
|  | O10419 | Pre-existing secondary hypertension complicating pregnancy, unspecified trimester |  |  |  |  |  |  |  |  |  |
|  | O1042 | Pre-existing secondary hypertension complicating childbirth |  |  |  |  |  |  |  |  |  |
|  | O1043 | Pre-existing secondary hypertension complicating the puerperium |  |  |  |  |  |  |  |  |  |
|  | O10911 | Unspecified pre-existing hypertension complicating pregnancy, first trimester |  |  |  |  |  |  |  |  |  |
|  | O10912 | Unspecified pre-existing hypertension complicating pregnancy, second trimester |  |  |  |  |  |  |  |  |  |
|  | O10913 | Unspecified pre-existing hypertension complicating pregnancy, third trimester |  |  |  |  |  |  |  |  |  |
|  | O10919 | Unspecified pre-existing hypertension complicating pregnancy, unspecified trimester |  |  |  |  |  |  |  |  |  |
|  | O1092 | Unspecified pre-existing hypertension complicating childbirth |  |  |  |  |  |  |  |  |  |
|  | O1093 | Unspecified pre-existing hypertension complicating the puerperium |  |  |  |  |  |  |  |  |  |
|  | O111 | Pre-existing hypertension with pre-eclampsia, first trimester |  |  |  |  |  |  |  |  |  |
|  | O112 | Pre-existing hypertension with pre-eclampsia, second trimester |  |  |  |  |  |  |  |  |  |
|  | O113 | Pre-existing hypertension with pre-eclampsia, third trimester |  |  |  |  |  |  |  |  |  |
|  | O114 | Pre-existing hypertension with pre-eclampsia, complicating childbirth |  |  |  |  |  |  |  |  |  |
|  | O115 | Pre-existing hypertension with pre-eclampsia, complicating the puerperium |  |  |  |  |  |  |  |  |  |
|  | O119 | Pre-existing hypertension with pre-eclampsia, unspecified trimester |  |  |  |  |  |  |  |  |  |
|  | O131 | Gestational [pregnancy-induced] hypertension without significant proteinuria, first trimester |  |  |  |  |  |  |  |  |  |
|  | O132 | Gestational [pregnancy-induced] hypertension without significant proteinuria, second trimester |  |  |  |  |  |  |  |  |  |
|  | O133 | Gestational [pregnancy-induced] hypertension without significant proteinuria, third trimester |  |  |  |  |  |  |  |  |  |
|  | O134 | Gestational [pregnancy-induced] hypertension without significant proteinuria, complicating childbirth |  |  |  |  |  |  |  |  |  |
|  | O135 | Gestational [pregnancy-induced] hypertension without significant proteinuria, complicating the puerperium |  |  |  |  |  |  |  |  |  |
|  | O139 | Gestational [pregnancy-induced] hypertension without significant proteinuria, unspecified trimester |  |  |  |  |  |  |  |  |  |
|  | O161 | Unspecified maternal hypertension, first trimester |  |  |  |  |  |  |  |  |  |
|  | O162 | Unspecified maternal hypertension, second trimester |  |  |  |  |  |  |  |  |  |
|  | O163 | Unspecified maternal hypertension, third trimester |  |  |  |  |  |  |  |  |  |
|  | O164 | Unspecified maternal hypertension, complicating childbirth |  |  |  |  |  |  |  |  |  |
|  | O165 | Unspecified maternal hypertension, complicating the puerperium |  |  |  |  |  |  |  |  |  |
|  | O169 | Unspecified maternal hypertension, unspecified trimester |  |  |  |  |  |  |  |  |  |
|  | P292 | Neonatal hypertension |  |  |  |  |  |  |  |  |  |
|  | P2930 | Pulmonary hypertension of newborn |  |  |  |  |  |  |  |  |  |
|  | R030 | Elevated blood-pressure reading, without diagnosis of hypertension |  |  |  |  |  |  |  |  |  |
|  | V811 | Screening for hypertension |  |  |  |  |  |  |  |  |  |
