# Supplementary material for: Relationship between Nonhepatic Serum Ammonia Levels and Sepsis-Associated Encephalopathy: A Retrospective Cohort Study
Source: Emerg Med Int. 2023 Oct 12;2023:6676033. doi: 10.1155/2023/6676033 (PMC10590267; doi:10.1155/2023/6676033)
Supplement: Supplementary Materials — 1: exclude patients with trauma of the skull from the MIMIC IV database according to ICD codes. Supplementary materials 2: exclude patients with intracerebral hemorrhage, cerebral embolism, and ischemic stroke disease from the MIMIC IV database according to ICD codes. Supplementary materials 3: exclude patients with meningitis and encephalitis disease from the MIMIC IV database according to ICD codes. Supplementary materials 4: exclude patients with epilepsy disease from the MIMIC IV database according to ICD codes. Supplementary materials 5: exclude patients with other cerebrovascular disease from the MIMIC IV database according to ICD codes. Supplementary materials 6: exclude patients with mental disorders and neurological disease from the MIMIC IV database according to ICD codes. Supplementary materials 7: exclude patients with alcoholic intoxication or drug abuse from the MIMIC IV database according to ICD codes. Supplementary materials 8: exclude patients with metabolic encephalopathy, hepatic encephalopathy, hypertensive encephalopathy, diabetes with coma, disorders of urea cycle, hypernatremia, and Wernicke's encephalopathy from the MIMIC IV database according to ICD codes. Supplementary materials 9: exclude patients with acute and chronic liver disease. Supplementary materials 10: hypertension disease and ICD codes. Supplementary materials 11: diabetes disease and ICD codes. Supplementary materials 12: lung disease and ICD codes. Supplementary materials 13: cardiovascular diseases and ICD codes. Supplementary materials 14: renal disease from the MIMIC IV database according to ICD codes. Supplementary materials 15: the standardized mean differences of the original cohort were compared with those of the IPW cohorts in sepsis patients. SMD: standardized mean differences. [file 6676033.f1.zip › Supplementary materials.12.docx]

| **Supplementary materials 12** Lung disease and ICD-codes | |  |  |  |  |  |  |  |  |  |  |
| --- | --- | --- | --- | --- | --- | --- | --- | --- | --- | --- | --- |
| ICD-Code | Description |  |  |  |  |  |  |  |  |  |  |
|  |  | O1013 |  |  |  |  |  |  |  |  |  |
| 322 | Salmonella pneumonia |  |  |  |  |  |  |  |  |  |  |
| 1160 | Tuberculous pneumonia [any form], unspecified |  |  |  |  |  |  |  |  |  |  |
| 1161 | Tuberculous pneumonia [any form], bacteriological or histological examination not done |  |  |  |  |  |  |  |  |  |  |
| 1162 | Tuberculous pneumonia [any form], bacteriological or histological examination unknown (at present) |  |  |  |  |  |  |  |  |  |  |
| 1163 | Tuberculous pneumonia [any form], tubercle bacilli found (in sputum) by microscopy |  |  |  |  |  |  |  |  |  |  |
| 1164 | Tuberculous pneumonia [any form], tubercle bacilli not found (in sputum) by microscopy, but found by bacterial culture |  |  |  |  |  |  |  |  |  |  |
| 1165 | Tuberculous pneumonia [any form], tubercle bacilli not found by bacteriological examination, but tuberculosis confirmed histologically |  |  |  |  |  |  |  |  |  |  |
| 1166 | Tuberculous pneumonia [any form], tubercle bacilli not found by bacteriological or histological examination, but tuberculosis confirmed by other methods [inoculation of animals] |  |  |  |  |  |  |  |  |  |  |
| 551 | Postmeasles pneumonia |  |  |  |  |  |  |  |  |  |  |
| 730 | Ornithosis with pneumonia |  |  |  |  |  |  |  |  |  |  |
| 11505 | Infection by Histoplasma capsulatum, pneumonia |  |  |  |  |  |  |  |  |  |  |
| 11515 | Infection by Histoplasma duboisii, pneumonia |  |  |  |  |  |  |  |  |  |  |
| 11595 | Histoplasmosis, unspecified, pneumonia |  |  |  |  |  |  |  |  |  |  |
| 4800 | Pneumonia due to adenovirus |  |  |  |  |  |  |  |  |  |  |
| 4801 | Pneumonia due to respiratory syncytial virus |  |  |  |  |  |  |  |  |  |  |
| 4802 | Pneumonia due to parainfluenza virus |  |  |  |  |  |  |  |  |  |  |
| 4803 | Pneumonia due to SARS-associated coronavirus |  |  |  |  |  |  |  |  |  |  |
| 4808 | Pneumonia due to other virus not elsewhere classified |  |  |  |  |  |  |  |  |  |  |
| 4809 | Viral pneumonia, unspecified |  |  |  |  |  |  |  |  |  |  |
| 481 | Pneumococcal pneumonia [Streptococcus pneumoniae pneumonia] |  |  |  |  |  |  |  |  |  |  |
| 4820 | Pneumonia due to Klebsiella pneumoniae |  |  |  |  |  |  |  |  |  |  |
| 4821 | Pneumonia due to Pseudomonas |  |  |  |  |  |  |  |  |  |  |
| 4822 | Pneumonia due to Hemophilus influenzae [H. influenzae] |  |  |  |  |  |  |  |  |  |  |
| 48230 | Pneumonia due to Streptococcus, unspecified |  |  |  |  |  |  |  |  |  |  |
| 48231 | Pneumonia due to Streptococcus, group A |  |  |  |  |  |  |  |  |  |  |
| 48232 | Pneumonia due to Streptococcus, group B |  |  |  |  |  |  |  |  |  |  |
| 48239 | Pneumonia due to other Streptococcus |  |  |  |  |  |  |  |  |  |  |
| 48240 | Pneumonia due to Staphylococcus, unspecified |  |  |  |  |  |  |  |  |  |  |
| 48241 | Methicillin susceptible pneumonia due to Staphylococcus aureus |  |  |  |  |  |  |  |  |  |  |
| 48242 | Methicillin resistant pneumonia due to Staphylococcus aureus |  |  |  |  |  |  |  |  |  |  |
| 48249 | Other Staphylococcus pneumonia |  |  |  |  |  |  |  |  |  |  |
| 48281 | Pneumonia due to anaerobes |  |  |  |  |  |  |  |  |  |  |
| 48282 | Pneumonia due to escherichia coli [E. coli] |  |  |  |  |  |  |  |  |  |  |
| 48283 | Pneumonia due to other gram-negative bacteria |  |  |  |  |  |  |  |  |  |  |
| 48284 | Pneumonia due to Legionnaires' disease |  |  |  |  |  |  |  |  |  |  |
| 48289 | Pneumonia due to other specified bacteria |  |  |  |  |  |  |  |  |  |  |
| 4829 | Bacterial pneumonia, unspecified |  |  |  |  |  |  |  |  |  |  |
| 4830 | Pneumonia due to mycoplasma pneumoniae |  |  |  |  |  |  |  |  |  |  |
| 4831 | Pneumonia due to chlamydia |  |  |  |  |  |  |  |  |  |  |
| 4838 | Pneumonia due to other specified organism |  |  |  |  |  |  |  |  |  |  |
| 4841 | Pneumonia in cytomegalic inclusion disease |  |  |  |  |  |  |  |  |  |  |
| 4843 | Pneumonia in whooping cough |  |  |  |  |  |  |  |  |  |  |
| 4845 | Pneumonia in anthrax |  |  |  |  |  |  |  |  |  |  |
| 4846 | Pneumonia in aspergillosis |  |  |  |  |  |  |  |  |  |  |
| 4847 | Pneumonia in other systemic mycoses |  |  |  |  |  |  |  |  |  |  |
| 4848 | Pneumonia in other infectious diseases classified elsewhere |  |  |  |  |  |  |  |  |  |  |
| 485 | Bronchopneumonia, organism unspecified |  |  |  |  |  |  |  |  |  |  |
| 486 | Pneumonia, organism unspecified |  |  |  |  |  |  |  |  |  |  |
| 4870 | Influenza with pneumonia |  |  |  |  |  |  |  |  |  |  |
| 51630 | Idiopathic interstitial pneumonia, not otherwise specified |  |  |  |  |  |  |  |  |  |  |
| 51635 | Idiopathic lymphoid interstitial pneumonia |  |  |  |  |  |  |  |  |  |  |
| 51636 | Cryptogenic organizing pneumonia |  |  |  |  |  |  |  |  |  |  |
| 51637 | Desquamative interstitial pneumonia |  |  |  |  |  |  |  |  |  |  |
| 5171 | Rheumatic pneumonia |  |  |  |  |  |  |  |  |  |  |
| 7700 | Congenital pneumonia |  |  |  |  |  |  |  |  |  |  |
| 99731 | Ventilator associated pneumonia |  |  |  |  |  |  |  |  |  |  |
| 99732 | Postprocedural aspiration pneumonia |  |  |  |  |  |  |  |  |  |  |
| A0222 | Salmonella pneumonia |  |  |  |  |  |  |  |  |  |  |
| A3700 | Whooping cough due to Bordetella pertussis without pneumonia |  |  |  |  |  |  |  |  |  |  |
| A3701 | Whooping cough due to Bordetella pertussis with pneumonia |  |  |  |  |  |  |  |  |  |  |
| A3710 | Whooping cough due to Bordetella parapertussis without pneumonia |  |  |  |  |  |  |  |  |  |  |
| A3711 | Whooping cough due to Bordetella parapertussis with pneumonia |  |  |  |  |  |  |  |  |  |  |
| A3780 | Whooping cough due to other Bordetella species without pneumonia |  |  |  |  |  |  |  |  |  |  |
| A3781 | Whooping cough due to other Bordetella species with pneumonia |  |  |  |  |  |  |  |  |  |  |
| A3790 | Whooping cough, unspecified species without pneumonia |  |  |  |  |  |  |  |  |  |  |
| A3791 | Whooping cough, unspecified species with pneumonia |  |  |  |  |  |  |  |  |  |  |
| A5004 | Early congenital syphilitic pneumonia |  |  |  |  |  |  |  |  |  |  |
| A5484 | Gonococcal pneumonia |  |  |  |  |  |  |  |  |  |  |
| B012 | Varicella pneumonia |  |  |  |  |  |  |  |  |  |  |
| B0681 | Rubella pneumonia |  |  |  |  |  |  |  |  |  |  |
| J95851 | Ventilator associated pneumonia |  |  |  |  |  |  |  |  |  |  |
| P230 | Congenital pneumonia due to viral agent |  |  |  |  |  |  |  |  |  |  |
| P231 | Congenital pneumonia due to Chlamydia |  |  |  |  |  |  |  |  |  |  |
| P232 | Congenital pneumonia due to staphylococcus |  |  |  |  |  |  |  |  |  |  |
| P233 | Congenital pneumonia due to streptococcus, group B |  |  |  |  |  |  |  |  |  |  |
| P234 | Congenital pneumonia due to Escherichia coli |  |  |  |  |  |  |  |  |  |  |
| P235 | Congenital pneumonia due to Pseudomonas |  |  |  |  |  |  |  |  |  |  |
| P236 | Congenital pneumonia due to other bacterial agents |  |  |  |  |  |  |  |  |  |  |
| P238 | Congenital pneumonia due to other organisms |  |  |  |  |  |  |  |  |  |  |
| V0382 | Other specified vaccinations against streptococcus pneumoniae [pneumococcus] |  |  |  |  |  |  |  |  |  |  |
| V066 | Need for prophylactic vaccination and inoculation against streptococcus pneumoniae [pneumococcus] and influenza |  |  |  |  |  |  |  |  |  |  |
| V1261 | Personal history of pneumonia (recurrent) |  |  |  |  |  |  |  |  |  |  |
| Z8701 | Personal history of pneumonia (recurrent) |  |  |  |  |  |  |  |  |  |  |
| 490 | Lymphocytic choriomeningitis |  |  |  |  |  |  |  |  |  |  |
| 4910 | Simple chronic bronchitis |  |  |  |  |  |  |  |  |  |  |
| 4911 | Mucopurulent chronic bronchitis |  |  |  |  |  |  |  |  |  |  |
| 49120 | Obstructive chronic bronchitis without exacerbation |  |  |  |  |  |  |  |  |  |  |
| 49121 | Obstructive chronic bronchitis with (acute) exacerbation |  |  |  |  |  |  |  |  |  |  |
| 49122 | Obstructive chronic bronchitis with acute bronchitis |  |  |  |  |  |  |  |  |  |  |
| 4918 | Other chronic bronchitis |  |  |  |  |  |  |  |  |  |  |
| 4919 | Unspecified chronic bronchitis |  |  |  |  |  |  |  |  |  |  |
| 5060 | Bronchitis and pneumonitis due to fumes and vapors |  |  |  |  |  |  |  |  |  |  |
| J40 | Bronchitis, not specified as acute or chronic |  |  |  |  |  |  |  |  |  |  |
| J410 | Simple chronic bronchitis |  |  |  |  |  |  |  |  |  |  |
| J411 | Mucopurulent chronic bronchitis |  |  |  |  |  |  |  |  |  |  |
| J418 | Mixed simple and mucopurulent chronic bronchitis |  |  |  |  |  |  |  |  |  |  |
| J42 | Unspecified chronic bronchitis |  |  |  |  |  |  |  |  |  |  |
| J680 | Bronchitis and pneumonitis due to chemicals, gases, fumes and vapors |  |  |  |  |  |  |  |  |  |  |
| V813 | Screening for chronic bronchitis and emphysema |  |  |  |  |  |  |  |  |  |  |
| J440 | Chronic obstructive pulmonary disease with (acute) lower respiratory infection |  |  |  |  |  |  |  |  |  |  |
| J441 | Chronic obstructive pulmonary disease with (acute) exacerbation |  |  |  |  |  |  |  |  |  |  |
| J449 | Chronic obstructive pulmonary disease, unspecified |  |  |  |  |  |  |  |  |  |  |
| 1180 | Other specified pulmonary tuberculosis, unspecified |  |  |  |  |  |  |  |  |  |  |
| 1181 | Other specified pulmonary tuberculosis, bacteriological or histological examination not done |  |  |  |  |  |  |  |  |  |  |
| 1182 | Other specified pulmonary tuberculosis, bacteriological or histological examination unknown (at present) |  |  |  |  |  |  |  |  |  |  |
| 1183 | Other specified pulmonary tuberculosis, tubercle bacilli found (in sputum) by microscopy |  |  |  |  |  |  |  |  |  |  |
| 1184 | Other specified pulmonary tuberculosis, tubercle bacilli not found (in sputum) by microscopy, but found by bacterial culture |  |  |  |  |  |  |  |  |  |  |
| 1185 | Other specified pulmonary tuberculosis, tubercle bacilli not found by bacteriological examination, but tuberculosis confirmed histologically |  |  |  |  |  |  |  |  |  |  |
| 1186 | Other specified pulmonary tuberculosis, tubercle bacilli not found by bacteriological or histological examination, but tuberculosis confirmed by other methods [inoculation of animals] |  |  |  |  |  |  |  |  |  |  |
| 1190 | Pulmonary tuberculosis, unspecified, unspecified |  |  |  |  |  |  |  |  |  |  |
| 1191 | Pulmonary tuberculosis, unspecified, bacteriological or histological examination not done |  |  |  |  |  |  |  |  |  |  |
| 1192 | Pulmonary tuberculosis, unspecified, bacteriological or histological examination unknown (at present) |  |  |  |  |  |  |  |  |  |  |
| 1193 | Pulmonary tuberculosis, unspecified, tubercle bacilli found (in sputum) by microscopy |  |  |  |  |  |  |  |  |  |  |
| 1194 | Pulmonary tuberculosis, unspecified, tubercle bacilli not found (in sputum) by microscopy, but found by bacterial culture |  |  |  |  |  |  |  |  |  |  |
| 1195 | Pulmonary tuberculosis, unspecified, tubercle bacilli not found by bacteriological examination, but tuberculosis confirmed histologically |  |  |  |  |  |  |  |  |  |  |
| 1196 | Pulmonary tuberculosis, unspecified, tubercle bacilli not found by bacteriological or histological examination, but tuberculosis confirmed by other methods [inoculation of animals] |  |  |  |  |  |  |  |  |  |  |
| 1220 | Echinococcus granulosus infection of liver |  |  |  |  |  |  |  |  |  |  |
| 1221 | Echinococcus granulosus infection of lung |  |  |  |  |  |  |  |  |  |  |
| 1222 | Echinococcus granulosus infection of thyroid |  |  |  |  |  |  |  |  |  |  |
| 1223 | Echinococcus granulosus infection, other |  |  |  |  |  |  |  |  |  |  |
| 1224 | Echinococcus granulosus infection, unspecified |  |  |  |  |  |  |  |  |  |  |
| 1225 | Echinococcus multilocularis infection of liver |  |  |  |  |  |  |  |  |  |  |
| 1226 | Echinococcus multilocularis infection, other |  |  |  |  |  |  |  |  |  |  |
| 49300 | Extrinsic asthma, unspecified |  |  |  |  |  |  |  |  |  |  |
| 49301 | Extrinsic asthma with status asthmaticus |  |  |  |  |  |  |  |  |  |  |
| 49302 | Extrinsic asthma with (acute) exacerbation |  |  |  |  |  |  |  |  |  |  |
| 49310 | Intrinsic asthma, unspecified |  |  |  |  |  |  |  |  |  |  |
| 49311 | Intrinsic asthma with status asthmaticus |  |  |  |  |  |  |  |  |  |  |
| 49312 | Intrinsic asthma with (acute) exacerbation |  |  |  |  |  |  |  |  |  |  |
| 49320 | Chronic obstructive asthma, unspecified |  |  |  |  |  |  |  |  |  |  |
| 49321 | Chronic obstructive asthma with status asthmaticus |  |  |  |  |  |  |  |  |  |  |
| 49322 | Chronic obstructive asthma with (acute) exacerbation |  |  |  |  |  |  |  |  |  |  |
| 49382 | Exercise induced bronchospasm |  |  |  |  |  |  |  |  |  |  |
| 49390 | Cough variant asthma |  |  |  |  |  |  |  |  |  |  |
| 49391 | Asthma, unspecified type, unspecified |  |  |  |  |  |  |  |  |  |  |
| 49392 | Asthma, unspecified type, with status asthmaticus |  |  |  |  |  |  |  |  |  |  |
| J4520 | Asthma, unspecified type, with (acute) exacerbation |  |  |  |  |  |  |  |  |  |  |
| J4521 | Mild intermittent asthma with (acute) exacerbation |  |  |  |  |  |  |  |  |  |  |
| J4522 | Mild intermittent asthma with status asthmaticus |  |  |  |  |  |  |  |  |  |  |
| J4530 | Mild persistent asthma, uncomplicated |  |  |  |  |  |  |  |  |  |  |
| J4531 | Mild persistent asthma with (acute) exacerbation |  |  |  |  |  |  |  |  |  |  |
| J4532 | Mild persistent asthma with status asthmaticus |  |  |  |  |  |  |  |  |  |  |
| J4540 | Moderate persistent asthma, uncomplicated |  |  |  |  |  |  |  |  |  |  |
| J4541 | Moderate persistent asthma with (acute) exacerbation |  |  |  |  |  |  |  |  |  |  |
| J4542 | Moderate persistent asthma with status asthmaticus |  |  |  |  |  |  |  |  |  |  |
| J4550 | Severe persistent asthma, uncomplicated |  |  |  |  |  |  |  |  |  |  |
| J4551 | Severe persistent asthma with (acute) exacerbation |  |  |  |  |  |  |  |  |  |  |
| J4552 | Severe persistent asthma with status asthmaticus |  |  |  |  |  |  |  |  |  |  |
| J45901 | Unspecified asthma with (acute) exacerbation |  |  |  |  |  |  |  |  |  |  |
| J45902 | Unspecified asthma with status asthmaticus |  |  |  |  |  |  |  |  |  |  |
| J45909 | Unspecified asthma, uncomplicated |  |  |  |  |  |  |  |  |  |  |
| J45991 | Cough variant asthma |  |  |  |  |  |  |  |  |  |  |
| J45998 | Other asthma |  |  |  |  |  |  |  |  |  |  |
| V175 | Family history of asthma |  |  |  |  |  |  |  |  |  |  |
| Z825 | Family history of asthma and other chronic lower respiratory diseases |  |  |  |  |  |  |  |  |  |  |
| 4168 | Other chronic pulmonary heart diseases |  |  |  |  |  |  |  |  |  |  |
| 4169 | Chronic pulmonary heart disease, unspecified |  |  |  |  |  |  |  |  |  |  |
| I2789 | Other specified pulmonary heart diseases |  |  |  |  |  |  |  |  |  |  |
| I279 | Pulmonary heart disease, unspecified |  |  |  |  |  |  |  |  |  |  |
| 1144 | Tuberculous fibrosis of lung, tubercle bacilli not found (in sputum) by microscopy, but found by bacterial culture |  |  |  |  |  |  |  |  |  |  |
| B381 | Chronic pulmonary coccidioidomycosis |  |  |  |  |  |  |  |  |  |  |
| B391 | Chronic pulmonary histoplasmosis capsulati |  |  |  |  |  |  |  |  |  |  |
| B401 | Chronic pulmonary blastomycosis |  |  |  |  |  |  |  |  |  |  |
| I2782 | Chronic pulmonary embolism |  |  |  |  |  |  |  |  |  |  |
| J811 | Chronic pulmonary edema |  |  |  |  |  |  |  |  |  |  |
| J953 | Chronic pulmonary insufficiency following surgery |  |  |  |  |  |  |  |  |  |  |
| 41511 | Iatrogenic pulmonary embolism and infarction |  |  |  |  |  |  |  |  |  |  |
| 41512 | Septic pulmonary embolism |  |  |  |  |  |  |  |  |  |  |
| 41519 | Other pulmonary embolism and infarction |  |  |  |  |  |  |  |  |  |  |
| 4162 | Chronic pulmonary embolism |  |  |  |  |  |  |  |  |  |  |
| 67380 | Other obstetrical pulmonary embolism, unspecified as to episode of care or not applicable |  |  |  |  |  |  |  |  |  |  |
| 67381 | Other obstetrical pulmonary embolism, delivered, with or without mention of antepartum condition |  |  |  |  |  |  |  |  |  |  |
| 67382 | Other obstetrical pulmonary embolism, delivered, with mention of postpartum complication |  |  |  |  |  |  |  |  |  |  |
| 67383 | Other obstetrical pulmonary embolism, antepartum condition or complication |  |  |  |  |  |  |  |  |  |  |
| 67384 | Other obstetrical pulmonary embolism, postpartum condition or complication |  |  |  |  |  |  |  |  |  |  |
| I2609 | Other pulmonary embolism with acute cor pulmonale |  |  |  |  |  |  |  |  |  |  |
| I2601 | Septic pulmonary embolism with acute cor pulmonale |  |  |  |  |  |  |  |  |  |  |
| I2690 | Septic pulmonary embolism without acute cor pulmonale |  |  |  |  |  |  |  |  |  |  |
| I2693 | Single subsegmental pulmonary embolism without acute cor pulmonale |  |  |  |  |  |  |  |  |  |  |
| I2699 | Other pulmonary embolism without acute cor pulmonale |  |  |  |  |  |  |  |  |  |  |
| I2782 | Chronic pulmonary embolism |  |  |  |  |  |  |  |  |  |  |
| J811 | Chronic pulmonary edema |  |  |  |  |  |  |  |  |  |  |
| 501 | Alastrim |  |  |  |  |  |  |  |  |  |  |
| 504 | Pneumonopathy due to inhalation of other dust |  |  |  |  |  |  |  |  |  |  |
| 51853 | Acute and chronic respiratory failure following trauma and surgery |  |  |  |  |  |  |  |  |  |  |
| 51883 | Chronic respiratory failure |  |  |  |  |  |  |  |  |  |  |
| 51884 | Acute and chronic respiratory failure |  |  |  |  |  |  |  |  |  |  |
| 7707 | Chronic respiratory disease arising in the perinatal period |  |  |  |  |  |  |  |  |  |  |
| J684 | Chronic respiratory conditions due to chemicals, gases, fumes and vapors |  |  |  |  |  |  |  |  |  |  |
| J9610 | Chronic respiratory failure, unspecified whether with hypoxia or hypercapnia |  |  |  |  |  |  |  |  |  |  |
| J9611 | Chronic respiratory failure with hypoxia |  |  |  |  |  |  |  |  |  |  |
| J9612 | Chronic respiratory failure with hypercapnia |  |  |  |  |  |  |  |  |  |  |
| J9620 | Acute and chronic respiratory failure, unspecified whether with hypoxia or hypercapnia |  |  |  |  |  |  |  |  |  |  |
| J9621 | Acute and chronic respiratory failure with hypoxia |  |  |  |  |  |  |  |  |  |  |
| J9622 | Acute and chronic respiratory failure with hypercapnia |  |  |  |  |  |  |  |  |  |  |
| J9690 | Respiratory failure, unspecified, unspecified whether with hypoxia or hypercapnia |  |  |  |  |  |  |  |  |  |  |
| J9691 | Respiratory failure, unspecified with hypoxia |  |  |  |  |  |  |  |  |  |  |
| J9692 | Respiratory failure, unspecified with hypercapnia |  |  |  |  |  |  |  |  |  |  |
| P278 | Other chronic respiratory diseases originating in the perinatal period |  |  |  |  |  |  |  |  |  |  |
| P279 | Unspecified chronic respiratory disease originating in the perinatal period |  |  |  |  |  |  |  |  |  |  |
| P285 | Respiratory failure of newborn |  |  |  |  |  |  |  |  |  |  |
| 51889 | Other diseases of lung, not elsewhere classified |  |  |  |  |  |  |  |  |  |  |
| J84115 | Respiratory bronchiolitis interstitial lung disease |  |  |  |  |  |  |  |  |  |  |
| 51634 | Respiratory bronchiolitis interstitial lung disease |  |  |  |  |  |  |  |  |  |  |
| I2723 | Pulmonary hypertension due to lung diseases and hypoxia |  |  |  |  |  |  |  |  |  |  |
