# Supplementary material for: Relationship between Nonhepatic Serum Ammonia Levels and Sepsis-Associated Encephalopathy: A Retrospective Cohort Study
Source: Emerg Med Int. 2023 Oct 12;2023:6676033. doi: 10.1155/2023/6676033 (PMC10590267; doi:10.1155/2023/6676033)
Supplement: Supplementary Materials — 1: exclude patients with trauma of the skull from the MIMIC IV database according to ICD codes. Supplementary materials 2: exclude patients with intracerebral hemorrhage, cerebral embolism, and ischemic stroke disease from the MIMIC IV database according to ICD codes. Supplementary materials 3: exclude patients with meningitis and encephalitis disease from the MIMIC IV database according to ICD codes. Supplementary materials 4: exclude patients with epilepsy disease from the MIMIC IV database according to ICD codes. Supplementary materials 5: exclude patients with other cerebrovascular disease from the MIMIC IV database according to ICD codes. Supplementary materials 6: exclude patients with mental disorders and neurological disease from the MIMIC IV database according to ICD codes. Supplementary materials 7: exclude patients with alcoholic intoxication or drug abuse from the MIMIC IV database according to ICD codes. Supplementary materials 8: exclude patients with metabolic encephalopathy, hepatic encephalopathy, hypertensive encephalopathy, diabetes with coma, disorders of urea cycle, hypernatremia, and Wernicke's encephalopathy from the MIMIC IV database according to ICD codes. Supplementary materials 9: exclude patients with acute and chronic liver disease. Supplementary materials 10: hypertension disease and ICD codes. Supplementary materials 11: diabetes disease and ICD codes. Supplementary materials 12: lung disease and ICD codes. Supplementary materials 13: cardiovascular diseases and ICD codes. Supplementary materials 14: renal disease from the MIMIC IV database according to ICD codes. Supplementary materials 15: the standardized mean differences of the original cohort were compared with those of the IPW cohorts in sepsis patients. SMD: standardized mean differences. [file 6676033.f1.zip › Supplementary materials.13.docx]

| **Supplementary materials.13** Cardiovascular diseases and ICD-codes | | |  |  |  |  |  |  |  |  |  |
| --- | --- | --- | --- | --- | --- | --- | --- | --- | --- | --- | --- |
| Disease | ICD-Code | Description |  |  |  |  |  |  |  |  |  |
| Cardiovascular diseases |  |  |  |  |  |  |  |  |  |  |  |
|  |  |  |  |  |  |  |  |  |  |  |  |
|  | 9381 | Syphilitic pericarditis |  |  |  |  |  |  |  |  |  |
|  | 9382 | Syphilitic myocarditis |  |  |  |  |  |  |  |  |  |
|  | 9883 | Gonococcal pericarditis |  |  |  |  |  |  |  |  |  |
|  | 9884 | Gonococcal endocarditis |  |  |  |  |  |  |  |  |  |
|  | 9885 | Other gonococcal heart disease |  |  |  |  |  |  |  |  |  |
|  | 11513 | Infection by Histoplasma duboisii, pericarditis |  |  |  |  |  |  |  |  |  |
|  | 11514 | Infection by Histoplasma duboisii, endocarditis |  |  |  |  |  |  |  |  |  |
|  | 39890 | Rheumatic heart disease, unspecified |  |  |  |  |  |  |  |  |  |
|  | 39899 | Other rheumatic heart diseases |  |  |  |  |  |  |  |  |  |
|  | 40201 | Malignant hypertensive heart disease with heart failure |  |  |  |  |  |  |  |  |  |
|  | 40211 | Benign hypertensive heart disease with heart failure |  |  |  |  |  |  |  |  |  |
|  | 40290 | Unspecified hypertensive heart disease without heart failure |  |  |  |  |  |  |  |  |  |
|  | 40291 | Unspecified hypertensive heart disease with heart failure |  |  |  |  |  |  |  |  |  |
|  | 40401 | Hypertensive heart and chronic kidney disease, malignant, with heart failure and with chronic kidney disease stage I through stage IV, or unspecified |  |  |  |  |  |  |  |  |  |
|  | 40403 | Hypertensive heart and chronic kidney disease, malignant, with heart failure and with chronic kidney disease stage V or end stage renal disease |  |  |  |  |  |  |  |  |  |
|  | 40411 | Hypertensive heart and chronic kidney disease, benign, with heart failure and with chronic kidney disease stage I through stage IV, or unspecified |  |  |  |  |  |  |  |  |  |
|  | 40413 | Hypertensive heart and chronic kidney disease, benign, with heart failure and chronic kidney disease stage V or end stage renal disease |  |  |  |  |  |  |  |  |  |
|  | 40491 | Hypertensive heart and chronic kidney disease, unspecified, with heart failure and with chronic kidney disease stage I through stage IV, or unspecified |  |  |  |  |  |  |  |  |  |
|  | 40493 | Hypertensive heart and chronic kidney disease, unspecified, with heart failure and chronic kidney disease stage V or end stage renal disease |  |  |  |  |  |  |  |  |  |
|  | 41000 | Acute myocardial infarction of anterolateral wall, episode of care |  |  |  |  |  |  |  |  |  |
|  | 41001 | Acute myocardial infarction of anterolateral wall, initial episode of care |  |  |  |  |  |  |  |  |  |
|  | 41002 | Acute myocardial infarction of anterolateral wall, subsequent episode of care |  |  |  |  |  |  |  |  |  |
|  | 41010 | Acute myocardial infarction of other anterior wall, episode of care unspecified |  |  |  |  |  |  |  |  |  |
|  | 41011 | Acute myocardial infarction of other anterior wall, initial episode of care |  |  |  |  |  |  |  |  |  |
|  | 41012 | Acute myocardial infarction of other anterior wall, subsequent episode of care |  |  |  |  |  |  |  |  |  |
|  | 41020 | Acute myocardial infarction of inferolateral wall, episode of care unspecified |  |  |  |  |  |  |  |  |  |
|  | 41021 | Acute myocardial infarction of inferolateral wall, initial episode of care |  |  |  |  |  |  |  |  |  |
|  | 41022 | Acute myocardial infarction of inferolateral wall, subsequent episode of care |  |  |  |  |  |  |  |  |  |
|  | 41030 | Acute myocardial infarction of inferoposterior wall, episode of care unspecified |  |  |  |  |  |  |  |  |  |
|  | 41031 | Acute myocardial infarction of inferoposterior wall, initial episode of care |  |  |  |  |  |  |  |  |  |
|  | 41032 | Acute myocardial infarction of inferoposterior wall, subsequent episode of care |  |  |  |  |  |  |  |  |  |
|  | 41040 | Acute myocardial infarction of other inferior wall, episode of care unspecified |  |  |  |  |  |  |  |  |  |
|  | 41041 | Acute myocardial infarction of other inferior wall, initial episode of care |  |  |  |  |  |  |  |  |  |
|  | 41042 | Acute myocardial infarction of other inferior wall, subsequent episode of care |  |  |  |  |  |  |  |  |  |
|  | 41050 | Acute myocardial infarction of other lateral wall, episode of care unspecified |  |  |  |  |  |  |  |  |  |
|  | 41051 | Acute myocardial infarction of other lateral wall, initial episode of care |  |  |  |  |  |  |  |  |  |
|  | 41052 | Acute myocardial infarction of other lateral wall, subsequent episode of care |  |  |  |  |  |  |  |  |  |
|  | 41060 | True posterior wall infarction, episode of care unspecified |  |  |  |  |  |  |  |  |  |
|  | 41061 | True posterior wall infarction, initial episode of care |  |  |  |  |  |  |  |  |  |
|  | 41062 | True posterior wall infarction, subsequent episode of care |  |  |  |  |  |  |  |  |  |
|  | 41070 | Subendocardial infarction, episode of care unspecified |  |  |  |  |  |  |  |  |  |
|  | 41071 | Subendocardial infarction, initial episode of care |  |  |  |  |  |  |  |  |  |
|  | 41072 | Subendocardial infarction, subsequent episode of care |  |  |  |  |  |  |  |  |  |
|  | 41080 | Acute myocardial infarction of other specified sites, episode of care unspecified |  |  |  |  |  |  |  |  |  |
|  | 41081 | Acute myocardial infarction of other specified sites, initial episode of care |  |  |  |  |  |  |  |  |  |
|  | 41082 | Acute myocardial infarction of other specified sites, subsequent episode of care |  |  |  |  |  |  |  |  |  |
|  | 41090 | Acute myocardial infarction of unspecified site, episode of care unspecified |  |  |  |  |  |  |  |  |  |
|  | 41091 | Acute myocardial infarction of unspecified site, initial episode of care |  |  |  |  |  |  |  |  |  |
|  | 41092 | Acute myocardial infarction of unspecified site, subsequent episode of care |  |  |  |  |  |  |  |  |  |
|  | 4110 | Postmyocardial infarction syndrome |  |  |  |  |  |  |  |  |  |
|  | 4111 | Intermediate coronary syndrome |  |  |  |  |  |  |  |  |  |
|  | 41181 | Acute coronary occlusion without myocardial infarction |  |  |  |  |  |  |  |  |  |
|  | 41189 | Other acute and subacute forms of ischemic heart disease, other |  |  |  |  |  |  |  |  |  |
|  | 412 | Old myocardial infarction |  |  |  |  |  |  |  |  |  |
|  | 4130 | Angina decubitus |  |  |  |  |  |  |  |  |  |
|  | 4131 | Prinzmetal angina |  |  |  |  |  |  |  |  |  |
|  | 4139 | Other and unspecified angina pectoris |  |  |  |  |  |  |  |  |  |
|  | 41400 | Coronary atherosclerosis of unspecified type of vessel, native or graft |  |  |  |  |  |  |  |  |  |
|  | 41401 | Coronary atherosclerosis of native coronary artery |  |  |  |  |  |  |  |  |  |
|  | 41402 | Coronary atherosclerosis of autologous vein bypass graft |  |  |  |  |  |  |  |  |  |
|  | 41404 | Coronary atherosclerosis of artery bypass graft |  |  |  |  |  |  |  |  |  |
|  | 41405 | Coronary atherosclerosis of unspecified bypass graft |  |  |  |  |  |  |  |  |  |
|  | 41406 | Coronary atherosclerosis of native coronary artery of transplanted heart |  |  |  |  |  |  |  |  |  |
|  | 41407 | Coronary atherosclerosis of bypass graft (artery) (vein) of transplanted heart |  |  |  |  |  |  |  |  |  |
|  | 41410 | Aneurysm of heart (wall) |  |  |  |  |  |  |  |  |  |
|  | 41411 | Aneurysm of coronary vessels |  |  |  |  |  |  |  |  |  |
|  | 41412 | Dissection of coronary artery |  |  |  |  |  |  |  |  |  |
|  | 42090 | Acute pericarditis, unspecified |  |  |  |  |  |  |  |  |  |
|  | 42091 | Acute idiopathic pericarditis |  |  |  |  |  |  |  |  |  |
|  | 42099 | Other acute pericarditis |  |  |  |  |  |  |  |  |  |
|  | 4210 | Acute and subacute bacterial endocarditis |  |  |  |  |  |  |  |  |  |
|  | 4211 | Acute and subacute infective endocarditis in diseases classified elsewhere |  |  |  |  |  |  |  |  |  |
|  | 4219 | Acute endocarditis, unspecified |  |  |  |  |  |  |  |  |  |
|  | 4220 | Acute myocarditis in diseases classified elsewhere |  |  |  |  |  |  |  |  |  |
|  | 42290 | Acute myocarditis, unspecified |  |  |  |  |  |  |  |  |  |
|  | 42291 | Idiopathic myocarditis |  |  |  |  |  |  |  |  |  |
|  | 42292 | Septic myocarditis |  |  |  |  |  |  |  |  |  |
|  | 42293 | Toxic myocarditis |  |  |  |  |  |  |  |  |  |
|  | 42299 | Other acute myocarditis |  |  |  |  |  |  |  |  |  |
|  | 4230 | Hemopericardium |  |  |  |  |  |  |  |  |  |
|  | 4231 | Adhesive pericarditis |  |  |  |  |  |  |  |  |  |
|  | 4232 | Constrictive pericarditis |  |  |  |  |  |  |  |  |  |
|  | 4233 | Cardiac tamponade |  |  |  |  |  |  |  |  |  |
|  | 4238 | Other specified diseases of pericardium |  |  |  |  |  |  |  |  |  |
|  | 4240 | Mitral valve disorders |  |  |  |  |  |  |  |  |  |
|  | 4241 | Aortic valve disorders |  |  |  |  |  |  |  |  |  |
|  | 4242 | Tricuspid valve disorders, specified as nonrheumatic |  |  |  |  |  |  |  |  |  |
|  | 4243 | Pulmonary valve disorders |  |  |  |  |  |  |  |  |  |
|  | 42490 | Endocarditis, valve unspecified, unspecified cause |  |  |  |  |  |  |  |  |  |
|  | 42491 | Endocarditis in diseases classified elsewhere |  |  |  |  |  |  |  |  |  |
|  | 42499 | Other endocarditis, valve unspecified |  |  |  |  |  |  |  |  |  |
|  | 4250 | Endomyocardial fibrosis |  |  |  |  |  |  |  |  |  |
|  | 42511 | Hypertrophic obstructive cardiomyopathy |  |  |  |  |  |  |  |  |  |
|  | 42518 | Other hypertrophic cardiomyopathy |  |  |  |  |  |  |  |  |  |
|  | 4252 | Obscure cardiomyopathy of Africa |  |  |  |  |  |  |  |  |  |
|  | 4253 | Endocardial fibroelastosis |  |  |  |  |  |  |  |  |  |
|  | 4254 | Other primary cardiomyopathies |  |  |  |  |  |  |  |  |  |
|  | 4255 | Toxic myocarditis |  |  |  |  |  |  |  |  |  |
|  | 4257 | Nutritional and metabolic cardiomyopathy |  |  |  |  |  |  |  |  |  |
|  | 4258 | Cardiomyopathy in other diseases classified elsewhere |  |  |  |  |  |  |  |  |  |
|  | 4259 | Secondary cardiomyopathy, unspecified |  |  |  |  |  |  |  |  |  |
|  | 4260 | Atrioventricular block, complete |  |  |  |  |  |  |  |  |  |
|  | 42610 | Atrioventricular block, unspecified |  |  |  |  |  |  |  |  |  |
|  | 42611 | First degree atrioventricular block |  |  |  |  |  |  |  |  |  |
|  | 42612 | Mobitz (type) II atrioventricular block |  |  |  |  |  |  |  |  |  |
|  | 42613 | Other second degree atrioventricular block |  |  |  |  |  |  |  |  |  |
|  | 4262 | Left bundle branch hemiblock |  |  |  |  |  |  |  |  |  |
|  | 4263 | Other left bundle branch block |  |  |  |  |  |  |  |  |  |
|  | 4264 | Right bundle branch block |  |  |  |  |  |  |  |  |  |
|  | 42650 | Bundle branch block, unspecified |  |  |  |  |  |  |  |  |  |
|  | 42651 | Right bundle branch block and left posterior fascicular block |  |  |  |  |  |  |  |  |  |
|  | 42652 | Right bundle branch block and left anterior fascicular block |  |  |  |  |  |  |  |  |  |
|  | 42653 | Other bilateral bundle branch block |  |  |  |  |  |  |  |  |  |
|  | 42654 | Trifascicular block |  |  |  |  |  |  |  |  |  |
|  | 4266 | Other heart block |  |  |  |  |  |  |  |  |  |
|  | 4267 | Anomalous atrioventricular excitation |  |  |  |  |  |  |  |  |  |
|  | 42681 | Lown-Ganong-Levine syndrome |  |  |  |  |  |  |  |  |  |
|  | 42682 | Long QT syndrome |  |  |  |  |  |  |  |  |  |
|  | 42689 | Other specified conduction disorders |  |  |  |  |  |  |  |  |  |
|  | 4269 | Conduction disorder, unspecified |  |  |  |  |  |  |  |  |  |
|  | 4270 | Paroxysmal supraventricular tachycardia |  |  |  |  |  |  |  |  |  |
|  | 4271 | Paroxysmal ventricular tachycardia |  |  |  |  |  |  |  |  |  |
|  | 4272 | Paroxysmal tachycardia, unspecified |  |  |  |  |  |  |  |  |  |
|  | 42731 | Atrial fibrillation |  |  |  |  |  |  |  |  |  |
|  | 42732 | Atrial flutter |  |  |  |  |  |  |  |  |  |
|  | 42741 | Ventricular fibrillation |  |  |  |  |  |  |  |  |  |
|  | 42742 | Ventricular flutter |  |  |  |  |  |  |  |  |  |
|  | 4275 | Cardiac arrest |  |  |  |  |  |  |  |  |  |
|  | 42760 | Premature beats, unspecified |  |  |  |  |  |  |  |  |  |
|  | 42761 | Supraventricular premature beats |  |  |  |  |  |  |  |  |  |
|  | 42769 | Other premature beats |  |  |  |  |  |  |  |  |  |
|  | 42781 | Sinoatrial node dysfunction |  |  |  |  |  |  |  |  |  |
|  | 42789 | Other specified cardiac dysrhythmias |  |  |  |  |  |  |  |  |  |
|  | 4279 | Cardiac dysrhythmia, unspecified |  |  |  |  |  |  |  |  |  |
|  | 4280 | Congestive heart failure, unspecified |  |  |  |  |  |  |  |  |  |
|  | 4281 | Left heart failure |  |  |  |  |  |  |  |  |  |
|  | 42820 | Systolic heart failure, unspecified |  |  |  |  |  |  |  |  |  |
|  | 42821 | Acute systolic heart failure |  |  |  |  |  |  |  |  |  |
|  | 42822 | Chronic systolic heart failure |  |  |  |  |  |  |  |  |  |
|  | 42823 | Acute on chronic systolic heart failure |  |  |  |  |  |  |  |  |  |
|  | 42830 | Diastolic heart failure, unspecified |  |  |  |  |  |  |  |  |  |
|  | 42831 | Acute diastolic heart failure |  |  |  |  |  |  |  |  |  |
|  | 42832 | Chronic diastolic heart failure |  |  |  |  |  |  |  |  |  |
|  | 42833 | Acute on chronic diastolic heart failure |  |  |  |  |  |  |  |  |  |
|  | 42840 | Combined systolic and diastolic heart failure, unspecified |  |  |  |  |  |  |  |  |  |
|  | 42841 | Acute combined systolic and diastolic heart failure |  |  |  |  |  |  |  |  |  |
|  | 42842 | Chronic combined systolic and diastolic heart failure |  |  |  |  |  |  |  |  |  |
|  | 42843 | Acute on chronic combined systolic and diastolic heart failure |  |  |  |  |  |  |  |  |  |
|  | 4289 | Heart failure, unspecified |  |  |  |  |  |  |  |  |  |
|  | 4290 | Myocarditis, unspecified |  |  |  |  |  |  |  |  |  |
|  | 4291 | Myocardial degeneration |  |  |  |  |  |  |  |  |  |
|  | 4292 | Cardiovascular disease, unspecified |  |  |  |  |  |  |  |  |  |
|  | 4293 | Cardiomegaly |  |  |  |  |  |  |  |  |  |
|  | 4294 | Functional disturbances following cardiac surgery |  |  |  |  |  |  |  |  |  |
|  | 4295 | Rupture of chordae tendineae |  |  |  |  |  |  |  |  |  |
|  | 4296 | Rupture of papillary muscle |  |  |  |  |  |  |  |  |  |
|  | 42971 | Acquired cardiac septal defect |  |  |  |  |  |  |  |  |  |
|  | 42979 | Certain sequelae of myocardial infarction, not elsewhere classified, other |  |  |  |  |  |  |  |  |  |
|  | 42981 | Other disorders of papillary muscle |  |  |  |  |  |  |  |  |  |
|  | 42982 | Hyperkinetic heart disease |  |  |  |  |  |  |  |  |  |
|  | 42983 | Takotsubo syndrome |  |  |  |  |  |  |  |  |  |
|  | 42989 | Other ill-defined heart diseases |  |  |  |  |  |  |  |  |  |
|  | 4299 | Heart disease, unspecified |  |  |  |  |  |  |  |  |  |
|  | 3910 | Acute rheumatic pericarditis |  |  |  |  |  |  |  |  |  |
|  | 3911 | Acute rheumatic endocarditis |  |  |  |  |  |  |  |  |  |
|  | 3912 | Acute rheumatic myocarditis |  |  |  |  |  |  |  |  |  |
|  | 3918 | Other acute rheumatic heart disease |  |  |  |  |  |  |  |  |  |
|  | 3919 | Acute rheumatic heart disease, unspecified |  |  |  |  |  |  |  |  |  |
|  | 3920 | Rheumatic chorea with heart involvement |  |  |  |  |  |  |  |  |  |
|  | 3929 | Rheumatic chorea without mention of heart involvement |  |  |  |  |  |  |  |  |  |
|  | 393 | Chronic rheumatic pericarditis |  |  |  |  |  |  |  |  |  |
|  | 3940 | Mitral stenosis |  |  |  |  |  |  |  |  |  |
|  | 3941 | Rheumatic mitral insufficiency |  |  |  |  |  |  |  |  |  |
|  | 3942 | Mitral stenosis with insufficiency |  |  |  |  |  |  |  |  |  |
|  | 3949 | Other and unspecified mitral valve diseases |  |  |  |  |  |  |  |  |  |
|  | 3950 | Rheumatic aortic stenosis |  |  |  |  |  |  |  |  |  |
|  | 3951 | Rheumatic aortic insufficiency |  |  |  |  |  |  |  |  |  |
|  | 3952 | Rheumatic aortic stenosis with insufficiency |  |  |  |  |  |  |  |  |  |
|  | 3959 | Other and unspecified rheumatic aortic diseases |  |  |  |  |  |  |  |  |  |
|  | 3960 | Mitral valve stenosis and aortic valve stenosis |  |  |  |  |  |  |  |  |  |
|  | 3961 | Mitral valve stenosis and aortic valve insufficiency |  |  |  |  |  |  |  |  |  |
|  | 3962 | Mitral valve insufficiency and aortic valve stenosis |  |  |  |  |  |  |  |  |  |
|  | 3963 | Mitral valve insufficiency and aortic valve insufficiency |  |  |  |  |  |  |  |  |  |
|  | 3968 | Multiple involvement of mitral and aortic valves |  |  |  |  |  |  |  |  |  |
|  | 3969 | Mitral and aortic valve diseases, unspecified |  |  |  |  |  |  |  |  |  |
|  | 3970 | Diseases of tricuspid valve |  |  |  |  |  |  |  |  |  |
|  | 3971 | Rheumatic diseases of pulmonary valve |  |  |  |  |  |  |  |  |  |
|  | 3979 | Rheumatic diseases of endocardium, valve unspecified |  |  |  |  |  |  |  |  |  |
|  | 3980 | Rheumatic myocarditis |  |  |  |  |  |  |  |  |  |
|  | I092 | Chronic rheumatic pericarditis |  |  |  |  |  |  |  |  |  |
|  | I300 | Acute nonspecific idiopathic pericarditis |  |  |  |  |  |  |  |  |  |
|  | I301 | Infective pericarditis |  |  |  |  |  |  |  |  |  |
|  | I308 | Other forms of acute pericarditis |  |  |  |  |  |  |  |  |  |
|  | I309 | Acute pericarditis, unspecified |  |  |  |  |  |  |  |  |  |
|  | I310 | Chronic adhesive pericarditis |  |  |  |  |  |  |  |  |  |
|  | I311 | Chronic constrictive pericarditis |  |  |  |  |  |  |  |  |  |
|  | I32 | Pericarditis in diseases classified elsewhere |  |  |  |  |  |  |  |  |  |
|  | M3212 | Pericarditis in systemic lupus erythematosus |  |  |  |  |  |  |  |  |  |
|  | A5206 | Other syphilitic heart involvement |  |  |  |  |  |  |  |  |  |
|  | A5483 | Gonococcal heart infection |  |  |  |  |  |  |  |  |  |
|  | B570 | Acute Chagas' disease with heart involvement |  |  |  |  |  |  |  |  |  |
|  | B571 | Acute Chagas' disease without heart involvement |  |  |  |  |  |  |  |  |  |
|  | B572 | Chagas' disease (chronic) with heart involvement |  |  |  |  |  |  |  |  |  |
|  | E8706 | Accidental cut, puncture, perforation or hemorrhage during heart catheterization |  |  |  |  |  |  |  |  |  |
|  | E8726 | Failure of sterile precautions during heart catheterization |  |  |  |  |  |  |  |  |  |
|  | E8745 | Mechanical failure of instrument or apparatus during heart catheterization |  |  |  |  |  |  |  |  |  |
|  | I00 | Rheumatic fever without heart involvement |  |  |  |  |  |  |  |  |  |
|  | I018 | Other acute rheumatic heart disease |  |  |  |  |  |  |  |  |  |
|  | I019 | Acute rheumatic heart disease, unspecified |  |  |  |  |  |  |  |  |  |
|  | I020 | Rheumatic chorea with heart involvement |  |  |  |  |  |  |  |  |  |
|  | I029 | Rheumatic chorea without heart involvement |  |  |  |  |  |  |  |  |  |
|  | I0981 | Rheumatic heart failure |  |  |  |  |  |  |  |  |  |
|  | I0989 | Other specified rheumatic heart diseases |  |  |  |  |  |  |  |  |  |
|  | I099 | Rheumatic heart disease, unspecified |  |  |  |  |  |  |  |  |  |
|  | I110 | Hypertensive heart disease with heart failure |  |  |  |  |  |  |  |  |  |
|  | I119 | Hypertensive heart disease without heart failure |  |  |  |  |  |  |  |  |  |
|  | I130 | Hypertensive heart and chronic kidney disease with heart failure and stage 1 through stage 4 chronic kidney disease, or unspecified chronic kidney disease |  |  |  |  |  |  |  |  |  |
|  | I1310 | Hypertensive heart and chronic kidney disease without heart failure, with stage 1 through stage 4 chronic kidney disease, or unspecified chronic kidney disease |  |  |  |  |  |  |  |  |  |
|  | I1311 | Hypertensive heart and chronic kidney disease without heart failure, with stage 5 chronic kidney disease, or end stage renal disease |  |  |  |  |  |  |  |  |  |
|  | I132 | Hypertensive heart and chronic kidney disease with heart failure and with stage 5 chronic kidney disease, or end stage renal disease |  |  |  |  |  |  |  |  |  |
|  | I248 | Other forms of acute ischemic heart disease |  |  |  |  |  |  |  |  |  |
|  | I249 | Acute ischemic heart disease, unspecified |  |  |  |  |  |  |  |  |  |
|  | I2510 | Atherosclerotic heart disease of native coronary artery without angina pectoris |  |  |  |  |  |  |  |  |  |
|  | I25110 | Atherosclerotic heart disease of native coronary artery with unstable angina pectoris |  |  |  |  |  |  |  |  |  |
|  | I25111 | Atherosclerotic heart disease of native coronary artery with angina pectoris with documented spasm |  |  |  |  |  |  |  |  |  |
|  | I25118 | Atherosclerotic heart disease of native coronary artery with other forms of angina pectoris |  |  |  |  |  |  |  |  |  |
|  | I25119 | Atherosclerotic heart disease of native coronary artery with unspecified angina pectoris |  |  |  |  |  |  |  |  |  |
|  | I252 | Old myocardial infarction |  |  |  |  |  |  |  |  |  |
|  | I253 | Aneurysm of heart |  |  |  |  |  |  |  |  |  |
|  | I25750 | Atherosclerosis of native coronary artery of transplanted heart with unstable angina |  |  |  |  |  |  |  |  |  |
|  | I25751 | Atherosclerosis of native coronary artery of transplanted heart with angina pectoris with documented spasm |  |  |  |  |  |  |  |  |  |
|  | I25758 | Atherosclerosis of native coronary artery of transplanted heart with other forms of angina pectoris |  |  |  |  |  |  |  |  |  |
|  | I25759 | Atherosclerosis of native coronary artery of transplanted heart with unspecified angina pectoris |  |  |  |  |  |  |  |  |  |
|  | I25750 | Atherosclerosis of native coronary artery of transplanted heart with unstable angina |  |  |  |  |  |  |  |  |  |
|  | I25751 | Atherosclerosis of native coronary artery of transplanted heart with angina pectoris with documented spasm |  |  |  |  |  |  |  |  |  |
|  | I25758 | Atherosclerosis of native coronary artery of transplanted heart with other forms of angina pectoris |  |  |  |  |  |  |  |  |  |
|  | I25759 | Atherosclerosis of native coronary artery of transplanted heart with unspecified angina pectoris |  |  |  |  |  |  |  |  |  |
|  | I25811 | Atherosclerosis of native coronary artery of transplanted heart without angina pectoris |  |  |  |  |  |  |  |  |  |
|  | I25812 | Atherosclerosis of bypass graft of coronary artery of transplanted heart without angina pectoris |  |  |  |  |  |  |  |  |  |
|  | I2589 | Other forms of chronic ischemic heart disease |  |  |  |  |  |  |  |  |  |
|  | I259 | Chronic ischemic heart disease, unspecified |  |  |  |  |  |  |  |  |  |
|  | I271 | Kyphoscoliotic heart disease |  |  |  |  |  |  |  |  |  |
|  | I2722 | Pulmonary hypertension due to left heart disease |  |  |  |  |  |  |  |  |  |
|  | I2722 | Pulmonary hypertension due to left heart disease |  |  |  |  |  |  |  |  |  |
|  | I2722 | Pulmonary hypertension due to left heart disease |  |  |  |  |  |  |  |  |  |
|  | I2722 | Pulmonary hypertension due to left heart disease |  |  |  |  |  |  |  |  |  |
|  | I2789 | Other specified pulmonary heart diseases |  |  |  |  |  |  |  |  |  |
|  | I279 | Pulmonary heart disease, unspecified |  |  |  |  |  |  |  |  |  |
|  | I2789 | Other specified pulmonary heart diseases |  |  |  |  |  |  |  |  |  |
|  | I279 | Pulmonary heart disease, unspecified |  |  |  |  |  |  |  |  |  |
|  | I39 | Endocarditis and heart valve disorders in diseases classified elsewhere |  |  |  |  |  |  |  |  |  |
|  | I455 | Other specified heart block |  |  |  |  |  |  |  |  |  |
|  | I5020 | Unspecified systolic (congestive) heart failure |  |  |  |  |  |  |  |  |  |
|  | I5021 | Acute systolic (congestive) heart failure |  |  |  |  |  |  |  |  |  |
|  | I5022 | Chronic systolic (congestive) heart failure |  |  |  |  |  |  |  |  |  |
|  | I5023 | Acute on chronic systolic (congestive) heart failure |  |  |  |  |  |  |  |  |  |
|  | I5030 | Unspecified diastolic (congestive) heart failure |  |  |  |  |  |  |  |  |  |
|  | I5031 | Acute diastolic (congestive) heart failure |  |  |  |  |  |  |  |  |  |
|  | I5032 | Chronic diastolic (congestive) heart failure |  |  |  |  |  |  |  |  |  |
|  | I5033 | Acute on chronic diastolic (congestive) heart failure |  |  |  |  |  |  |  |  |  |
|  | I5040 | Unspecified combined systolic (congestive) and diastolic (congestive) heart failure |  |  |  |  |  |  |  |  |  |
|  | I5041 | Acute combined systolic (congestive) and diastolic (congestive) heart failure |  |  |  |  |  |  |  |  |  |
|  | I5042 | Chronic combined systolic (congestive) and diastolic (congestive) heart failure |  |  |  |  |  |  |  |  |  |
|  | I5043 | Acute on chronic combined systolic (congestive) and diastolic (congestive) heart failure |  |  |  |  |  |  |  |  |  |
|  | I50810 | Right heart failure, unspecified |  |  |  |  |  |  |  |  |  |
|  | I50811 | Acute right heart failure |  |  |  |  |  |  |  |  |  |
|  | I50812 | Chronic right heart failure |  |  |  |  |  |  |  |  |  |
|  | I50813 | Acute on chronic right heart failure |  |  |  |  |  |  |  |  |  |
|  | I50814 | Right heart failure due to left heart failure |  |  |  |  |  |  |  |  |  |
|  | I5082 | Biventricular heart failure |  |  |  |  |  |  |  |  |  |
|  | I5083 | High output heart failure |  |  |  |  |  |  |  |  |  |
|  | I5084 | End stage heart failure |  |  |  |  |  |  |  |  |  |
|  | I5089 | Other heart failure |  |  |  |  |  |  |  |  |  |
|  | I509 | Heart failure, unspecified |  |  |  |  |  |  |  |  |  |
|  | I519 | Heart disease, unspecified |  |  |  |  |  |  |  |  |  |
|  | I52 | Other heart disorders in diseases classified elsewhere |  |  |  |  |  |  |  |  |  |
|  | I97130 | Postprocedural heart failure following cardiac surgery |  |  |  |  |  |  |  |  |  |
|  | I97131 | Postprocedural heart failure following other surgery |  |  |  |  |  |  |  |  |  |
|  | M0530 | Rheumatoid heart disease with rheumatoid arthritis of unspecified site |  |  |  |  |  |  |  |  |  |
|  | M05311 | Rheumatoid heart disease with rheumatoid arthritis of right shoulder |  |  |  |  |  |  |  |  |  |
|  | M05312 | Rheumatoid heart disease with rheumatoid arthritis of left shoulder |  |  |  |  |  |  |  |  |  |
|  | M05319 | Rheumatoid heart disease with rheumatoid arthritis of unspecified shoulder |  |  |  |  |  |  |  |  |  |
|  | M05321 | Rheumatoid heart disease with rheumatoid arthritis of right elbow |  |  |  |  |  |  |  |  |  |
|  | M05322 | Rheumatoid heart disease with rheumatoid arthritis of left elbow |  |  |  |  |  |  |  |  |  |
|  | M05329 | Rheumatoid heart disease with rheumatoid arthritis of unspecified elbow |  |  |  |  |  |  |  |  |  |
|  | M05331 | Rheumatoid heart disease with rheumatoid arthritis of right wrist |  |  |  |  |  |  |  |  |  |
|  | M05332 | Rheumatoid heart disease with rheumatoid arthritis of left wrist |  |  |  |  |  |  |  |  |  |
|  | M05339 | Rheumatoid heart disease with rheumatoid arthritis of unspecified wrist |  |  |  |  |  |  |  |  |  |
|  | M05341 | Rheumatoid heart disease with rheumatoid arthritis of right hand |  |  |  |  |  |  |  |  |  |
|  | M05342 | Rheumatoid heart disease with rheumatoid arthritis of left hand |  |  |  |  |  |  |  |  |  |
|  | M05349 | Rheumatoid heart disease with rheumatoid arthritis of unspecified hand |  |  |  |  |  |  |  |  |  |
|  | M05351 | Rheumatoid heart disease with rheumatoid arthritis of right hip |  |  |  |  |  |  |  |  |  |
|  | M05352 | Rheumatoid heart disease with rheumatoid arthritis of left hip |  |  |  |  |  |  |  |  |  |
|  | M05359 | Rheumatoid heart disease with rheumatoid arthritis of unspecified hip |  |  |  |  |  |  |  |  |  |
|  | M05361 | Rheumatoid heart disease with rheumatoid arthritis of right knee |  |  |  |  |  |  |  |  |  |
|  | M05362 | Rheumatoid heart disease with rheumatoid arthritis of left knee |  |  |  |  |  |  |  |  |  |
|  | M05369 | Rheumatoid heart disease with rheumatoid arthritis of unspecified knee |  |  |  |  |  |  |  |  |  |
|  | M05371 | Rheumatoid heart disease with rheumatoid arthritis of right ankle and foot |  |  |  |  |  |  |  |  |  |
|  | M05372 | Rheumatoid heart disease with rheumatoid arthritis of left ankle and foot |  |  |  |  |  |  |  |  |  |
|  | M0530 | Rheumatoid heart disease with rheumatoid arthritis of unspecified site |  |  |  |  |  |  |  |  |  |
|  | M05311 | Rheumatoid heart disease with rheumatoid arthritis of right shoulder |  |  |  |  |  |  |  |  |  |
|  | M05312 | Rheumatoid heart disease with rheumatoid arthritis of left shoulder |  |  |  |  |  |  |  |  |  |
|  | M05319 | Rheumatoid heart disease with rheumatoid arthritis of unspecified shoulder |  |  |  |  |  |  |  |  |  |
|  | M05321 | Rheumatoid heart disease with rheumatoid arthritis of right elbow |  |  |  |  |  |  |  |  |  |
|  | M05322 | Rheumatoid heart disease with rheumatoid arthritis of left elbow |  |  |  |  |  |  |  |  |  |
|  | M05329 | Rheumatoid heart disease with rheumatoid arthritis of unspecified elbow |  |  |  |  |  |  |  |  |  |
|  | M05331 | Rheumatoid heart disease with rheumatoid arthritis of right wrist |  |  |  |  |  |  |  |  |  |
|  | M05332 | Rheumatoid heart disease with rheumatoid arthritis of left wrist |  |  |  |  |  |  |  |  |  |
|  | M05339 | Rheumatoid heart disease with rheumatoid arthritis of unspecified wrist |  |  |  |  |  |  |  |  |  |
|  | M05341 | Rheumatoid heart disease with rheumatoid arthritis of right hand |  |  |  |  |  |  |  |  |  |
|  | M05342 | Rheumatoid heart disease with rheumatoid arthritis of left hand |  |  |  |  |  |  |  |  |  |
|  | M05349 | Rheumatoid heart disease with rheumatoid arthritis of unspecified hand |  |  |  |  |  |  |  |  |  |
|  | M05351 | Rheumatoid heart disease with rheumatoid arthritis of right hip |  |  |  |  |  |  |  |  |  |
|  | M05352 | Rheumatoid heart disease with rheumatoid arthritis of left hip |  |  |  |  |  |  |  |  |  |
|  | M05359 | Rheumatoid heart disease with rheumatoid arthritis of unspecified hip |  |  |  |  |  |  |  |  |  |
|  | M05361 | Rheumatoid heart disease with rheumatoid arthritis of right knee |  |  |  |  |  |  |  |  |  |
|  | M05362 | Rheumatoid heart disease with rheumatoid arthritis of left knee |  |  |  |  |  |  |  |  |  |
|  | M05369 | Rheumatoid heart disease with rheumatoid arthritis of unspecified knee |  |  |  |  |  |  |  |  |  |
|  | M05371 | Rheumatoid heart disease with rheumatoid arthritis of right ankle and foot |  |  |  |  |  |  |  |  |  |
|  | O368310 | Maternal care for abnormalities of the fetal heart rate or rhythm, first trimester, not applicable or unspecified |  |  |  |  |  |  |  |  |  |
|  | O368311 | Maternal care for abnormalities of the fetal heart rate or rhythm, first trimester, fetus 1 |  |  |  |  |  |  |  |  |  |
|  | O368312 | Maternal care for abnormalities of the fetal heart rate or rhythm, first trimester, fetus 2 |  |  |  |  |  |  |  |  |  |
|  | O368313 | Maternal care for abnormalities of the fetal heart rate or rhythm, first trimester, fetus 3 |  |  |  |  |  |  |  |  |  |
|  | O368314 | Maternal care for abnormalities of the fetal heart rate or rhythm, first trimester, fetus 4 |  |  |  |  |  |  |  |  |  |
|  | O368315 | Maternal care for abnormalities of the fetal heart rate or rhythm, first trimester, fetus 5 |  |  |  |  |  |  |  |  |  |
|  | O368319 | Maternal care for abnormalities of the fetal heart rate or rhythm, first trimester, other fetus |  |  |  |  |  |  |  |  |  |
|  | O368320 | Maternal care for abnormalities of the fetal heart rate or rhythm, second trimester, not applicable or unspecified |  |  |  |  |  |  |  |  |  |
|  | O368321 | Maternal care for abnormalities of the fetal heart rate or rhythm, second trimester, fetus 1 |  |  |  |  |  |  |  |  |  |
|  | O368322 | Maternal care for abnormalities of the fetal heart rate or rhythm, second trimester, fetus 2 |  |  |  |  |  |  |  |  |  |
|  | O368323 | Maternal care for abnormalities of the fetal heart rate or rhythm, second trimester, fetus 3 |  |  |  |  |  |  |  |  |  |
|  | O368324 | Maternal care for abnormalities of the fetal heart rate or rhythm, second trimester, fetus 4 |  |  |  |  |  |  |  |  |  |
|  | O368325 | Maternal care for abnormalities of the fetal heart rate or rhythm, second trimester, fetus 5 |  |  |  |  |  |  |  |  |  |
|  | O368329 | Maternal care for abnormalities of the fetal heart rate or rhythm, second trimester, other fetus |  |  |  |  |  |  |  |  |  |
|  | O368330 | Maternal care for abnormalities of the fetal heart rate or rhythm, third trimester, not applicable or unspecified |  |  |  |  |  |  |  |  |  |
|  | O368331 | Maternal care for abnormalities of the fetal heart rate or rhythm, third trimester, fetus 1 |  |  |  |  |  |  |  |  |  |
|  | O368332 | Maternal care for abnormalities of the fetal heart rate or rhythm, third trimester, fetus 2 |  |  |  |  |  |  |  |  |  |
|  | O368333 | Maternal care for abnormalities of the fetal heart rate or rhythm, third trimester, fetus 3 |  |  |  |  |  |  |  |  |  |
|  | O368334 | Maternal care for abnormalities of the fetal heart rate or rhythm, third trimester, fetus 4 |  |  |  |  |  |  |  |  |  |
|  | O368335 | Maternal care for abnormalities of the fetal heart rate or rhythm, third trimester, fetus 5 |  |  |  |  |  |  |  |  |  |
|  | O368339 | Maternal care for abnormalities of the fetal heart rate or rhythm, third trimester, other fetus |  |  |  |  |  |  |  |  |  |
|  | O368390 | Maternal care for abnormalities of the fetal heart rate or rhythm, unspecified trimester, not applicable or unspecified |  |  |  |  |  |  |  |  |  |
|  | O368391 | Maternal care for abnormalities of the fetal heart rate or rhythm, unspecified trimester, fetus 1 |  |  |  |  |  |  |  |  |  |
|  | O368392 | Maternal care for abnormalities of the fetal heart rate or rhythm, unspecified trimester, fetus 2 |  |  |  |  |  |  |  |  |  |
|  | O368393 | Maternal care for abnormalities of the fetal heart rate or rhythm, unspecified trimester, fetus 3 |  |  |  |  |  |  |  |  |  |
|  | O368394 | Maternal care for abnormalities of the fetal heart rate or rhythm, unspecified trimester, fetus 4 |  |  |  |  |  |  |  |  |  |
|  | O368395 | Maternal care for abnormalities of the fetal heart rate or rhythm, unspecified trimester, fetus 5 |  |  |  |  |  |  |  |  |  |
|  | O368399 | Maternal care for abnormalities of the fetal heart rate or rhythm, unspecified trimester, other fetus | M05372 |  |  |  |  |  |  |  |  |
|  | O76 | Abnormality in fetal heart rate and rhythm complicating labor and delivery | M05379 |  |  |  |  |  |  |  |  |
|  | Q234 | Hypoplastic left heart syndrome | M0539 |  |  |  |  |  |  |  |  |
|  | Q226 | Hypoplastic right heart syndrome | O10111 |  |  |  |  |  |  |  |  |
|  | Q246 | Congenital heart block | O10112 |  |  |  |  |  |  |  |  |
|  | Q248 | Other specified congenital malformations of heart | O10113 |  |  |  |  |  |  |  |  |
|  | Q249 | Congenital malformation of heart, unspecified | O10119 |  |  |  |  |  |  |  |  |
|  | R008 | Other abnormalities of heart beat | O1012 |  |  |  |  |  |  |  |  |
|  | R009 | Unspecified abnormalities of heart beat |  |  |  |  |  |  |  |  |  |
|  | R931 | Abnormal findings on diagnostic imaging of heart and coronary circulation |  |  |  |  |  |  |  |  |  |
|  | S2600XA | Unspecified injury of heart with hemopericardium, initial encounter |  |  |  |  |  |  |  |  |  |
|  | S2600XD | Unspecified injury of heart with hemopericardium, subsequent encounter |  |  |  |  |  |  |  |  |  |
|  | S2600XS | Unspecified injury of heart with hemopericardium, sequela |  |  |  |  |  |  |  |  |  |
|  | S2601XA | Contusion of heart with hemopericardium, initial encounter |  |  |  |  |  |  |  |  |  |
|  | S2601XD | Contusion of heart with hemopericardium, subsequent encounter |  |  |  |  |  |  |  |  |  |
|  | S2601XS | Contusion of heart with hemopericardium, sequela |  |  |  |  |  |  |  |  |  |
|  | S26020A | Mild laceration of heart with hemopericardium, initial encounter |  |  |  |  |  |  |  |  |  |
|  | S26020D | Mild laceration of heart with hemopericardium, subsequent encounter |  |  |  |  |  |  |  |  |  |
|  | S26020S | Mild laceration of heart with hemopericardium, sequela |  |  |  |  |  |  |  |  |  |
|  | S26021A | Moderate laceration of heart with hemopericardium, initial encounter |  |  |  |  |  |  |  |  |  |
|  | S26021D | Moderate laceration of heart with hemopericardium, subsequent encounter |  |  |  |  |  |  |  |  |  |
|  | S26021S | Moderate laceration of heart with hemopericardium, sequela |  |  |  |  |  |  |  |  |  |
|  | S26022A | Major laceration of heart with hemopericardium, initial encounter |  |  |  |  |  |  |  |  |  |
|  | S26022D | Major laceration of heart with hemopericardium, subsequent encounter |  |  |  |  |  |  |  |  |  |
|  | S26022S | Major laceration of heart with hemopericardium, sequela |  |  |  |  |  |  |  |  |  |
|  | S2609XA | Other injury of heart with hemopericardium, initial encounter |  |  |  |  |  |  |  |  |  |
|  | S2609XD | Other injury of heart with hemopericardium, subsequent encounter |  |  |  |  |  |  |  |  |  |
|  | S2609XS | Other injury of heart with hemopericardium, sequela |  |  |  |  |  |  |  |  |  |
|  | S2610XA | Unspecified injury of heart without hemopericardium, initial encounter |  |  |  |  |  |  |  |  |  |
|  | S2610XD | Unspecified injury of heart without hemopericardium, subsequent encounter |  |  |  |  |  |  |  |  |  |
|  | S2610XS | Unspecified injury of heart without hemopericardium, sequela |  |  |  |  |  |  |  |  |  |
|  | S2611XA | Contusion of heart without hemopericardium, initial encounter |  |  |  |  |  |  |  |  |  |
|  | S2611XD | Contusion of heart without hemopericardium, subsequent encounter |  |  |  |  |  |  |  |  |  |
|  | S2611XS | Contusion of heart without hemopericardium, sequela |  |  |  |  |  |  |  |  |  |
|  | S2612XA | Laceration of heart without hemopericardium, initial encounter |  |  |  |  |  |  |  |  |  |
|  | S2612XD | Laceration of heart without hemopericardium, subsequent encounter |  |  |  |  |  |  |  |  |  |
|  | S2612XS | Laceration of heart without hemopericardium, sequela |  |  |  |  |  |  |  |  |  |
|  | S2619XA | Other injury of heart without hemopericardium, initial encounter |  |  |  |  |  |  |  |  |  |
|  | S2619XD | Other injury of heart without hemopericardium, subsequent encounter |  |  |  |  |  |  |  |  |  |
|  | S2619XS | Other injury of heart without hemopericardium, sequela |  |  |  |  |  |  |  |  |  |
|  | S2690XA | Unspecified injury of heart, unspecified with or without hemopericardium, initial encounter |  |  |  |  |  |  |  |  |  |
|  | S2690XD | Unspecified injury of heart, unspecified with or without hemopericardium, subsequent encounter |  |  |  |  |  |  |  |  |  |
|  | S2690XS | Unspecified injury of heart, unspecified with or without hemopericardium, sequela |  |  |  |  |  |  |  |  |  |
|  | S2691XA | Contusion of heart, unspecified with or without hemopericardium, initial encounter |  |  |  |  |  |  |  |  |  |
|  | S2691XD | Contusion of heart, unspecified with or without hemopericardium, subsequent encounter |  |  |  |  |  |  |  |  |  |
|  | S2691XS | Contusion of heart, unspecified with or without hemopericardium, sequela |  |  |  |  |  |  |  |  |  |
|  | S2692XA | Laceration of heart, unspecified with or without hemopericardium, initial encounter |  |  |  |  |  |  |  |  |  |
|  | S2692XD | Laceration of heart, unspecified with or without hemopericardium, subsequent encounter |  |  |  |  |  |  |  |  |  |
|  | S2692XS | Laceration of heart, unspecified with or without hemopericardium, sequela |  |  |  |  |  |  |  |  |  |
|  | S2699XA | Other injury of heart, unspecified with or without hemopericardium, initial encounter |  |  |  |  |  |  |  |  |  |
|  | S2699XD | Other injury of heart, unspecified with or without hemopericardium, subsequent encounter |  |  |  |  |  |  |  |  |  |
|  | S2699XS | Other injury of heart, unspecified with or without hemopericardium, sequela |  |  |  |  |  |  |  |  |  |
|  | T81505A | Unspecified complication of foreign body accidentally left in body following heart catheterization, initial encounter |  |  |  |  |  |  |  |  |  |
|  | T81505D | Unspecified complication of foreign body accidentally left in body following heart catheterization, subsequent encounter |  |  |  |  |  |  |  |  |  |
|  | T81505S | Unspecified complication of foreign body accidentally left in body following heart catheterization, sequela |  |  |  |  |  |  |  |  |  |
|  | T81525A | Obstruction due to foreign body accidentally left in body following heart catheterization, initial encounter |  |  |  |  |  |  |  |  |  |
|  | T81525D | Obstruction due to foreign body accidentally left in body following heart catheterization, subsequent encounter |  |  |  |  |  |  |  |  |  |
|  | T81525S | Obstruction due to foreign body accidentally left in body following heart catheterization, sequela |  |  |  |  |  |  |  |  |  |
|  | T81535A | Perforation due to foreign body accidentally left in body following heart catheterization, initial encounter |  |  |  |  |  |  |  |  |  |
|  | T81535D | Perforation due to foreign body accidentally left in body following heart catheterization, subsequent encounter |  |  |  |  |  |  |  |  |  |
|  | T81535S | Perforation due to foreign body accidentally left in body following heart catheterization, sequela |  |  |  |  |  |  |  |  |  |
|  | T81595A | Other complications of foreign body accidentally left in body following heart catheterization, initial encounter |  |  |  |  |  |  |  |  |  |
|  | T81595D | Other complications of foreign body accidentally left in body following heart catheterization, subsequent encounter |  |  |  |  |  |  |  |  |  |
|  | T81595S | Other complications of foreign body accidentally left in body following heart catheterization, sequela |  |  |  |  |  |  |  |  |  |
|  | T8201XA | Breakdown (mechanical) of heart valve prosthesis, initial encounter |  |  |  |  |  |  |  |  |  |
|  | T8201XD | Breakdown (mechanical) of heart valve prosthesis, subsequent encounter |  |  |  |  |  |  |  |  |  |
|  | T8201XS | Breakdown (mechanical) of heart valve prosthesis, sequela |  |  |  |  |  |  |  |  |  |
|  | T8202XA | Displacement of heart valve prosthesis, initial encounter |  |  |  |  |  |  |  |  |  |
|  | T8202XD | Displacement of heart valve prosthesis, subsequent encounter |  |  |  |  |  |  |  |  |  |
|  | T8202XS | Displacement of heart valve prosthesis, sequela |  |  |  |  |  |  |  |  |  |
|  | T8203XA | Leakage of heart valve prosthesis, initial encounter |  |  |  |  |  |  |  |  |  |
|  | T8203XD | Leakage of heart valve prosthesis, subsequent encounter |  |  |  |  |  |  |  |  |  |
|  | T8203XS | Leakage of heart valve prosthesis, sequela |  |  |  |  |  |  |  |  |  |
|  | T8209XA | Other mechanical complication of heart valve prosthesis, initial encounter |  |  |  |  |  |  |  |  |  |
|  | T8209XD | Other mechanical complication of heart valve prosthesis, subsequent encounter |  |  |  |  |  |  |  |  |  |
|  | T8209XS | Other mechanical complication of heart valve prosthesis, sequela |  |  |  |  |  |  |  |  |  |
|  | T82221A | Breakdown (mechanical) of biological heart valve graft, initial encounter |  |  |  |  |  |  |  |  |  |
|  | T82221D | Breakdown (mechanical) of biological heart valve graft, subsequent encounter |  |  |  |  |  |  |  |  |  |
|  | T82221S | Breakdown (mechanical) of biological heart valve graft, sequela |  |  |  |  |  |  |  |  |  |
|  | T82222A | Displacement of biological heart valve graft, initial encounter |  |  |  |  |  |  |  |  |  |
|  | T82222D | Displacement of biological heart valve graft, subsequent encounter |  |  |  |  |  |  |  |  |  |
|  | T82222S | Displacement of biological heart valve graft, sequela |  |  |  |  |  |  |  |  |  |
|  | T86298 | Other complications of heart transplant |  |  |  |  |  |  |  |  |  |
|  | V1365 | Personal history of (corrected) congenital malformations of heart and circulatory system |  |  |  |  |  |  |  |  |  |
|  | V151 | Personal history of surgery to heart and great vessels, presenting hazards to health |  |  |  |  |  |  |  |  |  |
|  | V4321 | Organ or tissue replaced by other means, heart assist device |  |  |  |  |  |  |  |  |  |
|  | V810 | Screening for ischemic heart disease |  |  |  |  |  |  |  |  |  |
|  | Y625 | Failure of sterile precautions during heart catheterization |  |  |  |  |  |  |  |  |  |
|  | Z4821 | Encounter for aftercare following heart transplant |  |  |  |  |  |  |  |  |  |
|  | Z952 | Presence of prosthetic heart valve |  |  |  |  |  |  |  |  |  |
|  | Z953 | Presence of xenogenic heart valve |  |  |  |  |  |  |  |  |  |
|  | Z954 | Presence of other heart-valve replacement |  |  |  |  |  |  |  |  |  |
|  | Z955 | Presence of coronary angioplasty implant and graft |  |  |  |  |  |  |  |  |  |
|  | Z95810 | Presence of automatic (implantable) cardiac defibrillator |  |  |  |  |  |  |  |  |  |
|  | Z95811 | Presence of heart assist device |  |  |  |  |  |  |  |  |  |
|  | Z95812 | Presence of fully implantable artificial heart |  |  |  |  |  |  |  |  |  |
|  | Z95818 | Presence of other cardiac implants and grafts |  |  |  |  |  |  |  |  |  |
|  | E8790 | Cardiac catheterization as the cause of abnormal reaction of patient, or of later complication, without mention of misadventure at time of procedure |  |  |  |  |  |  |  |  |  |
|  | E9420 | Cardiac rhythm regulators causing adverse effects in therapeutic use |  |  |  |  |  |  |  |  |  |
|  | I233 | Rupture of cardiac wall without hemopericardium as current complication following acute myocardial infarction |  |  |  |  |  |  |  |  |  |
|  | I314 | Cardiac tamponade |  |  |  |  |  |  |  |  |  |
|  | I462 | Cardiac arrest due to underlying cardiac condition |  |  |  |  |  |  |  |  |  |
|  | I468 | Cardiac arrest due to other underlying condition |  |  |  |  |  |  |  |  |  |
|  | I469 | Cardiac arrest, cause unspecified |  |  |  |  |  |  |  |  |  |
|  | I498 | Other specified cardiac arrhythmias |  |  |  |  |  |  |  |  |  |
|  | I499 | Cardiac arrhythmia, unspecified |  |  |  |  |  |  |  |  |  |
|  | I510 | Cardiac septal defect, acquired |  |  |  |  |  |  |  |  |  |
|  | I513 | Intracardiac thrombosis, not elsewhere classified |  |  |  |  |  |  |  |  |  |
|  | I97110 | Postprocedural cardiac insufficiency following cardiac surgery |  |  |  |  |  |  |  |  |  |
|  | I97111 | Postprocedural cardiac insufficiency following other surgery |  |  |  |  |  |  |  |  |  |
|  | I97120 | Postprocedural cardiac arrest following cardiac surgery |  |  |  |  |  |  |  |  |  |
|  | J371 | Chronic laryngotracheitis |  |  |  |  |  |  |  |  |  |
|  | I97121 | Postprocedural cardiac arrest following other surgery |  |  |  |  |  |  |  |  |  |
|  | I97410 | Intraoperative hemorrhage and hematoma of a circulatory system organ or structure complicating a cardiac catheterization |  |  |  |  |  |  |  |  |  |
|  | I97411 | Intraoperative hemorrhage and hematoma of a circulatory system organ or structure complicating a cardiac bypass |  |  |  |  |  |  |  |  |  |
|  | I97610 | Postprocedural hemorrhage of a circulatory system organ or structure following a cardiac catheterization |  |  |  |  |  |  |  |  |  |
|  | I97611 | Postprocedural hemorrhage of a circulatory system organ or structure following cardiac bypass |  |  |  |  |  |  |  |  |  |
|  | I97630 | Postprocedural hematoma of a circulatory system organ or structure following a cardiac catheterization |  |  |  |  |  |  |  |  |  |
|  | I97631 | Postprocedural hematoma of a circulatory system organ or structure following cardiac bypass |  |  |  |  |  |  |  |  |  |
|  | I97638 | Postprocedural hematoma of a circulatory system organ or structure following other circulatory system procedure |  |  |  |  |  |  |  |  |  |
|  | I97640 | Postprocedural seroma of a circulatory system organ or structure following a cardiac catheterization |  |  |  |  |  |  |  |  |  |
|  | I97641 | Postprocedural seroma of a circulatory system organ or structure following cardiac bypass |  |  |  |  |  |  |  |  |  |
|  | I97648 | Postprocedural seroma of a circulatory system organ or structure following other circulatory system procedure |  |  |  |  |  |  |  |  |  |
|  | I97710 | Intraoperative cardiac arrest during cardiac surgery |  |  |  |  |  |  |  |  |  |
|  | I97711 | Intraoperative cardiac arrest during other surgery |  |  |  |  |  |  |  |  |  |
|  | I97790 | Other intraoperative cardiac functional disturbances during cardiac surgery |  |  |  |  |  |  |  |  |  |
|  | I97791 | Other intraoperative cardiac functional disturbances during other surgery |  |  |  |  |  |  |  |  |  |
|  | I97810 | Intraoperative cerebrovascular infarction during cardiac surgery |  |  |  |  |  |  |  |  |  |
|  | I97811 | Intraoperative cerebrovascular infarction during other surgery |  |  |  |  |  |  |  |  |  |
|  | I97820 | Postprocedural cerebrovascular infarction following cardiac surgery |  |  |  |  |  |  |  |  |  |
|  | I97821 | Postprocedural cerebrovascular infarction following other surgery |  |  |  |  |  |  |  |  |  |
|  | I9788 | Other intraoperative complications of the circulatory system, not elsewhere classified |  |  |  |  |  |  |  |  |  |
|  | O0336 | Cardiac arrest following incomplete spontaneous abortion |  |  |  |  |  |  |  |  |  |
|  | O0386 | Cardiac arrest following complete or unspecified spontaneous abortion |  |  |  |  |  |  |  |  |  |
|  | O0486 | Cardiac arrest following (induced) termination of pregnancy |  |  |  |  |  |  |  |  |  |
|  | O0736 | Cardiac arrest following failed attempted termination of pregnancy |  |  |  |  |  |  |  |  |  |
|  | O0881 | Cardiac arrest following an ectopic and molar pregnancy |  |  |  |  |  |  |  |  |  |
|  | O29111 | Cardiac arrest due to anesthesia during pregnancy, first trimester |  |  |  |  |  |  |  |  |  |
|  | O29112 | Cardiac arrest due to anesthesia during pregnancy, second trimester |  |  |  |  |  |  |  |  |  |
|  | O29113 | Cardiac arrest due to anesthesia during pregnancy, third trimester |  |  |  |  |  |  |  |  |  |
|  | O29119 | Cardiac arrest due to anesthesia during pregnancy, unspecified trimester |  |  |  |  |  |  |  |  |  |
|  | O29121 | Cardiac failure due to anesthesia during pregnancy, first trimester |  |  |  |  |  |  |  |  |  |
|  | O29122 | Cardiac failure due to anesthesia during pregnancy, second trimester |  |  |  |  |  |  |  |  |  |
|  | O29123 | Cardiac failure due to anesthesia during pregnancy, third trimester |  |  |  |  |  |  |  |  |  |
|  | O29129 | Cardiac failure due to anesthesia during pregnancy, unspecified trimester |  |  |  |  |  |  |  |  |  |
|  | O29192 | Other cardiac complications of anesthesia during pregnancy, second trimester |  |  |  |  |  |  |  |  |  |
|  | O29193 | Other cardiac complications of anesthesia during pregnancy, third trimester |  |  |  |  |  |  |  |  |  |
|  | O29199 | Other cardiac complications of anesthesia during pregnancy, unspecified trimester |  |  |  |  |  |  |  |  |  |
|  | O742 | Cardiac complications of anesthesia during labor and delivery |  |  |  |  |  |  |  |  |  |
|  | O891 | Cardiac complications of anesthesia during the puerperium |  |  |  |  |  |  |  |  |  |
|  | P290 | Neonatal cardiac failure |  |  |  |  |  |  |  |  |  |
|  | P2911 | Neonatal tachycardia |  |  |  |  |  |  |  |  |  |
|  | P2912 | Neonatal bradycardia |  |  |  |  |  |  |  |  |  |
|  | P292 | Neonatal hypertension |  |  |  |  |  |  |  |  |  |
|  | Q208 | Other congenital malformations of cardiac chambers and connections |  |  |  |  |  |  |  |  |  |
|  | Q209 | Congenital malformation of cardiac chambers and connections, unspecified |  |  |  |  |  |  |  |  |  |
|  | Q218 | Other congenital malformations of cardiac septa |  |  |  |  |  |  |  |  |  |
|  | R010 | Benign and innocent cardiac murmurs |  |  |  |  |  |  |  |  |  |
|  | R011 | Cardiac murmur, unspecified |  |  |  |  |  |  |  |  |  |
|  | R012 | Other cardiac sounds |  |  |  |  |  |  |  |  |  |
|  | T460X1A | Poisoning by cardiac-stimulant glycosides and drugs of similar action, accidental (unintentional), initial encounter |  |  |  |  |  |  |  |  |  |
|  | T460X1D | Poisoning by cardiac-stimulant glycosides and drugs of similar action, accidental (unintentional), subsequent encounter |  |  |  |  |  |  |  |  |  |
|  | T460X1S | Poisoning by cardiac-stimulant glycosides and drugs of similar action, accidental (unintentional), sequela |  |  |  |  |  |  |  |  |  |
|  | T460X2A | Poisoning by cardiac-stimulant glycosides and drugs of similar action, intentional self-harm, initial encounter |  |  |  |  |  |  |  |  |  |
|  | T460X2D | Poisoning by cardiac-stimulant glycosides and drugs of similar action, intentional self-harm, subsequent encounter |  |  |  |  |  |  |  |  |  |
|  | T460X2S | Poisoning by cardiac-stimulant glycosides and drugs of similar action, intentional self-harm, sequela |  |  |  |  |  |  |  |  |  |
|  | T460X3A | Poisoning by cardiac-stimulant glycosides and drugs of similar action, assault, initial encounter |  |  |  |  |  |  |  |  |  |
|  | T460X3D | Poisoning by cardiac-stimulant glycosides and drugs of similar action, assault, subsequent encounter |  |  |  |  |  |  |  |  |  |
|  | T460X3S | Poisoning by cardiac-stimulant glycosides and drugs of similar action, assault, sequela |  |  |  |  |  |  |  |  |  |
|  | T460X4A | Poisoning by cardiac-stimulant glycosides and drugs of similar action, undetermined, initial encounter |  |  |  |  |  |  |  |  |  |
|  | T460X4D | Poisoning by cardiac-stimulant glycosides and drugs of similar action, undetermined, subsequent encounter |  |  |  |  |  |  |  |  |  |
|  | T460X4S | Poisoning by cardiac-stimulant glycosides and drugs of similar action, undetermined, sequela |  |  |  |  |  |  |  |  |  |
|  | T460X5A | Adverse effect of cardiac-stimulant glycosides and drugs of similar action, initial encounter |  |  |  |  |  |  |  |  |  |
|  | T460X5D | Adverse effect of cardiac-stimulant glycosides and drugs of similar action, subsequent encounter |  |  |  |  |  |  |  |  |  |
|  | T460X5S | Adverse effect of cardiac-stimulant glycosides and drugs of similar action, sequela |  |  |  |  |  |  |  |  |  |
|  | T460X6A | Underdosing of cardiac-stimulant glycosides and drugs of similar action, initial encounter |  |  |  |  |  |  |  |  |  |
|  | T460X6D | Underdosing of cardiac-stimulant glycosides and drugs of similar action, subsequent encounter |  |  |  |  |  |  |  |  |  |
|  | T460X6S | Underdosing of cardiac-stimulant glycosides and drugs of similar action, sequela |  |  |  |  |  |  |  |  |  |
|  | T82110A | Breakdown (mechanical) of cardiac electrode, initial encounter |  |  |  |  |  |  |  |  |  |
|  | T82110D | Breakdown (mechanical) of cardiac electrode, subsequent encounter |  |  |  |  |  |  |  |  |  |
|  | T82110S | Breakdown (mechanical) of cardiac electrode, sequela |  |  |  |  |  |  |  |  |  |
|  | T82111A | Breakdown (mechanical) of cardiac pulse generator (battery), initial encounter |  |  |  |  |  |  |  |  |  |
|  | T82111D | Breakdown (mechanical) of cardiac pulse generator (battery), subsequent encounter |  |  |  |  |  |  |  |  |  |
|  | T82111S | Breakdown (mechanical) of cardiac pulse generator (battery), sequela |  |  |  |  |  |  |  |  |  |
|  | T82118A | Breakdown (mechanical) of other cardiac electronic device, initial encounter |  |  |  |  |  |  |  |  |  |
|  | T82118D | Breakdown (mechanical) of other cardiac electronic device, subsequent encounter |  |  |  |  |  |  |  |  |  |
|  | T82118S | Breakdown (mechanical) of other cardiac electronic device, sequela |  |  |  |  |  |  |  |  |  |
|  | T82119A | Breakdown (mechanical) of unspecified cardiac electronic device, initial encounter |  |  |  |  |  |  |  |  |  |
|  | T82119D | Breakdown (mechanical) of unspecified cardiac electronic device, subsequent encounter |  |  |  |  |  |  |  |  |  |
|  | T82119S | Breakdown (mechanical) of unspecified cardiac electronic device, sequela |  |  |  |  |  |  |  |  |  |
|  | T82120A | Displacement of cardiac electrode, initial encounter |  |  |  |  |  |  |  |  |  |
|  | T82120D | Displacement of cardiac electrode, subsequent encounter |  |  |  |  |  |  |  |  |  |
|  | T82120S | Displacement of cardiac electrode, sequela |  |  |  |  |  |  |  |  |  |
|  | T82121A | Displacement of cardiac pulse generator (battery), initial encounter |  |  |  |  |  |  |  |  |  |
|  | T82121D | Displacement of cardiac pulse generator (battery), subsequent encounter |  |  |  |  |  |  |  |  |  |
|  | T82121S | Displacement of cardiac pulse generator (battery), sequela |  |  |  |  |  |  |  |  |  |
|  | T82128A | Displacement of other cardiac electronic device, initial encounter |  |  |  |  |  |  |  |  |  |
|  | T82128D | Displacement of other cardiac electronic device, subsequent encounter |  |  |  |  |  |  |  |  |  |
|  | T82128S | Displacement of other cardiac electronic device, sequela |  |  |  |  |  |  |  |  |  |
|  | T82129A | Displacement of unspecified cardiac electronic device, initial encounter |  |  |  |  |  |  |  |  |  |
|  | T82129D | Displacement of unspecified cardiac electronic device, subsequent encounter |  |  |  |  |  |  |  |  |  |
|  | T82129S | Displacement of unspecified cardiac electronic device, sequela |  |  |  |  |  |  |  |  |  |
|  | T82190A | Other mechanical complication of cardiac electrode, initial encounter |  |  |  |  |  |  |  |  |  |
|  | T82190D | Other mechanical complication of cardiac electrode, subsequent encounter |  |  |  |  |  |  |  |  |  |
|  | T82190S | Other mechanical complication of cardiac electrode, sequela |  |  |  |  |  |  |  |  |  |
|  | T82191A | Other mechanical complication of cardiac pulse generator (battery), initial encounter |  |  |  |  |  |  |  |  |  |
|  | T82191D | Other mechanical complication of cardiac pulse generator (battery), subsequent encounter |  |  |  |  |  |  |  |  |  |
|  | T82191S | Other mechanical complication of cardiac pulse generator (battery), sequela |  |  |  |  |  |  |  |  |  |
|  | T82198A | Other mechanical complication of other cardiac electronic device, initial encounter |  |  |  |  |  |  |  |  |  |
|  | T82198D | Other mechanical complication of other cardiac electronic device, subsequent encounter |  |  |  |  |  |  |  |  |  |
|  | T82198S | Other mechanical complication of other cardiac electronic device, sequela |  |  |  |  |  |  |  |  |  |
|  | T82199A | Other mechanical complication of unspecified cardiac device, initial encounter |  |  |  |  |  |  |  |  |  |
|  | T82199D | Other mechanical complication of unspecified cardiac device, subsequent encounter |  |  |  |  |  |  |  |  |  |
|  | T82199S | Other mechanical complication of unspecified cardiac device, sequela |  |  |  |  |  |  |  |  |  |
|  | T82518D | Breakdown (mechanical) of other cardiac and vascular devices and implants, subsequent encounter |  |  |  |  |  |  |  |  |  |
|  | T82518S | Breakdown (mechanical) of other cardiac and vascular devices and implants, sequela |  |  |  |  |  |  |  |  |  |
|  | T82519A | Breakdown (mechanical) of unspecified cardiac and vascular devices and implants, initial encounter |  |  |  |  |  |  |  |  |  |
|  | T82519D | Breakdown (mechanical) of unspecified cardiac and vascular devices and implants, subsequent encounter |  |  |  |  |  |  |  |  |  |
|  | T82519S | Breakdown (mechanical) of unspecified cardiac and vascular devices and implants, sequela |  |  |  |  |  |  |  |  |  |
|  | T82538A | Leakage of other cardiac and vascular devices and implants, initial encounter |  |  |  |  |  |  |  |  |  |
|  | T82538D | Leakage of other cardiac and vascular devices and implants, subsequent encounter |  |  |  |  |  |  |  |  |  |
|  | T82538S | Leakage of other cardiac and vascular devices and implants, sequela |  |  |  |  |  |  |  |  |  |
|  | T82539A | Leakage of unspecified cardiac and vascular devices and implants, initial encounter |  |  |  |  |  |  |  |  |  |
|  | T82539D | Leakage of unspecified cardiac and vascular devices and implants, subsequent encounter |  |  |  |  |  |  |  |  |  |
|  | T82539S | Leakage of unspecified cardiac and vascular devices and implants, sequela |  |  |  |  |  |  |  |  |  |
|  | T82598A | Other mechanical complication of other cardiac and vascular devices and implants, initial encounter |  |  |  |  |  |  |  |  |  |
|  | T82598D | Other mechanical complication of other cardiac and vascular devices and implants, subsequent encounter |  |  |  |  |  |  |  |  |  |
|  | T82598S | Other mechanical complication of other cardiac and vascular devices and implants, sequela |  |  |  |  |  |  |  |  |  |
|  | T826XXA | Infection and inflammatory reaction due to cardiac valve prosthesis, initial encounter |  |  |  |  |  |  |  |  |  |
|  | T826XXD | Infection and inflammatory reaction due to cardiac valve prosthesis, subsequent encounter |  |  |  |  |  |  |  |  |  |
|  | T826XXS | Infection and inflammatory reaction due to cardiac valve prosthesis, sequela |  |  |  |  |  |  |  |  |  |
|  | T827XXA | Infection and inflammatory reaction due to other cardiac and vascular devices, implants and grafts, initial encounter |  |  |  |  |  |  |  |  |  |
|  | T827XXD | Infection and inflammatory reaction due to other cardiac and vascular devices, implants and grafts, subsequent encounter |  |  |  |  |  |  |  |  |  |
|  | T827XXS | Infection and inflammatory reaction due to other cardiac and vascular devices, implants and grafts, sequela |  |  |  |  |  |  |  |  |  |
|  | T82817A | Embolism due to cardiac prosthetic devices, implants and grafts, initial encounter |  |  |  |  |  |  |  |  |  |
|  | T82817D | Embolism due to cardiac prosthetic devices, implants and grafts, subsequent encounter |  |  |  |  |  |  |  |  |  |
|  | T82817S | Embolism due to cardiac prosthetic devices, implants and grafts, sequela |  |  |  |  |  |  |  |  |  |
|  | T826XXA | Infection and inflammatory reaction due to cardiac valve prosthesis, initial encounter |  |  |  |  |  |  |  |  |  |
|  | T826XXD | Infection and inflammatory reaction due to cardiac valve prosthesis, subsequent encounter |  |  |  |  |  |  |  |  |  |
|  | T826XXS | Infection and inflammatory reaction due to cardiac valve prosthesis, sequela |  |  |  |  |  |  |  |  |  |
|  | T827XXA | Infection and inflammatory reaction due to other cardiac and vascular devices, implants and grafts, initial encounter |  |  |  |  |  |  |  |  |  |
|  | T827XXD | Infection and inflammatory reaction due to other cardiac and vascular devices, implants and grafts, subsequent encounter |  |  |  |  |  |  |  |  |  |
|  | T827XXS | Infection and inflammatory reaction due to other cardiac and vascular devices, implants and grafts, sequela |  |  |  |  |  |  |  |  |  |
|  | T82817A | Embolism due to cardiac prosthetic devices, implants and grafts, initial encounter |  |  |  |  |  |  |  |  |  |
|  | T82817D | Embolism due to cardiac prosthetic devices, implants and grafts, subsequent encounter |  |  |  |  |  |  |  |  |  |
|  | T82817S | Embolism due to cardiac prosthetic devices, implants and grafts, sequela |  |  |  |  |  |  |  |  |  |
|  | T826XXA | Infection and inflammatory reaction due to cardiac valve prosthesis, initial encounter |  |  |  |  |  |  |  |  |  |
|  | T826XXD | Infection and inflammatory reaction due to cardiac valve prosthesis, subsequent encounter |  |  |  |  |  |  |  |  |  |
|  | T826XXS | Infection and inflammatory reaction due to cardiac valve prosthesis, sequela |  |  |  |  |  |  |  |  |  |
|  | T827XXA | Infection and inflammatory reaction due to other cardiac and vascular devices, implants and grafts, initial encounter |  |  |  |  |  |  |  |  |  |
|  | T827XXD | Infection and inflammatory reaction due to other cardiac and vascular devices, implants and grafts, subsequent encounter |  |  |  |  |  |  |  |  |  |
|  | T827XXS | Infection and inflammatory reaction due to other cardiac and vascular devices, implants and grafts, sequela |  |  |  |  |  |  |  |  |  |
|  | T82817A | Embolism due to cardiac prosthetic devices, implants and grafts, initial encounter |  |  |  |  |  |  |  |  |  |
|  | T82817D | Embolism due to cardiac prosthetic devices, implants and grafts, subsequent encounter |  |  |  |  |  |  |  |  |  |
|  | T82817S | Embolism due to cardiac prosthetic devices, implants and grafts, sequela |  |  |  |  |  |  |  |  |  |
|  | T82827A | Fibrosis due to cardiac prosthetic devices, implants and grafts, initial encounter |  |  |  |  |  |  |  |  |  |
|  | T82827D | Fibrosis due to cardiac prosthetic devices, implants and grafts, subsequent encounter |  |  |  |  |  |  |  |  |  |
|  | T82827S | Fibrosis due to cardiac prosthetic devices, implants and grafts, sequela |  |  |  |  |  |  |  |  |  |
|  | T82847A | Pain due to cardiac prosthetic devices, implants and grafts, initial encounter |  |  |  |  |  |  |  |  |  |
|  | T82847D | Pain due to cardiac prosthetic devices, implants and grafts, subsequent encounter |  |  |  |  |  |  |  |  |  |
|  | T82847S | Pain due to cardiac prosthetic devices, implants and grafts, sequela |  |  |  |  |  |  |  |  |  |
|  | T82848A | Pain due to vascular prosthetic devices, implants and grafts, initial encounter |  |  |  |  |  |  |  |  |  |
|  | T82848D | Pain due to vascular prosthetic devices, implants and grafts, subsequent encounter |  |  |  |  |  |  |  |  |  |
|  | T82848S | Pain due to vascular prosthetic devices, implants and grafts, sequela |  |  |  |  |  |  |  |  |  |
|  | T82857A | Stenosis of other cardiac prosthetic devices, implants and grafts, initial encounter |  |  |  |  |  |  |  |  |  |
|  | T82857D | Stenosis of other cardiac prosthetic devices, implants and grafts, subsequent encounter |  |  |  |  |  |  |  |  |  |
|  | T82857S | Stenosis of other cardiac prosthetic devices, implants and grafts, sequela |  |  |  |  |  |  |  |  |  |
|  | T82858A | Stenosis of other vascular prosthetic devices, implants and grafts, initial encounter |  |  |  |  |  |  |  |  |  |
|  | T82858D | Stenosis of other vascular prosthetic devices, implants and grafts, subsequent encounter |  |  |  |  |  |  |  |  |  |
|  | T82858S | Stenosis of other vascular prosthetic devices, implants and grafts, sequela |  |  |  |  |  |  |  |  |  |
|  | T82867A | Thrombosis due to cardiac prosthetic devices, implants and grafts, initial encounter |  |  |  |  |  |  |  |  |  |
|  | T82867D | Thrombosis due to cardiac prosthetic devices, implants and grafts, subsequent encounter |  |  |  |  |  |  |  |  |  |
|  | T82867S | Thrombosis due to cardiac prosthetic devices, implants and grafts, sequela |  |  |  |  |  |  |  |  |  |
|  | T829XXA | Unspecified complication of cardiac and vascular prosthetic device, implant and graft, initial encounter |  |  |  |  |  |  |  |  |  |
|  | T829XXD | Unspecified complication of cardiac and vascular prosthetic device, implant and graft, subsequent encounter |  |  |  |  |  |  |  |  |  |
|  | T829XXS | Unspecified complication of cardiac and vascular prosthetic device, implant and graft, sequela |  |  |  |  |  |  |  |  |  |
|  | T86290 | Cardiac allograft vasculopathy |  |  |  |  |  |  |  |  |  |
|  | V1253 | Personal history of sudden cardiac arrest |  |  |  |  |  |  |  |  |  |
|  | V1741 | Family history of sudden cardiac death (SCD) |  |  |  |  |  |  |  |  |  |
|  | V4500 | Unspecified cardiac device in situ |  |  |  |  |  |  |  |  |  |
|  | V4502 | Automatic implantable cardiac defibrillator in situ |  |  |  |  |  |  |  |  |  |
|  | V4509 | Other specified cardiac device in situ |  |  |  |  |  |  |  |  |  |
|  | V5332 | Fitting and adjustment of automatic implantable cardiac defibrillator |  |  |  |  |  |  |  |  |  |
|  | V5339 | Fitting and adjustment of other cardiac device |  |  |  |  |  |  |  |  |  |
|  | Y840 | Cardiac catheterization as the cause of abnormal reaction of the patient, or of later complication, without mention of misadventure at the time of the procedure |  |  |  |  |  |  |  |  |  |
|  | Z3683 | Encounter for fetal screening for congenital cardiac abnormalities |  |  |  |  |  |  |  |  |  |
|  | Z45010 | Encounter for checking and testing of cardiac pacemaker pulse generator [battery] |  |  |  |  |  |  |  |  |  |
|  | Z4502 | Encounter for adjustment and management of automatic implantable cardiac defibrillator |  |  |  |  |  |  |  |  |  |
|  | Z4509 | Encounter for adjustment and management of other cardiac device |  |  |  |  |  |  |  |  |  |
|  | Z8674 | Personal history of sudden cardiac arrest |  |  |  |  |  |  |  |  |  |
|  | Z950 | Presence of cardiac pacemaker |  |  |  |  |  |  |  |  |  |
|  | Z95810 | Presence of automatic (implantable) cardiac defibrillator |  |  |  |  |  |  |  |  |  |
|  | Z95818 | Presence of other cardiac implants and grafts |  |  |  |  |  |  |  |  |  |
|  | Z959 | Presence of cardiac and vascular implant and graft, unspecified |  |  |  |  |  |  |  |  |  |
|  | I2101 | ST elevation (STEMI) myocardial infarction involving left main coronary artery |  |  |  |  |  |  |  |  |  |
|  | I2102 | ST elevation (STEMI) myocardial infarction involving left anterior descending coronary artery |  |  |  |  |  |  |  |  |  |
|  | I2109 | ST elevation (STEMI) myocardial infarction involving other coronary artery of anterior wall |  |  |  |  |  |  |  |  |  |
|  | I2111 | ST elevation (STEMI) myocardial infarction involving right coronary artery |  |  |  |  |  |  |  |  |  |
|  | I2119 | ST elevation (STEMI) myocardial infarction involving other coronary artery of inferior wall |  |  |  |  |  |  |  |  |  |
|  | I2121 | ST elevation (STEMI) myocardial infarction involving left circumflex coronary artery |  |  |  |  |  |  |  |  |  |
|  | I2129 | ST elevation (STEMI) myocardial infarction involving other sites |  |  |  |  |  |  |  |  |  |
|  | I213 | ST elevation (STEMI) myocardial infarction of unspecified site |  |  |  |  |  |  |  |  |  |
|  | I214 | Non-ST elevation (NSTEMI) myocardial infarction |  |  |  |  |  |  |  |  |  |
|  | I219 | Acute myocardial infarction, unspecified |  |  |  |  |  |  |  |  |  |
|  | I21A1 | Myocardial infarction type 2 |  |  |  |  |  |  |  |  |  |
|  | I21A9 | Other myocardial infarction type |  |  |  |  |  |  |  |  |  |
|  | I220 | Subsequent ST elevation (STEMI) myocardial infarction of anterior wall |  |  |  |  |  |  |  |  |  |
|  | I221 | Subsequent ST elevation (STEMI) myocardial infarction of inferior wall |  |  |  |  |  |  |  |  |  |
|  | I222 | Subsequent non-ST elevation (NSTEMI) myocardial infarction |  |  |  |  |  |  |  |  |  |
|  | I228 | Subsequent ST elevation (STEMI) myocardial infarction of other sites |  |  |  |  |  |  |  |  |  |
|  | I229 | Subsequent ST elevation (STEMI) myocardial infarction of unspecified site |  |  |  |  |  |  |  |  |  |
|  | I230 | Hemopericardium as current complication following acute myocardial infarction |  |  |  |  |  |  |  |  |  |
|  | I231 | Atrial septal defect as current complication following acute myocardial infarction |  |  |  |  |  |  |  |  |  |
|  | I232 | Ventricular septal defect as current complication following acute myocardial infarction |  |  |  |  |  |  |  |  |  |
|  | I233 | Rupture of cardiac wall without hemopericardium as current complication following acute myocardial infarction |  |  |  |  |  |  |  |  |  |
|  | I234 | Rupture of chordae tendineae as current complication following acute myocardial infarction |  |  |  |  |  |  |  |  |  |
|  | I235 | Rupture of papillary muscle as current complication following acute myocardial infarction |  |  |  |  |  |  |  |  |  |
|  | I236 | Thrombosis of atrium, auricular appendage, and ventricle as current complications following acute myocardial infarction |  |  |  |  |  |  |  |  |  |
|  | I237 | Postinfarction angina |  |  |  |  |  |  |  |  |  |
|  | I238 | Other current complications following acute myocardial infarction |  |  |  |  |  |  |  |  |  |
|  | I240 | Acute coronary thrombosis not resulting in myocardial infarction |  |  |  |  |  |  |  |  |  |
|  | I423 | Endomyocardial (eosinophilic) disease |  |  |  |  |  |  |  |  |  |
|  | I514 | Myocarditis, unspecified |  |  |  |  |  |  |  |  |  |
|  | I515 | Myocardial degeneration |  |  |  |  |  |  |  |  |  |
|  | P294 | Transient myocardial ischemia in newborn |  |  |  |  |  |  |  |  |  |
