# Supplementary material for: Relationship between Nonhepatic Serum Ammonia Levels and Sepsis-Associated Encephalopathy: A Retrospective Cohort Study
Source: Emerg Med Int. 2023 Oct 12;2023:6676033. doi: 10.1155/2023/6676033 (PMC10590267; doi:10.1155/2023/6676033)
Supplement: Supplementary Materials — 1: exclude patients with trauma of the skull from the MIMIC IV database according to ICD codes. Supplementary materials 2: exclude patients with intracerebral hemorrhage, cerebral embolism, and ischemic stroke disease from the MIMIC IV database according to ICD codes. Supplementary materials 3: exclude patients with meningitis and encephalitis disease from the MIMIC IV database according to ICD codes. Supplementary materials 4: exclude patients with epilepsy disease from the MIMIC IV database according to ICD codes. Supplementary materials 5: exclude patients with other cerebrovascular disease from the MIMIC IV database according to ICD codes. Supplementary materials 6: exclude patients with mental disorders and neurological disease from the MIMIC IV database according to ICD codes. Supplementary materials 7: exclude patients with alcoholic intoxication or drug abuse from the MIMIC IV database according to ICD codes. Supplementary materials 8: exclude patients with metabolic encephalopathy, hepatic encephalopathy, hypertensive encephalopathy, diabetes with coma, disorders of urea cycle, hypernatremia, and Wernicke's encephalopathy from the MIMIC IV database according to ICD codes. Supplementary materials 9: exclude patients with acute and chronic liver disease. Supplementary materials 10: hypertension disease and ICD codes. Supplementary materials 11: diabetes disease and ICD codes. Supplementary materials 12: lung disease and ICD codes. Supplementary materials 13: cardiovascular diseases and ICD codes. Supplementary materials 14: renal disease from the MIMIC IV database according to ICD codes. Supplementary materials 15: the standardized mean differences of the original cohort were compared with those of the IPW cohorts in sepsis patients. SMD: standardized mean differences. [file 6676033.f1.zip › Supplementary materials.14.docx]

|  | **Supplementary materials.14** Renal disease from the MIMIC IV database according to ICD-codes | | | |
| --- | --- | --- | --- | --- |
|  | | ICD |  | Description |
| 586 | | 9 |  | Renal failure, unspecified |
| 5880 | | 9 |  | Renal osteodystrophy |
| V420 | | 9 |  | Kidney replaced by transplant |
| 5830 | | 9 |  | Nephritis and nephropathy, not specified as acute or chronic, with lesion of proliferative glomerulonephritis |
| 5837 | | 9 |  | Nephritis and nephropathy, not specified as acute or chronic, with lesion of renal medullary necrosis |
| 40301 | | 9 |  | Hypertensive chronic kidney disease, malignant, with chronic kidney disease stage V or end stage renal disease |
| 40311 | | 9 |  | Hypertensive chronic kidney disease, benign, with chronic kidney disease stage V or end stage renal disease |
| 40391 | | 9 |  | Hypertensive chronic kidney disease, unspecified, with chronic kidney disease stage V or end stage renal disease |
| 40402 | | 9 |  | Hypertensive heart and chronic kidney disease, malignant, without heart failure and with chronic kidney disease stage V or end stage renal disease |
| 40403 | | 9 |  | Hypertensive heart and chronic kidney disease, malignant, with heart failure and with chronic kidney disease stage V or end stage renal disease |
| 40412 | | 9 |  | Hypertensive heart and chronic kidney disease, benign, without heart failure and with chronic kidney disease stage V or end stage renal disease |
| 40413 | | 9 |  | Hypertensive heart and chronic kidney disease, benign, with heart failure and chronic kidney disease stage V or end stage renal disease |
| 40492 | | 9 |  | Hypertensive heart and chronic kidney disease, unspecified, without heart failure and with chronic kidney disease stage V or end stage renal disease |
| 40493 | | 9 |  | Hypertensive heart and chronic kidney disease, unspecified, with heart failure and chronic kidney disease stage V or end stage renal disease |
| N19 | | 10 |  | Unspecified kidney failure |
| I120 | | 10 |  | Hypertensive chronic kidney disease with stage 5 chronic kidney disease or end stage renal disease |
| N032 | | 10 |  | Chronic nephritic syndrome with diffuse membranous glomerulonephritis |
| N033 | | 10 |  | Chronic nephritic syndrome with diffuse mesangial proliferative glomerulonephritis |
| N034 | | 10 |  | Chronic nephritic syndrome with diffuse endocapillary proliferative glomerulonephritis |
| N035 | | 10 |  | Chronic nephritic syndrome with diffuse mesangiocapillary glomerulonephritis |
| N036 | | 10 |  | Chronic nephritic syndrome with dense deposit disease |
| N037 | | 10 |  | Chronic nephritic syndrome with diffuse crescentic glomerulonephritis |
| N052 | | 10 |  | Unspecified nephritic syndrome with diffuse membranous glomerulonephritis |
| N053 | | 10 |  | Unspecified nephritic syndrome with diffuse mesangial proliferative glomerulonephritis |
| N054 | | 10 |  | Unspecified nephritic syndrome with diffuse endocapillary proliferative glomerulonephritis |
| N055 | | 10 |  | Unspecified nephritic syndrome with diffuse mesangiocapillary glomerulonephritis |
| N056 | | 10 |  | Unspecified nephritic syndrome with dense deposit disease |
| N057 | | 10 |  | Unspecified nephritic syndrome with diffuse crescentic glomerulonephritis |
| N250 | | 10 |  | Renal osteodystrophy |
| Z940 | | 10 |  | Kidney transplant status |
| Z992 | | 10 |  | Dependence on renal dialysis |
