# Supplementary material for: Relationship between Nonhepatic Serum Ammonia Levels and Sepsis-Associated Encephalopathy: A Retrospective Cohort Study
Source: Emerg Med Int. 2023 Oct 12;2023:6676033. doi: 10.1155/2023/6676033 (PMC10590267; doi:10.1155/2023/6676033)
Supplement: Supplementary Materials — 1: exclude patients with trauma of the skull from the MIMIC IV database according to ICD codes. Supplementary materials 2: exclude patients with intracerebral hemorrhage, cerebral embolism, and ischemic stroke disease from the MIMIC IV database according to ICD codes. Supplementary materials 3: exclude patients with meningitis and encephalitis disease from the MIMIC IV database according to ICD codes. Supplementary materials 4: exclude patients with epilepsy disease from the MIMIC IV database according to ICD codes. Supplementary materials 5: exclude patients with other cerebrovascular disease from the MIMIC IV database according to ICD codes. Supplementary materials 6: exclude patients with mental disorders and neurological disease from the MIMIC IV database according to ICD codes. Supplementary materials 7: exclude patients with alcoholic intoxication or drug abuse from the MIMIC IV database according to ICD codes. Supplementary materials 8: exclude patients with metabolic encephalopathy, hepatic encephalopathy, hypertensive encephalopathy, diabetes with coma, disorders of urea cycle, hypernatremia, and Wernicke's encephalopathy from the MIMIC IV database according to ICD codes. Supplementary materials 9: exclude patients with acute and chronic liver disease. Supplementary materials 10: hypertension disease and ICD codes. Supplementary materials 11: diabetes disease and ICD codes. Supplementary materials 12: lung disease and ICD codes. Supplementary materials 13: cardiovascular diseases and ICD codes. Supplementary materials 14: renal disease from the MIMIC IV database according to ICD codes. Supplementary materials 15: the standardized mean differences of the original cohort were compared with those of the IPW cohorts in sepsis patients. SMD: standardized mean differences. [file 6676033.f1.zip › Supplementary materials.2.docx]

| **Supplementary material 2** Exclude patients with intracerebral hemorrhage, cerebral embolism and ischemic stroke disease from the MIMIC IV database according to ICD-codes | | | | | | | | | | | | | | | | | | | | |
| --- | --- | --- | --- | --- | --- | --- | --- | --- | --- | --- | --- | --- | --- | --- | --- | --- | --- | --- | --- | --- |
| ICD-code | | ICD | Description |  |  |  | |  | |  | |  | |  | |  | |  | | |
| 430 |  | ICD9 | Subarachnoid hemorrhage | | |  |  | |  | |  | |  | |  | |  | |  |  |
| 431 |  | ICD9 | Intracerebral hemorrhage | | |  |  | |  | |  | |  | |  | |  | |  |  |
| 5430 |  | ICD9 | Subarachnoid hemorrhage | | | | | | | | | | | | |  | |  | | |
| 4329 |  | ICD9 | Unspecified intracranial hemorrhage | | | | | | | | | | | | |  | |  | | |
| 4321 |  | ICD9 | Subdural hemorrhage | | | | | | | | | | |  | |  | |  | | |
| 4320 |  | ICD9 | Nontraumatic extradural hemorrhage | | | | | | | | |  | |  | |  | |  | | |
| 7670 |  | ICD9 | Subdural and cerebral hemorrhage | | | | | | | | |  | |  | |  | |  | | |
| 43411 |  | ICD9 | Cerebral embolism with cerebral infarction | | | | | | | | | | | | | | |  | | |
| 4376 |  | ICD9 | Nonpyogenic thrombosis of intracranial venous sinus | | | | | | | | | | | | | | |  | | |
| V1254 |  | ICD9 | Personal history of transient ischemic attack (TIA), and cerebral infarction without residual deficits | | | | | | | | | | | | | | |  | | |
| I6000 |  | ICD10 | Nontraumatic subarachnoid hemorrhage from unspecified carotid siphon and bifurcation | | | | | | | | | | | | | | |  | | |
| I6001 |  | ICD10 | Nontraumatic subarachnoid hemorrhage from right carotid siphon and bifurcation | | | | | | | | | | | | | | |  | | |
| I6002 |  | ICD10 | Nontraumatic subarachnoid hemorrhage from left carotid siphon and bifurcation | | | | | | | | | | | | | | |  | | |
| I6010 |  | ICD10 | Nontraumatic subarachnoid hemorrhage from unspecified middle cerebral artery | | | | | | | | | | | | | | |  | | |
| I6011 |  | ICD10 | Nontraumatic subarachnoid hemorrhage from right middle cerebral artery | | | | | | | | | | | | | | |  | | |
| I6012 |  | ICD10 | Nontraumatic subarachnoid hemorrhage from left middle cerebral artery | | | | | | | | | | | | | | |  | | |
| I602 |  | ICD10 | Nontraumatic subarachnoid hemorrhage from anterior communicating artery | | | | | | | | | | | | | | |  | | |
| I6030 |  | ICD10 | Nontraumatic subarachnoid hemorrhage from unspecified posterior communicating artery | | | | | | | | | | | | | | |  | | |
| I6031 |  | ICD10 | Nontraumatic subarachnoid hemorrhage from right posterior communicating artery | | | | | | | | | | | | | | |  | | |
| I6032 |  | ICD10 | Nontraumatic subarachnoid hemorrhage from left posterior communicating artery | | | | | | | | | | | | | | |  | | |
| I604 |  | ICD10 | Nontraumatic subarachnoid hemorrhage from basilar artery | | | | | | | | | | | | | | |  | | |
| I6050 |  | ICD10 | Nontraumatic subarachnoid hemorrhage from unspecified vertebral artery | | | | | | | | | | | | | | |  | | |
| I6051 |  | ICD10 | Nontraumatic subarachnoid hemorrhage from right vertebral artery | | | | | | | | | | | | | | |  | | |
| I6052 |  | ICD10 | Nontraumatic subarachnoid hemorrhage from left vertebral artery | | | | | | | | | | | | | | |  | | |
| I606 |  | ICD10 | Nontraumatic subarachnoid hemorrhage from other intracranial arteries | | | | | | | | | | | | | | |  | | |
| I607 |  | ICD10 | Nontraumatic subarachnoid hemorrhage from unspecified intracranial artery | | | | | | | | | | | | | | |  | | |
| I608 |  | ICD10 | Other nontraumatic subarachnoid hemorrhage | | | | | | | | | | | | | | |  | | |
| I609 |  | ICD10 | Nontraumatic subarachnoid hemorrhage, unspecified | | | | | | | | | | | | | | |  | | |
| I610 |  | ICD10 | Nontraumatic intracerebral hemorrhage in hemisphere, subcortical | | | | | | | | | | | | | | |  | | |
| I611 |  | ICD10 | Nontraumatic intracerebral hemorrhage in hemisphere, cortical | | | | | | | | | | | | | | |  | | |
| I612 |  | ICD10 | Nontraumatic intracerebral hemorrhage in hemisphere, unspecified | | | | | | | | | | | | | | |  | | |
| I613 |  | ICD10 | Nontraumatic intracerebral hemorrhage in brain stem | | | | | | | | | | | | | | |  | | |
| I614 |  | ICD10 | Nontraumatic intracerebral hemorrhage in cerebellum | | | | | | | | | | | | | | |  | | |
| I615 |  | ICD10 | Nontraumatic intracerebral hemorrhage, intraventricular | | | | | | | | | | | | | | |  | | |
| I616 |  | ICD10 | Nontraumatic intracerebral hemorrhage, multiple localized | | | | | | | | | | | | | | |  | | |
| I618 |  | ICD10 | Other nontraumatic intracerebral hemorrhage | | | | | | | | | | | | | | |  | | |
| I619 |  | ICD10 | Nontraumatic intracerebral hemorrhage, unspecified | | | | | | | | | | | | | | |  | | |
| I6200 |  | ICD10 | Nontraumatic subdural hemorrhage, unspecified | | | | | | | | | | | | | | |  | | |
| I6201 |  | ICD10 | Nontraumatic acute subdural hemorrhage | | | | | | | | | | | | | | |  | | |
| I6202 |  | ICD10 | Nontraumatic subacute subdural hemorrhage | | | | | | | | | | | | | | |  | | |
| I6203 |  | ICD10 | Nontraumatic chronic subdural hemorrhage | | | | | | | | | | | | | | |  | | |
| I621 |  | ICD10 | Nontraumatic extradural hemorrhage | | | | | | | | | | | | | | |  | | |
| I629 |  | ICD10 | Nontraumatic intracranial hemorrhage, unspecified | | | | | | | | | | | | | | |  | | |
| I6300 |  | ICD10 | Cerebral infarction due to thrombosis of unspecified precerebral artery | | | | | | | | | | | | | | |  | | |
| I63011 |  | ICD10 | Cerebral infarction due to thrombosis of right vertebral artery | | | | | | | | | | | | | | |  | | |
| I63012 |  | ICD10 | Cerebral infarction due to thrombosis of left vertebral artery | | | | | | | | | | | | | | |  | | |
| I63013 |  | ICD10 | Cerebral infarction due to thrombosis of bilateral vertebral arteries | | | | | | | | | | | | | | |  | | |
| I63019 |  | ICD10 | Cerebral infarction due to thrombosis of unspecified vertebral artery | | | | | | | | | | | | | | |  | | |
| I6302 |  | ICD10 | Cerebral infarction due to thrombosis of basilar artery | | | | | | | | | | | | | | |  | | |
| I63031 |  | ICD10 | Cerebral infarction due to thrombosis of right carotid artery | | | | | | | | | | | | | | |  | | |
| I63032 |  | ICD10 | Cerebral infarction due to thrombosis of left carotid artery | | | | | | | | | | | | | | |  | | |
| I63033 |  | ICD10 | Cerebral infarction due to thrombosis of bilateral carotid arteries | | | | | | | | | | | | | | |  | | |
| I63039 |  | ICD10 | Cerebral infarction due to thrombosis of unspecified carotid artery | | | | | | | | | | | | | | |  | | |
| I6309 |  | ICD10 | Cerebral infarction due to thrombosis of other precerebral artery | | | | | | | | | | | | | | |  | | |
| I6310 |  | ICD10 | Cerebral infarction due to embolism of unspecified precerebral artery | | | | | | | | | | | | | | |  | | |
| I63111 |  | ICD10 | Cerebral infarction due to embolism of right vertebral artery | | | | | | | | | | | | | | |  | | |
| I63112 |  | ICD10 | Cerebral infarction due to embolism of left vertebral artery | | | | | | | | | | | | | | |  | | |
| I63113 |  | ICD10 | Cerebral infarction due to embolism of bilateral vertebral arteries | | | | | | | | | | | | | | |  | | |
| I63119 |  | ICD10 | Cerebral infarction due to embolism of unspecified vertebral artery | | | | | | | | | | | | | | |  | | |
| I6312 |  | ICD10 | Cerebral infarction due to embolism of basilar artery | | | | | | | | | | | | | | |  | | |
| I63131 |  | ICD10 | Cerebral infarction due to embolism of right carotid artery | | | | | | | | | | | | | | |  | | |
| I63132 |  | ICD10 | Cerebral infarction due to embolism of left carotid artery | | | | | | | | | | | | | | |  | | |
| I63133 |  | ICD10 | Cerebral infarction due to embolism of bilateral carotid arteries | | | | | | | | | | | | | | |  | | |
| I63139 |  | ICD10 | Cerebral infarction due to embolism of unspecified carotid artery | | | | | | | | | | | | | | |  | | |
| I6319 |  | ICD10 | Cerebral infarction due to embolism of other precerebral artery | | | | | | | | | | | | | | |  | | |
| I6320 |  | ICD10 | Cerebral infarction due to unspecified occlusion or stenosis of unspecified precerebral arteries | | | | | | | | | | | | | | |  | | |
| I63211 |  | ICD10 | Cerebral infarction due to unspecified occlusion or stenosis of right vertebral artery | | | | | | | | | | | | | | |  | | |
| I63212 |  | ICD10 | Cerebral infarction due to unspecified occlusion or stenosis of left vertebral artery | | | | | | | | | | | | | | |  | | |
| I63213 |  | ICD10 | Cerebral infarction due to unspecified occlusion or stenosis of bilateral vertebral arteries | | | | | | | | | | | | | | |  | | |
| I63219 |  | ICD10 | Cerebral infarction due to unspecified occlusion or stenosis of unspecified vertebral artery | | | | | | | | | | | | | | |  | | |
| I6322 |  | ICD10 | Cerebral infarction due to unspecified occlusion or stenosis of basilar artery | | | | | | | | | | | | | | |  | | |
| I63231 |  | ICD10 | Cerebral infarction due to unspecified occlusion or stenosis of right carotid arteries | | | | | | | | | | | | | | |  | | |
| I63232 |  | ICD10 | Cerebral infarction due to unspecified occlusion or stenosis of left carotid arteries | | | | | | | | | | | | | | |  | | |
| I63233 |  | ICD10 | Cerebral infarction due to unspecified occlusion or stenosis of bilateral carotid arteries | | | | | | | | | | | | | | |  | | |
| I63239 |  | ICD10 | Cerebral infarction due to unspecified occlusion or stenosis of unspecified carotid artery | | | | | | | | | | | | | | |  | | |
| I6329 |  | ICD10 | Cerebral infarction due to unspecified occlusion or stenosis of other precerebral arteries | | | | | | | | | | | | | | |  | | |
| I6330 |  | ICD10 | Cerebral infarction due to thrombosis of unspecified cerebral artery | | | | | | | | | | | | | | |  | | |
| I63311 |  | ICD10 | Cerebral infarction due to thrombosis of right middle cerebral artery | | | | | | | | | | | | | | |  | | |
| I63312 |  | ICD10 | Cerebral infarction due to thrombosis of left middle cerebral artery | | | | | | | | | | | | | | |  | | |
| I63313 |  | ICD10 | Cerebral infarction due to thrombosis of bilateral middle cerebral arteries | | | | | | | | | | | | | | |  | | |
| I63319 |  | ICD10 | Cerebral infarction due to thrombosis of unspecified middle cerebral artery | | | | | | | | | | | | | | |  | | |
| I63321 |  | ICD10 | Cerebral infarction due to thrombosis of right anterior cerebral artery | | | | | | | | | | | | | | |  | | |
| I63322 |  | ICD10 | Cerebral infarction due to thrombosis of left anterior cerebral artery | | | | | | | | | | | | | | |  | | |
| I63323 |  | ICD10 | Cerebral infarction due to thrombosis of bilateral anterior cerebral arteries | | | | | | | | | | | | | | |  | | |
| I63329 |  | ICD10 | Cerebral infarction due to thrombosis of unspecified anterior cerebral artery | | | | | | | | | | | | | | |  | | |
| I63331 |  | ICD10 | Cerebral infarction due to thrombosis of right posterior cerebral artery | | | | | | | | | | | | | | |  | | |
| I63332 |  | ICD10 | Cerebral infarction due to thrombosis of left posterior cerebral artery | | | | | | | | | | | | | | |  | | |
| I63333 |  | ICD10 | Cerebral infarction due to thrombosis of bilateral posterior cerebral arteries | | | | | | | | | | | | | | |  | | |
| I63339 |  | ICD10 | Cerebral infarction due to thrombosis of unspecified posterior cerebral artery | | | | | | | | | | | | | | |  | | |
| I63341 |  | ICD10 | Cerebral infarction due to thrombosis of right cerebellar artery | | | | | | | | | | | | | | |  | | |
| I63342 |  | ICD10 | Cerebral infarction due to thrombosis of left cerebellar artery | | | | | | | | | | | | | | |  | | |
| I63343 |  | ICD10 | Cerebral infarction due to thrombosis of bilateral cerebellar arteries | | | | | | | | | | | | | | |  | | |
| I63349 |  | ICD10 | Cerebral infarction due to thrombosis of unspecified cerebellar artery | | | | | | | | | | | | | | |  | | |
| I6339 |  | ICD10 | Cerebral infarction due to thrombosis of other cerebral artery | | | | | | | | | | | | | | |  | | |
| I6340 |  | ICD10 | Cerebral infarction due to embolism of unspecified cerebral artery | | | | | | | | | | | | | | |  | | |
| I63411 |  | ICD10 | Cerebral infarction due to embolism of right middle cerebral artery | | | | | | | | | | | | | | |  | | |
| I63412 |  | ICD10 | Cerebral infarction due to embolism of left middle cerebral artery | | | | | | | | | | | | | | |  | | |
| I63413 |  | ICD10 | Cerebral infarction due to embolism of bilateral middle cerebral arteries | | | | | | | | | | | | | | |  | | |
| I63419 |  | ICD10 | Cerebral infarction due to embolism of unspecified middle cerebral artery | | | | | | | | | | | | | | |  | | |
| I63421 |  | ICD10 | Cerebral infarction due to embolism of right anterior cerebral artery | | | | | | | | | | | | | | |  | | |
| I63422 |  | ICD10 | Cerebral infarction due to embolism of left anterior cerebral artery | | | | | | | | | | | | | | |  | | |
| I63423 |  | ICD10 | Cerebral infarction due to embolism of bilateral anterior cerebral arteries | | | | | | | | | | | | | | |  | | |
| I63429 |  | ICD10 | Cerebral infarction due to embolism of unspecified anterior cerebral artery | | | | | | | | | | | | | | |  | | |
| I63431 |  | ICD10 | Cerebral infarction due to embolism of right posterior cerebral artery | | | | | | | | | | | | | | |  | | |
| I63432 |  | ICD10 | Cerebral infarction due to embolism of left posterior cerebral artery | | | | | | | | | | | | | | |  | | |
| I63433 |  | ICD10 | Cerebral infarction due to embolism of bilateral posterior cerebral arteries | | | | | | | | | | | | | | |  | | |
| I63439 |  | ICD10 | Cerebral infarction due to embolism of unspecified posterior cerebral artery | | | | | | | | | | | | | | |  | | |
| I63441 |  | ICD10 | Cerebral infarction due to embolism of right cerebellar artery | | | | | | | | | | | | | | |  | | |
| I63442 |  | ICD10 | Cerebral infarction due to embolism of left cerebellar artery | | | | | | | | | | | | | | |  | | |
| I63443 |  | ICD10 | Cerebral infarction due to embolism of bilateral cerebellar arteries | | | | | | | | | | | | | | |  | | |
| I63449 |  | ICD10 | Cerebral infarction due to embolism of unspecified cerebellar artery | | | | | | | | | | | | | | |  | | |
| I6349 |  | ICD10 | Cerebral infarction due to embolism of other cerebral artery | | | | | | | | | | | | | | |  | | |
| I6350 |  | ICD10 | Cerebral infarction due to unspecified occlusion or stenosis of unspecified cerebral artery | | | | | | | | | | | | | | |  | | |
| I63511 |  | ICD10 | Cerebral infarction due to unspecified occlusion or stenosis of right middle cerebral artery | | | | | | | | | | | | | | |  | | |
| I63512 |  | ICD10 | Cerebral infarction due to unspecified occlusion or stenosis of left middle cerebral artery | | | | | | | | | | | | | | |  | | |
| I63513 |  | ICD10 | Cerebral infarction due to unspecified occlusion or stenosis of bilateral middle cerebral arteries | | | | | | | | | | | | | | |  | | |
| I63519 |  | ICD10 | Cerebral infarction due to unspecified occlusion or stenosis of unspecified middle cerebral artery | | | | | | | | | | | | | | |  | | |
| I63521 |  | ICD10 | Cerebral infarction due to unspecified occlusion or stenosis of right anterior cerebral artery | | | | | | | | | | | | | | |  | | |
| I63522 |  | ICD10 | Cerebral infarction due to unspecified occlusion or stenosis of left anterior cerebral artery | | | | | | | | | | | | | | |  | | |
| I63523 |  | ICD10 | Cerebral infarction due to unspecified occlusion or stenosis of bilateral anterior cerebral arteries | | | | | | | | | | | | | | |  | | |
| I63529 |  | ICD10 | Cerebral infarction due to unspecified occlusion or stenosis of unspecified anterior cerebral artery | | | | | | | | | | | | | | |  | | |
| I63531 |  | ICD10 | Cerebral infarction due to unspecified occlusion or stenosis of right posterior cerebral artery | | | | | | | | | | | | | | |  | | |
| I63532 |  | ICD10 | Cerebral infarction due to unspecified occlusion or stenosis of left posterior cerebral artery | | | | | | | | | | | | | | |  | | |
| I63533 |  | ICD10 | Cerebral infarction due to unspecified occlusion or stenosis of bilateral posterior cerebral arteries | | | | | | | | | | | | | | |  | | |
| I63539 |  | ICD10 | Cerebral infarction due to unspecified occlusion or stenosis of unspecified posterior cerebral artery | | | | | | | | | | | | | | |  | | |
| I63541 |  | ICD10 | Cerebral infarction due to unspecified occlusion or stenosis of right cerebellar artery | | | | | | | | | | | | | | |  | | |
| I63542 |  | ICD10 | Cerebral infarction due to unspecified occlusion or stenosis of left cerebellar artery | | | | | | | | | | | | | | |  | | |
| I63543 |  | ICD10 | Cerebral infarction due to unspecified occlusion or stenosis of bilateral cerebellar arteries | | | | | | | | | | | | | | |  | | |
| I63549 |  | ICD10 | Cerebral infarction due to unspecified occlusion or stenosis of unspecified cerebellar artery | | | | | | | | | | | | | | |  | | |
| I6359 |  | ICD10 | Cerebral infarction due to unspecified occlusion or stenosis of other cerebral artery | | | | | | | | | | | | | | |  | | |
| I636 |  | ICD10 | Cerebral infarction due to cerebral venous thrombosis, nonpyogenic | | | | | | | | | | | | | | |  | | |
| I6381 |  | ICD10 | Other cerebral infarction due to occlusion or stenosis of small artery | | | | | | | | | | | | | | |  | | |
| I6389 |  | ICD10 | Other cerebral infarction | | | | | | | | | | | | | | |  | | |
| I639 |  | ICD10 | Cerebral infarction, unspecified | | | | | | | | | | | | | | |  | | |
| I6501 |  | ICD10 | Occlusion and stenosis of right vertebral artery | | | | | | | | | | | | | | |  | | |
| I6502 |  | ICD10 | Occlusion and stenosis of left vertebral artery | | | | | | | | | | | | | | |  | | |
| I6503 |  | ICD10 | Occlusion and stenosis of bilateral vertebral arteries | | | | | | | | | | | | | | |  | | |
| I6509 |  | ICD10 | Occlusion and stenosis of unspecified vertebral artery | | | | | | | | | | | | | | |  | | |
| I651 |  | ICD10 | Occlusion and stenosis of basilar artery | | | | | | | | | | | | | | |  | | |
| I6521 |  | ICD10 | Occlusion and stenosis of right carotid artery | | | | | | | | | | | | | | |  | | |
| I6522 |  | ICD10 | Occlusion and stenosis of left carotid artery | | | | | | | | | | | | | | |  | | |
| I6523 |  | ICD10 | Occlusion and stenosis of bilateral carotid arteries | | | | | | | | | | | | | | |  | | |
| I6529 |  | ICD10 | Occlusion and stenosis of unspecified carotid artery | | | | | | | | | | | | | | |  | | |
| I658 |  | ICD10 | Occlusion and stenosis of other precerebral arteries | | | | | | | | | | | | | | |  | | |
| I659 |  | ICD10 | Occlusion and stenosis of unspecified precerebral artery | | | | | | | | | | | | | | |  | | |
| I6601 |  | ICD10 | Occlusion and stenosis of right middle cerebral artery | | | | | | | | | | | | | | |  | | |
| I6602 |  | ICD10 | Occlusion and stenosis of left middle cerebral artery | | | | | | | | | | | | | | |  | | |
| I6603 |  | ICD10 | Occlusion and stenosis of bilateral middle cerebral arteries | | | | | | | | | | | | | | |  | | |
| I6609 |  | ICD10 | Occlusion and stenosis of unspecified middle cerebral artery | | | | | | | | | | | | | | |  | | |
| I6611 |  | ICD10 | Occlusion and stenosis of right anterior cerebral artery | | | | | | | | | | | | | | |  | | |
| I6612 |  | ICD10 | Occlusion and stenosis of left anterior cerebral artery | | | | | | | | | | | | | | |  | | |
| I6613 |  | ICD10 | Occlusion and stenosis of bilateral anterior cerebral arteries | | | | | | | | | | | | | | |  | | |
| I6619 |  | ICD10 | Occlusion and stenosis of unspecified anterior cerebral artery | | | | | | | | | | | | | | |  | | |
| I6621 |  | ICD10 | Occlusion and stenosis of right posterior cerebral artery | | | | | | | | | | | | | | |  | | |
| I6622 |  | ICD10 | Occlusion and stenosis of left posterior cerebral artery | | | | | | | | | | | | | | |  | | |
| I6623 |  | ICD10 | Occlusion and stenosis of bilateral posterior cerebral arteries | | | | | | | | | | | | | | |  | | |
| I6629 |  | ICD10 | Occlusion and stenosis of unspecified posterior cerebral artery | | | | | | | | | | | | | | |  | | |
| I663 |  | ICD10 | Occlusion and stenosis of cerebellar arteries | | | | | | | | | | | | | | |  | | |
| I668 |  | ICD10 | Occlusion and stenosis of other cerebral arteries | | | | | | | | | | | | | | |  | | |
| I669 |  | ICD10 | Occlusion and stenosis of unspecified cerebral artery | | | | | | | | | | | | | | |  | | |
| I670 |  | ICD10 | Dissection of cerebral arteries, nonruptured | | | | | | | | | | | | | | |  | | |
| I676 |  | ICD10 | Nonpyogenic thrombosis of intracranial venous system | | | | | | | | | | | | | | |  | | |
| I677 |  | ICD10 | Cerebral arteritis, not elsewhere classified | | | | | | | | | | | | | | |  | | |
| I6781 |  | ICD10 | Acute cerebrovascular insufficiency | | | | | | | | | | | | | | |  | | |
| I6782 |  | ICD10 | Cerebral ischemia | | | | | | | | | | | | | | |  | | |
| I6783 |  | ICD10 | Posterior reversible encephalopathy syndrome | | | | | | | | | | | | | | |  | | |
| I67841 |  | ICD10 | Reversible cerebrovascular vasoconstriction syndrome | | | | | | | | | | | | | | |  | | |
| I67848 |  | ICD10 | Other cerebrovascular vasospasm and vasoconstriction | | | | | | | | | | | | | | |  | | |
| I67850 |  | ICD10 | Cerebral autosomal dominant arteriopathy with subcortical infarcts and leukoencephalopathy | | | | | | | | | | | | | | |  | | |
| I67858 |  | ICD10 | Other hereditary cerebrovascular disease | | | | | | | | | | | | | | |  | | |
| I6789 |  | ICD10 | Other cerebrovascular disease | | | | | | | | | | | | | | |  | | |
| I679 |  | ICD10 | Cerebrovascular disease, unspecified | | | | | | | | | | | | | | |  | | |
| I680 |  | ICD10 | Cerebral amyloid angiopathy | | | | | | | | | | | | | | |  | | |
| I682 |  | ICD10 | Cerebral arteritis in other diseases classified elsewhere | | | | | | | | | | | | | | |  | | |
| I688 |  | ICD10 | Other cerebrovascular disorders in diseases classified elsewhere | | | | | | | | | | | | | | |  | | |
| I6900 |  | ICD10 | Unspecified sequelae of nontraumatic subarachnoid hemorrhage | | | | | | | | | | | | | | |  | | |
| I69010 |  | ICD10 | Attention and concentration deficit following nontraumatic subarachnoid hemorrhage | | | | | | | | | | | | | | |  | | |
| I69011 |  | ICD10 | Memory deficit following nontraumatic subarachnoid hemorrhage | | | | | | | | | | | | | | |  | | |
| I69012 |  | ICD10 | Visuospatial deficit and spatial neglect following nontraumatic subarachnoid hemorrhage | | | | | | | | | | | | | | |  | | |
| I69013 |  | ICD10 | Psychomotor deficit following nontraumatic subarachnoid hemorrhage | | | | | | | | | | | | | | |  | | |
| I69014 |  | ICD10 | Frontal lobe and executive function deficit following nontraumatic subarachnoid hemorrhage | | | | | | | | | | | | | | |  | | |
| I69015 |  | ICD10 | Cognitive social or emotional deficit following nontraumatic subarachnoid hemorrhage | | | | | | | | | | | | | | |  | | |
| I69018 |  | ICD10 | Other symptoms and signs involving cognitive functions following nontraumatic subarachnoid hemorrhage | | | | | | | | | | | | | | |  | | |
| I69019 |  | ICD10 | Unspecified symptoms and signs involving cognitive functions following nontraumatic subarachnoid hemorrhage | | | | | | | | | | | | | | |  | | |
| I69020 |  | ICD10 | Aphasia following nontraumatic subarachnoid hemorrhage | | | | | | | | | | | | | | |  | | |
| I69021 |  | ICD10 | Dysphasia following nontraumatic subarachnoid hemorrhage | | | | | | | | | | | | | | |  | | |
| I69022 |  | ICD10 | Dysarthria following nontraumatic subarachnoid hemorrhage | | | | | | | | | | | | | | |  | | |
| I69023 |  | ICD10 | Fluency disorder following nontraumatic subarachnoid hemorrhage | | | | | | | | | | | | | | |  | | |
| I69028 |  | ICD10 | Other speech and language deficits following nontraumatic subarachnoid hemorrhage | | | | | | | | | | | | | | |  | | |
| I69031 |  | ICD10 | Monoplegia of upper limb following nontraumatic subarachnoid hemorrhage affecting right dominant side | | | | | | | | | | | | | | |  | | |
| I69032 |  | ICD10 | Monoplegia of upper limb following nontraumatic subarachnoid hemorrhage affecting left dominant side | | | | | | | | | | | | | | |  | | |
| I69033 |  | ICD10 | Monoplegia of upper limb following nontraumatic subarachnoid hemorrhage affecting right non-dominant side | | | | | | | | | | | | | | |  | | |
| I69034 |  | ICD10 | Monoplegia of upper limb following nontraumatic subarachnoid hemorrhage affecting left non-dominant side | | | | | | | | | | | | | | |  | | |
| I69039 |  | ICD10 | Monoplegia of upper limb following nontraumatic subarachnoid hemorrhage affecting unspecified side | | | | | | | | | | | | | | |  | | |
| I69041 |  | ICD10 | Monoplegia of lower limb following nontraumatic subarachnoid hemorrhage affecting right dominant side | | | | | | | | | | | | | | |  | | |
| I69042 |  | ICD10 | Monoplegia of lower limb following nontraumatic subarachnoid hemorrhage affecting left dominant side | | | | | | | | | | | | | | |  | | |
| I69043 |  | ICD10 | Monoplegia of lower limb following nontraumatic subarachnoid hemorrhage affecting right non-dominant side | | | | | | | | | | | | | | |  | | |
| I69044 |  | ICD10 | Monoplegia of lower limb following nontraumatic subarachnoid hemorrhage affecting left non-dominant side | | | | | | | | | | | | | | |  | | |
| I69049 |  | ICD10 | Monoplegia of lower limb following nontraumatic subarachnoid hemorrhage affecting unspecified side | | | | | | | | | | | | | | |  | | |
| I69051 |  | ICD10 | Hemiplegia and hemiparesis following nontraumatic subarachnoid hemorrhage affecting right dominant side | | | | | | | | | | | | | | |  | | |
| I69052 |  | ICD10 | Hemiplegia and hemiparesis following nontraumatic subarachnoid hemorrhage affecting left dominant side | | | | | | | | | | | | | | |  | | |
| I69053 |  | ICD10 | Hemiplegia and hemiparesis following nontraumatic subarachnoid hemorrhage affecting right non-dominant side | | | | | | | | | | | | | | |  | | |
| I69054 |  | ICD10 | Hemiplegia and hemiparesis following nontraumatic subarachnoid hemorrhage affecting left non-dominant side | | | | | | | | | | | | | | |  | | |
| I69059 |  | ICD10 | Hemiplegia and hemiparesis following nontraumatic subarachnoid hemorrhage affecting unspecified side | | | | | | | | | | | | | | |  | | |
| I69061 |  | ICD10 | Other paralytic syndrome following nontraumatic subarachnoid hemorrhage affecting right dominant side | | | | | | | | | | | | | | |  | | |
| I69062 |  | ICD10 | Other paralytic syndrome following nontraumatic subarachnoid hemorrhage affecting left dominant side | | | | | | | | | | | | | | |  | | |
| I69063 |  | ICD10 | Other paralytic syndrome following nontraumatic subarachnoid hemorrhage affecting right non-dominant side | | | | | | | | | | | | | | |  | | |
| I69064 |  | ICD10 | Other paralytic syndrome following nontraumatic subarachnoid hemorrhage affecting left non-dominant side | | | | | | | | | | | | | | |  | | |
| I69065 |  | ICD10 | Other paralytic syndrome following nontraumatic subarachnoid hemorrhage, bilateral | | | | | | | | | | | | | | |  | | |
| I69069 |  | ICD10 | Other paralytic syndrome following nontraumatic subarachnoid hemorrhage affecting unspecified side | | | | | | | | | | | | | | |  | | |
| I69090 |  | ICD10 | Apraxia following nontraumatic subarachnoid hemorrhage | | | | | | | | | | | | | | |  | | |
| I69091 |  | ICD10 | Dysphagia following nontraumatic subarachnoid hemorrhage | | | | | | | | | | | | | | |  | | |
| I69092 |  | ICD10 | Facial weakness following nontraumatic subarachnoid hemorrhage | | | | | | | | | | | | | | |  | | |
| I69093 |  | ICD10 | Ataxia following nontraumatic subarachnoid hemorrhage | | | | | | | | | | | | | | |  | | |
| I69098 |  | ICD10 | Other sequelae following nontraumatic subarachnoid hemorrhage | | | | | | | | | | | | | | |  | | |
| I6910 |  | ICD10 | Unspecified sequelae of nontraumatic intracerebral hemorrhage | | | | | | | | | | | | | | |  | | |
| I69110 |  | ICD10 | Attention and concentration deficit following nontraumatic intracerebral hemorrhage | | | | | | | | | | | | | | |  | | |
| I69111 |  | ICD10 | Memory deficit following nontraumatic intracerebral hemorrhage | | | | | | | | | | | | | | |  | | |
| I69112 |  | ICD10 | Visuospatial deficit and spatial neglect following nontraumatic intracerebral hemorrhage | | | | | | | | | | | | | | |  | | |
| I69113 |  | ICD10 | Psychomotor deficit following nontraumatic intracerebral hemorrhage | | | | | | | | | | | | | | |  | | |
| I69114 |  | ICD10 | Frontal lobe and executive function deficit following nontraumatic intracerebral hemorrhage | | | | | | | | | | | | | | |  | | |
| I69115 |  | ICD10 | Cognitive social or emotional deficit following nontraumatic intracerebral hemorrhage | | | | | | | | | | | | | | |  | | |
| I69118 |  | ICD10 | Other symptoms and signs involving cognitive functions following nontraumatic intracerebral hemorrhage | | | | | | | | | | | | | | |  | | |
| I69119 |  | ICD10 | Unspecified symptoms and signs involving cognitive functions following nontraumatic intracerebral hemorrhage | | | | | | | | | | | | | | |  | | |
| I69120 |  | ICD10 | Aphasia following nontraumatic intracerebral hemorrhage | | | | | | | | | | | | | | |  | | |
| I69121 |  | ICD10 | Dysphasia following nontraumatic intracerebral hemorrhage | | | | | | | | | | | | | | |  | | |
| I69122 |  | ICD10 | Dysarthria following nontraumatic intracerebral hemorrhage | | | | | | | | | | | | | | |  | | |
| I69123 |  | ICD10 | Fluency disorder following nontraumatic intracerebral hemorrhage | | | | | | | | | | | | | | |  | | |
| I69128 |  | ICD10 | Other speech and language deficits following nontraumatic intracerebral hemorrhage | | | | | | | | | | | | | | |  | | |
| I69311 |  | ICD10 | Memory deficit following cerebral infarction | | | | | | | | | | | | | | |  | | |
| I69312 |  | ICD10 | Visuospatial deficit and spatial neglect following cerebral infarction | | | | | | | | | | | | | | |  | | |
| I69313 |  | ICD10 | Psychomotor deficit following cerebral infarction | | | | | | | | | | | | | | |  | | |
| I69314 |  | ICD10 | Frontal lobe and executive function deficit following cerebral infarction | | | | | | | | | | | | | | |  | | |
| I69315 |  | ICD10 | Cognitive social or emotional deficit following cerebral infarction | | | | | | | | | | | | | | |  | | |
| I69318 |  | ICD10 | Other symptoms and signs involving cognitive functions following cerebral infarction | | | | | | | | | | | | | | |  | | |
| I69319 |  | ICD10 | Unspecified symptoms and signs involving cognitive functions following cerebral infarction | | | | | | | | | | | | | | |  | | |
|  |  |  |  | | | | | | | | | | | | | | |  | | |
