# Supplementary material for: Relationship between Nonhepatic Serum Ammonia Levels and Sepsis-Associated Encephalopathy: A Retrospective Cohort Study
Source: Emerg Med Int. 2023 Oct 12;2023:6676033. doi: 10.1155/2023/6676033 (PMC10590267; doi:10.1155/2023/6676033)
Supplement: Supplementary Materials — 1: exclude patients with trauma of the skull from the MIMIC IV database according to ICD codes. Supplementary materials 2: exclude patients with intracerebral hemorrhage, cerebral embolism, and ischemic stroke disease from the MIMIC IV database according to ICD codes. Supplementary materials 3: exclude patients with meningitis and encephalitis disease from the MIMIC IV database according to ICD codes. Supplementary materials 4: exclude patients with epilepsy disease from the MIMIC IV database according to ICD codes. Supplementary materials 5: exclude patients with other cerebrovascular disease from the MIMIC IV database according to ICD codes. Supplementary materials 6: exclude patients with mental disorders and neurological disease from the MIMIC IV database according to ICD codes. Supplementary materials 7: exclude patients with alcoholic intoxication or drug abuse from the MIMIC IV database according to ICD codes. Supplementary materials 8: exclude patients with metabolic encephalopathy, hepatic encephalopathy, hypertensive encephalopathy, diabetes with coma, disorders of urea cycle, hypernatremia, and Wernicke's encephalopathy from the MIMIC IV database according to ICD codes. Supplementary materials 9: exclude patients with acute and chronic liver disease. Supplementary materials 10: hypertension disease and ICD codes. Supplementary materials 11: diabetes disease and ICD codes. Supplementary materials 12: lung disease and ICD codes. Supplementary materials 13: cardiovascular diseases and ICD codes. Supplementary materials 14: renal disease from the MIMIC IV database according to ICD codes. Supplementary materials 15: the standardized mean differences of the original cohort were compared with those of the IPW cohorts in sepsis patients. SMD: standardized mean differences. [file 6676033.f1.zip › Supplementary materials.3.docx]

|  | **Supplementary materials.3**  Exclude patients with meningitis and encephalitis disease from the MIMIC IV database according to ICD-codes | | | | | | | | | | | | | | | |  |  |
| --- | --- | --- | --- | --- | --- | --- | --- | --- | --- | --- | --- | --- | --- | --- | --- | --- | --- | --- |
|  | ICD-code ICD | | | | Description |  |  |  |  |  |  |  | |  | |  | | |
| 1300 | | ICD9 |  | Tuberculous meningitis, unspecified | | | | | | | | |  | |  | | |  |
| 1301 | | ICD9 |  | Tuberculous meningitis, bacteriological or histological examination not done | | | | | | | | |  | |  | | |  |
| 468 | | ICD9 |  | Other specified slow virus infection of central nervous system | | | | | | | | |  | |  | | |  |
| 469 | | ICD9 |  | Unspecified slow virus infection of central nervous system | | | | | | | | |  | |  | | |  |
| 470 | | ICD9 |  | Meningitis due to coxsackie virus | | | | | | | | |  | |  | | |  |
| 471 | | ICD9 |  | Meningitis due to echo virus | | | | | | | | |  | |  | | |  |
| 478 | | ICD9 |  | Other specified viral meningitis | | | | | | | | |  | |  | | |  |
| 479 | | ICD9 |  | Unspecified viral meningitis | | | | | | | | |  | |  | | |  |
| 491 | | ICD9 |  | Meningitis due to adenovirus | | | | | | | | |  | |  | | |  |
| 498 | | ICD9 |  | Other specified non-arthropod-borne viral diseases of central nervous system | | | | | | | | |  | |  | | |  |
| 499 | | ICD9 |  | Unspecified non-arthropod-borne viral diseases of central nervous system | | | | | | | | |  | |  | | |  |
| 520 | | ICD9 |  | Postvaricella encephalitis | | | | | | | | |  | |  | | |  |
| 530 | | ICD9 |  | Herpes zoster with meningitis | | | | | | | | |  | |  | | |  |
| 5319 | | ICD9 |  | Herpes zoster with other nervous system complications | | | | | | | | |  | |  | | |  |
| 5379 | | ICD9 |  | Herpes zoster with other specified complications | | | | | | | | |  | |  | | |  |
| 5472 | | ICD9 |  | Herpes simplex meningitis | | | | | | | | |  | |  | | |  |
| 550 | | ICD9 |  | Postmeasles encephalitis | | | | | | | | |  | |  | | |  |
| 360 | | ICD9 |  | Meningococcal meningitis | | | | | | | | |  | |  | | |  |
| 361 | | ICD9 |  | Meningococcal encephalitis | | | | | | | | |  | |  | | |  |
| 362 | | ICD9 |  | Meningococcemia | | | | | | | | |  | |  | | |  |
| 363 | | ICD9 |  | Waterhouse-Friderichsen syndrome, meningococcal | | | | | | | | |  | |  | | |  |
| 1302 | | ICD9 |  | Tuberculous meningitis, bacteriological or histological examination unknown (at present) | | | | | | | | |  | |  | | |  |
| 1303 | | ICD9 |  | Tuberculous meningitis, tubercle bacilli found (in sputum) by microscopy | | | | | | | | |  | |  | | |  |
| 1304 | | ICD9 |  | Tuberculous meningitis, tubercle bacilli not found (in sputum) by microscopy, but found by bacterial culture | | | | | | | | |  | |  | | |  |
| 1305 | | ICD9 |  | Tuberculous meningitis, tubercle bacilli not found by bacteriological examination, but tuberculosis confirmed histologically | | | | | | | | |  | |  | | |  |
| 1306 | | ICD9 |  | Tuberculous meningitis, tubercle bacilli not found by bacteriological or histological examination, but tuberculosis confirmed by other methods [inoculation of animals] | | | | | | | | |  | |  | | |  |
| 1310 | | ICD9 |  | Tuberculoma of meninges, unspecified | | | | | | | | |  | |  | | |  |
| 1311 | | ICD9 |  | Tuberculoma of meninges, bacteriological or histological examination not done | | | | | | | | |  | |  | | |  |
| 1312 | | ICD9 |  | Tuberculoma of meninges, bacteriological or histological examination unknown (at present) | | | | | | | | |  | |  | | |  |
| 1313 | | ICD9 |  | Tuberculoma of meninges, tubercle bacilli found (in sputum) by microscopy | | | | | | | | |  | |  | | |  |
| 1314 | | ICD9 |  | Tuberculoma of meninges, tubercle bacilli not found (in sputum) by microscopy, but found by bacterial culture | | | | | | | | |  | |  | | |  |
| 1315 | | ICD9 |  | Tuberculoma of meninges, tubercle bacilli not found by bacteriological examination, but tuberculosis confirmed histologically | | | | | | | | |  | |  | | |  |
| 1316 | | ICD9 |  | Tuberculoma of meninges, tubercle bacilli not found by bacteriological or histological examination, but tuberculosis confirmed by other methods [inoculation of animals] | | | | | | | | |  | |  | | |  |
| 1321 | | ICD9 |  | Tuberculoma of brain, bacteriological or histological examination not done | | | | | | | | |  | |  | | |  |
| 1322 | | ICD9 |  | Tuberculoma of brain, bacteriological or histological examination unknown (at present) | | | | | | | | |  | |  | | |  |
| 1323 | | ICD9 |  | Tuberculoma of brain, tubercle bacilli found (in sputum) by microscopy | | | | | | | | |  | |  | | |  |
| 1324 | | ICD9 |  | Tuberculoma of brain, tubercle bacilli not found (in sputum) by microscopy, but found by bacterial culture | | | | | | | | |  | |  | | |  |
| 1326 | | ICD9 |  | Tuberculoma of brain, tubercle bacilli not found by bacteriological or histological examination, but tuberculosis confirmed by other | | | | | | | | |  | |  | | |  |
| 1331 | | ICD9 |  | Tuberculous abscess of brain, bacteriological or histological examination not done | | | | | | | | |  | |  | | |  |
| 1332 | | ICD9 |  | Tuberculous abscess of brain, bacteriological or histological examination unknown (at present) | | | | | | | | |  | |  | | |  |
| 1333 | | ICD9 |  | Tuberculous abscess of brain, tubercle bacilli found (in sputum) by microscopy | | | | | | | | |  | |  | | |  |
| 1334 | | ICD9 |  | Tuberculous abscess of brain, tubercle bacilli not found (in sputum) by microscopy, but found by bacterial culture | | | | | | | | |  | |  | | |  |
| 1335 | | ICD9 |  | Tuberculous abscess of brain, tubercle bacilli not found by bacteriological examination, but tuberculosis confirmed histologically | | | | | | | | |  | |  | | |  |
| 1336 | | ICD9 |  | Tuberculous abscess of brain, tubercle bacilli not found by bacteriological or histological examination, but tuberculosis confirmed by other methods [inoculation of animals] | | | | | | | | |  | |  | | |  |
| 1360 | | ICD9 |  | Tuberculous encephalitis or myelitis, unspecified | | | | | | | | |  | |  | | |  |
| 1361 | | ICD9 |  | Tuberculous encephalitis or myelitis, bacteriological or histological examination not done | | | | | | | | |  | |  | | |  |
| 1362 | | ICD9 |  | Tuberculous encephalitis or myelitis, bacteriological or histological examination unknown (at present) | | | | | | | | |  | |  | | |  |
| 1363 | | ICD9 |  | Tuberculous encephalitis or myelitis, tubercle bacilli found (in sputum) by microscopy | | | | | | | | |  | |  | | |  |
| 1364 | | ICD9 |  | Tuberculous encephalitis or myelitis, tubercle bacilli not found (in sputum) by microscopy, but found by bacterial culture | | | | | | | | |  | |  | | |  |
| 1365 | | ICD9 |  | Tuberculous encephalitis or myelitis, tubercle bacilli not found by bacteriological examination, but tuberculosis confirmed histologically | | | | | | | | |  | |  | | |  |
| 1366 | | ICD9 |  | Tuberculous encephalitis or myelitis, tubercle bacilli not found by bacteriological or histological examination, but tuberculosis confirmed by other methods [inoculation of animals] | | | | | | | | |  | |  | | |  |
| 1380 | | ICD9 |  | Other specified tuberculosis of central nervous system, unspecified | | | | | | | | |  | |  | | |  |
| 1381 | | ICD9 |  | Other specified tuberculosis of central nervous system, bacteriological or histological examination not done | | | | | | | | |  | |  | | |  |
| 1382 | | ICD9 |  | Other specified tuberculosis of central nervous system, bacteriological or histological examination unknown (at present) | | | | | | | | |  | |  | | |  |
| 1383 | | ICD9 |  | Other specified tuberculosis of central nervous system, tubercle bacilli found (in sputum) by microscopy | | | | | | | | |  | |  | | |  |
| 1384 | | ICD9 |  | Other specified tuberculosis of central nervous system, tubercle bacilli not found (in sputum) by microscopy, but found by bacterial culture | | | | | | | | |  | |  | | |  |
| 1385 | | ICD9 |  | Other specified tuberculosis of central nervous system, tubercle bacilli not found by bacteriological examination, but tuberculosis confirmed histologically | | | | | | | | |  | |  | | |  |
| 1386 | | ICD9 |  | Other specified tuberculosis of central nervous system, tubercle bacilli not found by bacteriological or histological examination, but tuberculosis confirmed by other methods [inoculation of animals] | | | | | | | | |  | |  | | |  |
| 1390 1 | | ICD9 |  | Unspecified tuberculosis of central nervous system, unspecified | | | | | | | | |  | |  | | |  |
| 1391 | | ICD9 |  | Unspecified tuberculosis of central nervous system, bacteriological or histological examination not done | | | | | | | | |  | |  | | |  |
| 1392 | | ICD9 |  | Unspecified tuberculosis of central nervous system, bacteriological or histological examination unknown (at present) | | | | | | | | |  | |  | | |  |
| 1393 | | ICD9 |  | Unspecified tuberculosis of central nervous system, tubercle bacilli found (in sputum) by microscopy | | | | | | | | |  | |  | | |  |
| 1394 | | ICD9 |  | Unspecified tuberculosis of central nervous system, tubercle bacilli not found (in sputum) by microscopy, but found by bacterial culture | | | | | | | | |  | |  | | |  |
| 1395 | | ICD9 |  | Unspecified tuberculosis of central nervous system, tubercle bacilli not found by bacteriological examination, but tuberculosis confirmed histologically | | | | | | | | |  | |  | | |  |
| 1396 | | ICD9 |  | Unspecified tuberculosis of central nervous system, tubercle bacilli not found by bacteriological or histological examination, but tuberculosis confirmed by other methods [inoculation of animals] | | | | | | | | |  | |  | | |  |
| 1142 | | ICD9 |  | Coccidioidal meningitis | | | | | | | | |  | |  | | |  |
| 621 | | ICD9 |  | Western equine encephalitis | | | | | | | | |  | |  | | |  |
| 622 | | ICD9 |  | Eastern equine encephalitis | | | | | | | | |  | |  | | |  |
| 623 | | ICD9 |  | St. Louis encephalitis | | | | | | | | |  | |  | | |  |
| 624 | | ICD9 |  | Australian encephalitis | | | | | | | | |  | |  | | |  |
| 625 | | ICD9 |  | California virus encephalitis | | | | | | | | |  | |  | | |  |
| 632 | | ICD9 |  | Central european encephalitis | | | | | | | | |  | |  | | |  |
| 638 | | ICD9 |  | Other specified tick-borne viral encephalitis | | | | | | | | |  | |  | | |  |
| 3222 | | ICD9 |  | Chronic meningitis | | | | | | | | |  | |  | | |  |
| 3212 | | ICD9 |  | Meningitis due to viruses not elsewhere classified | | | | | | | | |  | |  | | |  |
| 3201 | | ICD9 |  | Pneumococcal meningitis | | | | | | | | |  | |  | | |  |
| A1782 | | ICD10 |  | Tuberculous meningoencephalitis | | | | | | | | |  | |  | | |  |
| A3212 | | ICD10 |  | Listerial meningoencephalitis | | | | | | | | |  | |  | | |  |
| A3981 | | ICD10 |  | Meningococcal encephalitis | | | | | | | | |  | |  | | |  |
| A4282 | | ICD10 |  | Actinomycotic encephalitis | | | | | | | | |  | |  | | |  |
| A5042 | | ICD10 |  | Late congenital syphilitic encephalitis | | | | | | | | |  | |  | | |  |
| A5214 | | ICD10 |  | Late syphilitic encephalitis | | | | | | | | |  | |  | | |  |
| A811 | | ICD10 |  | Subacute sclerosing panencephalitis | | | | | | | | |  | |  | | |  |
| A830 | | ICD10 |  | Japanese encephalitis | | | | | | | | |  | |  | | |  |
| A831 | | ICD10 |  | Western equine encephalitis | | | | | | | | |  | |  | | |  |
| A832 | | ICD10 |  | Eastern equine encephalitis | | | | | | | | |  | |  | | |  |
| A833 | | ICD10 |  | St Louis encephalitis | | | | | | | | |  | |  | | |  |
| A834 | | ICD10 |  | Australian encephalitis | | | | | | | | |  | |  | | |  |
| A835 | | ICD10 |  | California encephalitis | | | | | | | | |  | |  | | |  |
| A838 | | ICD10 |  | Other mosquito-borne viral encephalitis | | | | | | | | |  | |  | | |  |
| A839 | | ICD10 |  | Mosquito-borne viral encephalitis, unspecified | | | | | | | | |  | |  | | |  |
| A840 | | ICD10 |  | Far Eastern tick-borne encephalitis [Russian spring-summer encephalitis] | | | | | | | | |  | |  | | |  |
| A841 | | ICD10 |  | Central European tick-borne encephalitis | | | | | | | | |  | |  | | |  |
| A848 | | ICD10 |  | Other tick-borne viral encephalitis | | | | | | | | |  | |  | | |  |
| A849 | | ICD10 |  | Tick-borne viral encephalitis, unspecified | | | | | | | | |  | |  | | |  |
| A850 | | ICD10 |  | Enteroviral encephalitis | | | | | | | | |  | |  | | |  |
| A851 | | ICD10 |  | Adenoviral encephalitis | | | | | | | | |  | |  | | |  |
| A852 | | ICD10 |  | Arthropod-borne viral encephalitis, unspecified | | | | | | | | |  | |  | | |  |
| A858 | | ICD10 |  | Other specified viral encephalitis | | | | | | | | |  | |  | | |  |
| A86 | | ICD10 |  | Unspecified viral encephalitis | | | | | | | | |  | |  | | |  |
| A870 | | ICD10 |  | Enteroviral meningitis | | | | | | | | |  | |  | | |  |
| A871 | | ICD10 |  | Adenoviral meningitis | | | | | | | | |  | |  | | |  |
| A872 | | ICD10 |  | Lymphocytic choriomeningitis | | | | | | | | |  | |  | | |  |
| A878 | | ICD10 |  | Other viral meningitis | | | | | | | | |  | |  | | |  |
| A879 | | ICD10 |  | Viral meningitis, unspecified | | | | | | | | |  | |  | | |  |
| A880 | | ICD10 |  | Enteroviral exanthematous fever [Boston exanthem] | | | | | | | | |  | |  | | |  |
| A888 | | ICD10 |  | Other specified viral infections of central nervous system | | | | | | | | |  | |  | | |  |
| A89 | | ICD10 |  | Unspecified viral infection of central nervous system | | | | | | | | |  | |  | | |  |
| A90 | | ICD10 |  | Dengue fever [classical dengue] | | | | | | | | |  | |  | | |  |
| B004 | | ICD10 |  | Herpesviral encephalitis | | | | | | | | |  | |  | | |  |
| B0111 | | ICD10 |  | Varicella encephalitis and encephalomyelitis | | | | | | | | |  | |  | | |  |
| B020 | | ICD10 |  | Zoster encephalitis | | | | | | | | |  | |  | | |  |
| B050 | | ICD10 |  | Measles complicated by encephalitis | | | | | | | | |  | |  | | |  |
| B0601 | | ICD10 |  | Rubella encephalitis | | | | | | | | |  | |  | | |  |
| B1001 | | ICD10 |  | Human herpesvirus 6 encephalitis | | | | | | | | |  | |  | | |  |
| B1009 | | ICD10 |  | Other human herpesvirus encephalitis | | | | | | | | |  | |  | | |  |
| B262 | | ICD10 |  | Mumps encephalitis | | | | | | | | |  | |  | | |  |
| B4081 | | ICD10 |  | Blastomycotic meningoencephalitis | | | | | | | | |  | |  | | |  |
| B5742 | | ICD10 |  | Meningoencephalitis in Chagas' disease | | | | | | | | |  | |  | | |  |
| B582 | | ICD10 |  | Toxoplasma meningoencephalitis | | | | | | | | |  | |  | | |  |
| B6011 | | ICD10 |  | Meningoencephalitis due to Acanthamoeba (culbertsoni) | | | | | | | | |  | |  | | |  |
| B941 | | ICD10 |  | Sequelae of viral encephalitis | | | | | | | | |  | |  | | |  |
| G0400 | | ICD10 |  | Acute disseminated encephalitis and encephalomyelitis, unspecified | | | | | | | | |  | |  | | |  |
| G0401 | | ICD10 |  | Postinfectious acute disseminated encephalitis and encephalomyelitis (postinfectious ADEM) | | | | | | | | |  | |  | | |  |
| G0402 | | ICD10 |  | Postimmunization acute disseminated encephalitis, myelitis and encephalomyelitis | | | | | | | | |  | |  | | |  |
| G042 | | ICD10 |  | Bacterial meningoencephalitis and meningomyelitis, not elsewhere classified | | | | | | | | |  | |  | | |  |
| G0481 | | ICD10 |  | Other encephalitis and encephalomyelitis | | | | | | | | |  | |  | | |  |
| G0490 | | ICD10 |  | Encephalitis and encephalomyelitis, unspecified | | | | | | | | |  | |  | | |  |
| G361 | | ICD10 |  | Acute and subacute hemorrhagic leukoencephalitis [Hurst] | | | | | | | | |  | |  | | |  |
| A1782 | | ICD10 |  | Tuberculous meningoencephalitis | | | | | | | | |  | |  | | |  |
| A3212 | | ICD10 |  | Listerial meningoencephalitis | | | | | | | | |  | |  | | |  |
| A5141 | | ICD10 |  | Secondary syphilitic meningitis | | | | | | | | |  | |  | | |  |
| A5213 | | ICD10 |  | Late syphilitic meningitis | | | | | | | | |  | |  | | |  |
| A5481 | | ICD10 |  | Gonococcal meningitis | | | | | | | | |  | |  | | |  |
| A6921 | | ICD10 |  | Meningitis due to Lyme disease | | | | | | | | |  | |  | | |  |
| A870 | | ICD10 |  | Enteroviral meningitis | | | | | | | | |  | |  | | |  |
| A871 | | ICD10 |  | Adenoviral meningitis | | | | | | | | |  | |  | | |  |
| A872 | | ICD10 |  | Lymphocytic choriomeningitis | | | | | | | | |  | |  | | |  |
| A878 | | ICD10 |  | Other viral meningitis | | | | | | | | |  | |  | | |  |
| A879 | | ICD10 |  | Viral meningitis, unspecified | | | | | | | | |  | |  | | |  |
| A880 | | ICD10 |  | Enteroviral exanthematous fever [Boston exanthem] | | | | | | | | |  | |  | | |  |
| A888 | | ICD10 |  | Other specified viral infections of central nervous system | | | | | | | | |  | |  | | |  |
| A89 | | ICD10 |  | Unspecified viral infection of central nervous system | | | | | | | | |  | |  | | |  |
| B003 | | ICD10 |  | Herpesviral meningitis | | | | | | | | |  | |  | | |  |
| B010 | | ICD10 |  | Varicella meningitis | | | | | | | | |  | |  | | |  |
| B021 | | ICD10 |  | Zoster meningitis | | | | | | | | |  | |  | | |  |
| B051 | | ICD10 |  | Measles complicated by meningitis | | | | | | | | |  | |  | | |  |
| B0602 | | ICD10 |  | Rubella meningitis | | | | | | | | |  | |  | | |  |
| B261 | | ICD10 |  | Mumps meningitis | | | | | | | | |  | |  | | |  |
| B2702 | | ICD10 |  | Gammaherpesviral mononucleosis with meningitis | | | | | | | | |  | |  | | |  |
| B2712 | | ICD10 |  | Cytomegaloviral mononucleosis with meningitis | | | | | | | | |  | |  | | |  |
| B2782 | | ICD10 |  | Other infectious mononucleosis with meningitis | | | | | | | | |  | |  | | |  |
| B2792 | | ICD10 |  | Infectious mononucleosis, unspecified with meningitis | | | | | | | | |  | |  | | |  |
| B384 | | ICD10 |  | Coccidioidomycosis meningitis | | | | | | | | |  | |  | | |  |
| B5741 | | ICD10 |  | Meningitis in Chagas' disease | | | | | | | | |  | |  | | |  |
| D8681 | | ICD10 |  | Sarcoid meningitis | | | | | | | | |  | |  | | |  |
| G000 | | ICD10 |  | Hemophilus meningitis | | | | | | | | |  | |  | | |  |
| G001 | | ICD10 |  | Pneumococcal meningitis | | | | | | | | |  | |  | | |  |
| G002 | | ICD10 |  | Streptococcal meningitis | | | | | | | | |  | |  | | |  |
| G003 | | ICD10 |  | Staphylococcal meningitis | | | | | | | | |  | |  | | |  |
| G008 | | ICD10 |  | Other bacterial meningitis | | | | | | | | |  | |  | | |  |
| G009 | | ICD10 |  | Bacterial meningitis, unspecified | | | | | | | | |  | |  | | |  |
| G01 | | ICD10 |  | Meningitis in bacterial diseases classified elsewhere | | | | | | | | |  | |  | | |  |
| G02 | | ICD10 |  | Meningitis in other infectious and parasitic diseases classified elsewhere | | | | | | | | |  | |  | | |  |
| G030 | | ICD10 |  | Nonpyogenic meningitis | | | | | | | | |  | |  | | |  |
| G031 | | ICD10 |  | Chronic meningitis | | | | | | | | |  | |  | | |  |
| G032 | | ICD10 |  | Benign recurrent meningitis [Mollaret] | | | | | | | | |  | |  | | |  |
| G038 | | ICD10 |  | Meningitis due to other specified causes | | | | | | | | |  | |  | | |  |
| G039 | | ICD10 |  | Meningitis, unspecified | | | | | | | | |  | |  | | |  |
|  | |  |  |  | | | | | | | | |  | |  | | |  |
|  | |  |  |  | | | | | | | | |  | |  | | |  |
|  | |  |  |  | | | | | | | | |  | |  | | |  |
|  | |  |  |  | | | | | | | | |  | |  | | |  |
|  | |  |  |  | | | | | | | | |  | |  | | |  |
|  | |  |  |  | | | | | | | | |  | |  | | |  |
|  | |  |  |  | | | | | | | | |  | |  | | |  |
|  | |  |  |  | | | | | | | | |  | |  | | |  |
|  | |  |  |  | | | | | | | | |  | |  | | |  |
|  | |  |  |  | | | | | | | | |  | |  | | |  |
|  | |  |  |  | | | | | | | | |  | |  | | |  |
|  | |  |  |  | | | | | | | | |  | |  | | |  |
|  | |  |  |  | | | | | | | | |  | |  | | |  |
|  | |  |  |  | | | | | | | | |  | |  | | |  |
|  | |  |  |  | | | | | | | | |  | |  | | |  |
|  | |  |  |  | | | | | | | | |  | |  | | |  |
|  | |  |  |  | | | | | | | | |  | |  | | |  |
|  | |  |  |  | | | | | | | | |  | |  | | |  |
|  | |  |  |  | | | | | | | | |  | |  | | |  |
|  | |  |  |  | | | | | | | | |  | |  | | |  |
|  | |  |  |  | | | | | | | | |  | |  | | |  |
|  | |  |  |  | | | | | | | | |  | |  | | |  |
|  | |  |  |  | | | | | | | | |  | |  | | |  |
|  | |  |  |  | | | | | | | | |  | |  | | |  |
|  | |  |  |  | | | | | | | | |  | |  | | |  |
|  | |  |  |  | | | | | | | | |  | |  | | |  |
|  | |  |  |  | | | | | | | | |  | |  | | |  |
|  | |  |  |  | | | | | | | | |  | |  | | |  |
|  | |  |  |  | | | | | | | | |  | |  | | |  |
|  | |  |  |  | | | | | | | | |  | |  | | |  |
|  | |  |  |  | | | | | | | | |  | |  | | |  |
|  | |  |  |  | | | | | | | | |  | |  | | |  |
|  | |  |  |  | | | | | | | | |  | |  | | |  |
|  | |  |  |  | | | | | | | | |  | |  | | |  |
|  | |  |  |  | | | | | | | | |  | |  | | |  |
|  | |  |  |  | | | | | | | | |  | |  | | |  |
|  | |  |  |  | | | | | | | | |  | |  | | |  |
|  | |  |  |  | | | | | | | | |  | |  | | |  |
|  | |  |  |  | | | | | | | | |  | |  | | |  |
|  | |  |  |  | | | | | | | | |  | |  | | |  |
|  | |  |  |  | | | | | | | | |  | |  | | |  |
|  | |  |  |  | | | | | | | | |  | |  | | |  |
|  | |  |  |  | | | | | | | | |  | |  | | |  |
|  | |  |  |  | | | | | | | | |  | |  | | |  |
|  | |  |  |  | | | | | | | | |  | |  | | |  |
|  | |  |  |  | | | | | | | | |  | |  | | |  |
|  | |  |  |  | | | | | | | | |  | |  | | |  |
|  | |  |  |  | | | | | | | | |  | |  | | |  |
|  | |  |  |  | | | | | | | | |  | |  | | |  |
|  | |  |  |  | | | | | | | | |  | |  | | |  |
|  | |  |  |  | | | | | | | | |  | |  | | |  |
|  | |  |  |  | | | | | | | | |  | |  | | |  |
|  | |  |  |  | | | | | | | | |  | |  | | |  |
|  | |  |  |  | | | | | | | | |  | |  | | |  |
|  | |  |  |  | | | | | | | | |  | |  | | |  |
|  | |  |  |  | | | | | | | | |  | |  | | |  |
|  | |  |  |  | | | | | | | | |  | |  | | |  |
|  | |  |  |  | | | | | | | | |  | |  | | |  |
|  | |  |  |  | | | | | | | | |  | |  | | |  |
|  | |  |  |  | | | | | | | | |  | |  | | |  |
|  | |  |  |  | | | | | | | | |  | |  | | |  |
|  | |  |  |  | | | | | | | | |  | |  | | |  |
|  | |  |  |  | | | | | | | | |  | |  | | |  |
|  | |  |  |  | | | | | | | | |  | |  | | |  |
|  | |  |  |  | | | | | | | | |  | |  | | |  |
|  | |  |  |  | | | | | | | | |  | |  | | |  |
|  | |  |  |  | | | | | | | | |  | |  | | |  |
|  | |  |  |  | | | | | | | | |  | |  | | |  |
|  | |  |  |  | | | | | | | | |  | |  | | |  |
|  | |  |  |  | | | | | | | | |  | |  | | |  |
|  | |  |  |  | | | | | | | | |  | |  | | |  |
|  | |  |  |  | | | | | | | | |  | |  | | |  |
|  | |  |  |  | | | | | | | | |  | |  | | |  |
|  | |  |  |  | | | | | | | | |  | |  | | |  |
|  | |  |  |  | | | | | | | | |  | |  | | |  |
|  | |  |  |  | | | | | | | | |  | |  | | |  |
|  | |  |  |  | | | | | | | | |  | |  | | |  |
|  | |  |  |  | | | | | | | | |  | |  | | |  |
|  | |  |  |  | | | | | | | | |  | |  | | |  |
|  | |  |  |  | | | | | | | | |  | |  | | |  |
|  | |  |  |  | | | | | | | | |  | |  | | |  |
|  | |  |  |  | | | | | | | | |  | |  | | |  |
|  | |  |  |  | | | | | | | | |  | |  | | |  |
|  | |  |  |  | | | | | | | | |  | |  | | |  |
|  | |  |  |  | | | | | | | | |  | |  | | |  |
|  | |  |  |  | | | | | | | | |  | |  | | |  |
|  | |  |  |  | | | | | | | | |  | |  | | |  |
|  | |  |  |  | | | | | | | | |  | |  | | |  |
|  | |  |  |  | | | | | | | | |  | |  | | |  |
|  | |  |  |  | | | | | | | | |  | |  | | |  |
|  | |  |  |  | | | | | | | | |  | |  | | |  |
|  | |  |  |  | | | | | | | | |  | |  | | |  |
|  | |  |  |  | | | | | | | | |  | |  | | |  |
|  | |  |  |  | | | | | | | | |  | |  | | |  |
|  | |  |  |  | | | | | | | | |  | |  | | |  |
|  | |  |  |  | | | | | | | | |  | |  | | |  |
|  | |  |  |  | | | | | | | | |  | |  | | |  |
|  | |  |  |  | | | | | | | | |  | |  | | |  |
|  | |  |  |  | | | | | | | | |  | |  | | |  |
|  | |  |  |  | | | | | | | | |  | |  | | |  |
|  | |  |  |  | | | | | | | | |  | |  | | |  |
|  | |  |  |  | | | | | | | | |  | |  | | |  |
|  | |  |  |  | | | | | | | | |  | |  | | |  |
|  | |  |  |  | | | | | | | | |  | |  | | |  |
|  | |  |  |  | | | | | | | | |  | |  | | |  |
|  | |  |  |  | | | | | | | | |  | |  | | |  |
|  | |  |  |  | | | | | | | | |  | |  | | |  |
|  | |  |  |  | | | | | | | | |  | |  | | |  |
|  | |  |  |  | | | | | | | | |  | |  | | |  |
|  | |  |  |  | | | | | | | | |  | |  | | |  |
|  | |  |  |  | | | | | | | | |  | |  | | |  |
|  | |  |  |  | | | | | | | | |  | |  | | |  |
|  | |  |  |  | | | | | | | | |  | |  | | |  |
|  | |  |  |  | | | | | | | | |  | |  | | |  |
|  | |  |  |  | | | | | | | | |  | |  | | |  |
|  | |  |  |  | | | | | | | | |  | |  | | |  |
|  | |  |  |  | | | | | | | | |  | |  | | |  |
|  | |  |  |  | | | | | | | | |  | |  | | |  |
|  | |  |  |  | | | | | | | | |  | |  | | |  |
|  | |  |  |  | | | | | | | | |  | |  | | |  |
|  | |  |  |  | | | | | | | | |  | |  | | |  |
|  | |  |  |  | | | | | | | | |  | |  | | |  |
|  | |  |  |  | | | | | | | | |  | |  | | |  |
|  | |  |  |  | | | | | | | | |  | |  | | |  |
|  | |  |  |  | | | | | | | | |  | |  | | |  |
|  | |  |  |  | | | | | | | | |  | |  | | |  |
|  | |  |  |  | | | | | | | | |  | |  | | |  |
|  | |  |  |  | | | | | | | | |  | |  | | |  |
|  | |  |  |  | | | | | | | | |  | |  | | |  |
|  | |  |  |  | | | | | | | | |  | |  | | |  |
|  | |  |  |  | | | | | | | | |  | |  | | |  |
|  | |  |  |  | | | | | | | | |  | |  | | |  |
|  | |  |  |  | | | | | | | | |  | |  | | |  |
|  | |  |  |  | | | | | | | | |  | |  | | |  |
|  | |  |  |  | | | | | | | | |  | |  | | |  |
|  | |  |  |  | | | | | | | | |  | |  | | |  |
|  | |  |  |  | | | | | | | | |  | |  | | |  |
|  | |  |  |  | | | | | | | | |  | |  | | |  |
|  | |  |  |  | | | | | | | | |  | |  | | |  |
|  | |  |  |  | | | | | | | | |  | |  | | |  |
|  | |  |  |  | | | | | | | | |  | |  | | |  |
|  | |  |  |  | | | | | | | | |  | |  | | |  |
|  | |  |  |  | | | | | | | | |  | |  | | |  |
|  | |  |  |  | | | | | | | | |  | |  | | |  |
|  | |  |  |  | | | | | | | | |  | |  | | |  |
|  | |  |  |  | | | | | | | | |  | |  | | |  |
|  | |  |  |  | | | | | | | | |  | |  | | |  |
|  | |  |  |  | | | | | | | | |  | |  | | |  |
|  | |  |  |  | | | | | | | | |  | |  | | |  |
|  | |  |  |  | | | | | | | | |  | |  | | |  |
|  | |  |  |  | | | | | | | | |  | |  | | |  |
|  | |  |  |  | | | | | | | | |  | |  | | |  |
|  | |  |  |  | | | | | | | | |  | |  | | |  |
|  | |  |  |  | | | | | | | | |  | |  | | |  |
|  | |  |  |  | | | | | | | | |  | |  | | |  |
|  | |  |  |  | | | | | | | | |  | |  | | |  |
|  | |  |  |  | | | | | | | | |  | |  | | |  |
|  | |  |  |  | | | | | | | | |  | |  | | |  |
|  | |  |  |  | | | | | | | | |  | |  | | |  |
|  | |  |  |  | | | | | | | | |  | |  | | |  |
|  | |  |  |  | | | | | | | | |  | |  | | |  |
|  | |  |  |  | | | | | | | | |  | |  | | |  |
|  | |  |  |  | | | | | | | | |  | |  | | |  |
|  | |  |  |  | | | | | | | | |  | |  | | |  |
|  | |  |  |  | | | | | | | | |  | |  | | |  |
|  | |  |  |  | | | | | | | | |  | |  | | |  |
|  | |  |  |  | | | | | | | | |  | |  | | |  |
|  | |  |  |  | | | | | | | | |  | |  | | |  |
|  | |  |  |  | | | | | | | | |  | |  | | |  |
|  | |  |  |  | | | | | | | | |  | |  | | |  |
|  | |  |  |  | | | | | | | | |  | |  | | |  |
|  | |  |  |  | | | | | | | | |  | |  | | |  |
|  | |  |  |  | | | | | | | | |  | |  | | |  |
|  | |  |  |  | | | | | | | | |  | |  | | |  |
|  | |  |  |  | | | | | | | | |  | |  | | |  |
|  | |  |  |  | | | | | | | | |  | |  | | |  |
|  | |  |  |  | | | | | | | | |  | |  | | |  |
|  | |  |  |  | | | | | | | | |  | |  | | |  |
|  | |  |  |  | | | | | | | | |  | |  | | |  |
|  | |  |  |  | | | | | | | | |  | |  | | |  |
|  | |  |  |  | | | | | | | | |  | |  | | |  |
|  | |  |  |  | | | | | | | | |  | |  | | |  |
|  | |  |  |  | | | | | | | | |  | |  | | |  |
|  | |  |  |  | | | | | | | | |  | |  | | |  |
|  | |  |  |  | | | | | | | | |  | |  | | |  |
|  | |  |  |  | | | | | | | | |  | |  | | |  |
|  | |  |  |  | | | | | | | | |  | |  | | |  |
|  | |  |  |  | | | | | | | | |  | |  | | |  |
|  | |  |  |  | | | | | | | | |  | |  | | |  |
|  | |  |  |  | | | | | | | | |  | |  | | |  |
|  | |  |  |  | | | | | | | | |  | |  | | |  |
|  | |  |  |  | | | | | | | | |  | |  | | |  |
|  | |  |  |  | | | | | | | | |  | |  | | |  |
|  | |  |  |  | | | | | | | | |  | |  | | |  |
|  | |  |  |  | | | | | | | | |  | |  | | |  |
|  | |  |  |  | | | | | | | | |  | |  | | |  |
|  | |  |  |  | | | | | | | | |  | |  | | |  |
|  | |  |  |  | | | | | | | | |  | |  | | |  |
|  | |  |  |  | | | | | | | | |  | |  | | |  |
|  | |  |  |  | | | | | | | | |  | |  | | |  |
|  | |  |  |  | | | | | | | | |  | |  | | |  |
|  | |  |  |  | | | | | | | | |  | |  | | |  |
|  | |  |  |  | | | | | | | | |  | |  | | |  |
|  | |  |  |  | | | | | | | | |  | |  | | |  |
|  | |  |  |  | | | | | | | | |  | |  | | |  |
|  | |  |  |  | | | | | | | | |  | |  | | |  |
|  | |  |  |  | | | | | | | | |  | |  | | |  |
|  | |  |  |  | | | | | | | | |  | |  | | |  |
|  | |  |  |  | | | | | | | | |  | |  | | |  |
|  | |  |  |  | | | | | | | | |  | |  | | |  |
|  | |  |  |  | | | | | | | | |  | |  | | |  |
|  | |  |  |  | | | | | | | | |  | |  | | |  |
|  | |  |  |  | | | | | | | | |  | |  | | |  |
|  | |  |  |  | | | | | | | | |  | |  | | |  |
|  | |  |  |  | | | | | | | | |  | |  | | |  |
|  | |  |  |  | | | | | | | | |  | |  | | |  |
|  | |  |  |  | | | | | | | | |  | |  | | |  |
|  | |  |  |  | | | | | | | | |  | |  | | |  |
|  | |  |  |  | | | | | | | | |  | |  | | |  |
|  | |  |  |  | | | | | | | | |  | |  | | |  |
|  | |  |  |  | | | | | | | | |  | |  | | |  |
|  | |  |  |  | | | | | | | | |  | |  | | |  |
|  | |  |  |  | | | | | | | | |  | |  | | |  |
|  | |  |  |  | | | | | | | | |  | |  | | |  |
|  | |  |  |  | | | | | | | | |  | |  | | |  |
|  | |  |  |  | | | | | | | | |  | |  | | |  |
|  | |  |  |  | | | | | | | | |  | |  | | |  |
|  | |  |  |  | | | | | | | | |  | |  | | |  |
|  | |  |  |  | | | | | | | | |  | |  | | |  |
|  | |  |  |  | | | | | | | | |  | |  | | |  |
|  | |  |  |  | | | | | | | | |  | |  | | |  |
|  | |  |  |  | | | | | | | | |  | |  | | |  |
|  | |  |  |  | | | | | | | | |  | |  | | |  |
|  | |  |  |  | | | | | | | | |  | |  | | |  |
|  | |  |  |  | | | | | | | | |  | |  | | |  |
|  | |  |  |  | | | | | | | | |  | |  | | |  |
|  | |  |  |  | | | | | | | | |  | |  | | |  |
|  | |  |  |  | | | | | | | | |  | |  | | |  |
|  | |  |  |  | | | | | | | | |  | |  | | |  |
|  | |  |  |  | | | | | | | | |  | |  | | |  |
|  | |  |  |  | | | | | | | | |  | |  | | |  |
|  | |  |  |  | | | | | | | | |  | |  | | |  |
|  | |  |  |  | | | | | | | | |  | |  | | |  |
|  | |  |  |  | | | | | | | | |  | |  | | |  |
|  | |  |  |  | | | | | | | | |  | |  | | |  |
|  | |  |  |  | | | | | | | | |  | |  | | |  |
|  | |  |  |  | | | | | | | | |  | |  | | |  |
|  | |  |  |  | | | | | | | | |  | |  | | |  |
|  | |  |  |  | | | | | | | | |  | |  | | |  |
|  | |  |  |  | | | | | | | | |  | |  | | |  |
|  | |  |  |  | | | | | | | | |  | |  | | |  |
|  | |  |  |  | | | | | | | | |  | |  | | |  |
|  | |  |  |  | | | | | | | | |  | |  | | |  |
|  | |  |  |  | | | | | | | | |  | |  | | |  |
|  | |  |  |  | | | | | | | | |  | |  | | |  |
|  | |  |  |  | | | | | | | | |  | |  | | |  |
|  | |  |  |  | | | | | | | | |  | |  | | |  |
|  | |  |  |  | | | | | | | | |  | |  | | |  |
|  | |  |  |  | | | | | | | | |  | |  | | |  |
|  | |  |  |  | | | | | | | | |  | |  | | |  |
|  | |  |  |  | | | | | | | | |  | |  | | |  |
|  | |  |  |  | | | | | | | | |  | |  | | |  |
|  | |  |  |  | | | | | | | | |  | |  | | |  |
|  | |  |  |  | | | | | | | | |  | |  | | |  |
|  | |  |  |  | | | | | | | | |  | |  | | |  |
|  | |  |  |  | | | | | | | | |  | |  | | |  |
|  | |  |  |  | | | | | | | | |  | |  | | |  |
|  | |  |  |  | | | | | | | | |  | |  | | |  |
|  | |  |  |  | | | | | | | | |  | |  | | |  |
|  | |  |  |  | | | | | | | | |  | |  | | |  |
|  | |  |  |  | | | | | | | | |  | |  | | |  |
|  | |  |  |  | | | | | | | | |  | |  | | |  |
|  | |  |  |  | | | | | | | | |  | |  | | |  |
|  | |  |  |  | | | | | | | | |  | |  | | |  |
|  | |  |  |  | | | | | | | | |  | |  | | |  |
|  | |  |  |  | | | | | | | | |  | |  | | |  |
|  | |  |  |  | | | | | | | | |  | |  | | |  |
|  | |  |  |  | | | | | | | | |  | |  | | |  |
|  | |  |  |  | | | | | | | | |  | |  | | |  |
|  | |  |  |  | | | | | | | | |  | |  | | |  |
|  | |  |  |  | | | | | | | | |  | |  | | |  |
|  | |  |  |  | | | | | | | | |  | |  | | |  |
|  | |  |  |  | | | | | | | | |  | |  | | |  |
|  | |  |  |  | | | | | | | | |  | |  | | |  |
|  | |  |  |  | | | | | | | | |  | |  | | |  |
|  | |  |  |  | | | | | | | | |  | |  | | |  |
|  | |  |  |  | | | | | | | | |  | |  | | |  |
|  | |  |  |  | | | | | | | | |  | |  | | |  |
|  | |  |  |  | | | | | | | | |  | |  | | |  |
|  | |  |  |  | | | | | | | | |  | |  | | |  |
|  | |  |  |  | | | | | | | | |  | |  | | |  |
|  | |  |  |  | | | | | | | | |  | |  | | |  |
|  | |  |  |  | | | | | | | | |  | |  | | |  |
|  | |  |  |  | | | | | | | | |  | |  | | |  |
|  | |  |  |  | | | | | | | | |  | |  | | |  |
|  | |  |  |  | | | | | | | | |  | |  | | |  |
|  | |  |  |  | | | | | | | | |  | |  | | |  |
|  | |  |  |  | | | | | | | | |  | |  | | |  |
|  | |  |  |  | | | | | | | | |  | |  | | |  |
|  | |  |  |  | | | | | | | | |  | |  | | |  |
|  | |  |  |  | | | | | | | | |  | |  | | |  |
|  | |  |  |  | | | | | | | | |  | |  | | |  |
|  | |  |  |  | | | | | | | | |  | |  | | |  |
|  | |  |  |  | | | | | | | | |  | |  | | |  |
|  | |  |  |  | | | | | | | | |  | |  | | |  |
|  | |  |  |  | | | | | | | | |  | |  | | |  |
|  | |  |  |  | | | | | | | | |  | |  | | |  |
|  | |  |  |  | | | | | | | | |  | |  | | |  |
|  | |  |  |  | | | | | | | | |  | |  | | |  |
|  | |  |  |  | | | | | | | | |  | |  | | |  |
|  | |  |  |  | | | | | | | | |  | |  | | |  |
|  | |  |  |  | | | | | | | | |  | |  | | |  |
|  | |  |  |  | | | | | | | | |  | |  | | |  |
|  | |  |  |  | | | | | | | | |  | |  | | |  |
|  | |  |  |  | | | | | | | | |  | |  | | |  |
|  | |  |  |  | | | | | | | | |  | |  | | |  |
|  | |  |  |  | | | | | | | | |  | |  | | |  |
|  | |  |  |  | | | | | | | | |  | |  | | |  |
|  | |  |  |  | | | | | | | | |  | |  | | |  |
|  | |  |  |  | | | | | | | | |  | |  | | |  |
|  | |  |  |  | | | | | | | | |  | |  | | |  |
|  | |  |  |  | | | | | | | | |  | |  | | |  |
|  | |  |  |  | | | | | | | | |  | |  | | |  |
|  | |  |  |  | | | | | | | | |  | |  | | |  |
|  | |  |  |  | | | | | | | | |  | |  | | |  |
|  | |  |  |  | | | | | | | | |  | |  | | |  |
|  | |  |  |  | | | | | | | | |  | |  | | |  |
|  | |  |  |  | | | | | | | | |  | |  | | |  |
|  | |  |  |  | | | | | | | | |  | |  | | |  |
|  | |  |  |  | | | | | | | | |  | |  | | |  |
|  | |  |  |  | | | | | | | | |  | |  | | |  |
|  | |  |  |  | | | | | | | | |  | |  | | |  |
|  | |  |  |  | | | | | | | | |  | |  | | |  |
|  | |  |  |  | | | | | | | | |  | |  | | |  |
|  | |  |  |  | | | | | | | | |  | |  | | |  |
|  | |  |  |  | | | | | | | | |  | |  | | |  |
|  | |  |  |  | | | | | | | | |  | |  | | |  |
|  | |  |  |  | | | | | | | | |  | |  | | |  |
|  | |  |  |  | | | | | | | | |  | |  | | |  |
|  | |  |  |  | | | | | | | | |  | |  | | |  |
|  | |  |  |  | | | | | | | | |  | |  | | |  |
|  | |  |  |  | | | | | | | | |  | |  | | |  |
|  | |  |  |  | | | | | | | | |  | |  | | |  |
|  | |  |  |  | | | | | | | | |  | |  | | |  |
|  | |  |  |  | | | | | | | | |  | |  | | |  |
|  | |  |  |  | | | | | | | | |  | |  | | |  |
|  | |  |  |  | | | | | | | | |  | |  | | |  |
|  | |  |  |  | | | | | | | | |  | |  | | |  |
|  | |  |  |  | | | | | | | | |  | |  | | |  |
|  | |  |  |  | | | | | | | | |  | |  | | |  |
|  | |  |  |  | | | | | | | | |  | |  | | |  |
|  | |  |  |  | | | | | | | | |  | |  | | |  |
|  | |  |  |  | | | | | | | | |  | |  | | |  |
|  | |  |  |  | | | | | | | | |  | |  | | |  |
|  | |  |  |  | | | | | | | | |  | |  | | |  |
|  | |  |  |  | | | | | | | | |  | |  | | |  |
|  | |  |  |  | | | | | | | | |  | |  | | |  |
|  | |  |  |  | | | | | | | | |  | |  | | |  |
|  | |  |  |  | | | | | | | | |  | |  | | |  |
|  | |  |  |  | | | | | | | | |  | |  | | |  |
|  | |  |  |  | | | | | | | | |  | |  | | |  |
|  | |  |  |  | | | | | | | | |  | |  | | |  |
|  | |  |  |  | | | | | | | | |  | |  | | |  |
|  | |  |  |  | | | | | | | | |  | |  | | |  |
|  | |  |  |  | | | | | | | | |  | |  | | |  |
|  | |  |  |  | | | | | | | | |  | |  | | |  |
|  | |  |  |  | | | | | | | | |  | |  | | |  |
|  | |  |  |  | | | | | | | | |  | |  | | |  |
|  | |  |  |  | | | | | | | | |  | |  | | |  |
|  | |  |  |  | | | | | | | | |  | |  | | |  |
|  | |  |  |  | | | | | | | | |  | |  | | |  |
|  | |  |  |  | | | | | | | | |  | |  | | |  |
|  | |  |  |  | | | | | | | | |  | |  | | |  |
|  | |  |  |  | | | | | | | | |  | |  | | |  |
|  | |  |  |  | | | | | | | | |  | |  | | |  |
|  | |  |  |  | | | | | | | | |  | |  | | |  |
|  | |  |  |  | | | | | | | | |  | |  | | |  |
|  | |  |  |  | | | | | | | | |  | |  | | |  |
|  | |  |  |  | | | | | | | | |  | |  | | |  |
|  | |  |  |  | | | | | | | | |  | |  | | |  |
|  | |  |  |  | | | | | | | | |  | |  | | |  |
|  | |  |  |  | | | | | | | | |  | |  | | |  |
|  | |  |  |  | | | | | | | | |  | |  | | |  |
|  | |  |  |  | | | | | | | | |  | |  | | |  |
|  | |  |  |  | | | | | | | | |  | |  | | |  |
|  | |  |  |  | | | | | | | | |  | |  | | |  |
|  | |  |  |  | | | | | | | | |  | |  | | |  |
|  | |  |  |  | | | | | | | | |  | |  | | |  |
|  | |  |  |  | | | | | | | | |  | |  | | |  |
|  | |  |  |  | | | | | | | | |  | |  | | |  |
|  | |  |  |  | | | | | | | | |  | |  | | |  |
|  | |  |  |  | | | | | | | | |  | |  | | |  |
|  | |  |  |  | | | | | | | | |  | |  | | |  |
|  | |  |  |  | | | | | | | | |  | |  | | |  |
|  | |  |  |  | | | | | | | | |  | |  | | |  |
|  | |  |  |  | | | | | | | | |  | |  | | |  |
|  | |  |  |  | | | | | | | | |  | |  | | |  |
|  | |  |  |  | | | | | | | | |  | |  | | |  |
|  | |  |  |  | | | | | | | | |  | |  | | |  |
|  | |  |  |  | | | | | | | | |  | |  | | |  |
|  | |  |  |  | | | | | | | | |  | |  | | |  |
|  | |  |  |  | | | | | | | | |  | |  | | |  |
|  | |  |  |  | | | | | | | | |  | |  | | |  |
|  | |  |  |  | | | | | | | | |  | |  | | |  |
|  | |  |  |  | | | | | | | | |  | |  | | |  |
|  | |  |  |  | | | | | | | | |  | |  | | |  |
|  | |  |  |  | | | | | | | | |  | |  | | |  |
|  | |  |  |  | | | | | | | | |  | |  | | |  |
|  | |  |  |  | | | | | | | | |  | |  | | |  |
|  | |  |  |  | | | | | | | | |  | |  | | |  |
|  | |  |  |  | | | | | | | | |  | |  | | |  |
|  | |  |  |  | | | | | | | | |  | |  | | |  |
|  | |  |  |  | | | | | | | | |  | |  | | |  |
|  | |  |  |  | | | | | | | | |  | |  | | |  |
|  | |  |  |  | | | | | | | | |  | |  | | |  |
|  | |  |  |  | | | | | | | | |  | |  | | |  |
|  | |  |  |  | | | | | | | | |  | |  | | |  |
|  | |  |  |  | | | | | | | | |  | |  | | |  |
|  | |  |  |  | | | | | | | | |  | |  | | |  |
|  | |  |  |  | | | | | | | | |  | |  | | |  |
|  | |  |  |  | | | | | | | | |  | |  | | |  |
|  | |  |  |  | | | | | | | | |  | |  | | |  |
|  | |  |  |  | | | | | | | | |  | |  | | |  |
|  | |  |  |  | | | | | | | | |  | |  | | |  |
|  | |  |  |  | | | | | | | | |  | |  | | |  |
|  | |  |  |  | | | | | | | | |  | |  | | |  |
|  | |  |  |  | | | | | | | | |  | |  | | |  |
|  | |  |  |  | | | | | | | | |  | |  | | |  |
|  | |  |  |  | | | | | | | | |  | |  | | |  |
|  | |  |  |  | | | | | | | | |  | |  | | |  |
|  | |  |  |  | | | | | | | | |  | |  | | |  |
|  | |  |  |  | | | | | | | | |  | |  | | |  |
|  | |  |  |  | | | | | | | | |  | |  | | |  |
|  | |  |  |  | | | | | | | | |  | |  | | |  |
|  | |  |  |  | | | | | | | | |  | |  | | |  |
|  | |  |  |  | | | | | | | | |  | |  | | |  |
|  | |  |  |  | | | | | | | | |  | |  | | |  |
|  | |  |  |  | | | | | | | | |  | |  | | |  |
|  | |  |  |  | | | | | | | | |  | |  | | |  |
|  | |  |  |  | | | | | | | | |  | |  | | |  |
|  | |  |  |  | | | | | | | | |  | |  | | |  |
|  | |  |  |  | | | | | | | | |  | |  | | |  |
|  | |  |  |  | | | | | | | | |  | |  | | |  |
|  | |  |  |  | | | | | | | | |  | |  | | |  |
|  | |  |  |  | | | | | | | | |  | |  | | |  |
|  | |  |  |  | | | | | | | | |  | |  | | |  |
|  | |  |  |  | | | | | | | | |  | |  | | |  |
|  | |  |  |  | | | | | | | | |  | |  | | |  |
|  | |  |  |  | | | | | | | | |  | |  | | |  |
|  | |  |  |  | | | | | | | | |  | |  | | |  |
|  | |  |  |  | | | | | | | | |  | |  | | |  |
|  | |  |  |  | | | | | | | | |  | |  | | |  |
|  | |  |  |  | | | | | | | | |  | |  | | |  |
|  | |  |  |  | | | | | | | | |  | |  | | |  |
|  | |  |  |  | | | | | | | | |  | |  | | |  |
|  | |  |  |  | | | | | | | | |  | |  | | |  |
|  | |  |  |  | | | | | | | | |  | |  | | |  |
|  | |  |  |  | | | | | | | | |  | |  | | |  |
|  | |  |  |  | | | | | | | | |  | |  | | |  |
|  | |  |  |  | | | | | | | | |  | |  | | |  |
|  | |  |  |  | | | | | | | | |  | |  | | |  |
|  | |  |  |  | | | | | | | | |  | |  | | |  |
|  | |  |  |  | | | | | | | | |  | |  | | |  |
|  | |  |  |  | | | | | | | | |  | |  | | |  |
|  | |  |  |  | | | | | | | | |  | |  | | |  |
|  | |  |  |  | | | | | | | | |  | |  | | |  |
|  | |  |  |  | | | | | | | | |  | |  | | |  |
|  | |  |  |  | | | | | | | | |  | |  | | |  |
|  | |  |  |  | | | | | | | | |  | |  | | |  |
|  | |  |  |  | | | | | | | | |  | |  | | |  |
|  | |  |  |  | | | | | | | | |  | |  | | |  |
|  | |  |  |  | | | | | | | | |  | |  | | |  |
|  | |  |  |  | | | | | | | | |  | |  | | |  |
|  | |  |  |  | | | | | | | | |  | |  | | |  |
|  | |  |  |  | | | | | | | | |  | |  | | |  |
|  | |  |  |  | | | | | | | | |  | |  | | |  |
|  | |  |  |  | | | | | | | | |  | |  | | |  |
|  | |  |  |  | | | | | | | | |  | |  | | |  |
|  | |  |  |  | | | | | | | | |  | |  | | |  |
|  | |  |  |  | | | | | | | | |  | |  | | |  |
|  | |  |  |  | | | | | | | | |  | |  | | |  |
|  | |  |  |  | | | | | | | | |  | |  | | |  |
|  | |  |  |  | | | | | | | | |  | |  | | |  |
|  | |  |  |  | | | | | | | | |  | |  | | |  |
|  | |  |  |  | | | | | | | | |  | |  | | |  |
|  | |  |  |  | | | | | | | | |  | |  | | |  |
|  | |  |  |  | | | | | | | | |  | |  | | |  |
|  | |  |  |  | | | | | | | | |  | |  | | |  |
|  | |  |  |  | | | | | | | | |  | |  | | |  |
|  | |  |  |  | | | | | | | | |  | |  | | |  |
|  | |  |  |  | | | | | | | | |  | |  | | |  |
|  | |  |  |  | | | | | | | | |  | |  | | |  |
|  | |  |  |  | | | | | | | | |  | |  | | |  |
|  | |  |  |  | | | | | | | | |  | |  | | |  |
|  | |  |  |  | | | | | | | | |  | |  | | |  |
|  | |  |  |  | | | | | | | | |  | |  | | |  |
|  | |  |  |  | | | | | | | | |  | |  | | |  |
|  | |  |  |  | | | | | | | | |  | |  | | |  |
|  | |  |  |  | | | | | | | | |  | |  | | |  |
|  | |  |  |  | | | | | | | | |  | |  | | |  |
|  | |  |  |  | | | | | | | | |  | |  | | |  |
|  | |  |  |  | | | | | | | | |  | |  | | |  |
|  | |  |  |  | | | | | | | | |  | |  | | |  |
|  | |  |  |  | | | | | | | | |  | |  | | |  |
|  | |  |  |  | | | | | | | | |  | |  | | |  |
|  | |  |  |  | | | | | | | | |  | |  | | |  |
|  | |  |  |  | | | | | | | | |  | |  | | |  |
|  | |  |  |  | | | | | | | | |  | |  | | |  |
|  | |  |  |  | | | | | | | | |  | |  | | |  |
|  | |  |  |  | | | | | | | | |  | |  | | |  |
|  | |  |  |  | | | | | | | | |  | |  | | |  |
|  | |  |  |  | | | | | | | | |  | |  | | |  |
|  | |  |  |  | | | | | | | | |  | |  | | |  |
|  | |  |  |  | | | | | | | | |  | |  | | |  |
|  | |  |  |  | | | | | | | | |  | |  | | |  |
|  | |  |  |  | | | | | | | | |  | |  | | |  |
|  | |  |  |  | | | | | | | | |  | |  | | |  |
|  | |  |  |  | | | | | | | | |  | |  | | |  |
|  | |  |  |  | | | | | | | | |  | |  | | |  |
|  | |  |  |  | | | | | | | | |  | |  | | |  |
|  | |  |  |  | | | | | | | | |  | |  | | |  |
|  | |  |  |  | | | | | | | | |  | |  | | |  |
|  | |  |  |  | | | | | | | | |  | |  | | |  |
|  | |  |  |  | | | | | | | | |  | |  | | |  |
|  | |  |  |  | | | | | | | | |  | |  | | |  |
|  | |  |  |  | | | | | | | | |  | |  | | |  |
|  | |  |  |  | | | | | | | | |  | |  | | |  |
|  | |  |  |  | | | | | | | | |  | |  | | |  |
|  | |  |  |  | | | | | | | | |  | |  | | |  |
|  | |  |  |  | | | | | | | | |  | |  | | |  |
|  | |  |  |  | | | | | | | | |  | |  | | |  |
|  | |  |  |  | | | | | | | | |  | |  | | |  |
|  | |  |  |  | | | | | | | | |  | |  | | |  |
|  | |  |  |  | | | | | | | | |  | |  | | |  |
|  | |  |  |  | | | | | | | | |  | |  | | |  |
|  | |  |  |  | | | | | | | | |  | |  | | |  |
|  | |  |  |  | | | | | | | | |  | |  | | |  |
|  | |  |  |  | | | | | | | | |  | |  | | |  |
|  | |  |  |  | | | | | | | | |  | |  | | |  |
|  | |  |  |  | | | | | | | | |  | |  | | |  |
|  | |  |  |  | | | | | | | | |  | |  | | |  |
|  | |  |  |  | | | | | | | | |  | |  | | |  |
|  | |  |  |  | | | | | | | | |  | |  | | |  |
|  | |  |  |  | | | | | | | | |  | |  | | |  |
|  | |  |  |  | | | | | | | | |  | |  | | |  |
