# Supplementary material for: Relationship between Nonhepatic Serum Ammonia Levels and Sepsis-Associated Encephalopathy: A Retrospective Cohort Study
Source: Emerg Med Int. 2023 Oct 12;2023:6676033. doi: 10.1155/2023/6676033 (PMC10590267; doi:10.1155/2023/6676033)
Supplement: Supplementary Materials — 1: exclude patients with trauma of the skull from the MIMIC IV database according to ICD codes. Supplementary materials 2: exclude patients with intracerebral hemorrhage, cerebral embolism, and ischemic stroke disease from the MIMIC IV database according to ICD codes. Supplementary materials 3: exclude patients with meningitis and encephalitis disease from the MIMIC IV database according to ICD codes. Supplementary materials 4: exclude patients with epilepsy disease from the MIMIC IV database according to ICD codes. Supplementary materials 5: exclude patients with other cerebrovascular disease from the MIMIC IV database according to ICD codes. Supplementary materials 6: exclude patients with mental disorders and neurological disease from the MIMIC IV database according to ICD codes. Supplementary materials 7: exclude patients with alcoholic intoxication or drug abuse from the MIMIC IV database according to ICD codes. Supplementary materials 8: exclude patients with metabolic encephalopathy, hepatic encephalopathy, hypertensive encephalopathy, diabetes with coma, disorders of urea cycle, hypernatremia, and Wernicke's encephalopathy from the MIMIC IV database according to ICD codes. Supplementary materials 9: exclude patients with acute and chronic liver disease. Supplementary materials 10: hypertension disease and ICD codes. Supplementary materials 11: diabetes disease and ICD codes. Supplementary materials 12: lung disease and ICD codes. Supplementary materials 13: cardiovascular diseases and ICD codes. Supplementary materials 14: renal disease from the MIMIC IV database according to ICD codes. Supplementary materials 15: the standardized mean differences of the original cohort were compared with those of the IPW cohorts in sepsis patients. SMD: standardized mean differences. [file 6676033.f1.zip › Supplementary materials.4.docx]

| **Supplementary materials.4** Exclude patients with Epilepsy disease from the MIMIC IV database according to ICDcodes | | |
| --- | --- | --- |
| ICD-code | ICD | Description |
| 34591 | ICD9  ICD9  ICD9  ICD9  ICD9  ICD9  ICD9  ICD9  ICD9  ICD9  ICD9  ICD9 | Epilepsy, unspecified, with intractable epilepsy |
| 34570 |  | Epilepsia partialis continua, without mention of intractable epilepsy |
| 34571 |  | Epilepsia partialis continua, with intractable epilepsy |
| 34580 |  | Other forms of epilepsy and recurrent seizures, without mention of intractable epilepsy |
| 34581 |  | Other forms of epilepsy and recurrent seizures, with intractable epilepsy |
| 34590 |  | Epilepsy, unspecified, without mention of intractable epilepsy |
| 34591 |  | Epilepsy, unspecified, with intractable epilepsy |
| 64940 |  | Epilepsy complicating pregnancy, childbirth, or the puerperium, unspecified as to episode of care or not applicable |
| 64941 |  | Epilepsy complicating pregnancy, childbirth, or the puerperium, delivered, with or without mention of antepartum condition |
| 64942 |  | Epilepsy complicating pregnancy, childbirth, or the puerperium, delivered, with mention of postpartum complication |
| 64943 |  | Epilepsy complicating pregnancy, childbirth, or the puerperium, antepartum condition or complication |
| 64944 |  | Epilepsy complicating pregnancy, childbirth, or the puerperium, postpartum condition or complication |
| G40001 | ICD10 | Localization-related (focal) (partial) idiopathic epilepsy and epileptic syndromes with seizures of localized onset, not intractable, with status epilepticus |
| G40009 | ICD10 | Localization-related (focal) (partial) idiopathic epilepsy and epileptic syndromes with seizures of localized onset, not intractable, without status epilepticus |
| G40011 | ICD10 | Localization-related (focal) (partial) idiopathic epilepsy and epileptic syndromes with seizures of localized onset, intractable, with status epilepticus |
| G40019 | ICD10 | Localization-related (focal) (partial) idiopathic epilepsy and epileptic syndromes with seizures of localized onset, intractable, without status epilepticus |
| G40101 | ICD10 | Localization-related (focal) (partial) symptomatic epilepsy and epileptic syndromes with simple partial seizures, not intractable, with status epilepticus |
| G40109 | ICD10 | Localization-related (focal) (partial) symptomatic epilepsy and epileptic syndromes with simple partial seizures, not intractable, without status epilepticus |
| G40111 | ICD10 | Localization-related (focal) (partial) symptomatic epilepsy and epileptic syndromes with simple partial seizures, intractable, with status epilepticus |
| G40119 | ICD10 | Localization-related (focal) (partial) symptomatic epilepsy and epileptic syndromes with simple partial seizures, intractable, without status epilepticus |
| G40201 | ICD10 | Localization-related (focal) (partial) symptomatic epilepsy and epileptic syndromes with complex partial seizures, not intractable, with status epilepticus |
| G40209 | ICD10 | Localization-related (focal) (partial) symptomatic epilepsy and epileptic syndromes with complex partial seizures, not intractable, without status epilepticus |
| G40211 | ICD10 | Localization-related (focal) (partial) symptomatic epilepsy and epileptic syndromes with complex partial seizures, intractable, with status epilepticus |
| G40219 | ICD10 | Localization-related (focal) (partial) symptomatic epilepsy and epileptic syndromes with complex partial seizures, intractable, without status epilepticus |
| G40301 | ICD10 | Generalized idiopathic epilepsy and epileptic syndromes, not intractable, with status epilepticus |
| G40309 | ICD10 | Generalized idiopathic epilepsy and epileptic syndromes, not intractable, without status epilepticus |
| G40311 | ICD10 | Generalized idiopathic epilepsy and epileptic syndromes, intractable, with status epilepticus |
| G40319 | ICD10 | Generalized idiopathic epilepsy and epileptic syndromes, intractable, without status epilepticus |
| G40401 | ICD10 | Other generalized epilepsy and epileptic syndromes, not intractable, with status epilepticus |
| G40409 | ICD10 | Other generalized epilepsy and epileptic syndromes, not intractable, without status epilepticus |
| G40411 | ICD10 | Other generalized epilepsy and epileptic syndromes, intractable, with status epilepticus |
| G40419 | ICD10 | Other generalized epilepsy and epileptic syndromes, intractable, without status epilepticus |
| G40501 | ICD10 | Epileptic seizures related to external causes, not intractable, with status epilepticus |
| G40509 | ICD10 | Epileptic seizures related to external causes, not intractable, without status epilepticus |
| G40801 | ICD10 | Other epilepsy, not intractable, with status epilepticus |
| G40802 | ICD10 | Other epilepsy, not intractable, without status epilepticus |
| G40803 | ICD10 | Other epilepsy, intractable, with status epilepticus |
| G40804 | ICD10 | Other epilepsy, intractable, without status epilepticus |
| G40811 | ICD10 | Lennox-Gastaut syndrome, not intractable, with status epilepticus |
| G40812 | ICD10 | Lennox-Gastaut syndrome, not intractable, without status epilepticus |
| G40813 | ICD10 | Lennox-Gastaut syndrome, intractable, with status epilepticus |
| G40821 | ICD10 | Epileptic spasms, not intractable, with status epilepticus |
| G40822 | ICD10 | Epileptic spasms, not intractable, without status epilepticus |
| G40823 | ICD10 | Epileptic spasms, intractable, with status epilepticus |
| G40824 | ICD10 | Epileptic spasms, intractable, without status epilepticus |
| G4089 | ICD10 | Other seizures |
| G40901 | ICD10 | Epilepsy, unspecified, not intractable, with status epilepticus |
| G40909 | ICD10 | Epilepsy, unspecified, not intractable, without status epilepticus |
| G40911 | ICD10 | Epilepsy, unspecified, intractable, with status epilepticus |
| G40919 | ICD10 | Epilepsy, unspecified, intractable, without status epilepticus |
| G40A01 | ICD10 | Absence epileptic syndrome, not intractable, with status epilepticus |
| G40A09 | ICD10 | Absence epileptic syndrome, not intractable, without status epilepticus |
| G40A11 | ICD10 | Absence epileptic syndrome, intractable, with status epilepticus |
| G40A19 | ICD10 | Absence epileptic syndrome, intractable, without status epilepticus |
| G40B01 | ICD10 | Juvenile myoclonic epilepsy, not intractable, with status epilepticus |
| G40B09 | ICD10 | Juvenile myoclonic epilepsy, not intractable, without status epilepticus |
| G40B11 | ICD10 | Juvenile myoclonic epilepsy, intractable, with status epilepticus |
| G40B19 | ICD10 | Juvenile myoclonic epilepsy, intractable, without status epilepticus |
| G40A01 | ICD10 | Absence epileptic syndrome, not intractable, with status epilepticus |
| G40A09 | ICD10 | Absence epileptic syndrome, not intractable, without status epilepticus |
| G40A11 | ICD10 | Absence epileptic syndrome, intractable, with status epilepticus |
| G40A19 | ICD10 | Absence epileptic syndrome, intractable, without status epilepticus |
| G40B01 | ICD10 | Juvenile myoclonic epilepsy, not intractable, with status epilepticus |
| G40B09 | ICD10 | Juvenile myoclonic epilepsy, not intractable, without status epilepticus |
| G40B11 | ICD10 | Juvenile myoclonic epilepsy, intractable, with status epilepticus |
| G40B19 | ICD10 | Juvenile myoclonic epilepsy, intractable, without status epilepticus |
|  |  |  |
