# Supplementary material for: Relationship between Nonhepatic Serum Ammonia Levels and Sepsis-Associated Encephalopathy: A Retrospective Cohort Study
Source: Emerg Med Int. 2023 Oct 12;2023:6676033. doi: 10.1155/2023/6676033 (PMC10590267; doi:10.1155/2023/6676033)
Supplement: Supplementary Materials — 1: exclude patients with trauma of the skull from the MIMIC IV database according to ICD codes. Supplementary materials 2: exclude patients with intracerebral hemorrhage, cerebral embolism, and ischemic stroke disease from the MIMIC IV database according to ICD codes. Supplementary materials 3: exclude patients with meningitis and encephalitis disease from the MIMIC IV database according to ICD codes. Supplementary materials 4: exclude patients with epilepsy disease from the MIMIC IV database according to ICD codes. Supplementary materials 5: exclude patients with other cerebrovascular disease from the MIMIC IV database according to ICD codes. Supplementary materials 6: exclude patients with mental disorders and neurological disease from the MIMIC IV database according to ICD codes. Supplementary materials 7: exclude patients with alcoholic intoxication or drug abuse from the MIMIC IV database according to ICD codes. Supplementary materials 8: exclude patients with metabolic encephalopathy, hepatic encephalopathy, hypertensive encephalopathy, diabetes with coma, disorders of urea cycle, hypernatremia, and Wernicke's encephalopathy from the MIMIC IV database according to ICD codes. Supplementary materials 9: exclude patients with acute and chronic liver disease. Supplementary materials 10: hypertension disease and ICD codes. Supplementary materials 11: diabetes disease and ICD codes. Supplementary materials 12: lung disease and ICD codes. Supplementary materials 13: cardiovascular diseases and ICD codes. Supplementary materials 14: renal disease from the MIMIC IV database according to ICD codes. Supplementary materials 15: the standardized mean differences of the original cohort were compared with those of the IPW cohorts in sepsis patients. SMD: standardized mean differences. [file 6676033.f1.zip › Supplementary materials.5.docx]

|  | **Supplementary materials5** Exclude patients with other cerebrovascular disease from the MIMIC IV database according to ICD-codes | | | |
| --- | --- | --- | --- | --- |
|  | | ICD |  | Description |
| 3312 | | 9 |  | Senile degeneration of brain |
| 3313 | | 9 |  | Communicating hydrocephalus |
| 3314 | | 9 |  | Obstructive hydrocephalus |
| 33189 | | 9 |  | Other cerebral degeneration |
| 3319 | | 9 |  | Cerebral degeneration, unspecified |
| 3301 | | 9 |  | Cerebral lipidoses |
| 3302 | | 9 |  | Cerebral degeneration in generalized lipidoses |
| 3303 | | 9 |  | Cerebral degeneration of childhood in other diseases classified elsewhere |
| 3308 | | 9 |  | Other specified cerebral degenerations in childhood |
| 3309 | | 9 |  | Unspecified cerebral degeneration in childhood |
| 3310 | | 9 |  | Alzheimer's disease |
| 3317 | | 9 |  | Cerebral degeneration in diseases classified elsewhere |
| 33189 | | 9 |  | Other cerebral degeneration |
| 3319 | | 9 |  | Cerebral degeneration, unspecified |
|  | |  |  |  |
| 4378 | | 9 |  | Other ill-defined cerebrovascular disease |
| 4379 | | 9 |  | Unspecified cerebrovascular disease |
| 74100 | | 9 |  | Spina bifida with hydrocephalus, unspecified region |
| 74101 | | 9 |  | Spina bifida with hydrocephalus, cervical region |
| 74102 | | 9 |  | Spina bifida with hydrocephalus, dorsal (thoracic) region |
| 74103 | | 9 |  | Spina bifida with hydrocephalus, lumbar region |
| 74190 | | 9 |  | Spina bifida without mention of hydrocephalus, unspecified region |
| 74191 | | 9 |  | Spina bifida without mention of hydrocephalus, cervical region |
| 74192 | | 9 |  | Spina bifida without mention of hydrocephalus, dorsal (thoracic) region |
| 74193 | | 9 |  | Spina bifida without mention of hydrocephalus, lumbar region |
| 7423 | | 9 |  | Coxsackie myocarditis |
| 4380 | | 9 |  | Late effects of cerebrovascular disease, cognitive deficits |
| 43810 | | 9 |  | Late effects of cerebrovascular disease, speech and language deficit, unspecified |
| 43811 | | 9 |  | Late effects of cerebrovascular disease, aphasia |
| 43812 | | 9 |  | Late effects of cerebrovascular disease, dysphasia |
| 43813 | | 9 |  | Late effects of cerebrovascular disease, dysarthria |
| 43814 | | 9 |  | Late effects of cerebrovascular disease, fluency disorder |
| 43819 | | 9 |  | Late effects of cerebrovascular disease, other speech and language deficits |
| 43820 | | 9 |  | Late effects of cerebrovascular disease, hemiplegia affecting unspecified side |
| 43821 | | 9 |  | Late effects of cerebrovascular disease, hemiplegia affecting dominant side |
| 43822 | | 9 |  | Late effects of cerebrovascular disease, hemiplegia affecting nondominant side |
| 43830 | | 9 |  | Late effects of cerebrovascular disease, monoplegia of upper limb affecting unspecified side |
| 43831 | | 9 |  | Late effects of cerebrovascular disease, monoplegia of upper limb affecting dominant side |
| 43832 | | 9 |  | Late effects of cerebrovascular disease, monoplegia of upper limb affecting nondominant side |
| 43840 | | 9 |  | Late effects of cerebrovascular disease, monoplegia of lower limb affecting unspecified side |
| 43841 | | 9 |  | Late effects of cerebrovascular disease, monoplegia of lower limb affecting dominant side |
| 43842 | | 9 |  | Late effects of cerebrovascular disease, monoplegia of lower limb affecting nondominant side |
| 43850 | | 9 |  | Late effects of cerebrovascular disease, other paralytic syndrome affecting unspecified side |
| 43851 | | 9 |  | Late effects of cerebrovascular disease, other paralytic syndrome affecting dominant side |
| 43852 | | 9 |  | Late effects of cerebrovascular disease, other paralytic syndrome affecting nondominant side |
| 43853 | | 9 |  | Late effects of cerebrovascular disease, other paralytic syndrome, bilateral |
| 4386 | | 9 |  | Late effects of cerebrovascular disease, alterations of sensations |
| 4387 | | 9 |  | Late effects of cerebrovascular disease, disturbances of vision |
| 43881 | | 9 |  | Other late effects of cerebrovascular disease, apraxia |
| 43882 | | 9 |  | Other late effects of cerebrovascular disease, dysphagia |
| 43883 | | 9 |  | Other late effects of cerebrovascular disease, facial weakness |
| 43884 | | 9 |  | Other late effects of cerebrovascular disease, ataxia |
| 43885 | | 9 |  | Other late effects of cerebrovascular disease, vertigo |
| 43889 | | 9 |  | Other late effects of cerebrovascular disease |
| 4380 | | 9 |  | Unspecified cerebrovascular disease |
| 43810 | | 9 |  | Late effects of cerebrovascular disease, speech and language deficit, unspecified |
| G311 | | 10 |  | Senile degeneration of brain, not elsewhere classified |
| G910 | | 10 |  | Communicating hydrocephalus |
| G911 | | 10 |  | Obstructive hydrocephalus |
| G912 | | 10 |  | (Idiopathic) normal pressure hydrocephalus |
| G913 | | 10 |  | Post-traumatic hydrocephalus, unspecified |
| G914 | | 10 |  | Hydrocephalus in diseases classified elsewhere |
| Q038 | | 10 |  | Other congenital hydrocephalus |
| Q039 | | 10 |  | Congenital hydrocephalus, unspecified |
| Q050 | | 10 |  | Cervical spina bifida with hydrocephalus |
| Q051 | | 10 |  | Thoracic spina bifida with hydrocephalus |
| Q052 | | 10 |  | Lumbar spina bifida with hydrocephalus |
| Q053 | | 10 |  | Sacral spina bifida with hydrocephalus |
| Q054 | | 10 |  | Unspecified spina bifida with hydrocephalus |
| Q055 | | 10 |  | Cervical spina bifida without hydrocephalus |
| Q056 | | 10 |  | Thoracic spina bifida without hydrocephalus |
| Q057 | | 10 |  | Lumbar spina bifida without hydrocephalus |
| Q058 | | 10 |  | Sacral spina bifida without hydrocephalus |
| Q050 | | 10 |  | Cervical spina bifida with hydrocephalus |
| Q0700 | | 10 |  | Arnold-Chiari syndrome without spina bifida or hydrocephalus |
| Q0701 | | 10 |  | Arnold-Chiari syndrome with spina bifida |
| Q0702 | | 10 |  | Arnold-Chiari syndrome with hydrocephalus |
| Q0703 | | 10 |  | Arnold-Chiari syndrome with spina bifida and hydrocephalus |
| Q078 | | 10 |  | Other specified congenital malformations of nervous system |
| Q079 | | 10 |  | Congenital malformation of nervous system, unspecified |
| A066 | | 10 |  | Amebic brain abscess |
| A5482 | | 10 |  | Gonococcal brain abscess |
| B431 | | 10 |  | Pheomycotic brain abscess |
| C710 | | 10 |  | Malignant neoplasm of cerebrum, except lobes and ventricles |
| C711 | | 10 |  | Malignant neoplasm of frontal lobe |
| C712 | | 10 |  | Malignant neoplasm of temporal lobe |
| C713 | | 10 |  | Malignant neoplasm of parietal lobe |
| C714 | | 10 |  | Malignant neoplasm of occipital lobe |
| C715 | | 10 |  | Malignant neoplasm of cerebral ventricle |
| C716 | | 10 |  | Malignant neoplasm of cerebellum |
| C717 | | 10 |  | Malignant neoplasm of brain stem |
| C718 | | 10 |  | Malignant neoplasm of overlapping sites of brain |
| C719 | | 10 |  | Malignant neoplasm of brain, unspecified |
| C729 | | 10 |  | Malignant neoplasm of central nervous system, unspecified |
| C7932 | | 10 |  | Secondary malignant neoplasm of cerebral meninges |
| C7932 | | 10 |  | Secondary malignant neoplasm of cerebral meninges |
| C7940 | | 10 |  | Secondary malignant neoplasm of unspecified part of nervous system |
| C7949 | | 10 |  | Secondary malignant neoplasm of other parts of nervous system |
| D320 | | 10 |  | Benign neoplasm of cerebral meninges |
| D321 | | 10 |  | Benign neoplasm of spinal meninges |
| D329 | | 10 |  | Benign neoplasm of meninges, unspecified |
| D330 | | 10 |  | Benign neoplasm of brain, supratentorial |
| D331 | | 10 |  | Benign neoplasm of brain, infratentorial |
| D332 | | 10 |  | Benign neoplasm of brain, unspecified |
| D333 | | 10 |  | Benign neoplasm of cranial nerves |
| D337 | | 10 |  | Benign neoplasm of other specified parts of central nervous system |
| D339 | | 10 |  | Benign neoplasm of central nervous system, unspecified |
| D420 | | 10 |  | Neoplasm of uncertain behavior of cerebral meninges |
| D430 | | 10 |  | Neoplasm of uncertain behavior of brain, supratentorial |
| D431 | | 10 |  | Neoplasm of uncertain behavior of brain, infratentorial |
| D432 | | 10 |  | Neoplasm of uncertain behavior of brain, unspecified |
| D433 | | 10 |  | Neoplasm of uncertain behavior of cranial nerves |
| D438 | | 10 |  | Neoplasm of uncertain behavior of other specified parts of central nervous system |
| D439 | | 10 |  | Neoplasm of uncertain behavior of central nervous system, unspecified |
| D496 | | 10 |  | Neoplasm of unspecified behavior of brain |
| V1085 | | 9 |  | Personal history of malignant neoplasm of brain |
| V1086 | | 9 |  | Personal history of malignant neoplasm of other parts of nervous system |
| V1241 | | 9 |  | Personal history of benign neoplasm of the brain |
| V1242 | | 9 |  | Personal history of infections of the central nervous system |
| Z1282 | | 10 |  | Encounter for screening for malignant neoplasm of nervous system |
| Z85841 | | 10 |  | Personal history of malignant neoplasm of brain |
| Z8603 | | 10 |  | Personal history of neoplasm of uncertain behavior |
|  | |  |  |  |
