# Supplementary material for: Relationship between Nonhepatic Serum Ammonia Levels and Sepsis-Associated Encephalopathy: A Retrospective Cohort Study
Source: Emerg Med Int. 2023 Oct 12;2023:6676033. doi: 10.1155/2023/6676033 (PMC10590267; doi:10.1155/2023/6676033)
Supplement: Supplementary Materials — 1: exclude patients with trauma of the skull from the MIMIC IV database according to ICD codes. Supplementary materials 2: exclude patients with intracerebral hemorrhage, cerebral embolism, and ischemic stroke disease from the MIMIC IV database according to ICD codes. Supplementary materials 3: exclude patients with meningitis and encephalitis disease from the MIMIC IV database according to ICD codes. Supplementary materials 4: exclude patients with epilepsy disease from the MIMIC IV database according to ICD codes. Supplementary materials 5: exclude patients with other cerebrovascular disease from the MIMIC IV database according to ICD codes. Supplementary materials 6: exclude patients with mental disorders and neurological disease from the MIMIC IV database according to ICD codes. Supplementary materials 7: exclude patients with alcoholic intoxication or drug abuse from the MIMIC IV database according to ICD codes. Supplementary materials 8: exclude patients with metabolic encephalopathy, hepatic encephalopathy, hypertensive encephalopathy, diabetes with coma, disorders of urea cycle, hypernatremia, and Wernicke's encephalopathy from the MIMIC IV database according to ICD codes. Supplementary materials 9: exclude patients with acute and chronic liver disease. Supplementary materials 10: hypertension disease and ICD codes. Supplementary materials 11: diabetes disease and ICD codes. Supplementary materials 12: lung disease and ICD codes. Supplementary materials 13: cardiovascular diseases and ICD codes. Supplementary materials 14: renal disease from the MIMIC IV database according to ICD codes. Supplementary materials 15: the standardized mean differences of the original cohort were compared with those of the IPW cohorts in sepsis patients. SMD: standardized mean differences. [file 6676033.f1.zip › Supplementary materials.6.docx]

|  | **Supplementary materials.6** Exclude patients with mental disorders and and neurological disease from the MIMIC IVdatabase according to ICD-codes | | | | | |  |
| --- | --- | --- | --- | --- | --- | --- | --- |
|  | | ICD |  | Description |  |  | |
| 29634 | | 9 |  | Major depressive affective disorder, recurrent episode, severe, specified as with psychotic behavior |  |  | |
| 29635 | | 9 |  | Major depressive affective disorder, recurrent episode, in partial or unspecified remission |  |  | |
| 29636 | | 9 |  | Major depressive affective disorder, recurrent episode, in full remission |  |  | |
| 29640 | | 9 |  | Bipolar I disorder, most recent episode (or current) manic, unspecified |  |  | |
| 29641 | | 9 |  | Bipolar I disorder, most recent episode (or current) manic, mild |  |  | |
| 29642 | | 9 |  | Bipolar I disorder, most recent episode (or current) manic, moderate |  |  | |
| 29643 | | 9 |  | Bipolar I disorder, most recent episode (or current) manic, severe, without mention of psychotic behavior |  |  | |
| 29644 | | 9 |  | Bipolar I disorder, most recent episode (or current) manic, severe, specified as with psychotic behavior |  |  | |
| 29645 | | 9 |  | Bipolar I disorder, most recent episode (or current) manic, in partial or unspecified remission |  |  | |
| 29646 | | 9 |  | Bipolar I disorder, most recent episode (or current) manic, in full remission |  |  | |
| 29650 | | 9 |  | Bipolar I disorder, most recent episode (or current) depressed, unspecified |  |  | |
| 29651 | | 9 |  | Bipolar I disorder, most recent episode (or current) depressed, mild |  |  | |
| 29652 | | 9 |  | Bipolar I disorder, most recent episode (or current) depressed, moderate |  |  | |
| 29653 | | 9 |  | Bipolar I disorder, most recent episode (or current) depressed, severe, without mention of psychotic behavior |  |  | |
| 29654 | | 9 |  | Bipolar I disorder, most recent episode (or current) depressed, severe, specified as with psychotic behavior |  |  | |
| 29655 | | 9 |  | Bipolar I disorder, most recent episode (or current) depressed, in partial or unspecified remission |  |  | |
| 29656 | | 9 |  | Bipolar I disorder, most recent episode (or current) depressed, in full remission |  |  | |
| 29660 | | 9 |  | Bipolar I disorder, most recent episode (or current) mixed, unspecified |  |  | |
| 29661 | | 9 |  | Bipolar I disorder, most recent episode (or current) mixed, mild |  |  | |
| 29662 | | 9 |  | Bipolar I disorder, most recent episode (or current) mixed, moderate |  |  | |
| 29620 | | 9 |  | Major depressive affective disorder, single episode, unspecified |  |  | |
| 29621 | | 9 |  | Major depressive affective disorder, single episode, mild |  |  | |
| 29622 | | 9 |  | Major depressive affective disorder, single episode, moderate |  |  | |
| 29623 | | 9 |  | Major depressive affective disorder, single episode, severe, without mention of psychotic behavior |  |  | |
| 29624 | | 9 |  | Major depressive affective disorder, single episode, severe, specified as with psychotic behavior |  |  | |
| 29625 | | 9 |  | Major depressive affective disorder, single episode, in partial or unspecified remission |  |  | |
| 29626 | | 9 |  | Major depressive affective disorder, single episode, in full remission |  |  | |
| 29630 | | 9 |  | Major depressive affective disorder, recurrent episode, unspecified |  |  | |
| 29631 | | 9 |  | Major depressive affective disorder, recurrent episode, mild |  |  | |
| 29632 | | 9 |  | Major depressive affective disorder, recurrent episode, moderate |  |  | |
| 29633 | | 9 |  | Major depressive affective disorder, recurrent episode, severe, without mention of psychotic behavior |  |  | |
| 29634 | | 9 |  | Major depressive affective disorder, recurrent episode, severe, specified as with psychotic behavior |  |  | |
| 29635 | | 9 |  | Major depressive affective disorder, recurrent episode, in partial or unspecified remission |  |  | |
| 29636 | | 9 |  | Major depressive affective disorder, recurrent episode, in full remission |  |  | |
| 29663 | | 9 |  | Bipolar I disorder, most recent episode (or current) mixed, severe, without mention of psychotic behavior |  |  | |
| 29664 | | 9 |  | Bipolar I disorder, most recent episode (or current) mixed, severe, specified as with psychotic behavior |  |  | |
| 29665 | | 9 |  | Bipolar I disorder, most recent episode (or current) mixed, in partial or unspecified remission |  |  | |
| 29666 | | 9 |  | Bipolar I disorder, most recent episode (or current) mixed, in full remission |  |  | |
| 2967 | | 9 |  | Bipolar I disorder, most recent episode (or current) unspecified |  |  | |
| 29680 | | 9 |  | Bipolar disorder, unspecified |  |  | |
| 29681 | | 9 |  | Atypical manic disorder |  |  | |
| 29682 | | 9 |  | Atypical depressive disorder |  |  | |
| 29689 | | 9 |  | Other bipolar disorders |  |  | |
| 30289 | | 9 |  | Other specified psychosexual disorders |  |  | |
| 3029 | | 9 |  | Unspecified psychosexual disorder |  |  | |
| 29682 | | 9 |  | Atypical depressive disorder |  |  | |
| 2971 | | 9 |  | Delusional disorder |  |  | |
| 2972 | | 9 |  | Paraphrenia |  |  | |
| 2980 | | 9 |  | Depressive type psychosis |  |  | |
| 2981 | | 9 |  | Excitative type psychosis |  |  | |
| 2982 | | 9 |  | Reactive confusion |  |  | |
| 2983 | | 9 |  | Acute paranoid reaction |  |  | |
| 2984 | | 9 |  | Psychogenic paranoid psychosis |  |  | |
| 2988 | | 9 |  | Other and unspecified reactive psychosis |  |  | |
| 2989 | | 9 |  | Unspecified psychosis |  |  | |
| 30111 | | 9 |  | Chronic hypomanic personality disorder |  |  | |
| 30020 | | 9 |  | Phobia, unspecified |  |  | |
| 30021 | | 9 |  | Agoraphobia with panic disorder |  |  | |
| 30022 | | 9 |  | Agoraphobia without mention of panic attacks |  |  | |
| 30023 | | 9 |  | Social phobia |  |  | |
| 30029 | | 9 |  | Other isolated or specific phobias |  |  | |
| 30112 | | 9 |  | Chronic depressive personality disorder |  |  | |
| 30113 | | 9 |  | Cyclothymic disorder |  |  | |
| 30120 | | 9 |  | Schizoid personality disorder, unspecified |  |  | |
| 29381 | | 9 |  | Psychotic disorder with delusions in conditions classified elsewhere |  |  | |
| 29410 | | 9 |  | Dementia in conditions classified elsewhere without behavioral disturbance |  |  | |
| 29411 | | 9 |  | Dementia in conditions classified elsewhere with behavioral disturbance |  |  | |
| 29420 | | 9 |  | Dementia, unspecified, without behavioral disturbance |  |  | |
| 29421 | | 9 |  | Dementia, unspecified, with behavioral disturbance |  |  | |
| 2949 | | 9 |  | Unspecified persistent mental disorders due to conditions classified elsewhere |  |  | |
| 29500 | | 9 |  | Simple type schizophrenia, unspecified |  |  | |
| 29501 | | 9 |  | Simple type schizophrenia, subchronic |  |  | |
| 29502 | | 9 |  | Simple type schizophrenia, chronic |  |  | |
| 29503 | | 9 |  | Simple type schizophrenia, subchronic with acute exacerbation |  |  | |
| 29504 | | 9 |  | Simple type schizophrenia, chronic with acute exacerbation |  |  | |
| 29505 | | 9 |  | Simple type schizophrenia, in remission |  |  | |
| 29510 | | 9 |  | Disorganized type schizophrenia, unspecified |  |  | |
| 29511 | | 9 |  | Disorganized type schizophrenia, subchronic |  |  | |
| 29512 | | 9 |  | Disorganized type schizophrenia, chronic |  |  | |
| 29513 | | 9 |  | Disorganized type schizophrenia, subchronic with acute exacerbation |  |  | |
| 29514 | | 9 |  | Disorganized type schizophrenia, chronic with acute exacerbation |  |  | |
| 29515 | | 9 |  | Disorganized type schizophrenia, in remission |  |  | |
| 29520 | | 9 |  | Catatonic type schizophrenia, unspecified |  |  | |
| 29521 | | 9 |  | Catatonic type schizophrenia, subchronic |  |  | |
| 29522 | | 9 |  | Catatonic type schizophrenia, chronic |  |  | |
| 29523 | | 9 |  | Catatonic type schizophrenia, subchronic with acute exacerbation |  |  | |
| 29524 | | 9 |  | Catatonic type schizophrenia, chronic with acute exacerbation |  |  | |
| 29525 | | 9 |  | Catatonic type schizophrenia, in remission |  |  | |
| 29530 | | 9 |  | Paranoid type schizophrenia, unspecified |  |  | |
| 29531 | | 9 |  | Paranoid type schizophrenia, subchronic |  |  | |
| 29532 | | 9 |  | Paranoid type schizophrenia, chronic |  |  | |
| 29533 | | 9 |  | Paranoid type schizophrenia, subchronic with acute exacerbation |  |  | |
| 29534 | | 9 |  | Paranoid type schizophrenia, chronic with acute exacerbation |  |  | |
| 29535 | | 9 |  | Paranoid type schizophrenia, in remission |  |  | |
| 29540 | | 9 |  | Schizophreniform disorder, unspecified |  |  | |
| 29541 | | 9 |  | Schizophreniform disorder, subchronic |  |  | |
| 29542 | | 9 |  | Schizophreniform disorder, chronic |  |  | |
| 29543 | | 9 |  | Schizophreniform disorder, subchronic with acute exacerbation |  |  | |
| 29544 | | 9 |  | Schizophreniform disorder, chronic with acute exacerbation |  |  | |
| 29545 | | 9 |  | Schizophreniform disorder, in remission |  |  | |
| 29550 | | 9 |  | Latent schizophrenia, unspecified |  |  | |
| 29551 | | 9 |  | Latent schizophrenia, unspecified |  |  | |
| 29552 | | 9 |  | Latent schizophrenia, chronic |  |  | |
| 29553 | | 9 |  | Latent schizophrenia, subchronic with acute exacerbation |  |  | |
| 29554 | | 9 |  | Latent schizophrenia, chronic with acute exacerbation |  |  | |
| 29555 | | 9 |  | Latent schizophrenia, in remission |  |  | |
| 29560 | | 9 |  | Schizophrenic disorders, residual type, unspecified |  |  | |
| 29561 | | 9 |  | Schizophrenic disorders, residual type, subchronic |  |  | |
| 29562 | | 9 |  | Schizophrenic disorders, residual type, chronic |  |  | |
| 29563 | | 9 |  | Schizophrenic disorders, residual type, subchronic with acute exacerbation |  |  | |
| 29564 | | 9 |  | Schizophrenic disorders, residual type, chronic with acute exacerbation |  |  | |
| 29565 | | 9 |  | Schizophrenic disorders, residual type, in remission |  |  | |
| 29570 | | 9 |  | Schizoaffective disorder, unspecified |  |  | |
| 29571 | | 9 |  | Schizoaffective disorder, subchronic |  |  | |
| 29572 | | 9 |  | Schizoaffective disorder, chronic |  |  | |
| 29573 | | 9 |  | Schizoaffective disorder, subchronic with acute exacerbation |  |  | |
| 29574 | | 9 |  | Schizoaffective disorder, chronic with acute exacerbation |  |  | |
| 29580 | | 9 |  | Other specified types of schizophrenia, unspecified |  |  | |
| 29581 | | 9 |  | Other specified types of schizophrenia, subchronic |  |  | |
| 29582 | | 9 |  | Other specified types of schizophrenia, chronic |  |  | |
| 29583 | | 9 |  | Other specified types of schizophrenia, subchronic with acute exacerbation |  |  | |
| 29584 | | 9 |  | Other specified types of schizophrenia, chronic with acute exacerbation |  |  | |
| 29585 | | 9 |  | Other specified types of schizophrenia, in remission |  |  | |
| 29590 | | 9 |  | Unspecified schizophrenia, unspecified |  |  | |
| 29591 | | 9 |  | Unspecified schizophrenia, subchronic |  |  | |
| 29592 | | 9 |  | Unspecified schizophrenia, chronic |  |  | |
| 29593 | | 9 |  | Unspecified schizophrenia, subchronic with acute exacerbation |  |  | |
| 29594 | | 9 |  | Unspecified schizophrenia, chronic with acute exacerbation |  |  | |
| 29595 | | 9 |  | Unspecified schizophrenia, in remission |  |  | |
| 29600 | | 9 |  | Bipolar I disorder, single manic episode, unspecified |  |  | |
| 29601 | | 9 |  | Bipolar I disorder, single manic episode, mild |  |  | |
| 29602 | | 9 |  | Bipolar I disorder, single manic episode, moderate |  |  | |
| 29603 | | 9 |  | Bipolar I disorder, single manic episode, severe, without mention of psychotic behavior |  |  | |
| 29604 | | 9 |  | Bipolar I disorder, single manic episode, severe, specified as with psychotic behavior |  |  | |
| 29605 | | 9 |  | Bipolar I disorder, single manic episode, in partial or unspecified remission |  |  | |
| 29606 | | 9 |  | Bipolar I disorder, single manic episode, in full remission |  |  | |
| 29610 | | 9 |  | Manic affective disorder, recurrent episode, unspecified |  |  | |
| 29611 | | 9 |  | Manic affective disorder, recurrent episode, mild |  |  | |
| 29612 | | 9 |  | Manic affective disorder, recurrent episode, moderate |  |  | |
| 29613 | | 9 |  | Manic affective disorder, recurrent episode, severe, without mention of psychotic behavior |  |  | |
| 29614 | | 9 |  | Manic affective disorder, recurrent episode, severe, specified as with psychotic behavior |  |  | |
| 29615 | | 9 |  | Manic affective disorder, recurrent episode, in partial or unspecified remission |  |  | |
| 29616 | | 9 |  | Manic affective disorder, recurrent episode, in full remission |  |  | |
| 29620 | | 9 |  | Major depressive affective disorder, single episode, unspecified |  |  | |
| 29621 | | 9 |  | Major depressive affective disorder, single episode, mild |  |  | |
| 29622 | | 9 |  | Major depressive affective disorder, single episode, moderate |  |  | |
| 29623 | | 9 |  | Major depressive affective disorder, single episode, severe, without mention of psychotic behavior |  |  | |
| 29624 | | 9 |  | Major depressive affective disorder, single episode, severe, specified as with psychotic behavior |  |  | |
| 29625 | | 9 |  | Major depressive affective disorder, single episode, in partial or unspecified remission |  |  | |
| 29626 | | 9 |  | Major depressive affective disorder, single episode, in full remission |  |  | |
| 29630 | | 9 |  | Major depressive affective disorder, recurrent episode, unspecified |  |  | |
| 29631 | | 9 |  | Major depressive affective disorder, recurrent episode, mild |  |  | |
| 29632 | | 9 |  | Major depressive affective disorder, recurrent episode, moderate |  |  | |
| 29633 | | 9 |  | Major depressive affective disorder, recurrent episode, severe, without mention of psychotic behavior |  |  | |
| 33182 | | 9 |  | Dementia with lewy bodies |  |  | |
| 3310 | | 9 |  | Alzheimer's disease |  |  | |
| 33119 | | 9 |  | Other frontotemporal dementia |  |  | |
| 64842 | | 9 |  | Mental disorders of mother, delivered, with mention of postpartum complication |  |  | |
| 64843 | | 9 |  | Mental disorders of mother, antepartum condition or complication |  |  | |
| 64844 | | 9 |  | Mental disorders of mother, postpartum condition or complication |  |  | |
| 3181 | | 9 |  | Severe intellectual disabilities |  |  | |
| 3182 | | 9 |  | Profound intellectual disabilities |  |  | |
| 33182 | | 9 |  | Dementia with lewy bodies |  |  | |
| F0150 | | 10 |  | Vascular dementia without behavioral disturbance |  |  | |
| F0151 | | 10 |  | Vascular dementia with behavioral disturbance |  |  | |
| F0280 | | 10 |  | Dementia in other diseases classified elsewhere without behavioral disturbance |  |  | |
| F0281 | | 10 |  | Dementia in other diseases classified elsewhere with behavioral disturbance |  |  | |
| F0390 | | 10 |  | Unspecified dementia without behavioral disturbance |  |  | |
| F0391 | | 10 |  | Unspecified dementia with behavioral disturbance |  |  | |
| F04 | | 10 |  | Amnestic disorder due to known physiological condition |  |  | |
| F05 | | 10 |  | Delirium due to known physiological condition |  |  | |
| F060 | | 10 |  | Psychotic disorder with hallucinations due to known physiological condition |  |  | |
| F061 | | 10 |  | Catatonic disorder due to known physiological condition |  |  | |
| F062 | | 10 |  | Psychotic disorder with delusions due to known physiological condition |  |  | |
| F0630 | | 10 |  | Mood disorder due to known physiological condition, unspecified |  |  | |
| F0631 | | 10 |  | Mood disorder due to known physiological condition with depressive features |  |  | |
| F0632 | | 10 |  | Mood disorder due to known physiological condition with major depressive-like episode |  |  | |
| F0633 | | 10 |  | Mood disorder due to known physiological condition with manic features |  |  | |
| F0634 | | 10 |  | Mood disorder due to known physiological condition with mixed features |  |  | |
| F064 | | 10 |  | Anxiety disorder due to known physiological condition |  |  | |
| F068 | | 10 |  | Other specified mental disorders due to known physiological condition |  |  | |
| F070 | | 10 |  | Personality change due to known physiological condition |  |  | |
| F0781 | | 10 |  | Postconcussional syndrome |  |  | |
| F0789 | | 10 |  | Other personality and behavioral disorders due to known physiological condition |  |  | |
| F079 | | 10 |  | Unspecified personality and behavioral disorder due to known physiological condition |  |  | |
| F09 | | 10 |  | Unspecified mental disorder due to known physiological condition |  |  | |
| F200 | | 10 |  | Paranoid schizophrenia |  |  | |
| F201 | | 10 |  | Disorganized schizophrenia |  |  | |
| F202 | | 10 |  | Catatonic schizophrenia |  |  | |
| F203 | | 10 |  | Undifferentiated schizophrenia |  |  | |
| F205 | | 10 |  | Residual schizophrenia |  |  | |
| F2081 | | 10 |  | Schizophreniform disorder |  |  | |
| F2089 | | 10 |  | Other schizophrenia |  |  | |
| F209 | | 10 |  | Schizophrenia, unspecified |  |  | |
| F21 | | 10 |  | Schizotypal disorder |  |  | |
| F22 | | 10 |  | Delusional disorders |  |  | |
| F23 | | 10 |  | Brief psychotic disorder |  |  | |
| F24 | | 10 |  | Shared psychotic disorder |  |  | |
| F250 | | 10 |  | Schizoaffective disorder, bipolar type |  |  | |
| F251 | | 10 |  | Schizoaffective disorder, depressive type |  |  | |
| F258 | | 10 |  | Other schizoaffective disorders |  |  | |
| F259 | | 10 |  | Schizoaffective disorder, unspecified |  |  | |
| F28 | | 10 |  | Other psychotic disorder not due to a substance or known physiological condition |  |  | |
| F29 | | 10 |  | Unspecified psychosis not due to a substance or known physiological condition |  |  | |
| F3010 | | 10 |  | Manic episode without psychotic symptoms, unspecified |  |  | |
| F3011 | | 10 |  | Manic episode without psychotic symptoms, mild |  |  | |
| F3012 | | 10 |  | Manic episode without psychotic symptoms, moderate |  |  | |
| F3013 | | 10 |  | Manic episode, severe, without psychotic symptoms |  |  | |
| F302 | | 10 |  | Manic episode, severe with psychotic symptoms |  |  | |
| F303 | | 10 |  | Manic episode in partial remission |  |  | |
| F304 | | 10 |  | Manic episode in full remission |  |  | |
| F308 | | 10 |  | Other manic episodes |  |  | |
| F309 | | 10 |  | Manic episode, unspecified |  |  | |
| F310 | | 10 |  | Bipolar disorder, current episode hypomanic |  |  | |
| F3110 | | 10 |  | Bipolar disorder, current episode manic without psychotic features, unspecified |  |  | |
| F3111 | | 10 |  | Bipolar disorder, current episode manic without psychotic features, mild |  |  | |
| F3112 | | 10 |  | Bipolar disorder, current episode manic without psychotic features, moderate |  |  | |
| F3113 | | 10 |  | Bipolar disorder, current episode manic without psychotic features, severe |  |  | |
| F312 | | 10 |  | Bipolar disorder, current episode manic severe with psychotic features |  |  | |
| F3130 | | 10 |  | Bipolar disorder, current episode depressed, mild or moderate severity, unspecified |  |  | |
| F3131 | | 10 |  | Bipolar disorder, current episode depressed, mild |  |  | |
| F3132 | | 10 |  | Bipolar disorder, current episode depressed, moderate |  |  | |
| F314 | | 10 |  | Bipolar disorder, current episode depressed, severe, without psychotic features |  |  | |
| F315 | | 10 |  | Bipolar disorder, current episode depressed, severe, with psychotic features |  |  | |
| F3160 | | 10 |  | Bipolar disorder, current episode mixed, unspecified |  |  | |
| F3161 | | 10 |  | Bipolar disorder, current episode mixed, mild |  |  | |
| F3162 | | 10 |  | Bipolar disorder, current episode mixed, moderate |  |  | |
| F3163 | | 10 |  | Bipolar disorder, current episode mixed, severe, without psychotic features |  |  | |
| F3164 | | 10 |  | Bipolar disorder, current episode mixed, severe, with psychotic features |  |  | |
| F3170 | | 10 |  | Bipolar disorder, currently in remission, most recent episode unspecified |  |  | |
| F3171 | | 10 |  | Bipolar disorder, in partial remission, most recent episode hypomanic |  |  | |
| F3172 | | 10 |  | Bipolar disorder, in full remission, most recent episode hypomanic |  |  | |
| F3173 | | 10 |  | Bipolar disorder, in partial remission, most recent episode manic |  |  | |
| F3174 | | 10 |  | Bipolar disorder, in full remission, most recent episode manic |  |  | |
| F3175 | | 10 |  | Bipolar disorder, in partial remission, most recent episode depressed |  |  | |
| F3176 | | 10 |  | Bipolar disorder, in full remission, most recent episode depressed |  |  | |
| F3177 | | 10 |  | Bipolar disorder, in partial remission, most recent episode mixed |  |  | |
| F3178 | | 10 |  | Bipolar disorder, in full remission, most recent episode mixed |  |  | |
| F3181 | | 10 |  | Bipolar II disorder |  |  | |
| F3189 | | 10 |  | Other bipolar disorder |  |  | |
| F319 | | 10 |  | Bipolar disorder, unspecified |  |  | |
| F320 | | 10 |  | Major depressive disorder, single episode, mild |  |  | |
| F321 | | 10 |  | Major depressive disorder, single episode, moderate |  |  | |
| F322 | | 10 |  | Major depressive disorder, single episode, severe without psychotic features |  |  | |
| F323 | | 10 |  | Major depressive disorder, single episode, severe with psychotic features |  |  | |
| F324 | | 10 |  | Major depressive disorder, single episode, in partial remission |  |  | |
| F325 | | 10 |  | Major depressive disorder, single episode, in full remission |  |  | |
| F3281 | | 10 |  | Premenstrual dysphoric disorder |  |  | |
| F3289 | | 10 |  | Other specified depressive episodes |  |  | |
| F329 | | 10 |  | Major depressive disorder, single episode, unspecified |  |  | |
| F330 | | 10 |  | Major depressive disorder, recurrent, mild |  |  | |
| F331 | | 10 |  | Major depressive disorder, recurrent, moderate |  |  | |
| F332 | | 10 |  | Major depressive disorder, recurrent severe without psychotic features |  |  | |
| F333 | | 10 |  | Major depressive disorder, recurrent, severe with psychotic symptoms |  |  | |
| F3340 | | 10 |  | Major depressive disorder, recurrent, in remission, unspecified |  |  | |
| F3341 | | 10 |  | Major depressive disorder, recurrent, in partial remission |  |  | |
| F3342 | | 10 |  | Major depressive disorder, recurrent, in full remission |  |  | |
| F338 | | 10 |  | Other recurrent depressive disorders |  |  | |
| F340 | | 10 |  | Cyclothymic disorder |  |  | |
| F341 | | 10 |  | Dysthymic disorder |  |  | |
| F3481 | | 10 |  | Disruptive mood dysregulation disorder |  |  | |
| F3489 | | 10 |  | Other specified persistent mood disorders |  |  | |
| F349 | | 10 |  | Persistent mood [affective] disorder, unspecified |  |  | |
| F39 | | 10 |  | Unspecified mood [affective] disorder |  |  | |
| F4000 | | 10 |  | Agoraphobia, unspecified |  |  | |
| F4001 | | 10 |  | Agoraphobia with panic disorder |  |  | |
| F4002 | | 10 |  | Agoraphobia without panic disorder |  |  | |
| F4010 | | 10 |  | Social phobia, unspecified |  |  | |
| F4011 | | 10 |  | Social phobia, generalized |  |  | |
| F40248 | | 10 |  | Other situational type phobia |  |  | |
| F40290 | | 10 |  | Androphobia |  |  | |
| F40291 | | 10 |  | Gynephobia |  |  | |
| F40298 | | 10 |  | Other specified phobia |  |  | |
| F408 | | 10 |  | Other phobic anxiety disorders |  |  | |
| F409 | | 10 |  | Phobic anxiety disorder, unspecified |  |  | |
| F410 | | 10 |  | Panic disorder [episodic paroxysmal anxiety] |  |  | |
| F411 | | 10 |  | Generalized anxiety disorder |  |  | |
| F413 | | 10 |  | Other mixed anxiety disorders |  |  | |
| F418 | | 10 |  | Other specified anxiety disorders |  |  | |
| F419 | | 10 |  | Anxiety disorder, unspecified |  |  | |
| F422 | | 10 |  | Mixed obsessional thoughts and acts |  |  | |
| F423 | | 10 |  | Hoarding disorder |  |  | |
| F424 | | 10 |  | Excoriation (skin-picking) disorder |  |  | |
| F428 | | 10 |  | Other obsessive-compulsive disorder |  |  | |
| F429 | | 10 |  | Obsessive-compulsive disorder, unspecified |  |  | |
| F430 | | 10 |  | Acute stress reaction |  |  | |
| F4310 | | 10 |  | Post-traumatic stress disorder, unspecified |  |  | |
| F4311 | | 10 |  | Post-traumatic stress disorder, acute |  |  | |
| F4312 | | 10 |  | Post-traumatic stress disorder, chronic |  |  | |
| F4320 | | 10 |  | Adjustment disorder, unspecified |  |  | |
| F4321 | | 10 |  | Adjustment disorder with depressed mood |  |  | |
| F4322 | | 10 |  | Adjustment disorder with anxiety |  |  | |
| F4323 | | 10 |  | Adjustment disorder with mixed anxiety and depressed mood |  |  | |
| F4324 | | 10 |  | Adjustment disorder with disturbance of conduct |  |  | |
| F4325 | | 10 |  | Adjustment disorder with mixed disturbance of emotions and conduct |  |  | |
| F4329 | | 10 |  | Adjustment disorder with other symptoms |  |  | |
| F70 | | 10 |  | Mild intellectual disabilities |  |  | |
| F71 | | 10 |  | Moderate intellectual disabilities |  |  | |
| F72 | | 10 |  | Severe intellectual disabilities |  |  | |
| F73 | | 10 |  | Profound intellectual disabilities |  |  | |
| F78 | | 10 |  | Other intellectual disabilities |  |  | |
| F79 | | 10 |  | Unspecified intellectual disabilities |  |  | |
| F800 | | 10 |  | Phonological disorder |  |  | |
| F801 | | 10 |  | Expressive language disorder |  |  | |
| F802 | | 10 |  | Mixed receptive-expressive language disorder |  |  | |
| F804 | | 10 |  | Speech and language development delay due to hearing loss |  |  | |
| F8081 | | 10 |  | Childhood onset fluency disorder |  |  | |
| F8082 | | 10 |  | Social pragmatic communication disorder |  |  | |
| F8089 | | 10 |  | Other developmental disorders of speech and language |  |  | |
| F809 | | 10 |  | Developmental disorder of speech and language, unspecified |  |  | |
| F812 | | 10 |  | Mathematics disorder |  |  | |
| F8181 | | 10 |  | Disorder of written expression |  |  | |
| G300 | | 10 |  | Alzheimer's disease with early onset |  |  | |
| G301 | | 10 |  | Alzheimer's disease with late onset |  |  | |
| G308 | | 10 |  | Other Alzheimer's disease |  |  | |
| G309 | | 10 |  | Alzheimer's disease, unspecified |  |  | |
| G3101 | | 10 |  | Pick's disease |  |  | |
| G3109 | | 10 |  | Other frontotemporal dementia |  |  | |
| G311 | | 10 |  | Senile degeneration of brain, not elsewhere classified |  |  | |
| G312 | | 10 |  | Degeneration of nervous system due to alcohol |  |  | |
| G3101 | | 10 |  | Pick's disease |  |  | |
| G3109 | | 10 |  | Other frontotemporal dementia |  |  | |
| G311 | | 10 |  | Senile degeneration of brain, not elsewhere classified |  |  | |
| G312 | | 10 |  | Degeneration of nervous system due to alcohol |  |  | |
| G3181 | | 10 |  | Alpers disease |  |  | |
| G3182 | | 10 |  | Leigh's disease |  |  | |
| G3183 | | 10 |  | Dementia with Lewy bodies |  |  | |
| G360 | | 10 |  | Neuromyelitis optica [Devic] |  |  | |
| G361 | | 10 |  | Acute and subacute hemorrhagic leukoencephalitis [Hurst] |  |  | |
| G368 | | 10 |  | Other specified acute disseminated demyelination |  |  | |
| G369 | | 10 |  | Acute disseminated demyelination, unspecified |  |  | |
| G370 | | 10 |  | Diffuse sclerosis of central nervous system |  |  | |
| G371 | | 10 |  | Central demyelination of corpus callosum |  |  | |
| G372 | | 10 |  | Central pontine myelinolysis |  |  | |
| G373 | | 10 |  | Acute transverse myelitis in demyelinating disease of central nervous system |  |  | |
| G374 | | 10 |  | Subacute necrotizing myelitis of central nervous system |  |  | |
| G375 | | 10 |  | Concentric sclerosis [Balo] of central nervous system |  |  | |
| G378 | | 10 |  | Other specified demyelinating diseases of central nervous system |  |  | |
| G379 | | 10 |  | Demyelinating disease of central nervous system, unspecified |  |  | |
|  | |  |  |  |  |  | |
