# Supplementary material for: Relationship between Nonhepatic Serum Ammonia Levels and Sepsis-Associated Encephalopathy: A Retrospective Cohort Study
Source: Emerg Med Int. 2023 Oct 12;2023:6676033. doi: 10.1155/2023/6676033 (PMC10590267; doi:10.1155/2023/6676033)
Supplement: Supplementary Materials — 1: exclude patients with trauma of the skull from the MIMIC IV database according to ICD codes. Supplementary materials 2: exclude patients with intracerebral hemorrhage, cerebral embolism, and ischemic stroke disease from the MIMIC IV database according to ICD codes. Supplementary materials 3: exclude patients with meningitis and encephalitis disease from the MIMIC IV database according to ICD codes. Supplementary materials 4: exclude patients with epilepsy disease from the MIMIC IV database according to ICD codes. Supplementary materials 5: exclude patients with other cerebrovascular disease from the MIMIC IV database according to ICD codes. Supplementary materials 6: exclude patients with mental disorders and neurological disease from the MIMIC IV database according to ICD codes. Supplementary materials 7: exclude patients with alcoholic intoxication or drug abuse from the MIMIC IV database according to ICD codes. Supplementary materials 8: exclude patients with metabolic encephalopathy, hepatic encephalopathy, hypertensive encephalopathy, diabetes with coma, disorders of urea cycle, hypernatremia, and Wernicke's encephalopathy from the MIMIC IV database according to ICD codes. Supplementary materials 9: exclude patients with acute and chronic liver disease. Supplementary materials 10: hypertension disease and ICD codes. Supplementary materials 11: diabetes disease and ICD codes. Supplementary materials 12: lung disease and ICD codes. Supplementary materials 13: cardiovascular diseases and ICD codes. Supplementary materials 14: renal disease from the MIMIC IV database according to ICD codes. Supplementary materials 15: the standardized mean differences of the original cohort were compared with those of the IPW cohorts in sepsis patients. SMD: standardized mean differences. [file 6676033.f1.zip › Supplementary materials.7.docx]

|  | **Supplementary materials7** Exclude patients with alcoholic intoxication or or drug abuse from the MIMIC IV database according to ICD-codes | | | |  |
| --- | --- | --- | --- | --- | --- |
|  | | ICD |  | Description |  |
| 30303 | | 9 |  | Acute alcoholic intoxication in alcoholism, in remission |  |
| 30390 | | 9 |  | Other and unspecified alcohol dependence, unspecified |  |
| 30391 | | 9 |  | Other and unspecified alcohol dependence, continuous |  |
| 30392 | | 9 |  | Other and unspecified alcohol dependence, episodic |  |
| 30393 | | 9 |  | Other and unspecified alcohol dependence, in remission |  |
| 30400 | | 9 |  | Opioid type dependence, unspecified |  |
| 30401 | | 9 |  | Opioid type dependence, continuous |  |
| 30300 | | 9 |  | Acute alcoholic intoxication in alcoholism, unspecified |  |
| 30301 | | 9 |  | Acute alcoholic intoxication in alcoholism, continuous |  |
| 30302 | | 9 |  | Acute alcoholic intoxication in alcoholism, episodic |  |
| 30402 | | 9 |  | Opioid type dependence, episodic |  |
| 30403 | | 9 |  | Opioid type dependence, in remission |  |
| 30410 | | 9 |  | Sedative, hypnotic or anxiolytic dependence, unspecified |  |
| 30411 | | 9 |  | Sedative, hypnotic or anxiolytic dependence, continuous |  |
| 30412 | | 9 |  | Sedative, hypnotic or anxiolytic dependence, episodic |  |
| 30413 | | 9 |  | Sedative, hypnotic or anxiolytic dependence, in remission |  |
| 30420 | | 9 |  | Cocaine dependence, unspecified |  |
| 30421 | | 9 |  | Cocaine dependence, continuous |  |
| 30422 | | 9 |  | Cocaine dependence, episodic |  |
| 30423 | | 9 |  | Cocaine dependence, in remission |  |
| 30430 | | 9 |  | Cannabis dependence, unspecified |  |
| 30431 | | 9 |  | Cannabis dependence, continuous |  |
| 30432 | | 9 |  | Cannabis dependence, episodic |  |
| 30433 | | 9 |  | Cannabis dependence, in remission |  |
| 30440 | | 9 |  | Amphetamine and other psychostimulant dependence, unspecified |  |
| 30441 | | 9 |  | Amphetamine and other psychostimulant dependence, continuous |  |
| 30442 | | 9 |  | Amphetamine and other psychostimulant dependence, episodic |  |
| 30443 | | 9 |  | Amphetamine and other psychostimulant dependence, in remission |  |
| 30450 | | 9 |  | Hallucinogen dependence, unspecified |  |
| 30451 | | 9 |  | Hallucinogen dependence, continuous |  |
| 30452 | | 9 |  | Hallucinogen dependence, episodic |  |
| 30453 | | 9 |  | Hallucinogen dependence, in remission |  |
| 30460 | | 9 |  | Other specified drug dependence, unspecified |  |
| Z7141 | | 10 |  | Alcohol abuse counseling and surveillance of alcoholic |  |
| Z811 | | 10 |  | Family history of alcohol abuse and dependence |  |
| 2910 | | 9 |  | Alcohol withdrawal delirium |  |
| 2911 | | 9 |  | Alcohol-induced persisting amnestic disorder |  |
| 2912 | | 9 |  | Alcohol-induced persisting dementia |  |
| 2913 | | 9 |  | Alcohol-induced psychotic disorder with hallucinations |  |
| 2914 | | 9 |  | Idiosyncratic alcohol intoxication |  |
| 2915 | | 9 |  | Alcohol-induced psychotic disorder with delusions |  |
| 29181 | | 9 |  | Alcohol withdrawal |  |
| 29182 | | 9 |  | Alcohol induced sleep disorders |  |
| 29189 | | 9 |  | Other alcohol-induced mental disorders |  |
| 2919 | | 9 |  | Unspecified alcohol-induced mental disorders |  |
| 3051 | | 9 |  | Tobacco use disorder |  |
| 30520 | | 9 |  | Cannabis abuse, unspecified |  |
| 30521 | | 9 |  | Cannabis abuse, continuous |  |
| 30522 | | 9 |  | Cannabis abuse, episodic |  |
| 30523 | | 9 |  | Cannabis abuse, in remission |  |
| 30530 | | 9 |  | Hallucinogen abuse, unspecified |  |
| 30531 | | 9 |  | Hallucinogen abuse, continuous |  |
| 30532 | | 9 |  | Hallucinogen abuse, episodic |  |
| 30533 | | 9 |  | Hallucinogen abuse, in remission |  |
| 30540 | | 9 |  | Sedative, hypnotic or anxiolytic abuse, unspecified |  |
| 30541 | | 9 |  | Sedative, hypnotic or anxiolytic abuse, continuous |  |
| 30542 | | 9 |  | Sedative, hypnotic or anxiolytic abuse, episodic |  |
| 30543 | | 9 |  | Sedative, hypnotic or anxiolytic abuse, in remission |  |
| 30550 | | 9 |  | Opioid abuse, unspecified |  |
| 30551 | | 9 |  | Opioid abuse, continuous |  |
| 30552 | | 9 |  | Opioid abuse, episodic |  |
| 30553 | | 9 |  | Opioid abuse, in remission |  |
| 30560 | | 9 |  | Cocaine abuse, unspecified |  |
| 30561 | | 9 |  | Cocaine abuse, continuous |  |
| 30562 | | 9 |  | Cocaine abuse, episodic |  |
| 30563 | | 9 |  | Cocaine abuse, in remission |  |
| 30570 | | 9 |  | Amphetamine or related acting sympathomimetic abuse, unspecified |  |
| 30571 | | 9 |  | Amphetamine or related acting sympathomimetic abuse, continuous |  |
| 30572 | | 9 |  | Amphetamine or related acting sympathomimetic abuse, episodic |  |
| 30573 | | 9 |  | Amphetamine or related acting sympathomimetic abuse, in remission |  |
| 30580 | | 9 |  | Antidepressant type abuse, unspecified |  |
| 30581 | | 9 |  | Antidepressant type abuse, continuous |  |
| 30582 | | 9 |  | Antidepressant type abuse, episodic |  |
| 30583 | | 9 |  | Antidepressant type abuse, in remission |  |
| 64900 | | 9 |  | Tobacco use disorder complicating pregnancy, childbirth, or the puerperium, unspecified as to episode of care or not applicable |  |
| 64901 | | 9 |  | Tobacco use disorder complicating pregnancy, childbirth, or the puerperium, delivered, with or without mention of antepartum condition |  |
| 64902 | | 9 |  | Tobacco use disorder complicating pregnancy, childbirth, or the puerperium, delivered, with mention of postpartum complication |  |
| 64903 | | 9 |  | Tobacco use disorder complicating pregnancy, childbirth, or the puerperium, antepartum condition or complication |  |
| 64904 | | 9 |  | Tobacco use disorder complicating pregnancy, childbirth, or the puerperium, postpartum condition or complication |  |
| F1010 | | 10 |  | Alcohol abuse, uncomplicated |  |
| F1011 | | 10 |  | Alcohol abuse, in remission |  |
| F10120 | | 10 |  | Alcohol abuse with intoxication, uncomplicated |  |
| F10121 | | 10 |  | Alcohol abuse with intoxication delirium |  |
| F10129 | | 10 |  | Alcohol abuse with intoxication, unspecified |  |
| F1014 | | 10 |  | Alcohol abuse with alcohol-induced mood disorder |  |
| F10150 | | 10 |  | Alcohol abuse with alcohol-induced psychotic disorder with delusions |  |
| F10151 | | 10 |  | Alcohol abuse with alcohol-induced psychotic disorder with hallucinations |  |
| F10159 | | 10 |  | Alcohol abuse with alcohol-induced psychotic disorder, unspecified |  |
| F10180 | | 10 |  | Alcohol abuse with alcohol-induced anxiety disorder |  |
| F10181 | | 10 |  | Alcohol abuse with alcohol-induced sexual dysfunction |  |
| F10182 | | 10 |  | Alcohol abuse with alcohol-induced sleep disorder |  |
| F10188 | | 10 |  | Alcohol abuse with other alcohol-induced disorder |  |
| F1019 | | 10 |  | Alcohol abuse with unspecified alcohol-induced disorder |  |
| F1020 | | 10 |  | Alcohol dependence, uncomplicated |  |
| F1021 | | 10 |  | Alcohol dependence, in remission |  |
| F10220 | | 10 |  | Alcohol dependence with intoxication, uncomplicated |  |
| F10221 | | 10 |  | Alcohol dependence with intoxication delirium |  |
| F10229 | | 10 |  | Alcohol dependence with intoxication, unspecified |  |
| F10230 | | 10 |  | Alcohol dependence with withdrawal, uncomplicated |  |
| F10231 | | 10 |  | Alcohol dependence with withdrawal delirium |  |
| F10232 | | 10 |  | Alcohol dependence with withdrawal with perceptual disturbance |  |
| F10239 | | 10 |  | Alcohol dependence with withdrawal, unspecified |  |
| F1024 | | 10 |  | Alcohol dependence with alcohol-induced mood disorder |  |
| F10250 | | 10 |  | Alcohol dependence with alcohol-induced psychotic disorder with delusions |  |
| F10251 | | 10 |  | Alcohol dependence with alcohol-induced psychotic disorder with hallucinations |  |
| F10259 | | 10 |  | Alcohol dependence with alcohol-induced psychotic disorder, unspecified |  |
| F1026 | | 10 |  | Alcohol dependence with alcohol-induced persisting amnestic disorder |  |
| F1027 | | 10 |  | Alcohol dependence with alcohol-induced persisting dementia |  |
| F10280 | | 10 |  | Alcohol dependence with alcohol-induced anxiety disorder |  |
| F10281 | | 10 |  | Alcohol dependence with alcohol-induced sexual dysfunction |  |
| F10282 | | 10 |  | Alcohol dependence with alcohol-induced sleep disorder |  |
| F10288 | | 10 |  | Alcohol dependence with other alcohol-induced disorder |  |
| F1029 | | 10 |  | Alcohol dependence with unspecified alcohol-induced disorder |  |
| F10920 | | 10 |  | Alcohol use, unspecified with intoxication, uncomplicated |  |
| F10921 | | 10 |  | Alcohol use, unspecified with intoxication delirium |  |
| F10929 | | 10 |  | Alcohol use, unspecified with intoxication, unspecified |  |
| F1094 | | 10 |  | Alcohol use, unspecified with alcohol-induced mood disorder |  |
| F10950 | | 10 |  | Alcohol use, unspecified with alcohol-induced psychotic disorder with delusions |  |
| F10951 | | 10 |  | Alcohol use, unspecified with alcohol-induced psychotic disorder with hallucinations |  |
| F10959 | | 10 |  | Alcohol use, unspecified with alcohol-induced psychotic disorder, unspecified |  |
| F1096 | | 10 |  | Alcohol use, unspecified with alcohol-induced persisting amnestic disorder |  |
| F1097 | | 10 |  | Alcohol use, unspecified with alcohol-induced persisting dementia |  |
| F10980 | | 10 |  | Alcohol use, unspecified with alcohol-induced anxiety disorder |  |
| F10981 | | 10 |  | Alcohol use, unspecified with alcohol-induced sexual dysfunction |  |
| F10982 | | 10 |  | Alcohol use, unspecified with alcohol-induced sleep disorder |  |
| F10988 | | 10 |  | Alcohol use, unspecified with other alcohol-induced disorder |  |
| F1099 | | 10 |  | Alcohol use, unspecified with unspecified alcohol-induced disorder |  |
| F1110 | | 10 |  | Opioid abuse, uncomplicated |  |
| F1111 | | 10 |  | Opioid abuse, in remission |  |
| F11120 | | 10 |  | Opioid abuse with intoxication, uncomplicated |  |
| F11121 | | 10 |  | Opioid abuse with intoxication delirium |  |
| F11122 | | 10 |  | Opioid abuse with intoxication with perceptual disturbance |  |
| F11129 | | 10 |  | Opioid abuse with intoxication, unspecified |  |
| F1114 | | 10 |  | Opioid abuse with opioid-induced mood disorder |  |
| F11150 | | 10 |  | Opioid abuse with opioid-induced psychotic disorder with delusions |  |
| F11151 | | 10 |  | Opioid abuse with opioid-induced psychotic disorder with hallucinations |  |
| F11159 | | 10 |  | Opioid abuse with opioid-induced psychotic disorder, unspecified |  |
| F11181 | | 10 |  | Opioid abuse with opioid-induced sexual dysfunction |  |
| F11182 | | 10 |  | Opioid abuse with opioid-induced sleep disorder |  |
| F11188 | | 10 |  | Opioid abuse with other opioid-induced disorder |  |
| F1119 | | 10 |  | Opioid abuse with unspecified opioid-induced disorder |  |
| F1120 | | 10 |  | Opioid dependence, uncomplicated |  |
| F1121 | | 10 |  | Opioid dependence, in remission |  |
| F11220 | | 10 |  | Opioid dependence with intoxication, uncomplicated |  |
| F11221 | | 10 |  | Opioid dependence with intoxication delirium |  |
| F11222 | | 10 |  | Opioid dependence with intoxication with perceptual disturbance |  |
| F11229 | | 10 |  | Opioid dependence with intoxication, unspecified |  |
| F1123 | | 10 |  | Opioid dependence with withdrawal |  |
| F1124 | | 10 |  | Opioid dependence with opioid-induced mood disorder |  |
| F11250 | | 10 |  | Opioid dependence with opioid-induced psychotic disorder with delusions |  |
| F11251 | | 10 |  | Opioid dependence with opioid-induced psychotic disorder with hallucinations |  |
| F11259 | | 10 |  | Opioid dependence with opioid-induced psychotic disorder, unspecified |  |
| F11281 | | 10 |  | Opioid dependence with opioid-induced sexual dysfunction |  |
| F11282 | | 10 |  | Opioid dependence with opioid-induced sleep disorder |  |
| F11288 | | 10 |  | Opioid dependence with other opioid-induced disorder |  |
| F1129 | | 10 |  | Opioid dependence with unspecified opioid-induced disorder |  |
| F1190 | | 10 |  | Opioid use, unspecified, uncomplicated |  |
| F11920 | | 10 |  | Opioid use, unspecified with intoxication, uncomplicated |  |
| F11921 | | 10 |  | Opioid use, unspecified with intoxication delirium |  |
| F11922 | | 10 |  | Opioid use, unspecified with intoxication with perceptual disturbance |  |
| F11929 | | 10 |  | Opioid use, unspecified with intoxication, unspecified |  |
| F1193 | | 10 |  | Opioid use, unspecified with withdrawal |  |
| F1194 | | 10 |  | Opioid use, unspecified with opioid-induced mood disorder |  |
| F11950 | | 10 |  | Opioid use, unspecified with opioid-induced psychotic disorder with delusions |  |
| F11951 | | 10 |  | Opioid use, unspecified with opioid-induced psychotic disorder with hallucinations |  |
| F11959 | | 10 |  | Opioid use, unspecified with opioid-induced psychotic disorder, unspecified |  |
| F11981 | | 10 |  | Opioid use, unspecified with opioid-induced sexual dysfunction |  |
| F11982 | | 10 |  | Opioid use, unspecified with opioid-induced sleep disorder |  |
| F11988 | | 10 |  | Opioid use, unspecified with other opioid-induced disorder |  |
| F1199 | | 10 |  | Opioid use, unspecified with unspecified opioid-induced disorder |  |
| F1210 | | 10 |  | Cannabis abuse, uncomplicated |  |
| F1211 | | 10 |  | Cannabis abuse, in remission |  |
| F12120 | | 10 |  | Cannabis abuse with intoxication, uncomplicated |  |
| F12121 | | 10 |  | Cannabis abuse with intoxication delirium |  |
| F12122 | | 10 |  | Cannabis abuse with intoxication with perceptual disturbance |  |
| F12129 | | 10 |  | Cannabis abuse with intoxication, unspecified |  |
| F12150 | | 10 |  | Cannabis abuse with psychotic disorder with delusions |  |
| F12151 | | 10 |  | Cannabis abuse with psychotic disorder with hallucinations |  |
| F12159 | | 10 |  | Cannabis abuse with psychotic disorder, unspecified |  |
| F12180 | | 10 |  | Cannabis abuse with cannabis-induced anxiety disorder |  |
| F12188 | | 10 |  | Cannabis abuse with other cannabis-induced disorder |  |
| F1219 | | 10 |  | Cannabis abuse with unspecified cannabis-induced disorder |  |
| F1220 | | 10 |  | Cannabis dependence, uncomplicated |  |
| F1221 | | 10 |  | Cannabis dependence, in remission |  |
| F12220 | | 10 |  | Cannabis dependence with intoxication, uncomplicated |  |
| F12221 | | 10 |  | Cannabis dependence with intoxication delirium |  |
| F12222 | | 10 |  | Cannabis dependence with intoxication with perceptual disturbance |  |
| F12229 | | 10 |  | Cannabis dependence with intoxication, unspecified |  |
| F1223 | | 10 |  | Cannabis dependence with withdrawal |  |
| F12250 | | 10 |  | Cannabis dependence with psychotic disorder with delusions |  |
| F12251 | | 10 |  | Cannabis dependence with psychotic disorder with hallucinations |  |
| F12259 | | 10 |  | Cannabis dependence with psychotic disorder, unspecified |  |
| F12280 | | 10 |  | Cannabis dependence with cannabis-induced anxiety disorder |  |
| F12288 | | 10 |  | Cannabis dependence with other cannabis-induced disorder |  |
| F1229 | | 10 |  | Cannabis dependence with unspecified cannabis-induced disorder |  |
| F1290 | | 10 |  | Cannabis use, unspecified, uncomplicated |  |
| F12920 | | 10 |  | Cannabis use, unspecified with intoxication, uncomplicated |  |
| F12921 | | 10 |  | Cannabis use, unspecified with intoxication delirium |  |
| F12922 | | 10 |  | Cannabis use, unspecified with intoxication with perceptual disturbance |  |
| F12929 | | 10 |  | Cannabis use, unspecified with intoxication, unspecified |  |
| F1293 | | 10 |  | Cannabis use, unspecified with withdrawal |  |
| F12950 | | 10 |  | Cannabis use, unspecified with psychotic disorder with delusions |  |
| F12951 | | 10 |  | Cannabis use, unspecified with psychotic disorder with hallucinations |  |
| F12959 | | 10 |  | Cannabis use, unspecified with psychotic disorder, unspecified |  |
| F12980 | | 10 |  | Cannabis use, unspecified with anxiety disorder |  |
| F12988 | | 10 |  | Cannabis use, unspecified with other cannabis-induced disorder |  |
| F1299 | | 10 |  | Cannabis use, unspecified with unspecified cannabis-induced disorder |  |
| F1310 | | 10 |  | Sedative, hypnotic or anxiolytic abuse, uncomplicated |  |
| F1311 | | 10 |  | Sedative, hypnotic or anxiolytic abuse, in remission |  |
| F13120 | | 10 |  | Sedative, hypnotic or anxiolytic abuse with intoxication, uncomplicated |  |
| F13121 | | 10 |  | Sedative, hypnotic or anxiolytic abuse with intoxication delirium |  |
| F13129 | | 10 |  | Sedative, hypnotic or anxiolytic abuse with intoxication, unspecified |  |
| F1314 | | 10 |  | Sedative, hypnotic or anxiolytic abuse with sedative, hypnotic or anxiolytic-induced mood disorder |  |
| F13150 | | 10 |  | Sedative, hypnotic or anxiolytic abuse with sedative, hypnotic or anxiolytic-induced psychotic disorder with delusions |  |
| F13151 | | 10 |  | Sedative, hypnotic or anxiolytic abuse with sedative, hypnotic or anxiolytic-induced psychotic disorder with hallucinations |  |
| F13159 | | 10 |  | Sedative, hypnotic or anxiolytic abuse with sedative, hypnotic or anxiolytic-induced psychotic disorder, unspecified |  |
| F13180 | | 10 |  | Sedative, hypnotic or anxiolytic abuse with sedative, hypnotic or anxiolytic-induced anxiety disorder |  |
| F13181 | | 10 |  | Sedative, hypnotic or anxiolytic abuse with sedative, hypnotic or anxiolytic-induced sexual dysfunction |  |
| F13182 | | 10 |  | Sedative, hypnotic or anxiolytic abuse with sedative, hypnotic or anxiolytic-induced sleep disorder |  |
| F13188 | | 10 |  | Sedative, hypnotic or anxiolytic abuse with other sedative, hypnotic or anxiolytic-induced disorder |  |
| F1319 | | 10 |  | Sedative, hypnotic or anxiolytic abuse with unspecified sedative, hypnotic or anxiolytic-induced disorder |  |
| F1320 | | 10 |  | Sedative, hypnotic or anxiolytic dependence, uncomplicated |  |
| F1321 | | 10 |  | Sedative, hypnotic or anxiolytic dependence, in remission |  |
| F13220 | | 10 |  | Sedative, hypnotic or anxiolytic dependence with intoxication, uncomplicated |  |
| F13221 | | 10 |  | Sedative, hypnotic or anxiolytic dependence with intoxication delirium |  |
| F13229 | | 10 |  | Sedative, hypnotic or anxiolytic dependence with intoxication, unspecified |  |
| F13230 | | 10 |  | Sedative, hypnotic or anxiolytic dependence with withdrawal, uncomplicated |  |
| F13231 | | 10 |  | Sedative, hypnotic or anxiolytic dependence with withdrawal delirium |  |
| F13232 | | 10 |  | Sedative, hypnotic or anxiolytic dependence with withdrawal with perceptual disturbance |  |
| F13239 | | 10 |  | Sedative, hypnotic or anxiolytic dependence with withdrawal, unspecified |  |
| F1324 | | 10 |  | Sedative, hypnotic or anxiolytic dependence with sedative, hypnotic or anxiolytic-induced mood disorder |  |
| F13250 | | 10 |  | Sedative, hypnotic or anxiolytic dependence with sedative, hypnotic or anxiolytic-induced psychotic disorder with delusions |  |
| F13251 | | 10 |  | Sedative, hypnotic or anxiolytic dependence with sedative, hypnotic or anxiolytic-induced psychotic disorder with hallucinations |  |
| F13259 | | 10 |  | Sedative, hypnotic or anxiolytic dependence with sedative, hypnotic or anxiolytic-induced psychotic disorder, unspecified |  |
| F1326 | | 10 |  | Sedative, hypnotic or anxiolytic dependence with sedative, hypnotic or anxiolytic-induced persisting amnestic disorder |  |
| F1327 | | 10 |  | Sedative, hypnotic or anxiolytic dependence with sedative, hypnotic or anxiolytic-induced persisting dementia |  |
| F13280 | | 10 |  | Sedative, hypnotic or anxiolytic dependence with sedative, hypnotic or anxiolytic-induced anxiety disorder |  |
| F13281 | | 10 |  | Sedative, hypnotic or anxiolytic dependence with sedative, hypnotic or anxiolytic-induced sexual dysfunction |  |
| F13282 | | 10 |  | Sedative, hypnotic or anxiolytic dependence with sedative, hypnotic or anxiolytic-induced sleep disorder |  |
| F13288 | | 10 |  | Sedative, hypnotic or anxiolytic dependence with other sedative, hypnotic or anxiolytic-induced disorder |  |
| F1329 | | 10 |  | Sedative, hypnotic or anxiolytic dependence with unspecified sedative, hypnotic or anxiolytic-induced disorder |  |
| F1390 | | 10 |  | Sedative, hypnotic, or anxiolytic use, unspecified, uncomplicated |  |
| F13920 | | 10 |  | Sedative, hypnotic or anxiolytic use, unspecified with intoxication, uncomplicated |  |
| F13921 | | 10 |  | Sedative, hypnotic or anxiolytic use, unspecified with intoxication delirium |  |
| F13929 | | 10 |  | Sedative, hypnotic or anxiolytic use, unspecified with intoxication, unspecified |  |
| F13930 | | 10 |  | Sedative, hypnotic or anxiolytic use, unspecified with withdrawal, uncomplicated |  |
| F13931 | | 10 |  | Sedative, hypnotic or anxiolytic use, unspecified with withdrawal delirium |  |
| F13932 | | 10 |  | Sedative, hypnotic or anxiolytic use, unspecified with withdrawal with perceptual disturbances |  |
| F15221 | | 10 |  | Other stimulant dependence with intoxication delirium |  |
| F13939 | | 10 |  | Sedative, hypnotic or anxiolytic use, unspecified with withdrawal, unspecified |  |
| F1394 | | 10 |  | Sedative, hypnotic or anxiolytic use, unspecified with sedative, hypnotic or anxiolytic-induced mood disorder |  |
| F13950 | | 10 |  | Sedative, hypnotic or anxiolytic use, unspecified with sedative, hypnotic or anxiolytic-induced psychotic disorder with delusions |  |
| F13951 | | 10 |  | Sedative, hypnotic or anxiolytic use, unspecified with sedative, hypnotic or anxiolytic-induced psychotic disorder with hallucinations |  |
| F13959 | | 10 |  | Sedative, hypnotic or anxiolytic use, unspecified with sedative, hypnotic or anxiolytic-induced psychotic disorder, unspecified |  |
| F1396 | | 10 |  | Sedative, hypnotic or anxiolytic use, unspecified with sedative, hypnotic or anxiolytic-induced persisting amnestic disorder |  |
| F1397 | | 10 |  | Sedative, hypnotic or anxiolytic use, unspecified with sedative, hypnotic or anxiolytic-induced persisting dementia |  |
| F13980 | | 10 |  | Sedative, hypnotic or anxiolytic use, unspecified with sedative, hypnotic or anxiolytic-induced anxiety disorder |  |
| F13981 | | 10 |  | Sedative, hypnotic or anxiolytic use, unspecified with sedative, hypnotic or anxiolytic-induced sexual dysfunction |  |
| F13982 | | 10 |  | Sedative, hypnotic or anxiolytic use, unspecified with sedative, hypnotic or anxiolytic-induced sleep disorder |  |
| F13988 | | 10 |  | Sedative, hypnotic or anxiolytic use, unspecified with other sedative, hypnotic or anxiolytic-induced disorder |  |
| F1399 | | 10 |  | Sedative, hypnotic or anxiolytic use, unspecified with unspecified sedative, hypnotic or anxiolytic-induced disorder |  |
| F1410 | | 10 |  | Cocaine abuse, uncomplicated |  |
| F1411 | | 10 |  | Cocaine abuse, in remission |  |
| F14120 | | 10 |  | Cocaine abuse with intoxication, uncomplicated |  |
| F14121 | | 10 |  | Cocaine abuse with intoxication with delirium |  |
| F14122 | | 10 |  | Cocaine abuse with intoxication with perceptual disturbance |  |
| F14129 | | 10 |  | Cocaine abuse with intoxication, unspecified |  |
| F1414 | | 10 |  | Cocaine abuse with cocaine-induced mood disorder |  |
| F14150 | | 10 |  | Cocaine abuse with cocaine-induced psychotic disorder with delusions |  |
| F14151 | | 10 |  | Cocaine abuse with cocaine-induced psychotic disorder with hallucinations |  |
| F14159 | | 10 |  | Cocaine abuse with cocaine-induced psychotic disorder, unspecified |  |
| F14180 | | 10 |  | Cocaine abuse with cocaine-induced anxiety disorder |  |
| F14181 | | 10 |  | Cocaine abuse with cocaine-induced sexual dysfunction |  |
| F14182 | | 10 |  | Cocaine abuse with cocaine-induced sleep disorder |  |
| F14188 | | 10 |  | Cocaine abuse with other cocaine-induced disorder |  |
| F1419 | | 10 |  | Cocaine abuse with unspecified cocaine-induced disorder |  |
| F1420 | | 10 |  | Cocaine dependence, uncomplicated |  |
| F1421 | | 10 |  | Cocaine dependence, in remission |  |
| F14220 | | 10 |  | Cocaine dependence with intoxication, uncomplicated |  |
| F14221 | | 10 |  | Cocaine dependence with intoxication delirium |  |
| F14222 | | 10 |  | Cocaine dependence with intoxication with perceptual disturbance |  |
| F14229 | | 10 |  | Cocaine dependence with intoxication, unspecified |  |
| F1423 | | 10 |  | Cocaine dependence with withdrawal |  |
| F1424 | | 10 |  | Cocaine dependence with cocaine-induced mood disorder |  |
| F14250 | | 10 |  | Cocaine dependence with cocaine-induced psychotic disorder with delusions |  |
| F14251 | | 10 |  | Cocaine dependence with cocaine-induced psychotic disorder with hallucinations |  |
| F14259 | | 10 |  | Cocaine dependence with cocaine-induced psychotic disorder, unspecified |  |
| F14280 | | 10 |  | Cocaine dependence with cocaine-induced anxiety disorder |  |
| F14281 | | 10 |  | Cocaine dependence with cocaine-induced sexual dysfunction |  |
| F14282 | | 10 |  | Cocaine dependence with cocaine-induced sleep disorder |  |
| F14288 | | 10 |  | Cocaine dependence with other cocaine-induced disorder |  |
| F1429 | | 10 |  | Cocaine dependence with unspecified cocaine-induced disorder |  |
| F1490 | | 10 |  | Cocaine use, unspecified, uncomplicated |  |
| F14920 | | 10 |  | Cocaine use, unspecified with intoxication, uncomplicated |  |
| F14921 | | 10 |  | Cocaine use, unspecified with intoxication delirium |  |
| F14922 | | 10 |  | Cocaine use, unspecified with intoxication with perceptual disturbance |  |
| F14929 | | 10 |  | Cocaine use, unspecified with intoxication, unspecified |  |
| F1494 | | 10 |  | Cocaine use, unspecified with cocaine-induced mood disorder |  |
| F14950 | | 10 |  | Cocaine use, unspecified with cocaine-induced psychotic disorder with delusions |  |
| F14951 | | 10 |  | Cocaine use, unspecified with cocaine-induced psychotic disorder with hallucinations |  |
| F14959 | | 10 |  | Cocaine use, unspecified with cocaine-induced psychotic disorder, unspecified |  |
| F14980 | | 10 |  | Cocaine use, unspecified with cocaine-induced anxiety disorder |  |
| F14981 | | 10 |  | Cocaine use, unspecified with cocaine-induced sexual dysfunction |  |
| F14982 | | 10 |  | Cocaine use, unspecified with cocaine-induced sleep disorder |  |
| F14988 | | 10 |  | Cocaine use, unspecified with other cocaine-induced disorder |  |
| F1499 | | 10 |  | Cocaine use, unspecified with unspecified cocaine-induced disorder |  |
| F1510 | | 10 |  | Other stimulant abuse, uncomplicated |  |
| F1511 | | 10 |  | Other stimulant abuse, in remission |  |
| F15120 | | 10 |  | Other stimulant abuse with intoxication, uncomplicated |  |
| F15121 | | 10 |  | Other stimulant abuse with intoxication delirium |  |
| F15122 | | 10 |  | Other stimulant abuse with intoxication with perceptual disturbance |  |
| F15129 | | 10 |  | Other stimulant abuse with intoxication, unspecified |  |
| F1514 | | 10 |  | Other stimulant abuse with stimulant-induced mood disorder |  |
| F15150 | | 10 |  | Other stimulant abuse with stimulant-induced psychotic disorder with delusions |  |
| F15151 | | 10 |  | Other stimulant abuse with stimulant-induced psychotic disorder with hallucinations |  |
| F15159 | | 10 |  | Other stimulant abuse with stimulant-induced psychotic disorder, unspecified |  |
| F15180 | | 10 |  | Other stimulant abuse with stimulant-induced anxiety disorder |  |
| F15181 | | 10 |  | Other stimulant abuse with stimulant-induced sexual dysfunction |  |
| F15182 | | 10 |  | Other stimulant abuse with stimulant-induced sleep disorder |  |
| F15188 | | 10 |  | Other stimulant abuse with other stimulant-induced disorder |  |
| F1519 | | 10 |  | Other stimulant abuse with unspecified stimulant-induced disorder |  |
| F1520 | | 10 |  | Other stimulant dependence, uncomplicated |  |
| F1521 | | 10 |  | Other stimulant dependence, in remission |  |
| F15220 | | 10 |  | Other stimulant dependence with intoxication, uncomplicated |  |
| F15222 | | 10 |  | Other stimulant dependence with intoxication with perceptual disturbance |  |
| F15229 | | 10 |  | Other stimulant dependence with intoxication, unspecified |  |
| F1523 | | 10 |  | Other stimulant dependence with withdrawal |  |
| F1524 | | 10 |  | Other stimulant dependence with stimulant-induced mood disorder |  |
| F15250 | | 10 |  | Other stimulant dependence with stimulant-induced psychotic disorder with delusions |  |
| F15251 | | 10 |  | Other stimulant dependence with stimulant-induced psychotic disorder with hallucinations |  |
| F15259 | | 10 |  | Other stimulant dependence with stimulant-induced psychotic disorder, unspecified |  |
| F15280 | | 10 |  | Other stimulant dependence with stimulant-induced anxiety disorder |  |
| F15281 | | 10 |  | Other stimulant dependence with stimulant-induced sexual dysfunction |  |
| F15282 | | 10 |  | Other stimulant dependence with stimulant-induced sleep disorder |  |
| F15288 | | 10 |  | Other stimulant dependence with other stimulant-induced disorder |  |
| F1529 | | 10 |  | Other stimulant dependence with unspecified stimulant-induced disorder |  |
| F1590 | | 10 |  | Other stimulant use, unspecified, uncomplicated |  |
| F15920 | | 10 |  | Other stimulant use, unspecified with intoxication, uncomplicated |  |
| F15921 | | 10 |  | Other stimulant use, unspecified with intoxication delirium |  |
| F15922 | | 10 |  | Other stimulant use, unspecified with intoxication with perceptual disturbance |  |
| F15929 | | 10 |  | Other stimulant use, unspecified with intoxication, unspecified |  |
| F1593 | | 10 |  | Other stimulant use, unspecified with withdrawal |  |
| F1594 | | 10 |  | Other stimulant use, unspecified with stimulant-induced mood disorder |  |
| F15950 | | 10 |  | Other stimulant use, unspecified with stimulant-induced psychotic disorder with delusions |  |
| F15951 | | 10 |  | Other stimulant use, unspecified with stimulant-induced psychotic disorder with hallucinations |  |
| F15959 | | 10 |  | Other stimulant use, unspecified with stimulant-induced psychotic disorder, unspecified |  |
| F15980 | | 10 |  | Other stimulant use, unspecified with stimulant-induced anxiety disorder |  |
| F15981 | | 10 |  | Other stimulant use, unspecified with stimulant-induced sexual dysfunction |  |
| F15982 | | 10 |  | Other stimulant use, unspecified with stimulant-induced sleep disorder |  |
| F15988 | | 10 |  | Other stimulant use, unspecified with other stimulant-induced disorder |  |
| F1599 | | 10 |  | Other stimulant use, unspecified with unspecified stimulant-induced disorder |  |
| F1511 | | 10 |  | Other stimulant abuse, in remission |  |
| F15120 | | 10 |  | Other stimulant abuse with intoxication, uncomplicated |  |
| F15121 | | 10 |  | Other stimulant abuse with intoxication delirium |  |
| F15122 | | 10 |  | Other stimulant abuse with intoxication with perceptual disturbance |  |
| F15129 | | 10 |  | Other stimulant abuse with intoxication, unspecified |  |
| F1514 | | 10 |  | Other stimulant abuse with stimulant-induced mood disorder |  |
| F15150 | | 10 |  | Other stimulant abuse with stimulant-induced psychotic disorder with delusions |  |
| F15151 | | 10 |  | Other stimulant abuse with stimulant-induced psychotic disorder with hallucinations |  |
| F15159 | | 10 |  | Other stimulant abuse with stimulant-induced psychotic disorder, unspecified |  |
| F15180 | | 10 |  | Other stimulant abuse with stimulant-induced anxiety disorder |  |
| F15181 | | 10 |  | Other stimulant abuse with stimulant-induced sexual dysfunction |  |
| F15182 | | 10 |  | Other stimulant abuse with stimulant-induced sleep disorder |  |
| F15188 | | 10 |  | Other stimulant abuse with other stimulant-induced disorder |  |
| F1519 | | 10 |  | Other stimulant abuse with unspecified stimulant-induced disorder |  |
| F1520 | | 10 |  | Other stimulant dependence, uncomplicated |  |
| F1521 | | 10 |  | Other stimulant dependence, in remission |  |
| F15220 | | 10 |  | Other stimulant dependence with intoxication, uncomplicated |  |
| F15222 | | 10 |  | Other stimulant dependence with intoxication with perceptual disturbance |  |
| F15229 | | 10 |  | Other stimulant dependence with intoxication, unspecified |  |
| F1523 | | 10 |  | Other stimulant dependence with withdrawal |  |
| F1524 | | 10 |  | Other stimulant dependence with stimulant-induced mood disorder |  |
| F15250 | | 10 |  | Other stimulant dependence with stimulant-induced psychotic disorder with delusions |  |
| F15251 | | 10 |  | Other stimulant dependence with stimulant-induced psychotic disorder with hallucinations |  |
| F15259 | | 10 |  | Other stimulant dependence with stimulant-induced psychotic disorder, unspecified |  |
| F15280 | | 10 |  | Other stimulant dependence with stimulant-induced anxiety disorder |  |
| F15281 | | 10 |  | Other stimulant dependence with stimulant-induced sexual dysfunction |  |
| F15282 | | 10 |  | Other stimulant dependence with stimulant-induced sleep disorder |  |
| F15288 | | 10 |  | Other stimulant dependence with other stimulant-induced disorder |  |
| F1529 | | 10 |  | Other stimulant dependence with unspecified stimulant-induced disorder |  |
| F1590 | | 10 |  | Other stimulant use, unspecified, uncomplicated |  |
| F15920 | | 10 |  | Other stimulant use, unspecified with intoxication, uncomplicated |  |
| F15921 | | 10 |  | Other stimulant use, unspecified with intoxication delirium |  |
| F15922 | | 10 |  | Other stimulant use, unspecified with intoxication with perceptual disturbance |  |
| F15929 | | 10 |  | Other stimulant use, unspecified with intoxication, unspecified |  |
| F1593 | | 10 |  | Other stimulant use, unspecified with withdrawal |  |
| F1594 | | 10 |  | Other stimulant use, unspecified with stimulant-induced mood disorder |  |
| F15950 | | 10 |  | Other stimulant use, unspecified with stimulant-induced psychotic disorder with delusions |  |
| F15951 | | 10 |  | Other stimulant use, unspecified with stimulant-induced psychotic disorder with hallucinations |  |
| F15959 | | 10 |  | Other stimulant use, unspecified with stimulant-induced psychotic disorder, unspecified |  |
| F15980 | | 10 |  | Other stimulant use, unspecified with stimulant-induced anxiety disorder |  |
| F15981 | | 10 |  | Other stimulant use, unspecified with stimulant-induced sexual dysfunction |  |
| F15982 | | 10 |  | Other stimulant use, unspecified with stimulant-induced sleep disorder |  |
| F15988 | | 10 |  | Other stimulant use, unspecified with other stimulant-induced disorder |  |
| F1599 | | 10 |  | Other stimulant use, unspecified with unspecified stimulant-induced disorder |  |
| F1610 | | 10 |  | Hallucinogen abuse, uncomplicated |  |
| F1611 | | 10 |  | Hallucinogen abuse, in remission |  |
| F16120 | | 10 |  | Hallucinogen abuse with intoxication, uncomplicated |  |
| F16121 | | 10 |  | Hallucinogen abuse with intoxication with delirium |  |
| F16122 | | 10 |  | Hallucinogen abuse with intoxication with perceptual disturbance |  |
| F16129 | | 10 |  | Hallucinogen abuse with intoxication, unspecified |  |
| F1614 | | 10 |  | Hallucinogen abuse with hallucinogen-induced mood disorder |  |
| F16150 | | 10 |  | Hallucinogen abuse with hallucinogen-induced psychotic disorder with delusions |  |
| F16151 | | 10 |  | Hallucinogen abuse with hallucinogen-induced psychotic disorder with hallucinations |  |
| F16159 | | 10 |  | Hallucinogen abuse with hallucinogen-induced psychotic disorder, unspecified |  |
| F16180 | | 10 |  | Hallucinogen abuse with hallucinogen-induced anxiety disorder |  |
| F16183 | | 10 |  | Hallucinogen abuse with hallucinogen persisting perception disorder (flashbacks) |  |
| F16188 | | 10 |  | Hallucinogen abuse with other hallucinogen-induced disorder |  |
| F1619 | | 10 |  | Hallucinogen abuse with unspecified hallucinogen-induced disorder |  |
| F1620 | | 10 |  | Hallucinogen dependence, uncomplicated |  |
| F1621 | | 10 |  | Hallucinogen dependence, in remission |  |
| F16220 | | 10 |  | Hallucinogen dependence with intoxication, uncomplicated |  |
| F16221 | | 10 |  | Hallucinogen dependence with intoxication with delirium |  |
| F16229 | | 10 |  | Hallucinogen dependence with intoxication, unspecified |  |
| F1624 | | 10 |  | Hallucinogen dependence with hallucinogen-induced mood disorder |  |
| F16250 | | 10 |  | Hallucinogen dependence with hallucinogen-induced psychotic disorder with delusions |  |
| F16251 | | 10 |  | Hallucinogen dependence with hallucinogen-induced psychotic disorder with hallucinations |  |
| F16259 | | 10 |  | Hallucinogen dependence with hallucinogen-induced psychotic disorder, unspecified |  |
| F16280 | | 10 |  | Hallucinogen dependence with hallucinogen-induced anxiety disorder |  |
| F16283 | | 10 |  | Hallucinogen dependence with hallucinogen persisting perception disorder (flashbacks) |  |
| F16288 | | 10 |  | Hallucinogen dependence with other hallucinogen-induced disorder |  |
| F1629 | | 10 |  | Hallucinogen dependence with unspecified hallucinogen-induced disorder |  |
| F1690 | | 10 |  | Hallucinogen use, unspecified, uncomplicated |  |
| F16920 | | 10 |  | Hallucinogen use, unspecified with intoxication, uncomplicated |  |
| F16921 | | 10 |  | Hallucinogen use, unspecified with intoxication with delirium |  |
| F16929 | | 10 |  | Hallucinogen use, unspecified with intoxication, unspecified |  |
| F1694 | | 10 |  | Hallucinogen use, unspecified with hallucinogen-induced mood disorder |  |
| F16950 | | 10 |  | Hallucinogen use, unspecified with hallucinogen-induced psychotic disorder with delusions |  |
| F16951 | | 10 |  | Hallucinogen use, unspecified with hallucinogen-induced psychotic disorder with hallucinations |  |
| F16959 | | 10 |  | Hallucinogen use, unspecified with hallucinogen-induced psychotic disorder, unspecified |  |
| F16980 | | 10 |  | Hallucinogen use, unspecified with hallucinogen-induced anxiety disorder |  |
| F16983 | | 10 |  | Hallucinogen use, unspecified with hallucinogen persisting perception disorder (flashbacks) |  |
| F16988 | | 10 |  | Hallucinogen use, unspecified with other hallucinogen-induced disorder |  |
| F1699 | | 10 |  | Hallucinogen use, unspecified with unspecified hallucinogen-induced disorder |  |
| F17200 | | 10 |  | Nicotine dependence, unspecified, uncomplicated |  |
| F17201 | | 10 |  | Nicotine dependence, unspecified, in remission |  |
| F17203 | | 10 |  | Nicotine dependence unspecified, with withdrawal |  |
| F17208 | | 10 |  | Nicotine dependence, unspecified, with other nicotine-induced disorders |  |
| F17209 | | 10 |  | Nicotine dependence, unspecified, with unspecified nicotine-induced disorders |  |
| F17210 | | 10 |  | Nicotine dependence, cigarettes, uncomplicated |  |
| F17211 | | 10 |  | Nicotine dependence, cigarettes, in remission |  |
| F17213 | | 10 |  | Nicotine dependence, cigarettes, with withdrawal |  |
| F17218 | | 10 |  | Nicotine dependence, cigarettes, with other nicotine-induced disorders |  |
| F17219 | | 10 |  | Nicotine dependence, cigarettes, with unspecified nicotine-induced disorders |  |
| F17220 | | 10 |  | Nicotine dependence, chewing tobacco, uncomplicated |  |
| F17221 | | 10 |  | Nicotine dependence, chewing tobacco, in remission |  |
| F17223 | | 10 |  | Nicotine dependence, chewing tobacco, with withdrawal |  |
| F17228 | | 10 |  | Nicotine dependence, chewing tobacco, with other nicotine-induced disorders |  |
| F17229 | | 10 |  | Nicotine dependence, chewing tobacco, with unspecified nicotine-induced disorders |  |
| F17290 | | 10 |  | Nicotine dependence, other tobacco product, uncomplicated |  |
| F17291 | | 10 |  | Nicotine dependence, other tobacco product, in remission |  |
| F17293 | | 10 |  | Nicotine dependence, other tobacco product, with withdrawal |  |
| F17298 | | 10 |  | Nicotine dependence, other tobacco product, with other nicotine-induced disorders |  |
| F17299 | | 10 |  | Nicotine dependence, other tobacco product, with unspecified nicotine-induced disorders |  |
| F1910 | | 10 |  | Other psychoactive substance abuse, uncomplicated |  |
| F1911 | | 10 |  | Other psychoactive substance abuse, in remission |  |
| F19120 | | 10 |  | Other psychoactive substance abuse with intoxication, uncomplicated |  |
| F19121 | | 10 |  | Other psychoactive substance abuse with intoxication delirium |  |
| F19122 | | 10 |  | Other psychoactive substance abuse with intoxication with perceptual disturbances |  |
| F19129 | | 10 |  | Other psychoactive substance abuse with intoxication, unspecified |  |
| F1914 | | 10 |  | Other psychoactive substance abuse with psychoactive substance-induced mood disorder |  |
| F19150 | | 10 |  | Other psychoactive substance abuse with psychoactive substance-induced psychotic disorder with delusions |  |
| F19151 | | 10 |  | Other psychoactive substance abuse with psychoactive substance-induced psychotic disorder with hallucinations |  |
| F19159 | | 10 |  | Other psychoactive substance abuse with psychoactive substance-induced psychotic disorder, unspecified |  |
| F1916 | | 10 |  | Other psychoactive substance abuse with psychoactive substance-induced persisting amnestic disorder |  |
| F1917 | | 10 |  | Other psychoactive substance abuse with psychoactive substance-induced persisting dementia |  |
| F19180 | | 10 |  | Other psychoactive substance abuse with psychoactive substance-induced anxiety disorder |  |
| F19181 | | 10 |  | Other psychoactive substance abuse with psychoactive substance-induced sexual dysfunction |  |
| F19182 | | 10 |  | Other psychoactive substance abuse with psychoactive substance-induced sleep disorder |  |
| F19188 | | 10 |  | Other psychoactive substance abuse with other psychoactive substance-induced disorder |  |
| F1919 | | 10 |  | Other psychoactive substance abuse with unspecified psychoactive substance-induced disorder |  |
| F1920 | | 10 |  | Other psychoactive substance dependence, uncomplicated |  |
| F1921 | | 10 |  | Other psychoactive substance dependence, in remission |  |
| F19220 | | 10 |  | Other psychoactive substance dependence with intoxication, uncomplicated |  |
| F19221 | | 10 |  | Other psychoactive substance dependence with intoxication delirium |  |
| F19222 | | 10 |  | Other psychoactive substance dependence with intoxication with perceptual disturbance |  |
| F19229 | | 10 |  | Other psychoactive substance dependence with intoxication, unspecified |  |
| F19230 | | 10 |  | Other psychoactive substance dependence with withdrawal, uncomplicated |  |
| F19231 | | 10 |  | Other psychoactive substance dependence with withdrawal delirium |  |
| F19232 | | 10 |  | Other psychoactive substance dependence with withdrawal with perceptual disturbance |  |
| F458 | | 10 |  | Other somatoform disorders |  |
| F19239 | | 10 |  | Other psychoactive substance dependence with withdrawal, unspecified |  |
| F1924 | | 10 |  | Other psychoactive substance dependence with psychoactive substance-induced mood disorder |  |
| F19250 | | 10 |  | Other psychoactive substance dependence with psychoactive substance-induced psychotic disorder with delusions |  |
| F19251 | | 10 |  | Other psychoactive substance dependence with psychoactive substance-induced psychotic disorder with hallucinations |  |
| F19259 | | 10 |  | Other psychoactive substance dependence with psychoactive substance-induced psychotic disorder, unspecified |  |
| F1926 | | 10 |  | Other psychoactive substance dependence with psychoactive substance-induced persisting amnestic disorder |  |
| F1927 | | 10 |  | Other psychoactive substance dependence with psychoactive substance-induced persisting dementia |  |
| F19280 | | 10 |  | Other psychoactive substance dependence with psychoactive substance-induced anxiety disorder |  |
| F19281 | | 10 |  | Other psychoactive substance dependence with psychoactive substance-induced sexual dysfunction |  |
| F19282 | | 10 |  | Other psychoactive substance dependence with psychoactive substance-induced sleep disorder |  |
| F19288 | | 10 |  | Other psychoactive substance dependence with other psychoactive substance-induced disorder |  |
| F1929 | | 10 |  | Other psychoactive substance dependence with unspecified psychoactive substance-induced disorder |  |
| F1990 | | 10 |  | Other psychoactive substance use, unspecified, uncomplicated |  |
| F19920 | | 10 |  | Other psychoactive substance use, unspecified with intoxication, uncomplicated |  |
| F19921 | | 10 |  | Other psychoactive substance use, unspecified with intoxication with delirium |  |
| F19922 | | 10 |  | Other psychoactive substance use, unspecified with intoxication with perceptual disturbance |  |
| F19929 | | 10 |  | Other psychoactive substance use, unspecified with intoxication, unspecified |  |
| F19930 | | 10 |  | Other psychoactive substance use, unspecified with withdrawal, uncomplicated |  |
| F19931 | | 10 |  | Other psychoactive substance use, unspecified with withdrawal delirium |  |
| F19932 | | 10 |  | Other psychoactive substance use, unspecified with withdrawal with perceptual disturbance |  |
| F19939 | | 10 |  | Other psychoactive substance use, unspecified with withdrawal, unspecified |  |
| F1994 | | 10 |  | Other psychoactive substance use, unspecified with psychoactive substance-induced mood disorder |  |
| F19950 | | 10 |  | Other psychoactive substance use, unspecified with psychoactive substance-induced psychotic disorder with delusions |  |
| F19951 | | 10 |  | Other psychoactive substance use, unspecified with psychoactive substance-induced psychotic disorder with hallucinations |  |
| F19959 | | 10 |  | Other psychoactive substance use, unspecified with psychoactive substance-induced psychotic disorder, unspecified |  |
| F1996 | | 10 |  | Other psychoactive substance use, unspecified with psychoactive substance-induced persisting amnestic disorder |  |
| F1997 | | 10 |  | Other psychoactive substance use, unspecified with psychoactive substance-induced persisting dementia |  |
| F19980 | | 10 |  | Other psychoactive substance use, unspecified with psychoactive substance-induced anxiety disorder |  |
| F19981 | | 10 |  | Other psychoactive substance use, unspecified with psychoactive substance-induced sexual dysfunction |  |
| F19982 | | 10 |  | Other psychoactive substance use, unspecified with psychoactive substance-induced sleep disorder |  |
| F19988 | | 10 |  | Other psychoactive substance use, unspecified with other psychoactive substance-induced disorder |  |
| F1999 | | 10 |  | Other psychoactive substance use, unspecified with unspecified psychoactive substance-induced disorder |  |
|  | |  |  |  |  |
