# Supplementary material for: Relationship between Nonhepatic Serum Ammonia Levels and Sepsis-Associated Encephalopathy: A Retrospective Cohort Study
Source: Emerg Med Int. 2023 Oct 12;2023:6676033. doi: 10.1155/2023/6676033 (PMC10590267; doi:10.1155/2023/6676033)
Supplement: Supplementary Materials — 1: exclude patients with trauma of the skull from the MIMIC IV database according to ICD codes. Supplementary materials 2: exclude patients with intracerebral hemorrhage, cerebral embolism, and ischemic stroke disease from the MIMIC IV database according to ICD codes. Supplementary materials 3: exclude patients with meningitis and encephalitis disease from the MIMIC IV database according to ICD codes. Supplementary materials 4: exclude patients with epilepsy disease from the MIMIC IV database according to ICD codes. Supplementary materials 5: exclude patients with other cerebrovascular disease from the MIMIC IV database according to ICD codes. Supplementary materials 6: exclude patients with mental disorders and neurological disease from the MIMIC IV database according to ICD codes. Supplementary materials 7: exclude patients with alcoholic intoxication or drug abuse from the MIMIC IV database according to ICD codes. Supplementary materials 8: exclude patients with metabolic encephalopathy, hepatic encephalopathy, hypertensive encephalopathy, diabetes with coma, disorders of urea cycle, hypernatremia, and Wernicke's encephalopathy from the MIMIC IV database according to ICD codes. Supplementary materials 9: exclude patients with acute and chronic liver disease. Supplementary materials 10: hypertension disease and ICD codes. Supplementary materials 11: diabetes disease and ICD codes. Supplementary materials 12: lung disease and ICD codes. Supplementary materials 13: cardiovascular diseases and ICD codes. Supplementary materials 14: renal disease from the MIMIC IV database according to ICD codes. Supplementary materials 15: the standardized mean differences of the original cohort were compared with those of the IPW cohorts in sepsis patients. SMD: standardized mean differences. [file 6676033.f1.zip › Supplementary materials.8.docx]

|  | **Supplementary materials 8** Exclude patients with metabolic encephalopathy, hepatic encephalopathy, hypertensive encephalopathy, diabetes with coma, disorders of urea cycle, hypernatremia, wrnicke's encephalopathy from the MIMIC IV database according to ICD-codes | | | |
| --- | --- | --- | --- | --- |
|  | | ICD |  | Description |
| 34831 | | 9 |  | Metabolic encephalopathy |
| 5722 | | 9 |  | Hepatic encephalopathy |
| 700 | | 9 |  | Viral hepatitis A with hepatic coma |
| 7020 | | 9 |  | Viral hepatitis B with hepatic coma, acute or unspecified, without mention of hepatitis delta |
| 7021 | | 9 |  | Viral hepatitis B with hepatic coma, acute or unspecified, with hepatitis delta |
| 7022 | | 9 |  | Chronic viral hepatitis B with hepatic coma without hepatitis delta |
| 7023 | | 9 |  | Chronic viral hepatitis B with hepatic coma with hepatitis delta |
| 7041 | | 9 |  | Acute hepatitis C with hepatic coma |
| 7042 | | 9 |  | Hepatitis delta without mention of active hepatitis B disease with hepatic coma |
| 7043 | | 9 |  | Hepatitis E with hepatic coma |
| 7044 | | 9 |  | Chronic hepatitis C with hepatic coma |
| 7049 | | 9 |  | Other specified viral hepatitis with hepatic coma |
| 7052 | | 9 |  | Hepatitis delta without mention of active hepatitis B disease or hepatic coma |
| 706 | | 9 |  | Unspecified viral hepatitis with hepatic coma |
| 7071 | | 9 |  | Unspecified viral hepatitis C with hepatic coma |
| 2706 | | 9 |  | Disorders of urea cycle metabolism |
| 2510 | | 9 |  | Hypoglycemic coma |
| 4372 | | 9 |  | Hypertensive encephalopathy |
| G9341 | | 10 |  | Metabolic encephalopathy |
| B150 | | 10 |  | Hepatitis A with hepatic coma |
| B159 | | 10 |  | Hepatitis A without hepatic coma |
| B160 | | 10 |  | Acute hepatitis B with delta-agent with hepatic coma |
| B161 | | 10 |  | Acute hepatitis B with delta-agent without hepatic coma |
| B162 | | 10 |  | Acute hepatitis B without delta-agent with hepatic coma |
| B169 | | 10 |  | Acute hepatitis B without delta-agent and without hepatic coma |
| B170 | | 10 |  | Acute delta-(super) infection of hepatitis B carrier |
| B1710 | | 10 |  | Acute hepatitis C without hepatic coma |
| B1711 | | 10 |  | Acute hepatitis C with hepatic coma |
| B190 | | 10 |  | Unspecified viral hepatitis with hepatic coma |
| B1910 | | 10 |  | Unspecified viral hepatitis B without hepatic coma |
| B1911 | | 10 |  | Unspecified viral hepatitis B with hepatic coma |
| B1920 | | 10 |  | Unspecified viral hepatitis C without hepatic coma |
| B1921 | | 10 |  | Unspecified viral hepatitis C with hepatic coma |
| B199 | | 10 |  | Unspecified viral hepatitis without hepatic coma |
| E7220 | | 10 |  | Disorder of urea cycle metabolism, unspecified |
| E7229 | | 10 |  | Other disorders of urea cycle metabolism |
| E15 | | 10 |  | Nondiabetic hypoglycemic coma |
| E160 | | 10 |  | Drug-induced hypoglycemia without coma |
| E161 | | 10 |  | Other hypoglycemia |
| E162 | | 10 |  | Hypoglycemia, unspecified |
| E512 | | 10 |  | Wernicke's encephalopathy |
| G92 | | 10 |  | Toxic encephalopathy |
| G9341 | | 10 |  | Metabolic encephalopathy |
| I674 | | 10 |  | Hypertensive encephalopathy |
| P9160 | | 10 |  | Hypoxic ischemic encephalopathy [HIE], unspecified |
| P9161 | | 10 |  | Mild hypoxic ischemic encephalopathy [HIE] |
| P9162 | | 10 |  | Moderate hypoxic ischemic encephalopathy [HIE] |
| P9163 | | 10 |  | Severe hypoxic ischemic encephalopathy [HIE] |
