# Supplementary material for: Relationship between Nonhepatic Serum Ammonia Levels and Sepsis-Associated Encephalopathy: A Retrospective Cohort Study
Source: Emerg Med Int. 2023 Oct 12;2023:6676033. doi: 10.1155/2023/6676033 (PMC10590267; doi:10.1155/2023/6676033)
Supplement: Supplementary Materials — 1: exclude patients with trauma of the skull from the MIMIC IV database according to ICD codes. Supplementary materials 2: exclude patients with intracerebral hemorrhage, cerebral embolism, and ischemic stroke disease from the MIMIC IV database according to ICD codes. Supplementary materials 3: exclude patients with meningitis and encephalitis disease from the MIMIC IV database according to ICD codes. Supplementary materials 4: exclude patients with epilepsy disease from the MIMIC IV database according to ICD codes. Supplementary materials 5: exclude patients with other cerebrovascular disease from the MIMIC IV database according to ICD codes. Supplementary materials 6: exclude patients with mental disorders and neurological disease from the MIMIC IV database according to ICD codes. Supplementary materials 7: exclude patients with alcoholic intoxication or drug abuse from the MIMIC IV database according to ICD codes. Supplementary materials 8: exclude patients with metabolic encephalopathy, hepatic encephalopathy, hypertensive encephalopathy, diabetes with coma, disorders of urea cycle, hypernatremia, and Wernicke's encephalopathy from the MIMIC IV database according to ICD codes. Supplementary materials 9: exclude patients with acute and chronic liver disease. Supplementary materials 10: hypertension disease and ICD codes. Supplementary materials 11: diabetes disease and ICD codes. Supplementary materials 12: lung disease and ICD codes. Supplementary materials 13: cardiovascular diseases and ICD codes. Supplementary materials 14: renal disease from the MIMIC IV database according to ICD codes. Supplementary materials 15: the standardized mean differences of the original cohort were compared with those of the IPW cohorts in sepsis patients. SMD: standardized mean differences. [file 6676033.f1.zip › Supplementary materials.9.docx]

| Supplementary materials 9 Exclude patients with acute and chronic liver disease | | | | | | | | | | | |
| --- | --- | --- | --- | --- | --- | --- | --- | --- | --- | --- | --- |
| ICD | | Description |  |  |  |  |  |  |  |  |  |
| 700 | 9 | Hepatitis A with coma | |  |  |  |  |  |  |  |  |
| 0701 | 9 | Viral hepatitis A without mention of hepatic coma | | | | | | | |  |  |
| 07020 | 9 | Viral hepatitis B with hepatic coma, acute or unspecified, without mention of hepatitis delta | | | | | | | |  |  |
| 07021 | 9 | Viral hepatitis B with hepatic coma, acute or unspecified, with hepatitis delta | | | | | | |  |  |  |
| 07022 | 9 | Chronic viral hepatitis B with hepatic coma without hepatitis delta | | | | | |  |  |  |  |
| 07023 | 9 | Chronic viral hepatitis B with hepatic coma with hepatitis delta | | | | | | | | | |
| 07030 | 9 | Viral hepatitis B without mention of hepatic coma, acute or unspecified, without mention of hepatitis | | | | | | | | |  |
| 07031 | 9 | Viral hepatitis B without mention of hepatic coma, acute or unspecified, with hepatitis delta | | | | | | | |  |  |
| 07032 | 9 | Chronic viral hepatitis B without mention of hepatic coma without mention of hepatitis delta | | | | | | | |  |  |
| 07033 | 9 | Chronic viral hepatitis B without mention of hepatic coma with hepatitis delta | | | | | | |  |  |  |
| 07041 | 9 | Acute hepatitis C with hepatic coma | | | | | | | | | |
| 07042 | 9 | Hepatitis delta without mention of active hepatitis B disease with hepatic coma | | | | | | |  |  |  |
| 07043 | 9 | Hepatitis E with hepatic coma | | | | | | | | |  |
| 07044 | 9 | Chronic hepatitis C with hepatic coma | | | | | | | | |  |
| 07049 | 9 | Other specified viral hepatitis with hepatic coma | | | | | | | | |  |
| 07051 | 9 | Acute hepatitis C without mention of hepatic coma | | | | | | | | |  |
| 07052 | 9 | Hepatitis delta without mention of active hepatitis B disease or hepatic coma | | | | | | |  |  |  |
| 07053 | 9 | Hepatitis E without mention of hepatic coma | | | |  |  |  |  |  |  |
| 07054 | 9 | Chronic hepatitis C without mention of hepatic coma | | | | | | | |  |  |
| 07059 | 9 | Other specified viral hepatitis without mention of hepatic coma | | | | | | | |  |  |
| 0706 | 9 | Unspecified viral hepatitis with hepatic coma | | | |  |  |  |  |  |  |
| 07070 | 9 | Unspecified viral hepatitis C without hepatic coma | | | | | | | | |  |
| 07071 | 9 | Unspecified viral hepatitis C with hepatic coma | | | |  |  |  |  |  |  |
| 0709 | 9 | Unspecified viral hepatitis without mention of hepatic coma | | | | |  |  |  |  |  |
| 5712 | 9 | Alcoholic cirrhosis of liver | | | | | | |  |  |  |
| 5713 | 9 | Alcoholic liver damage, unspecified | | | | | | | |  |  |
| 57140 | 9 | Chronic hepatitis, unspecified | | | | | | | | | |
| 57141 | 9 | Chronic persistent hepatitis | | | | | | | |  |  |
| 57142 | 9 | Autoimmune hepatitis | |  |  |  |  |  |  |  |  |
| 57149 | 9 | Other chronic hepatitis | |  |  |  |  |  |  |  |  |
| 5715 | 9 | Cirrhosis of liver without mention of alcohol | | | |  |  |  |  |  |  |
| 5716 | 9 | Biliary cirrhosis | | | | | | |  |  |  |
| 5718 | 9 | Other chronic nonalcoholic liver disease | | | | | | | | | |
| 5719 | 9 | Unspecified chronic liver disease without mention of alcohol | | | | | | | |  |  |
| 5722 | 9 | Hepatic encephalopathy | | | | | | | | | |
| 5724 | 9 | Hepatorenal syndrome | |  |  |  |  |  |  |  |  |
| 5728 | 9 | Other sequelae of chronic liver disease | | | | | | |  |  |  |
| 5738 | 9 | Other specified disorders of liver | | |  |  |  |  |  |  |  |
| 5735 | 9 | Hepatopulmonary syndrome | | | | | | | | | |
| 5734 | 9 | Hepatic infarction | | | | | | | | | |
| 5733 | 9 | Hepatitis, unspecified | |  |  |  |  |  |  |  |  |
| 5732 | 9 | Hepatitis in other infectious diseases classified elsewhere | | | | |  |  |  |  |  |
| 5731 | 9 | Hepatitis in viral diseases classified elsewhere | | | |  |  |  |  |  |  |
| 5730 | 9 | Chronic passive congestion of liver | | |  |  |  |  |  |  |  |
| V0260 | 9 | Viral hepatitis carrier, unspecified | | |  |  |  |  |  |  |  |
| V0261 | 9 | Hepatitis B | carrier |  |  |  |  |  |  |  |  |
| V0262 | 9 | Hepatitis C | carrier |  |  |  |  |  |  |  |  |
| V0269 | 9 | Other viral hepatitis carrier | | | | |  |  |  |  |  |
| 86400 | 9 | Injury to liver without mention of open wound into cavity, unspecified injury | | | | | | |  |  |  |
| 86401 | 9 | Injury to liver without mention of open wound into cavity, hematoma and contusion | | | | | | |  |  |  |
| 86402 | 9 | Injury to liver without mention of open wound into cavity, laceration, minor | | | | | | |  |  |  |
| 86403 | 9 | Injury to liver without mention of open wound into cavity, laceration, moderate | | | | | | |  |  |  |
| 86404 | 9 | Injury to liver without mention of open wound into cavity, laceration, major | | | | | | |  |  |  |
| 86405 | 9 | Injury to liver without mention of open wound into cavity laceration, unspecified | | | | | | |  |  |  |
| 86409 | 9 | Other injury to liver without mention of open wound into cavity | | | | | | |  |  |  |
| 86410 | 9 | Injury to liver with open wound into cavity, unspecified injury | | | | | | | |  |  |
| 4560 | 9 | Esophageal varices with bleeding | | |  |  |  |  |  |  |  |
| 4561 | 9 | Esophageal varices without mention of bleeding | | | | | | | |  |  |
| 45620 | 9 | Esophageal varices in diseases classified elsewhere, with bleeding | | | | | |  |  |  |  |
| B0081 | 10 | Herpesviral hepatitis | | | | | |  |  |  |  |
| A064 | 10 | Amebic liver abscess | | | | | |  |  |  |  |
| B150 | 10 | Hepatitis A with hepatic coma | | | | | |  |  |  |  |
| B159 | 10 | Hepatitis A without hepatic coma | | | | | |  |  |  |  |
| B160 | 10 | Acute hepatitis B with delta-agent with hepatic coma | | | | | |  |  |  |  |
| B161 | 10 | Acute hepatitis B with delta-agent without hepatic coma | | | | | |  |  |  |  |
| B162 | 10 | Acute hepatitis B without delta-agent with hepatic coma | | | | | |  |  |  |  |
| B169 | 10 | Acute hepatitis B without delta-agent and without hepatic coma | | | | | |  |  |  |  |
| B170 | 10 | Acute delta-(super) infection of hepatitis B carrier | | | | | |  |  |  |  |
| B1710 | 10 | Acute hepatitis C without hepatic coma | | | | | |  |  |  |  |
| B1711 | 10 | Acute hepatitis C with hepatic coma | | | | | |  |  |  |  |
| B172 | 10 | Acute hepatitis E | | | | | |  |  |  |  |
| B178 | 10 | Other specified acute viral hepatitis | | | | | |  |  |  |  |
| B179 | 10 | Acute viral hepatitis, unspecified | | | | | |  |  |  |  |
| B180 | 10 | Chronic viral hepatitis B with delta-agent | | | | | |  |  |  |  |
| B181 | 10 | Chronic viral hepatitis B without delta-agent | | | | | |  |  |  |  |
| B182 | 10 | Chronic viral hepatitis C | | | | | |  |  |  |  |
| B188 | 10 | Other chronic viral hepatitis | | | | | |  |  |  |  |
| B189 | 10 | Chronic viral hepatitis, unspecified | | | | | |  |  |  |  |
| B190 | 10 | Unspecified viral hepatitis with hepatic coma | | | | | |  |  |  |  |
| B1910 | 10 | Unspecified viral hepatitis B without hepatic coma | | | | | |  |  |  |  |
| B1911 | 10 | Unspecified viral hepatitis B with hepatic coma | | | | | |  |  |  |  |
| B1920 | 10 | Unspecified viral hepatitis C without hepatic coma | | | | | |  |  |  |  |
| B1921 | 10 | Unspecified viral hepatitis C with hepatic coma | | | | | |  |  |  |  |
| B199 | 10 | Unspecified viral hepatitis without hepatic coma | | | | | |  |  |  |  |
| B251 | 10 | Cytomegaloviral hepatitis | | | | | |  |  |  |  |
| B2681 | 10 | Mumps hepatitis | | | | | |  |  |  |  |
| B581 | 10 | Toxoplasma hepatitis | | | | | |  |  |  |  |
| B942 | 10 | Sequelae of viral hepatitis | | | | | |  |  |  |  |
| K7010 | 10 | Alcoholic hepatitis without ascites | | | | | |  |  |  |  |
| K7011 | 10 | Alcoholic hepatitis with ascites | | | | | |  |  |  |  |
| K702 | 10 | Alcoholic fibrosis and sclerosis of liver | | | | | |  |  |  |  |
| K7030 | 10 | Alcoholic cirrhosis of liver without ascites | | | | | |  |  |  |  |
| K7031 | 10 | Alcoholic cirrhosis of liver with ascites | | | | | |  |  |  |  |
| K7040 | 10 | Alcoholic hepatic failure without coma | | | | | |  |  |  |  |
| K7041 | 10 | Alcoholic hepatic failure with coma | | | | | |  |  |  |  |
| K709 | 10 | Alcoholic liver disease, unspecified | | | | | |  |  |  |  |
| K710 | 10 | Toxic liver disease with cholestasis | | | | | |  |  |  |  |
| K7110 | 10 | Toxic liver disease with hepatic necrosis, without coma | | | | | |  |  |  |  |
| K7111 | 10 | Toxic liver disease with hepatic necrosis, with coma | | | | | |  |  |  |  |
| K712 | 10 | Toxic liver disease with acute hepatitis | | | | | |  |  |  |  |
| K713 | 10 | Toxic liver disease with chronic persistent hepatitis | | | | | |  |  |  |  |
| K714 | 10 | Toxic liver disease with chronic lobular hepatitis | | | | | |  |  |  |  |
| K7150 | 10 | Toxic liver disease with chronic active hepatitis without ascites | | | | | |  |  |  |  |
| K7151 | 10 | Toxic liver disease with chronic active hepatitis with ascites | | | | | |  |  |  |  |
| K716 | 10 | Toxic liver disease with hepatitis, not elsewhere classified | | | | | |  |  |  |  |
| K717 | 10 | Toxic liver disease with fibrosis and cirrhosis of liver | | | | | |  |  |  |  |
| K718 | 10 | Toxic liver disease with other disorders of liver | | | | | |  |  |  |  |
| K719 | 10 | Toxic liver disease, unspecified | | | | | |  |  |  |  |
| K7200 | 10 | Acute and subacute hepatic failure without coma | | | | | |  |  |  |  |
| K7201 | 10 | Acute and subacute hepatic failure with coma | | | | | |  |  |  |  |
| K7210 | 10 | Chronic hepatic failure without coma | | | | | |  |  |  |  |
| K7211 | 10 | Chronic hepatic failure with coma | | | | | |  |  |  |  |
| K7290 | 10 | Hepatic failure, unspecified without coma | | | | | |  |  |  |  |
| K7291 | 10 | Hepatic failure, unspecified with coma | | | | | |  |  |  |  |
| K730 | 10 | Chronic persistent hepatitis, not elsewhere classified | | | | | |  |  |  |  |
| K731 | 10 | Chronic lobular hepatitis, not elsewhere classified | | | | | |  |  |  |  |
| K732 | 10 | Chronic active hepatitis, not elsewhere classified | | | | | |  |  |  |  |
| K738 | 10 | Other chronic hepatitis, not elsewhere classified | | | | | |  |  |  |  |
| K739 | 10 | Chronic hepatitis, unspecified | | | | | |  |  |  |  |
| K740 | 10 | Hepatic fibrosis | | | | | |  |  |  |  |
| K741 | 10 | Hepatic sclerosis | | | | | |  |  |  |  |
| K742 | 10 | Hepatic fibrosis with hepatic sclerosis | | | | | |  |  |  |  |
| K743 | 10 | Primary biliary cirrhosis | | | | | |  |  |  |  |
| K744 | 10 | Secondary biliary cirrhosis | | | | | |  |  |  |  |
| K7460 | 10 | Unspecified cirrhosis of liver | | | | | |  |  |  |  |
| K7469 | 10 | Other cirrhosis of liver | | | | | |  |  |  |  |
| K750 | 10 | Abscess of liver | | | | | |  |  |  |  |
| K751 | 10 | Phlebitis of portal vein | | | | | |  |  |  |  |
| K752 | 10 | Nonspecific reactive hepatitis | | | | | |  |  |  |  |
| K753 | 10 | Granulomatous hepatitis, not elsewhere classified | | | | | |  |  |  |  |
| K754 | 10 | Autoimmune hepatitis | | | | | |  |  |  |  |
| K7581 | 10 | Nonalcoholic steatohepatitis (NASH) | | | | | |  |  |  |  |
| K7589 | 10 | Other specified inflammatory liver diseases | | | | | |  |  |  |  |
| K759 | 10 | Inflammatory liver disease, unspecified | | | | | |  |  |  |  |
| K760 | 10 | Fatty (change of) liver, not elsewhere classified | | | | | |  |  |  |  |
| K761 | 10 | Chronic passive congestion of liver | | | | | |  |  |  |  |
| K762 | 10 | Central hemorrhagic necrosis of liver | | | | | |  |  |  |  |
| K763 | 10 | Infarction of liver | | | | | |  |  |  |  |
| K764 | 10 | Peliosis hepatis | | | | | |  |  |  |  |
| K765 | 10 | Hepatic veno-occlusive disease | | | | | |  |  |  |  |
| K7689 | 10 | Other specified diseases of liver | | | | | |  |  |  |  |
| K769 | 10 | Liver disease, unspecified | | | | | |  |  |  |  |
| K77 | 10 | Liver disorders in diseases classified elsewhere | | | | | |  |  |  |  |
| O9833 | 10 | Other infections with a predominantly sexual mode of transmission complicating the puerperium | | | | | |  |  |  |  |
| O98411 | 10 | Viral hepatitis complicating pregnancy, first trimester | | | | | |  |  |  |  |
| O98412 | 10 | Viral hepatitis complicating pregnancy, second trimester | | | | | |  |  |  |  |
| O98413 | 10 | Viral hepatitis complicating pregnancy, third trimester | | | | | |  |  |  |  |
| O98419 | 10 | Viral hepatitis complicating pregnancy, unspecified trimester | | | | | |  |  |  |  |
| O9842 | 10 | Viral hepatitis complicating childbirth | | | | | |  |  |  |  |
| O9843 | 10 | Viral hepatitis complicating the puerperium | | | | | |  |  |  |  |
| V0260 | 10 | Viral hepatitis carrier, unspecified | | | | | |  |  |  |  |
| V0261 | 10 | Hepatitis B carrier | | | | | |  |  |  |  |
| V0262 | 10 | Hepatitis C carrier | | | | | |  |  |  |  |
| V0269 | 10 | Other viral hepatitis carrier | | | | | |  |  |  |  |
| V0260 | 10 | Viral hepatitis carrier, unspecified | | | | | |  |  |  |  |
| V0261 | 10 | Hepatitis B carrier | | | | | |  |  |  |  |
| V0262 | 10 | Hepatitis C carrier | | | | | |  |  |  |  |
| V0269 | 10 | Other viral hepatitis carrier | | | | | |  |  |  |  |
| A5274 | 10 | Syphilis of liver and other viscera | | | | | |  |  |  |  |
| C220 | 10 | Liver cell carcinoma | | | | | |  |  |  |  |
| C787 | 10 | Secondary malignant neoplasm of liver and intrahepatic bile duct | | | | | |  |  |  |  |
| E9341 | 10 | Liver preparations and other antianemic agents causing adverse effects in therapeutic use | | | | | |  |  |  |  |
| O26611 | 10 | Liver and biliary tract disorders in pregnancy, first trimester | | | | | |  |  |  |  |
| O26612 | 10 | Liver and biliary tract disorders in pregnancy, second trimester | | | | | |  |  |  |  |
| O26613 | 10 | Liver and biliary tract disorders in pregnancy, third trimester | | | | | |  |  |  |  |
| O26619 | 10 | Liver and biliary tract disorders in pregnancy, unspecified trimester | | | | | |  |  |  |  |
| O2662 | 10 | Liver and biliary tract disorders in childbirth | | | | | |  |  |  |  |
| O2663 | 10 | Liver and biliary tract disorders in the puerperium | | | | | |  |  |  |  |
| P7884 | 10 | Gestational alloimmune liver disease | | | | | |  |  |  |  |
| T8640 | 10 | Unspecified complication of liver transplant | | | | | |  |  |  |  |
| T8641 | 10 | Liver transplant rejection | | | | | |  |  |  |  |
| T8642 | 10 | Liver transplant failure | | | | | |  |  |  |  |
| T8643 | 10 | Liver transplant infection | | | | | |  |  |  |  |
| T8649 | 10 | Other complications of liver transplant | | | | | |  |  |  |  |
| T865 | 10 | Complications of stem cell transplant | | | | | |  |  |  |  |
| V427 | 10 | Liver replaced by transplant | | | | | |  |  |  |  |
| Z526 | 10 | Liver donor | | | | | |  |  |  |  |
| Z4823 | 10 | Encounter for aftercare following liver transplant | | | | | |  |  |  |  |
| Z944 | 10 | Liver transplant status | | | | | |  |  |  |  |
| C221 | 10 | Intrahepatic bile duct carcinoma | | | | | |  |  |  |  |
| D135 | 10 | Benign neoplasm of extrahepatic bile ducts | | | | | |  |  |  |  |
| E8021 | 10 | Acute intermittent (hepatic) porphyria | | | | | |  |  |  |  |
| K9182 | 10 | Postprocedural hepatic failure | | | | | |  |  |  |  |
| Q266 | 10 | Portal vein-hepatic artery fistula | | | | | |  |  |  |  |
| 45621 | 9 | Esophageal varices in diseases classified elsewhere, without mention of bleeding | | | | | | |  |  |  |

**^[[1]](#footnote-1)^**

1. [↑](#footnote-ref-1)
