# Supplementary material for: Relationship between Nonhepatic Serum Ammonia Levels and Sepsis-Associated Encephalopathy: A Retrospective Cohort Study
Source: Emerg Med Int. 2023 Oct 12;2023:6676033. doi: 10.1155/2023/6676033 (PMC10590267; doi:10.1155/2023/6676033)
Supplement: Supplementary Materials — 1: exclude patients with trauma of the skull from the MIMIC IV database according to ICD codes. Supplementary materials 2: exclude patients with intracerebral hemorrhage, cerebral embolism, and ischemic stroke disease from the MIMIC IV database according to ICD codes. Supplementary materials 3: exclude patients with meningitis and encephalitis disease from the MIMIC IV database according to ICD codes. Supplementary materials 4: exclude patients with epilepsy disease from the MIMIC IV database according to ICD codes. Supplementary materials 5: exclude patients with other cerebrovascular disease from the MIMIC IV database according to ICD codes. Supplementary materials 6: exclude patients with mental disorders and neurological disease from the MIMIC IV database according to ICD codes. Supplementary materials 7: exclude patients with alcoholic intoxication or drug abuse from the MIMIC IV database according to ICD codes. Supplementary materials 8: exclude patients with metabolic encephalopathy, hepatic encephalopathy, hypertensive encephalopathy, diabetes with coma, disorders of urea cycle, hypernatremia, and Wernicke's encephalopathy from the MIMIC IV database according to ICD codes. Supplementary materials 9: exclude patients with acute and chronic liver disease. Supplementary materials 10: hypertension disease and ICD codes. Supplementary materials 11: diabetes disease and ICD codes. Supplementary materials 12: lung disease and ICD codes. Supplementary materials 13: cardiovascular diseases and ICD codes. Supplementary materials 14: renal disease from the MIMIC IV database according to ICD codes. Supplementary materials 15: the standardized mean differences of the original cohort were compared with those of the IPW cohorts in sepsis patients. SMD: standardized mean differences. [file 6676033.f1.zip › Supplementary materials11.doc]

| **Supplementary materials11** Diabetes disease and ICD-codes | | |  |  |  |  |  |  |  |  |  |
| --- | --- | --- | --- | --- | --- | --- | --- | --- | --- | --- | --- |
| Disease | ICD-Code | Description |  |  |  |  |  |  |  |  |  |
| Diabetes |  |  |  |  |  |  |  |  |  |  |  |
|  | E9323 | Insulins and antidiabetic agents causing adverse effects in therapeutic use |  |  |  |  |  |  |  |  |  |
|  | 9623 | Poisoning by insulins and antidiabetic agents |  |  |  |  |  |  |  |  |  |
|  | 25000 | Diabetes mellitus without mention of complication, type II or unspecified type, not stated as uncontrolled |  |  |  |  |  |  |  |  |  |
|  | 25001 | Diabetes mellitus without mention of complication, type I [juvenile type], not stated as uncontrolled |  |  |  |  |  |  |  |  |  |
|  | 25002 | Diabetes mellitus without mention of complication, type II or unspecified type, uncontrolled |  |  |  |  |  |  |  |  |  |
|  | 25003 | Diabetes mellitus without mention of complication, type I [juvenile type], uncontrolled |  |  |  |  |  |  |  |  |  |
|  | 25010 | Diabetes with ketoacidosis, type II or unspecified type, not stated as uncontrolled |  |  |  |  |  |  |  |  |  |
|  | 25011 | Diabetes with ketoacidosis, type I [juvenile type], not stated as uncontrolled |  |  |  |  |  |  |  |  |  |
|  | 25012 | Diabetes with ketoacidosis, type II or unspecified type, uncontrolled |  |  |  |  |  |  |  |  |  |
|  | 25013 | Diabetes with ketoacidosis, type I [juvenile type], uncontrolled |  |  |  |  |  |  |  |  |  |
|  | 25020 | Diabetes with hyperosmolarity, type II or unspecified type, not stated as uncontrolled |  |  |  |  |  |  |  |  |  |
|  | 25021 | Diabetes with hyperosmolarity, type I [juvenile type], not stated as uncontrolled |  |  |  |  |  |  |  |  |  |
|  | 25022 | Diabetes with hyperosmolarity, type II or unspecified type, uncontrolled |  |  |  |  |  |  |  |  |  |
|  | 25023 | Diabetes with hyperosmolarity, type I [juvenile type], uncontrolled |  |  |  |  |  |  |  |  |  |
|  | 25030 | Diabetes with other coma, type II or unspecified type, not stated as uncontrolled |  |  |  |  |  |  |  |  |  |
|  | 25031 | Diabetes with other coma, type I [juvenile type], not stated as uncontrolled |  |  |  |  |  |  |  |  |  |
|  | 25032 | Diabetes with other coma, type II or unspecified type, uncontrolled |  |  |  |  |  |  |  |  |  |
|  | 25033 | Diabetes with other coma, type I [juvenile type], uncontrolled |  |  |  |  |  |  |  |  |  |
|  | 25040 | Diabetes with renal manifestations, type II or unspecified type, not stated as uncontrolled |  |  |  |  |  |  |  |  |  |
|  | 24900 | Secondary diabetes mellitus without mention of complication, not stated as uncontrolled, or unspecified |  |  |  |  |  |  |  |  |  |
|  | 24901 | Secondary diabetes mellitus without mention of complication, uncontrolled |  |  |  |  |  |  |  |  |  |
|  | 24910 | Secondary diabetes mellitus with ketoacidosis, not stated as uncontrolled, or unspecified |  |  |  |  |  |  |  |  |  |
|  | 24911 | Secondary diabetes mellitus with ketoacidosis, uncontrolled |  |  |  |  |  |  |  |  |  |
|  | 24920 | Secondary diabetes mellitus with hyperosmolarity, not stated as uncontrolled, or unspecified |  |  |  |  |  |  |  |  |  |
|  | 24921 | Secondary diabetes mellitus with hyperosmolarity, uncontrolled |  |  |  |  |  |  |  |  |  |
|  | 24930 | Secondary diabetes mellitus with other coma, not stated as uncontrolled, or unspecified |  |  |  |  |  |  |  |  |  |
|  | 24931 | Secondary diabetes mellitus with other coma, uncontrolled |  |  |  |  |  |  |  |  |  |
|  | 24940 | Secondary diabetes mellitus with renal manifestations, not stated as uncontrolled, or unspecified |  |  |  |  |  |  |  |  |  |
|  | 24941 | Secondary diabetes mellitus with renal manifestations, uncontrolled |  |  |  |  |  |  |  |  |  |
|  | 24950 | Secondary diabetes mellitus with ophthalmic manifestations, not stated as uncontrolled, or unspecified |  |  |  |  |  |  |  |  |  |
|  | 24951 | Secondary diabetes mellitus with ophthalmic manifestations, uncontrolled |  |  |  |  |  |  |  |  |  |
|  | 24960 | Secondary diabetes mellitus with neurological manifestations, not stated as uncontrolled, or unspecified |  |  |  |  |  |  |  |  |  |
|  | 24961 | Secondary diabetes mellitus with neurological manifestations, uncontrolled |  |  |  |  |  |  |  |  |  |
|  | 24970 | Secondary diabetes mellitus with peripheral circulatory disorders, not stated as uncontrolled, or unspecified |  |  |  |  |  |  |  |  |  |
|  | 24971 | Secondary diabetes mellitus with peripheral circulatory disorders, uncontrolled |  |  |  |  |  |  |  |  |  |
|  | 24980 | Secondary diabetes mellitus with other specified manifestations, not stated as uncontrolled, or unspecified |  |  |  |  |  |  |  |  |  |
|  | 24981 | Secondary diabetes mellitus with other specified manifestations, uncontrolled |  |  |  |  |  |  |  |  |  |
|  | 24990 | Secondary diabetes mellitus with unspecified complication, not stated as uncontrolled, or unspecified |  |  |  |  |  |  |  |  |  |
|  | 24991 | Secondary diabetes mellitus with unspecified complication, uncontrolled |  |  |  |  |  |  |  |  |  |
|  | 25041 | Diabetes with renal manifestations, type I [juvenile type], not stated as uncontrolled |  |  |  |  |  |  |  |  |  |
|  | 25042 | Diabetes with renal manifestations, type II or unspecified type, uncontrolled |  |  |  |  |  |  |  |  |  |
|  | 25043 | Diabetes with renal manifestations, type I [juvenile type], uncontrolled |  |  |  |  |  |  |  |  |  |
|  | 25050 | Diabetes with ophthalmic manifestations, type II or unspecified type, not stated as uncontrolled |  |  |  |  |  |  |  |  |  |
|  | 25051 | Diabetes with ophthalmic manifestations, type I [juvenile type], not stated as uncontrolled |  |  |  |  |  |  |  |  |  |
|  | 25052 | Diabetes with ophthalmic manifestations, type II or unspecified type, uncontrolled |  |  |  |  |  |  |  |  |  |
|  | 25053 | Diabetes with ophthalmic manifestations, type I [juvenile type], uncontrolled |  |  |  |  |  |  |  |  |  |
|  | 25060 | Diabetes with neurological manifestations, type II or unspecified type, not stated as uncontrolled |  |  |  |  |  |  |  |  |  |
|  | 25061 | Diabetes with neurological manifestations, type I [juvenile type], not stated as uncontrolled |  |  |  |  |  |  |  |  |  |
|  | 25062 | Diabetes with neurological manifestations, type II or unspecified type, uncontrolled |  |  |  |  |  |  |  |  |  |
|  | 25063 | Diabetes with neurological manifestations, type I [juvenile type], uncontrolled |  |  |  |  |  |  |  |  |  |
|  | 25070 | Diabetes with peripheral circulatory disorders, type II or unspecified type, not stated as uncontrolled |  |  |  |  |  |  |  |  |  |
|  | 25071 | Diabetes with peripheral circulatory disorders, type I [juvenile type], not stated as uncontrolled |  |  |  |  |  |  |  |  |  |
|  | 25072 | Diabetes with peripheral circulatory disorders, type II or unspecified type, uncontrolled |  |  |  |  |  |  |  |  |  |
|  | 25073 | Diabetes with peripheral circulatory disorders, type I [juvenile type], uncontrolled |  |  |  |  |  |  |  |  |  |
|  | 25080 | Diabetes with other specified manifestations, type II or unspecified type, not stated as uncontrolled |  |  |  |  |  |  |  |  |  |
|  | 25081 | Diabetes with other specified manifestations, type I [juvenile type], not stated as uncontrolled |  |  |  |  |  |  |  |  |  |
|  | 25082 | Diabetes with other specified manifestations, type II or unspecified type, uncontrolled |  |  |  |  |  |  |  |  |  |
|  | 25083 | Diabetes with other specified manifestations, type I [juvenile type], uncontrolled |  |  |  |  |  |  |  |  |  |
|  | 25090 | Diabetes with unspecified complication, type II or unspecified type, not stated as uncontrolled |  |  |  |  |  |  |  |  |  |
|  | 25091 | Diabetes with unspecified complication, type I [juvenile type], not stated as uncontrolled |  |  |  |  |  |  |  |  |  |
|  | 25092 | Diabetes with unspecified complication, type II or unspecified type, uncontrolled |  |  |  |  |  |  |  |  |  |
|  | 25093 | Diabetes with unspecified complication, type I [juvenile type], uncontrolled |  |  |  |  |  |  |  |  |  |
|  | E0801 | Diabetes mellitus due to underlying condition with hyperosmolarity with coma |  |  |  |  |  |  |  |  |  |
|  | E0810 | Diabetes mellitus due to underlying condition with ketoacidosis without coma |  |  |  |  |  |  |  |  |  |
|  | E0811 | Diabetes mellitus due to underlying condition with ketoacidosis with coma |  |  |  |  |  |  |  |  |  |
|  | E0821 | Diabetes mellitus due to underlying condition with diabetic nephropathy |  |  |  |  |  |  |  |  |  |
|  | E0822 | Diabetes mellitus due to underlying condition with diabetic chronic kidney disease |  |  |  |  |  |  |  |  |  |
|  | E0829 | Diabetes mellitus due to underlying condition with other diabetic kidney complication |  |  |  |  |  |  |  |  |  |
|  | E08311 | Diabetes mellitus due to underlying condition with unspecified diabetic retinopathy with macular edema |  |  |  |  |  |  |  |  |  |
|  | E08319 | Diabetes mellitus due to underlying condition with unspecified diabetic retinopathy without macular edema |  |  |  |  |  |  |  |  |  |
|  | E083211 | Diabetes mellitus due to underlying condition with mild nonproliferative diabetic retinopathy with macular edema, right eye |  |  |  |  |  |  |  |  |  |
|  | E083212 | Diabetes mellitus due to underlying condition with mild nonproliferative diabetic retinopathy with macular edema, left eye |  |  |  |  |  |  |  |  |  |
|  | E083213 | Diabetes mellitus due to underlying condition with mild nonproliferative diabetic retinopathy with macular edema, bilateral |  |  |  |  |  |  |  |  |  |
|  | E083219 | Diabetes mellitus due to underlying condition with mild nonproliferative diabetic retinopathy with macular edema, unspecified eye |  |  |  |  |  |  |  |  |  |
|  | E083291 | Diabetes mellitus due to underlying condition with mild nonproliferative diabetic retinopathy without macular edema, right eye |  |  |  |  |  |  |  |  |  |
|  | E083292 | Diabetes mellitus due to underlying condition with mild nonproliferative diabetic retinopathy without macular edema, left eye |  |  |  |  |  |  |  |  |  |
|  | E083293 | Diabetes mellitus due to underlying condition with mild nonproliferative diabetic retinopathy without macular edema, bilateral |  |  |  |  |  |  |  |  |  |
|  | E083299 | Diabetes mellitus due to underlying condition with mild nonproliferative diabetic retinopathy without macular edema, unspecified eye |  |  |  |  |  |  |  |  |  |
|  | E083311 | Diabetes mellitus due to underlying condition with moderate nonproliferative diabetic retinopathy with macular edema, right eye |  |  |  |  |  |  |  |  |  |
|  | E083312 | Diabetes mellitus due to underlying condition with moderate nonproliferative diabetic retinopathy with macular edema, left eye |  |  |  |  |  |  |  |  |  |
|  | E083313 | Diabetes mellitus due to underlying condition with moderate nonproliferative diabetic retinopathy with macular edema, bilateral |  |  |  |  |  |  |  |  |  |
|  | E083319 | Diabetes mellitus due to underlying condition with moderate nonproliferative diabetic retinopathy with macular edema, unspecified eye |  |  |  |  |  |  |  |  |  |
|  | E083391 | Diabetes mellitus due to underlying condition with moderate nonproliferative diabetic retinopathy without macular edema, right eye |  |  |  |  |  |  |  |  |  |
|  | E083392 | Diabetes mellitus due to underlying condition with moderate nonproliferative diabetic retinopathy without macular edema, left eye |  |  |  |  |  |  |  |  |  |
|  | E083393 | Diabetes mellitus due to underlying condition with moderate nonproliferative diabetic retinopathy without macular edema, bilateral |  |  |  |  |  |  |  |  |  |
|  | E083399 | Diabetes mellitus due to underlying condition with moderate nonproliferative diabetic retinopathy without macular edema, unspecified eye |  |  |  |  |  |  |  |  |  |
|  | E083411 | Diabetes mellitus due to underlying condition with severe nonproliferative diabetic retinopathy with macular edema, right eye |  |  |  |  |  |  |  |  |  |
|  | E083412 | Diabetes mellitus due to underlying condition with severe nonproliferative diabetic retinopathy with macular edema, left eye |  |  |  |  |  |  |  |  |  |
|  | E083413 | Diabetes mellitus due to underlying condition with severe nonproliferative diabetic retinopathy with macular edema, bilateral |  |  |  |  |  |  |  |  |  |
|  | E083419 | Diabetes mellitus due to underlying condition with severe nonproliferative diabetic retinopathy with macular edema, unspecified eye |  |  |  |  |  |  |  |  |  |
|  | E083491 | Diabetes mellitus due to underlying condition with severe nonproliferative diabetic retinopathy without macular edema, right eye |  |  |  |  |  |  |  |  |  |
|  | E083492 | Diabetes mellitus due to underlying condition with severe nonproliferative diabetic retinopathy without macular edema, left eye |  |  |  |  |  |  |  |  |  |
|  | E083493 | Diabetes mellitus due to underlying condition with severe nonproliferative diabetic retinopathy without macular edema, bilateral |  |  |  |  |  |  |  |  |  |
|  | E109 | Type 1 diabetes mellitus without complications |  |  |  |  |  |  |  |  |  |
|  | E083499 | Diabetes mellitus due to underlying condition with severe nonproliferative diabetic retinopathy without macular edema, unspecified eye |  |  |  |  |  |  |  |  |  |
|  | E083511 | Diabetes mellitus due to underlying condition with proliferative diabetic retinopathy with macular edema, right eye |  |  |  |  |  |  |  |  |  |
|  | E083512 | Diabetes mellitus due to underlying condition with proliferative diabetic retinopathy with macular edema, left eye |  |  |  |  |  |  |  |  |  |
|  | E083513 | Diabetes mellitus due to underlying condition with proliferative diabetic retinopathy with macular edema, bilateral |  |  |  |  |  |  |  |  |  |
|  | E083519 | Diabetes mellitus due to underlying condition with proliferative diabetic retinopathy with macular edema, unspecified eye |  |  |  |  |  |  |  |  |  |
|  | E083521 | Diabetes mellitus due to underlying condition with proliferative diabetic retinopathy with traction retinal detachment involving the macula, right eye |  |  |  |  |  |  |  |  |  |
|  | E083522 | Diabetes mellitus due to underlying condition with proliferative diabetic retinopathy with traction retinal detachment involving the macula, left eye |  |  |  |  |  |  |  |  |  |
|  | E083523 | Diabetes mellitus due to underlying condition with proliferative diabetic retinopathy with traction retinal detachment involving the macula, bilateral |  |  |  |  |  |  |  |  |  |
|  | E083529 | Diabetes mellitus due to underlying condition with proliferative diabetic retinopathy with traction retinal detachment involving the macula, unspecified eye |  |  |  |  |  |  |  |  |  |
|  | E083531 | Diabetes mellitus due to underlying condition with proliferative diabetic retinopathy with traction retinal detachment not involving the macula, right eye |  |  |  |  |  |  |  |  |  |
|  | E083532 | Diabetes mellitus due to underlying condition with proliferative diabetic retinopathy with traction retinal detachment not involving the macula, left eye |  |  |  |  |  |  |  |  |  |
|  | E083533 | Diabetes mellitus due to underlying condition with proliferative diabetic retinopathy with traction retinal detachment not involving the macula, bilateral |  |  |  |  |  |  |  |  |  |
|  | E083539 | Diabetes mellitus due to underlying condition with proliferative diabetic retinopathy with traction retinal detachment not involving the macula, unspecified eye |  |  |  |  |  |  |  |  |  |
|  | E083541 | Diabetes mellitus due to underlying condition with proliferative diabetic retinopathy with combined traction retinal detachment and rhegmatogenous retinal detachment, right eye |  |  |  |  |  |  |  |  |  |
|  | E083542 | Diabetes mellitus due to underlying condition with proliferative diabetic retinopathy with combined traction retinal detachment and rhegmatogenous retinal detachment, left eye |  |  |  |  |  |  |  |  |  |
|  | E083543 | Diabetes mellitus due to underlying condition with proliferative diabetic retinopathy with combined traction retinal detachment and rhegmatogenous retinal detachment, bilateral |  |  |  |  |  |  |  |  |  |
|  | E083549 | Diabetes mellitus due to underlying condition with proliferative diabetic retinopathy with combined traction retinal detachment and rhegmatogenous retinal detachment, unspecified eye |  |  |  |  |  |  |  |  |  |
|  | E083551 | Diabetes mellitus due to underlying condition with stable proliferative diabetic retinopathy, right eye |  |  |  |  |  |  |  |  |  |
|  | E083552 | Diabetes mellitus due to underlying condition with stable proliferative diabetic retinopathy, left eye |  |  |  |  |  |  |  |  |  |
|  | E083553 | Diabetes mellitus due to underlying condition with stable proliferative diabetic retinopathy, bilateral |  |  |  |  |  |  |  |  |  |
|  | E083559 | Diabetes mellitus due to underlying condition with stable proliferative diabetic retinopathy, unspecified eye |  |  |  |  |  |  |  |  |  |
|  | E083591 | Diabetes mellitus due to underlying condition with proliferative diabetic retinopathy without macular edema, right eye |  |  |  |  |  |  |  |  |  |
|  | E083592 | Diabetes mellitus due to underlying condition with proliferative diabetic retinopathy without macular edema, left eye |  |  |  |  |  |  |  |  |  |
|  | E083593 | Diabetes mellitus due to underlying condition with proliferative diabetic retinopathy without macular edema, bilateral |  |  |  |  |  |  |  |  |  |
|  | E083599 | Diabetes mellitus due to underlying condition with proliferative diabetic retinopathy without macular edema, unspecified eye |  |  |  |  |  |  |  |  |  |
|  | E0836 | Diabetes mellitus due to underlying condition with diabetic cataract |  |  |  |  |  |  |  |  |  |
|  | E0837X1 | Diabetes mellitus due to underlying condition with diabetic macular edema, resolved following treatment, right eye |  |  |  |  |  |  |  |  |  |
|  | E0837X2 | Diabetes mellitus due to underlying condition with diabetic macular edema, resolved following treatment, left eye |  |  |  |  |  |  |  |  |  |
|  | E0837X3 | Diabetes mellitus due to underlying condition with diabetic macular edema, resolved following treatment, bilateral |  |  |  |  |  |  |  |  |  |
|  | E0837X9 | Diabetes mellitus due to underlying condition with diabetic macular edema, resolved following treatment, unspecified eye |  |  |  |  |  |  |  |  |  |
|  | E0839 | Diabetes mellitus due to underlying condition with other diabetic ophthalmic complication |  |  |  |  |  |  |  |  |  |
|  | E0840 | Diabetes mellitus due to underlying condition with diabetic neuropathy, unspecified |  |  |  |  |  |  |  |  |  |
|  | E0841 | Diabetes mellitus due to underlying condition with diabetic mononeuropathy |  |  |  |  |  |  |  |  |  |
|  | E0842 | Diabetes mellitus due to underlying condition with diabetic polyneuropathy |  |  |  |  |  |  |  |  |  |
|  | E0843 | Diabetes mellitus due to underlying condition with diabetic autonomic (poly)neuropathy |  |  |  |  |  |  |  |  |  |
|  | E0844 | Diabetes mellitus due to underlying condition with diabetic amyotrophy |  |  |  |  |  |  |  |  |  |
|  | E0849 | Diabetes mellitus due to underlying condition with other diabetic neurological complication |  |  |  |  |  |  |  |  |  |
|  | E0851 | Diabetes mellitus due to underlying condition with diabetic peripheral angiopathy without gangrene |  |  |  |  |  |  |  |  |  |
|  | E0852 | Diabetes mellitus due to underlying condition with diabetic peripheral angiopathy with gangrene |  |  |  |  |  |  |  |  |  |
|  | E0859 | Diabetes mellitus due to underlying condition with other circulatory complications |  |  |  |  |  |  |  |  |  |
|  | E08610 | Diabetes mellitus due to underlying condition with diabetic neuropathic arthropathy |  |  |  |  |  |  |  |  |  |
|  | E08618 | Diabetes mellitus due to underlying condition with other diabetic arthropathy |  |  |  |  |  |  |  |  |  |
|  | E08620 | Diabetes mellitus due to underlying condition with diabetic dermatitis |  |  |  |  |  |  |  |  |  |
|  | E08621 | Diabetes mellitus due to underlying condition with foot ulcer |  |  |  |  |  |  |  |  |  |
|  | E08622 | Diabetes mellitus due to underlying condition with other skin ulcer |  |  |  |  |  |  |  |  |  |
|  | E08628 | Diabetes mellitus due to underlying condition with other skin complications |  |  |  |  |  |  |  |  |  |
|  | E08630 | Diabetes mellitus due to underlying condition with periodontal disease |  |  |  |  |  |  |  |  |  |
|  | E08638 | Diabetes mellitus due to underlying condition with other oral complications |  |  |  |  |  |  |  |  |  |
|  | E08641 | Diabetes mellitus due to underlying condition with hypoglycemia with coma |  |  |  |  |  |  |  |  |  |
|  | E08649 | Diabetes mellitus due to underlying condition with hypoglycemia without coma |  |  |  |  |  |  |  |  |  |
|  | E0865 | Diabetes mellitus due to underlying condition with hyperglycemia |  |  |  |  |  |  |  |  |  |
|  | E0869 | Diabetes mellitus due to underlying condition with other specified complication |  |  |  |  |  |  |  |  |  |
|  | E088 | Diabetes mellitus due to underlying condition with unspecified complications |  |  |  |  |  |  |  |  |  |
|  | E089 | Diabetes mellitus due to underlying condition without complications |  |  |  |  |  |  |  |  |  |
|  | E0900 | Drug or chemical induced diabetes mellitus with hyperosmolarity without nonketotic hyperglycemic-hyperosmolar coma (NKHHC) |  |  |  |  |  |  |  |  |  |
|  | E0901 | Drug or chemical induced diabetes mellitus with hyperosmolarity with coma |  |  |  |  |  |  |  |  |  |
|  | E0910 | Drug or chemical induced diabetes mellitus with ketoacidosis without coma |  |  |  |  |  |  |  |  |  |
|  | E0911 | Drug or chemical induced diabetes mellitus with ketoacidosis with coma |  |  |  |  |  |  |  |  |  |
|  | E0921 | Drug or chemical induced diabetes mellitus with diabetic nephropathy |  |  |  |  |  |  |  |  |  |
|  | E0922 | Drug or chemical induced diabetes mellitus with diabetic chronic kidney disease |  |  |  |  |  |  |  |  |  |
|  | E0929 | Drug or chemical induced diabetes mellitus with other diabetic kidney complication |  |  |  |  |  |  |  |  |  |
|  | E09311 | Drug or chemical induced diabetes mellitus with unspecified diabetic retinopathy with macular edema |  |  |  |  |  |  |  |  |  |
|  | E09319 | Drug or chemical induced diabetes mellitus with unspecified diabetic retinopathy without macular edema |  |  |  |  |  |  |  |  |  |
|  | E093211 | Drug or chemical induced diabetes mellitus with mild nonproliferative diabetic retinopathy with macular edema, right eye |  |  |  |  |  |  |  |  |  |
|  | E093212 | Drug or chemical induced diabetes mellitus with mild nonproliferative diabetic retinopathy with macular edema, left eye |  |  |  |  |  |  |  |  |  |
|  | E093213 | Drug or chemical induced diabetes mellitus with mild nonproliferative diabetic retinopathy with macular edema, bilateral |  |  |  |  |  |  |  |  |  |
|  | E093219 | Drug or chemical induced diabetes mellitus with mild nonproliferative diabetic retinopathy with macular edema, unspecified eye |  |  |  |  |  |  |  |  |  |
|  | E093291 | Drug or chemical induced diabetes mellitus with mild nonproliferative diabetic retinopathy without macular edema, right eye |  |  |  |  |  |  |  |  |  |
|  | E093292 | Drug or chemical induced diabetes mellitus with mild nonproliferative diabetic retinopathy without macular edema, left eye |  |  |  |  |  |  |  |  |  |
|  | E093293 | Drug or chemical induced diabetes mellitus with mild nonproliferative diabetic retinopathy without macular edema, bilateral |  |  |  |  |  |  |  |  |  |
|  | E093299 | Drug or chemical induced diabetes mellitus with mild nonproliferative diabetic retinopathy without macular edema, unspecified eye |  |  |  |  |  |  |  |  |  |
|  | E093311 | Drug or chemical induced diabetes mellitus with moderate nonproliferative diabetic retinopathy with macular edema, right eye |  |  |  |  |  |  |  |  |  |
|  | E093312 | Drug or chemical induced diabetes mellitus with moderate nonproliferative diabetic retinopathy with macular edema, left eye |  |  |  |  |  |  |  |  |  |
|  | E093313 | Drug or chemical induced diabetes mellitus with moderate nonproliferative diabetic retinopathy with macular edema, bilateral |  |  |  |  |  |  |  |  |  |
|  | E093319 | Drug or chemical induced diabetes mellitus with moderate nonproliferative diabetic retinopathy with macular edema, unspecified eye |  |  |  |  |  |  |  |  |  |
|  | E093391 | Drug or chemical induced diabetes mellitus with moderate nonproliferative diabetic retinopathy without macular edema, right eye |  |  |  |  |  |  |  |  |  |
|  | E093392 | Drug or chemical induced diabetes mellitus with moderate nonproliferative diabetic retinopathy without macular edema, left eye |  |  |  |  |  |  |  |  |  |
|  | E093393 | Drug or chemical induced diabetes mellitus with moderate nonproliferative diabetic retinopathy without macular edema, bilateral |  |  |  |  |  |  |  |  |  |
|  | E093399 | Drug or chemical induced diabetes mellitus with moderate nonproliferative diabetic retinopathy without macular edema, unspecified eye |  |  |  |  |  |  |  |  |  |
|  | E093411 | Drug or chemical induced diabetes mellitus with severe nonproliferative diabetic retinopathy with macular edema, right eye |  |  |  |  |  |  |  |  |  |
|  | E093412 | Drug or chemical induced diabetes mellitus with severe nonproliferative diabetic retinopathy with macular edema, left eye |  |  |  |  |  |  |  |  |  |
|  | E093413 | Drug or chemical induced diabetes mellitus with severe nonproliferative diabetic retinopathy with macular edema, bilateral |  |  |  |  |  |  |  |  |  |
|  | E093419 | Drug or chemical induced diabetes mellitus with severe nonproliferative diabetic retinopathy with macular edema, unspecified eye |  |  |  |  |  |  |  |  |  |
|  | E093491 | Drug or chemical induced diabetes mellitus with severe nonproliferative diabetic retinopathy without macular edema, right eye |  |  |  |  |  |  |  |  |  |
|  | E093492 | Drug or chemical induced diabetes mellitus with severe nonproliferative diabetic retinopathy without macular edema, left eye |  |  |  |  |  |  |  |  |  |
|  | E093493 | Drug or chemical induced diabetes mellitus with severe nonproliferative diabetic retinopathy without macular edema, bilateral |  |  |  |  |  |  |  |  |  |
|  | E093499 | Drug or chemical induced diabetes mellitus with severe nonproliferative diabetic retinopathy without macular edema, unspecified eye |  |  |  |  |  |  |  |  |  |
|  | E093511 | Drug or chemical induced diabetes mellitus with proliferative diabetic retinopathy with macular edema, right eye |  |  |  |  |  |  |  |  |  |
|  | E093512 | Drug or chemical induced diabetes mellitus with proliferative diabetic retinopathy with macular edema, left eye |  |  |  |  |  |  |  |  |  |
|  | E093513 | Drug or chemical induced diabetes mellitus with proliferative diabetic retinopathy with macular edema, bilateral |  |  |  |  |  |  |  |  |  |
|  | E093519 | Drug or chemical induced diabetes mellitus with proliferative diabetic retinopathy with macular edema, unspecified eye |  |  |  |  |  |  |  |  |  |
|  | E093521 | Drug or chemical induced diabetes mellitus with proliferative diabetic retinopathy with traction retinal detachment involving the macula, right eye |  |  |  |  |  |  |  |  |  |
|  | E093522 | Drug or chemical induced diabetes mellitus with proliferative diabetic retinopathy with traction retinal detachment involving the macula, left eye |  |  |  |  |  |  |  |  |  |
|  | E093523 | Drug or chemical induced diabetes mellitus with proliferative diabetic retinopathy with traction retinal detachment involving the macula, bilateral |  |  |  |  |  |  |  |  |  |
|  | E093529 | Drug or chemical induced diabetes mellitus with proliferative diabetic retinopathy with traction retinal detachment involving the macula, unspecified eye |  |  |  |  |  |  |  |  |  |
|  | E093531 | Drug or chemical induced diabetes mellitus with proliferative diabetic retinopathy with traction retinal detachment not involving the macula, right eye |  |  |  |  |  |  |  |  |  |
|  | E093532 | Drug or chemical induced diabetes mellitus with proliferative diabetic retinopathy with traction retinal detachment not involving the macula, left eye |  |  |  |  |  |  |  |  |  |
|  | E093533 | Drug or chemical induced diabetes mellitus with proliferative diabetic retinopathy with traction retinal detachment not involving the macula, bilateral |  |  |  |  |  |  |  |  |  |
|  | E093539 | Drug or chemical induced diabetes mellitus with proliferative diabetic retinopathy with traction retinal detachment not involving the macula, unspecified eye |  |  |  |  |  |  |  |  |  |
|  | E093541 | Drug or chemical induced diabetes mellitus with proliferative diabetic retinopathy with combined traction retinal detachment and rhegmatogenous retinal detachment, right eye |  |  |  |  |  |  |  |  |  |
|  | E093542 | Drug or chemical induced diabetes mellitus with proliferative diabetic retinopathy with combined traction retinal detachment and rhegmatogenous retinal detachment, left eye |  |  |  |  |  |  |  |  |  |
|  | E093543 | Drug or chemical induced diabetes mellitus with proliferative diabetic retinopathy with combined traction retinal detachment and rhegmatogenous retinal detachment, bilateral |  |  |  |  |  |  |  |  |  |
|  | E208 | Other hypoparathyroidism |  |  |  |  |  |  |  |  |  |
|  | E093549 | Drug or chemical induced diabetes mellitus with proliferative diabetic retinopathy with combined traction retinal detachment and rhegmatogenous retinal detachment, unspecified eye |  |  |  |  |  |  |  |  |  |
|  | E093551 | Drug or chemical induced diabetes mellitus with stable proliferative diabetic retinopathy, right eye |  |  |  |  |  |  |  |  |  |
|  | E093552 | Drug or chemical induced diabetes mellitus with stable proliferative diabetic retinopathy, left eye |  |  |  |  |  |  |  |  |  |
|  | E093553 | Drug or chemical induced diabetes mellitus with stable proliferative diabetic retinopathy, bilateral |  |  |  |  |  |  |  |  |  |
|  | E093559 | Drug or chemical induced diabetes mellitus with stable proliferative diabetic retinopathy, unspecified eye |  |  |  |  |  |  |  |  |  |
|  | E093591 | Drug or chemical induced diabetes mellitus with proliferative diabetic retinopathy without macular edema, right eye |  |  |  |  |  |  |  |  |  |
|  | E093592 | Drug or chemical induced diabetes mellitus with proliferative diabetic retinopathy without macular edema, left eye |  |  |  |  |  |  |  |  |  |
|  | E093593 | Drug or chemical induced diabetes mellitus with proliferative diabetic retinopathy without macular edema, bilateral |  |  |  |  |  |  |  |  |  |
|  | E093599 | Drug or chemical induced diabetes mellitus with proliferative diabetic retinopathy without macular edema, unspecified eye |  |  |  |  |  |  |  |  |  |
|  | E0936 | Drug or chemical induced diabetes mellitus with diabetic cataract |  |  |  |  |  |  |  |  |  |
|  | E0937X1 | Drug or chemical induced diabetes mellitus with diabetic macular edema, resolved following treatment, right eye |  |  |  |  |  |  |  |  |  |
|  | E0937X2 | Drug or chemical induced diabetes mellitus with diabetic macular edema, resolved following treatment, left eye |  |  |  |  |  |  |  |  |  |
|  | E0937X3 | Drug or chemical induced diabetes mellitus with diabetic macular edema, resolved following treatment, bilateral |  |  |  |  |  |  |  |  |  |
|  | E0937X9 | Drug or chemical induced diabetes mellitus with diabetic macular edema, resolved following treatment, unspecified eye |  |  |  |  |  |  |  |  |  |
|  | E0939 | Drug or chemical induced diabetes mellitus with other diabetic ophthalmic complication |  |  |  |  |  |  |  |  |  |
|  | E0940 | Drug or chemical induced diabetes mellitus with neurological complications with diabetic neuropathy, unspecified |  |  |  |  |  |  |  |  |  |
|  | E0941 | Drug or chemical induced diabetes mellitus with neurological complications with diabetic mononeuropathy |  |  |  |  |  |  |  |  |  |
|  | E0942 | Drug or chemical induced diabetes mellitus with neurological complications with diabetic polyneuropathy |  |  |  |  |  |  |  |  |  |
|  | E0943 | Drug or chemical induced diabetes mellitus with neurological complications with diabetic autonomic (poly)neuropathy |  |  |  |  |  |  |  |  |  |
|  | E0944 | Drug or chemical induced diabetes mellitus with neurological complications with diabetic amyotrophy |  |  |  |  |  |  |  |  |  |
|  | E0949 | Drug or chemical induced diabetes mellitus with neurological complications with other diabetic neurological complication |  |  |  |  |  |  |  |  |  |
|  | E0951 | Drug or chemical induced diabetes mellitus with diabetic peripheral angiopathy without gangrene |  |  |  |  |  |  |  |  |  |
|  | E0952 | Drug or chemical induced diabetes mellitus with diabetic peripheral angiopathy with gangrene |  |  |  |  |  |  |  |  |  |
|  | E0959 | Drug or chemical induced diabetes mellitus with other circulatory complications |  |  |  |  |  |  |  |  |  |
|  | E09610 | Drug or chemical induced diabetes mellitus with diabetic neuropathic arthropathy |  |  |  |  |  |  |  |  |  |
|  | E09618 | Drug or chemical induced diabetes mellitus with other diabetic arthropathy |  |  |  |  |  |  |  |  |  |
|  | E09620 | Drug or chemical induced diabetes mellitus with diabetic dermatitis |  |  |  |  |  |  |  |  |  |
|  | E09621 | Drug or chemical induced diabetes mellitus with foot ulcer |  |  |  |  |  |  |  |  |  |
|  | E09622 | Drug or chemical induced diabetes mellitus with other skin ulcer |  |  |  |  |  |  |  |  |  |
|  | E09628 | Drug or chemical induced diabetes mellitus with other skin complications |  |  |  |  |  |  |  |  |  |
|  | E09630 | Drug or chemical induced diabetes mellitus with periodontal disease |  |  |  |  |  |  |  |  |  |
|  | E09638 | Drug or chemical induced diabetes mellitus with other oral complications |  |  |  |  |  |  |  |  |  |
|  | E09641 | Drug or chemical induced diabetes mellitus with hypoglycemia with coma |  |  |  |  |  |  |  |  |  |
|  | E09649 | Drug or chemical induced diabetes mellitus with hypoglycemia without coma |  |  |  |  |  |  |  |  |  |
|  | E0965 | Drug or chemical induced diabetes mellitus with hyperglycemia |  |  |  |  |  |  |  |  |  |
|  | E0969 | Drug or chemical induced diabetes mellitus with other specified complication |  |  |  |  |  |  |  |  |  |
|  | E098 | Drug or chemical induced diabetes mellitus with unspecified complications |  |  |  |  |  |  |  |  |  |
|  | E099 | Drug or chemical induced diabetes mellitus without complications |  |  |  |  |  |  |  |  |  |
|  | E1010 | Type 1 diabetes mellitus with ketoacidosis without coma |  |  |  |  |  |  |  |  |  |
|  | E1011 | Type 1 diabetes mellitus with ketoacidosis with coma |  |  |  |  |  |  |  |  |  |
|  | E1021 | Type 1 diabetes mellitus with diabetic nephropathy |  |  |  |  |  |  |  |  |  |
|  | E1022 | Type 1 diabetes mellitus with diabetic chronic kidney disease |  |  |  |  |  |  |  |  |  |
|  | E1029 | Type 1 diabetes mellitus with other diabetic kidney complication |  |  |  |  |  |  |  |  |  |
|  | E10311 | Type 1 diabetes mellitus with unspecified diabetic retinopathy with macular edema |  |  |  |  |  |  |  |  |  |
|  | E10319 | Type 1 diabetes mellitus with unspecified diabetic retinopathy without macular edema |  |  |  |  |  |  |  |  |  |
|  | E103211 | Type 1 diabetes mellitus with mild nonproliferative diabetic retinopathy with macular edema, right eye |  |  |  |  |  |  |  |  |  |
|  | E103212 | Type 1 diabetes mellitus with mild nonproliferative diabetic retinopathy with macular edema, left eye |  |  |  |  |  |  |  |  |  |
|  | E103213 | Type 1 diabetes mellitus with mild nonproliferative diabetic retinopathy with macular edema, bilateral |  |  |  |  |  |  |  |  |  |
|  | E103219 | Type 1 diabetes mellitus with mild nonproliferative diabetic retinopathy with macular edema, unspecified eye |  |  |  |  |  |  |  |  |  |
|  | E103291 | Type 1 diabetes mellitus with mild nonproliferative diabetic retinopathy without macular edema, right eye |  |  |  |  |  |  |  |  |  |
|  | E103292 | Type 1 diabetes mellitus with mild nonproliferative diabetic retinopathy without macular edema, left eye |  |  |  |  |  |  |  |  |  |
|  | E103293 | Type 1 diabetes mellitus with mild nonproliferative diabetic retinopathy without macular edema, bilateral |  |  |  |  |  |  |  |  |  |
|  | E103299 | Type 1 diabetes mellitus with mild nonproliferative diabetic retinopathy without macular edema, unspecified eye |  |  |  |  |  |  |  |  |  |
|  | E103311 | Type 1 diabetes mellitus with moderate nonproliferative diabetic retinopathy with macular edema, right eye |  |  |  |  |  |  |  |  |  |
|  | E103312 | Type 1 diabetes mellitus with moderate nonproliferative diabetic retinopathy with macular edema, left eye |  |  |  |  |  |  |  |  |  |
|  | E103313 | Type 1 diabetes mellitus with moderate nonproliferative diabetic retinopathy with macular edema, bilateral |  |  |  |  |  |  |  |  |  |
|  | E103319 | Type 1 diabetes mellitus with moderate nonproliferative diabetic retinopathy with macular edema, unspecified eye |  |  |  |  |  |  |  |  |  |
|  | E103391 | Type 1 diabetes mellitus with moderate nonproliferative diabetic retinopathy without macular edema, right eye |  |  |  |  |  |  |  |  |  |
|  | E103392 | Type 1 diabetes mellitus with moderate nonproliferative diabetic retinopathy without macular edema, left eye |  |  |  |  |  |  |  |  |  |
|  | E103393 | Type 1 diabetes mellitus with moderate nonproliferative diabetic retinopathy without macular edema, bilateral |  |  |  |  |  |  |  |  |  |
|  | E103399 | Type 1 diabetes mellitus with moderate nonproliferative diabetic retinopathy without macular edema, unspecified eye |  |  |  |  |  |  |  |  |  |
|  | E103411 | Type 1 diabetes mellitus with severe nonproliferative diabetic retinopathy with macular edema, right eye |  |  |  |  |  |  |  |  |  |
|  | E103412 | Type 1 diabetes mellitus with severe nonproliferative diabetic retinopathy with macular edema, left eye |  |  |  |  |  |  |  |  |  |
|  | E103413 | Type 1 diabetes mellitus with severe nonproliferative diabetic retinopathy with macular edema, bilateral |  |  |  |  |  |  |  |  |  |
|  | E103419 | Type 1 diabetes mellitus with severe nonproliferative diabetic retinopathy with macular edema, unspecified eye |  |  |  |  |  |  |  |  |  |
|  | E103491 | Type 1 diabetes mellitus with severe nonproliferative diabetic retinopathy without macular edema, right eye |  |  |  |  |  |  |  |  |  |
|  | E103492 | Type 1 diabetes mellitus with severe nonproliferative diabetic retinopathy without macular edema, left eye |  |  |  |  |  |  |  |  |  |
|  | E103493 | Type 1 diabetes mellitus with severe nonproliferative diabetic retinopathy without macular edema, bilateral |  |  |  |  |  |  |  |  |  |
|  | E103499 | Type 1 diabetes mellitus with severe nonproliferative diabetic retinopathy without macular edema, unspecified eye |  |  |  |  |  |  |  |  |  |
|  | E103511 | Type 1 diabetes mellitus with proliferative diabetic retinopathy with macular edema, right eye |  |  |  |  |  |  |  |  |  |
|  | E103512 | Type 1 diabetes mellitus with proliferative diabetic retinopathy with macular edema, left eye |  |  |  |  |  |  |  |  |  |
|  | E103513 | Type 1 diabetes mellitus with proliferative diabetic retinopathy with macular edema, bilateral |  |  |  |  |  |  |  |  |  |
|  | E103519 | Type 1 diabetes mellitus with proliferative diabetic retinopathy with macular edema, unspecified eye |  |  |  |  |  |  |  |  |  |
|  | E103521 | Type 1 diabetes mellitus with proliferative diabetic retinopathy with traction retinal detachment involving the macula, right eye |  |  |  |  |  |  |  |  |  |
|  | E103522 | Type 1 diabetes mellitus with proliferative diabetic retinopathy with traction retinal detachment involving the macula, left eye |  |  |  |  |  |  |  |  |  |
|  | E103523 | Type 1 diabetes mellitus with proliferative diabetic retinopathy with traction retinal detachment involving the macula, bilateral |  |  |  |  |  |  |  |  |  |
|  | E103529 | Type 1 diabetes mellitus with proliferative diabetic retinopathy with traction retinal detachment involving the macula, unspecified eye |  |  |  |  |  |  |  |  |  |
|  | E103531 | Type 1 diabetes mellitus with proliferative diabetic retinopathy with traction retinal detachment not involving the macula, right eye |  |  |  |  |  |  |  |  |  |
|  | E103532 | Type 1 diabetes mellitus with proliferative diabetic retinopathy with traction retinal detachment not involving the macula, left eye |  |  |  |  |  |  |  |  |  |
|  | E103533 | Type 1 diabetes mellitus with proliferative diabetic retinopathy with traction retinal detachment not involving the macula, bilateral |  |  |  |  |  |  |  |  |  |
|  | E103539 | Type 1 diabetes mellitus with proliferative diabetic retinopathy with traction retinal detachment not involving the macula, unspecified eye |  |  |  |  |  |  |  |  |  |
|  | E103541 | Type 1 diabetes mellitus with proliferative diabetic retinopathy with combined traction retinal detachment and rhegmatogenous retinal detachment, right eye |  |  |  |  |  |  |  |  |  |
|  | E103542 | Type 1 diabetes mellitus with proliferative diabetic retinopathy with combined traction retinal detachment and rhegmatogenous retinal detachment, left eye |  |  |  |  |  |  |  |  |  |
|  | E103543 | Type 1 diabetes mellitus with proliferative diabetic retinopathy with combined traction retinal detachment and rhegmatogenous retinal detachment, bilateral |  |  |  |  |  |  |  |  |  |
|  | E103549 | Type 1 diabetes mellitus with proliferative diabetic retinopathy with combined traction retinal detachment and rhegmatogenous retinal detachment, unspecified eye |  |  |  |  |  |  |  |  |  |
|  | E103551 | Type 1 diabetes mellitus with stable proliferative diabetic retinopathy, right eye |  |  |  |  |  |  |  |  |  |
|  | E103552 | Type 1 diabetes mellitus with stable proliferative diabetic retinopathy, left eye |  |  |  |  |  |  |  |  |  |
|  | E103553 | Type 1 diabetes mellitus with stable proliferative diabetic retinopathy, bilateral |  |  |  |  |  |  |  |  |  |
|  | E103559 | Type 1 diabetes mellitus with stable proliferative diabetic retinopathy, unspecified eye |  |  |  |  |  |  |  |  |  |
|  | E103591 | Type 1 diabetes mellitus with proliferative diabetic retinopathy without macular edema, right eye |  |  |  |  |  |  |  |  |  |
|  | E103592 | Type 1 diabetes mellitus with proliferative diabetic retinopathy without macular edema, left eye |  |  |  |  |  |  |  |  |  |
|  | E103593 | Type 1 diabetes mellitus with proliferative diabetic retinopathy without macular edema, bilateral |  |  |  |  |  |  |  |  |  |
|  | E103599 | Type 1 diabetes mellitus with proliferative diabetic retinopathy without macular edema, unspecified eye |  |  |  |  |  |  |  |  |  |
|  | E1036 | Type 1 diabetes mellitus with diabetic cataract |  |  |  |  |  |  |  |  |  |
|  | E1037X1 | Type 1 diabetes mellitus with diabetic macular edema, resolved following treatment, right eye |  |  |  |  |  |  |  |  |  |
|  | E1037X2 | Type 1 diabetes mellitus with diabetic macular edema, resolved following treatment, left eye |  |  |  |  |  |  |  |  |  |
|  | E1037X3 | Type 1 diabetes mellitus with diabetic macular edema, resolved following treatment, bilateral |  |  |  |  |  |  |  |  |  |
|  | E1037X9 | Type 1 diabetes mellitus with diabetic macular edema, resolved following treatment, unspecified eye |  |  |  |  |  |  |  |  |  |
|  | E1039 | Type 1 diabetes mellitus with other diabetic ophthalmic complication |  |  |  |  |  |  |  |  |  |
|  | E1040 | Type 1 diabetes mellitus with diabetic neuropathy, unspecified |  |  |  |  |  |  |  |  |  |
|  | E1041 | Type 1 diabetes mellitus with diabetic mononeuropathy |  |  |  |  |  |  |  |  |  |
|  | E1042 | Type 1 diabetes mellitus with diabetic polyneuropathy |  |  |  |  |  |  |  |  |  |
|  | E1043 | Type 1 diabetes mellitus with diabetic autonomic (poly)neuropathy |  |  |  |  |  |  |  |  |  |
|  | E1044 | Type 1 diabetes mellitus with diabetic amyotrophy |  |  |  |  |  |  |  |  |  |
|  | E1049 | Type 1 diabetes mellitus with other diabetic neurological complication |  |  |  |  |  |  |  |  |  |
|  | E1051 | Type 1 diabetes mellitus with diabetic peripheral angiopathy without gangrene |  |  |  |  |  |  |  |  |  |
|  | E1052 | Type 1 diabetes mellitus with diabetic peripheral angiopathy with gangrene |  |  |  |  |  |  |  |  |  |
|  | E1059 | Type 1 diabetes mellitus with other circulatory complications |  |  |  |  |  |  |  |  |  |
|  | E10610 | Type 1 diabetes mellitus with diabetic neuropathic arthropathy |  |  |  |  |  |  |  |  |  |
|  | E10618 | Type 1 diabetes mellitus with other diabetic arthropathy |  |  |  |  |  |  |  |  |  |
|  | E10620 | Type 1 diabetes mellitus with diabetic dermatitis |  |  |  |  |  |  |  |  |  |
|  | E10621 | Type 1 diabetes mellitus with foot ulcer |  |  |  |  |  |  |  |  |  |
|  | E10622 | Type 1 diabetes mellitus with other skin ulcer |  |  |  |  |  |  |  |  |  |
|  | E10628 | Type 1 diabetes mellitus with other skin complications |  |  |  |  |  |  |  |  |  |
|  | E10630 | Type 1 diabetes mellitus with periodontal disease |  |  |  |  |  |  |  |  |  |
|  | E10638 | Type 1 diabetes mellitus with other oral complications |  |  |  |  |  |  |  |  |  |
|  | E10641 | Type 1 diabetes mellitus with hypoglycemia with coma |  |  |  |  |  |  |  |  |  |
|  | E10649 | Type 1 diabetes mellitus with hypoglycemia without coma |  |  |  |  |  |  |  |  |  |
|  | E1065 | Type 1 diabetes mellitus with hyperglycemia |  |  |  |  |  |  |  |  |  |
|  | E1069 | Type 1 diabetes mellitus with other specified complication |  |  |  |  |  |  |  |  |  |
|  | E108 | Type 1 diabetes mellitus with unspecified complications |  |  |  |  |  |  |  |  |  |
|  | E1100 | Type 2 diabetes mellitus with hyperosmolarity without nonketotic hyperglycemic-hyperosmolar coma (NKHHC) |  |  |  |  |  |  |  |  |  |
|  | E1101 | Type 2 diabetes mellitus with hyperosmolarity with coma |  |  |  |  |  |  |  |  |  |
|  | E1110 | Type 2 diabetes mellitus with ketoacidosis without coma |  |  |  |  |  |  |  |  |  |
|  | E1111 | Type 2 diabetes mellitus with ketoacidosis with coma |  |  |  |  |  |  |  |  |  |
|  | E1121 | Type 2 diabetes mellitus with diabetic nephropathy |  |  |  |  |  |  |  |  |  |
|  | E1122 | Type 2 diabetes mellitus with diabetic chronic kidney disease |  |  |  |  |  |  |  |  |  |
|  | E1129 | Type 2 diabetes mellitus with other diabetic kidney complication |  |  |  |  |  |  |  |  |  |
|  | E11311 | Type 2 diabetes mellitus with unspecified diabetic retinopathy with macular edema |  |  |  |  |  |  |  |  |  |
|  | E11319 | Type 2 diabetes mellitus with unspecified diabetic retinopathy without macular edema |  |  |  |  |  |  |  |  |  |
|  | E113211 | Type 2 diabetes mellitus with mild nonproliferative diabetic retinopathy with macular edema, right eye |  |  |  |  |  |  |  |  |  |
|  | E113212 | Type 2 diabetes mellitus with mild nonproliferative diabetic retinopathy with macular edema, left eye |  |  |  |  |  |  |  |  |  |
|  | E113213 | Type 2 diabetes mellitus with mild nonproliferative diabetic retinopathy with macular edema, bilateral |  |  |  |  |  |  |  |  |  |
|  | E113219 | Type 2 diabetes mellitus with mild nonproliferative diabetic retinopathy with macular edema, unspecified eye |  |  |  |  |  |  |  |  |  |
|  | E113291 | Type 2 diabetes mellitus with mild nonproliferative diabetic retinopathy without macular edema, right eye |  |  |  |  |  |  |  |  |  |
|  | E113292 | Type 2 diabetes mellitus with mild nonproliferative diabetic retinopathy without macular edema, left eye |  |  |  |  |  |  |  |  |  |
|  | E113293 | Type 2 diabetes mellitus with mild nonproliferative diabetic retinopathy without macular edema, bilateral |  |  |  |  |  |  |  |  |  |
|  | E113299 | Type 2 diabetes mellitus with mild nonproliferative diabetic retinopathy without macular edema, unspecified eye |  |  |  |  |  |  |  |  |  |
|  | E113311 | Type 2 diabetes mellitus with moderate nonproliferative diabetic retinopathy with macular edema, right eye |  |  |  |  |  |  |  |  |  |
|  | E113312 | Type 2 diabetes mellitus with moderate nonproliferative diabetic retinopathy with macular edema, left eye |  |  |  |  |  |  |  |  |  |
|  | E113313 | Type 2 diabetes mellitus with moderate nonproliferative diabetic retinopathy with macular edema, bilateral |  |  |  |  |  |  |  |  |  |
|  | E113319 | Type 2 diabetes mellitus with moderate nonproliferative diabetic retinopathy with macular edema, unspecified eye |  |  |  |  |  |  |  |  |  |
|  | E113391 | Type 2 diabetes mellitus with moderate nonproliferative diabetic retinopathy without macular edema, right eye |  |  |  |  |  |  |  |  |  |
|  | E113392 | Type 2 diabetes mellitus with moderate nonproliferative diabetic retinopathy without macular edema, left eye |  |  |  |  |  |  |  |  |  |
|  | E113393 | Type 2 diabetes mellitus with moderate nonproliferative diabetic retinopathy without macular edema, bilateral |  |  |  |  |  |  |  |  |  |
|  | E113399 | Type 2 diabetes mellitus with moderate nonproliferative diabetic retinopathy without macular edema, unspecified eye |  |  |  |  |  |  |  |  |  |
|  | E113411 | Type 2 diabetes mellitus with severe nonproliferative diabetic retinopathy with macular edema, right eye |  |  |  |  |  |  |  |  |  |
|  | E113412 | Type 2 diabetes mellitus with severe nonproliferative diabetic retinopathy with macular edema, left eye |  |  |  |  |  |  |  |  |  |
|  | E113413 | Type 2 diabetes mellitus with severe nonproliferative diabetic retinopathy with macular edema, bilateral |  |  |  |  |  |  |  |  |  |
|  | E113419 | Type 2 diabetes mellitus with severe nonproliferative diabetic retinopathy with macular edema, unspecified eye |  |  |  |  |  |  |  |  |  |
|  | E113491 | Type 2 diabetes mellitus with severe nonproliferative diabetic retinopathy without macular edema, right eye |  |  |  |  |  |  |  |  |  |
|  | E113492 | Type 2 diabetes mellitus with severe nonproliferative diabetic retinopathy without macular edema, left eye |  |  |  |  |  |  |  |  |  |
|  | E113493 | Type 2 diabetes mellitus with severe nonproliferative diabetic retinopathy without macular edema, bilateral |  |  |  |  |  |  |  |  |  |
|  | E113499 | Type 2 diabetes mellitus with severe nonproliferative diabetic retinopathy without macular edema, unspecified eye |  |  |  |  |  |  |  |  |  |
|  | E113511 | Type 2 diabetes mellitus with proliferative diabetic retinopathy with macular edema, right eye |  |  |  |  |  |  |  |  |  |
|  | E113512 | Type 2 diabetes mellitus with proliferative diabetic retinopathy with macular edema, left eye |  |  |  |  |  |  |  |  |  |
|  | E113513 | Type 2 diabetes mellitus with proliferative diabetic retinopathy with macular edema, bilateral |  |  |  |  |  |  |  |  |  |
|  | E113519 | Type 2 diabetes mellitus with proliferative diabetic retinopathy with macular edema, unspecified eye |  |  |  |  |  |  |  |  |  |
|  | E113521 | Type 2 diabetes mellitus with proliferative diabetic retinopathy with traction retinal detachment involving the macula, right eye |  |  |  |  |  |  |  |  |  |
|  | E113522 | Type 2 diabetes mellitus with proliferative diabetic retinopathy with traction retinal detachment involving the macula, left eye |  |  |  |  |  |  |  |  |  |
|  | E113523 | Type 2 diabetes mellitus with proliferative diabetic retinopathy with traction retinal detachment involving the macula, bilateral |  |  |  |  |  |  |  |  |  |
|  | E113529 | Type 2 diabetes mellitus with proliferative diabetic retinopathy with traction retinal detachment involving the macula, unspecified eye |  |  |  |  |  |  |  |  |  |
|  | E113531 | Type 2 diabetes mellitus with proliferative diabetic retinopathy with traction retinal detachment not involving the macula, right eye |  |  |  |  |  |  |  |  |  |
|  | E113532 | Type 2 diabetes mellitus with proliferative diabetic retinopathy with traction retinal detachment not involving the macula, left eye |  |  |  |  |  |  |  |  |  |
|  | E113533 | Type 2 diabetes mellitus with proliferative diabetic retinopathy with traction retinal detachment not involving the macula, bilateral |  |  |  |  |  |  |  |  |  |
|  | E113539 | Type 2 diabetes mellitus with proliferative diabetic retinopathy with traction retinal detachment not involving the macula, unspecified eye |  |  |  |  |  |  |  |  |  |
|  | E113541 | Type 2 diabetes mellitus with proliferative diabetic retinopathy with combined traction retinal detachment and rhegmatogenous retinal detachment, right eye |  |  |  |  |  |  |  |  |  |
|  | E113542 | Type 2 diabetes mellitus with proliferative diabetic retinopathy with combined traction retinal detachment and rhegmatogenous retinal detachment, left eye |  |  |  |  |  |  |  |  |  |
|  | E113543 | Type 2 diabetes mellitus with proliferative diabetic retinopathy with combined traction retinal detachment and rhegmatogenous retinal detachment, bilateral |  |  |  |  |  |  |  |  |  |
|  | E113549 | Type 2 diabetes mellitus with proliferative diabetic retinopathy with combined traction retinal detachment and rhegmatogenous retinal detachment, unspecified eye |  |  |  |  |  |  |  |  |  |
|  | E113551 | Type 2 diabetes mellitus with stable proliferative diabetic retinopathy, right eye |  |  |  |  |  |  |  |  |  |
|  | E113552 | Type 2 diabetes mellitus with stable proliferative diabetic retinopathy, left eye |  |  |  |  |  |  |  |  |  |
|  | E113553 | Type 2 diabetes mellitus with stable proliferative diabetic retinopathy, bilateral |  |  |  |  |  |  |  |  |  |
|  | E113559 | Type 2 diabetes mellitus with stable proliferative diabetic retinopathy, unspecified eye |  |  |  |  |  |  |  |  |  |
|  | E113591 | Type 2 diabetes mellitus with proliferative diabetic retinopathy without macular edema, right eye |  |  |  |  |  |  |  |  |  |
|  | E169 | Disorder of pancreatic internal secretion, unspecified |  |  |  |  |  |  |  |  |  |
|  | E113592 | Type 2 diabetes mellitus with proliferative diabetic retinopathy without macular edema, left eye |  |  |  |  |  |  |  |  |  |
|  | E113593 | Type 2 diabetes mellitus with proliferative diabetic retinopathy without macular edema, bilateral |  |  |  |  |  |  |  |  |  |
|  | E113599 | Type 2 diabetes mellitus with proliferative diabetic retinopathy without macular edema, unspecified eye |  |  |  |  |  |  |  |  |  |
|  | E1136 | Type 2 diabetes mellitus with diabetic cataract |  |  |  |  |  |  |  |  |  |
|  | E1137X1 | Type 2 diabetes mellitus with diabetic macular edema, resolved following treatment, right eye |  |  |  |  |  |  |  |  |  |
|  | E1137X2 | Type 2 diabetes mellitus with diabetic macular edema, resolved following treatment, left eye |  |  |  |  |  |  |  |  |  |
|  | E1137X3 | Type 2 diabetes mellitus with diabetic macular edema, resolved following treatment, bilateral |  |  |  |  |  |  |  |  |  |
|  | E1137X9 | Type 2 diabetes mellitus with diabetic macular edema, resolved following treatment, unspecified eye |  |  |  |  |  |  |  |  |  |
|  | E1139 | Type 2 diabetes mellitus with other diabetic ophthalmic complication |  |  |  |  |  |  |  |  |  |
|  | E1140 | Type 2 diabetes mellitus with diabetic neuropathy, unspecified |  |  |  |  |  |  |  |  |  |
|  | E1141 | Type 2 diabetes mellitus with diabetic mononeuropathy |  |  |  |  |  |  |  |  |  |
|  | E1142 | Type 2 diabetes mellitus with diabetic polyneuropathy |  |  |  |  |  |  |  |  |  |
|  | E1143 | Type 2 diabetes mellitus with diabetic autonomic (poly)neuropathy |  |  |  |  |  |  |  |  |  |
|  | E1144 | Type 2 diabetes mellitus with diabetic amyotrophy |  |  |  |  |  |  |  |  |  |
|  | E1149 | Type 2 diabetes mellitus with other diabetic neurological complication |  |  |  |  |  |  |  |  |  |
|  | E1151 | Type 2 diabetes mellitus with diabetic peripheral angiopathy without gangrene |  |  |  |  |  |  |  |  |  |
|  | E1152 | Type 2 diabetes mellitus with diabetic peripheral angiopathy with gangrene |  |  |  |  |  |  |  |  |  |
|  | E1159 | Type 2 diabetes mellitus with other circulatory complications |  |  |  |  |  |  |  |  |  |
|  | E11610 | Type 2 diabetes mellitus with diabetic neuropathic arthropathy |  |  |  |  |  |  |  |  |  |
|  | E11618 | Type 2 diabetes mellitus with other diabetic arthropathy |  |  |  |  |  |  |  |  |  |
|  | E11620 | Type 2 diabetes mellitus with diabetic dermatitis |  |  |  |  |  |  |  |  |  |
|  | E11621 | Type 2 diabetes mellitus with foot ulcer |  |  |  |  |  |  |  |  |  |
|  | E11622 | Type 2 diabetes mellitus with other skin ulcer |  |  |  |  |  |  |  |  |  |
|  | E11628 | Type 2 diabetes mellitus with other skin complications |  |  |  |  |  |  |  |  |  |
|  | E11630 | Type 2 diabetes mellitus with periodontal disease |  |  |  |  |  |  |  |  |  |
|  | E11638 | Type 2 diabetes mellitus with other oral complications |  |  |  |  |  |  |  |  |  |
|  | E11641 | Type 2 diabetes mellitus with hypoglycemia with coma |  |  |  |  |  |  |  |  |  |
|  | E11649 | Type 2 diabetes mellitus with hypoglycemia without coma |  |  |  |  |  |  |  |  |  |
|  | E1165 | Type 2 diabetes mellitus with hyperglycemia |  |  |  |  |  |  |  |  |  |
|  | E1169 | Type 2 diabetes mellitus with other specified complication |  |  |  |  |  |  |  |  |  |
|  | E118 | Type 2 diabetes mellitus with unspecified complications |  |  |  |  |  |  |  |  |  |
|  | E119 | Type 2 diabetes mellitus without complications |  |  |  |  |  |  |  |  |  |
|  | E1300 | Other specified diabetes mellitus with hyperosmolarity without nonketotic hyperglycemic-hyperosmolar coma (NKHHC) |  |  |  |  |  |  |  |  |  |
|  | E1301 | Other specified diabetes mellitus with hyperosmolarity with coma |  |  |  |  |  |  |  |  |  |
|  | E1310 | Other specified diabetes mellitus with ketoacidosis without coma |  |  |  |  |  |  |  |  |  |
|  | E1311 | Other specified diabetes mellitus with ketoacidosis with coma |  |  |  |  |  |  |  |  |  |
|  | E1321 | Other specified diabetes mellitus with diabetic nephropathy |  |  |  |  |  |  |  |  |  |
|  | E1322 | Other specified diabetes mellitus with diabetic chronic kidney disease |  |  |  |  |  |  |  |  |  |
|  | E1329 | Other specified diabetes mellitus with other diabetic kidney complication |  |  |  |  |  |  |  |  |  |
|  | E13311 | Other specified diabetes mellitus with unspecified diabetic retinopathy with macular edema |  |  |  |  |  |  |  |  |  |
|  | E13319 | Other specified diabetes mellitus with unspecified diabetic retinopathy without macular edema |  |  |  |  |  |  |  |  |  |
|  | E133211 | Other specified diabetes mellitus with mild nonproliferative diabetic retinopathy with macular edema, right eye |  |  |  |  |  |  |  |  |  |
|  | E133212 | Other specified diabetes mellitus with mild nonproliferative diabetic retinopathy with macular edema, left eye |  |  |  |  |  |  |  |  |  |
|  | E133213 | Other specified diabetes mellitus with mild nonproliferative diabetic retinopathy with macular edema, bilateral |  |  |  |  |  |  |  |  |  |
|  | E133219 | Other specified diabetes mellitus with mild nonproliferative diabetic retinopathy with macular edema, unspecified eye |  |  |  |  |  |  |  |  |  |
|  | E133291 | Other specified diabetes mellitus with mild nonproliferative diabetic retinopathy without macular edema, right eye |  |  |  |  |  |  |  |  |  |
|  | E133292 | Other specified diabetes mellitus with mild nonproliferative diabetic retinopathy without macular edema, left eye |  |  |  |  |  |  |  |  |  |
|  | E133293 | Other specified diabetes mellitus with mild nonproliferative diabetic retinopathy without macular edema, bilateral |  |  |  |  |  |  |  |  |  |
|  | E133299 | Other specified diabetes mellitus with mild nonproliferative diabetic retinopathy without macular edema, unspecified eye |  |  |  |  |  |  |  |  |  |
|  | E133311 | Other specified diabetes mellitus with moderate nonproliferative diabetic retinopathy with macular edema, right eye |  |  |  |  |  |  |  |  |  |
|  | E133312 | Other specified diabetes mellitus with moderate nonproliferative diabetic retinopathy with macular edema, left eye |  |  |  |  |  |  |  |  |  |
|  | E133313 | Other specified diabetes mellitus with moderate nonproliferative diabetic retinopathy with macular edema, bilateral |  |  |  |  |  |  |  |  |  |
|  | E133319 | Other specified diabetes mellitus with moderate nonproliferative diabetic retinopathy with macular edema, unspecified eye |  |  |  |  |  |  |  |  |  |
|  | E133391 | Other specified diabetes mellitus with moderate nonproliferative diabetic retinopathy without macular edema, right eye |  |  |  |  |  |  |  |  |  |
|  | E133392 | Other specified diabetes mellitus with moderate nonproliferative diabetic retinopathy without macular edema, left eye |  |  |  |  |  |  |  |  |  |
|  | E133393 | Other specified diabetes mellitus with moderate nonproliferative diabetic retinopathy without macular edema, bilateral |  |  |  |  |  |  |  |  |  |
|  | E133399 | Other specified diabetes mellitus with moderate nonproliferative diabetic retinopathy without macular edema, unspecified eye |  |  |  |  |  |  |  |  |  |
|  | E133411 | Other specified diabetes mellitus with severe nonproliferative diabetic retinopathy with macular edema, right eye |  |  |  |  |  |  |  |  |  |
|  | E133412 | Other specified diabetes mellitus with severe nonproliferative diabetic retinopathy with macular edema, left eye |  |  |  |  |  |  |  |  |  |
|  | E133413 | Other specified diabetes mellitus with severe nonproliferative diabetic retinopathy with macular edema, bilateral |  |  |  |  |  |  |  |  |  |
|  | E133419 | Other specified diabetes mellitus with severe nonproliferative diabetic retinopathy with macular edema, unspecified eye |  |  |  |  |  |  |  |  |  |
|  | E133491 | Other specified diabetes mellitus with severe nonproliferative diabetic retinopathy without macular edema, right eye |  |  |  |  |  |  |  |  |  |
|  | E133492 | Other specified diabetes mellitus with severe nonproliferative diabetic retinopathy without macular edema, left eye |  |  |  |  |  |  |  |  |  |
|  | E133493 | Other specified diabetes mellitus with severe nonproliferative diabetic retinopathy without macular edema, bilateral |  |  |  |  |  |  |  |  |  |
|  | E133499 | Other specified diabetes mellitus with severe nonproliferative diabetic retinopathy without macular edema, unspecified eye |  |  |  |  |  |  |  |  |  |
|  | E133511 | Other specified diabetes mellitus with proliferative diabetic retinopathy with macular edema, right eye |  |  |  |  |  |  |  |  |  |
|  | E133512 | Other specified diabetes mellitus with proliferative diabetic retinopathy with macular edema, left eye |  |  |  |  |  |  |  |  |  |
|  | E133513 | Other specified diabetes mellitus with proliferative diabetic retinopathy with macular edema, bilateral |  |  |  |  |  |  |  |  |  |
|  | E133519 | Other specified diabetes mellitus with proliferative diabetic retinopathy with macular edema, unspecified eye |  |  |  |  |  |  |  |  |  |
|  | E133521 | Other specified diabetes mellitus with proliferative diabetic retinopathy with traction retinal detachment involving the macula, right eye |  |  |  |  |  |  |  |  |  |
|  | E133522 | Other specified diabetes mellitus with proliferative diabetic retinopathy with traction retinal detachment involving the macula, left eye |  |  |  |  |  |  |  |  |  |
|  | E133523 | Other specified diabetes mellitus with proliferative diabetic retinopathy with traction retinal detachment involving the macula, bilateral |  |  |  |  |  |  |  |  |  |
|  | E133529 | Other specified diabetes mellitus with proliferative diabetic retinopathy with traction retinal detachment involving the macula, unspecified eye |  |  |  |  |  |  |  |  |  |
|  | E133531 | Other specified diabetes mellitus with proliferative diabetic retinopathy with traction retinal detachment not involving the macula, right eye |  |  |  |  |  |  |  |  |  |
|  | E133532 | Other specified diabetes mellitus with proliferative diabetic retinopathy with traction retinal detachment not involving the macula, left eye |  |  |  |  |  |  |  |  |  |
|  | E133533 | Other specified diabetes mellitus with proliferative diabetic retinopathy with traction retinal detachment not involving the macula, bilateral |  |  |  |  |  |  |  |  |  |
|  | E133539 | Other specified diabetes mellitus with proliferative diabetic retinopathy with traction retinal detachment not involving the macula, unspecified eye |  |  |  |  |  |  |  |  |  |
|  | E133541 | Other specified diabetes mellitus with proliferative diabetic retinopathy with combined traction retinal detachment and rhegmatogenous retinal detachment, right eye |  |  |  |  |  |  |  |  |  |
|  | E133542 | Other specified diabetes mellitus with proliferative diabetic retinopathy with combined traction retinal detachment and rhegmatogenous retinal detachment, left eye |  |  |  |  |  |  |  |  |  |
|  | E133543 | Other specified diabetes mellitus with proliferative diabetic retinopathy with combined traction retinal detachment and rhegmatogenous retinal detachment, bilateral |  |  |  |  |  |  |  |  |  |
|  | E133549 | Other specified diabetes mellitus with proliferative diabetic retinopathy with combined traction retinal detachment and rhegmatogenous retinal detachment, unspecified eye |  |  |  |  |  |  |  |  |  |
|  | E133551 | Other specified diabetes mellitus with stable proliferative diabetic retinopathy, right eye |  |  |  |  |  |  |  |  |  |
|  | E133552 | Other specified diabetes mellitus with stable proliferative diabetic retinopathy, left eye |  |  |  |  |  |  |  |  |  |
|  | E133553 | Other specified diabetes mellitus with stable proliferative diabetic retinopathy, bilateral |  |  |  |  |  |  |  |  |  |
|  | E133559 | Other specified diabetes mellitus with stable proliferative diabetic retinopathy, unspecified eye |  |  |  |  |  |  |  |  |  |
|  | E133591 | Other specified diabetes mellitus with proliferative diabetic retinopathy without macular edema, right eye |  |  |  |  |  |  |  |  |  |
|  | E133592 | Other specified diabetes mellitus with proliferative diabetic retinopathy without macular edema, left eye |  |  |  |  |  |  |  |  |  |
|  | E133593 | Other specified diabetes mellitus with proliferative diabetic retinopathy without macular edema, bilateral |  |  |  |  |  |  |  |  |  |
|  | E133599 | Other specified diabetes mellitus with proliferative diabetic retinopathy without macular edema, unspecified eye |  |  |  |  |  |  |  |  |  |
|  | E1336 | Other specified diabetes mellitus with diabetic cataract |  |  |  |  |  |  |  |  |  |
|  | E1337X1 | Other specified diabetes mellitus with diabetic macular edema, resolved following treatment, right eye |  |  |  |  |  |  |  |  |  |
|  | E1337X2 | Other specified diabetes mellitus with diabetic macular edema, resolved following treatment, left eye |  |  |  |  |  |  |  |  |  |
|  | E1337X3 | Other specified diabetes mellitus with diabetic macular edema, resolved following treatment, bilateral |  |  |  |  |  |  |  |  |  |
|  | E1337X9 | Other specified diabetes mellitus with diabetic macular edema, resolved following treatment, unspecified eye |  |  |  |  |  |  |  |  |  |
|  | E1339 | Other specified diabetes mellitus with other diabetic ophthalmic complication |  |  |  |  |  |  |  |  |  |
|  | E1340 | Other specified diabetes mellitus with diabetic neuropathy, unspecified |  |  |  |  |  |  |  |  |  |
|  | E1341 | Other specified diabetes mellitus with diabetic mononeuropathy |  |  |  |  |  |  |  |  |  |
|  | E1342 | Other specified diabetes mellitus with diabetic polyneuropathy |  |  |  |  |  |  |  |  |  |
|  | E1343 | Other specified diabetes mellitus with diabetic autonomic (poly)neuropathy |  |  |  |  |  |  |  |  |  |
|  | E1344 | Other specified diabetes mellitus with diabetic amyotrophy |  |  |  |  |  |  |  |  |  |
|  | E1349 | Other specified diabetes mellitus with other diabetic neurological complication |  |  |  |  |  |  |  |  |  |
|  | E1351 | Other specified diabetes mellitus with diabetic peripheral angiopathy without gangrene |  |  |  |  |  |  |  |  |  |
|  | E1352 | Other specified diabetes mellitus with diabetic peripheral angiopathy with gangrene |  |  |  |  |  |  |  |  |  |
|  | E1359 | Other specified diabetes mellitus with other circulatory complications |  |  |  |  |  |  |  |  |  |
|  | E13610 | Other specified diabetes mellitus with diabetic neuropathic arthropathy |  |  |  |  |  |  |  |  |  |
|  | E13618 | Other specified diabetes mellitus with other diabetic arthropathy |  |  |  |  |  |  |  |  |  |
|  | E13620 | Other specified diabetes mellitus with diabetic dermatitis |  |  |  |  |  |  |  |  |  |
|  | E13621 | Other specified diabetes mellitus with foot ulcer |  |  |  |  |  |  |  |  |  |
|  | E13622 | Other specified diabetes mellitus with other skin ulcer |  |  |  |  |  |  |  |  |  |
|  | E13628 | Other specified diabetes mellitus with other skin complications |  |  |  |  |  |  |  |  |  |
|  | E13630 | Other specified diabetes mellitus with periodontal disease |  |  |  |  |  |  |  |  |  |
|  | E13638 | Other specified diabetes mellitus with other oral complications |  |  |  |  |  |  |  |  |  |
|  | E13641 | Other specified diabetes mellitus with hypoglycemia with coma |  |  |  |  |  |  |  |  |  |
|  | E13649 | Other specified diabetes mellitus with hypoglycemia without coma |  |  |  |  |  |  |  |  |  |
|  | E1365 | Other specified diabetes mellitus with hyperglycemia |  |  |  |  |  |  |  |  |  |
|  | E1369 | Other specified diabetes mellitus with other specified complication |  |  |  |  |  |  |  |  |  |
|  | E138 | Other specified diabetes mellitus with unspecified complications |  |  |  |  |  |  |  |  |  |
|  | E139 | Other specified diabetes mellitus without complications |  |  |  |  |  |  |  |  |  |
|  | E0900 | Drug or chemical induced diabetes mellitus with hyperosmolarity without nonketotic hyperglycemic-hyperosmolar coma (NKHHC) |  |  |  |  |  |  |  |  |  |
|  | E0901 | Drug or chemical induced diabetes mellitus with hyperosmolarity with coma |  |  |  |  |  |  |  |  |  |
|  | E0910 | Drug or chemical induced diabetes mellitus with ketoacidosis without coma |  |  |  |  |  |  |  |  |  |
|  | E0911 | Drug or chemical induced diabetes mellitus with ketoacidosis with coma |  |  |  |  |  |  |  |  |  |
|  | E0921 | Drug or chemical induced diabetes mellitus with diabetic nephropathy |  |  |  |  |  |  |  |  |  |
|  | E0922 | Drug or chemical induced diabetes mellitus with diabetic chronic kidney disease |  |  |  |  |  |  |  |  |  |
|  | E0929 | Drug or chemical induced diabetes mellitus with other diabetic kidney complication |  |  |  |  |  |  |  |  |  |
|  | E09311 | Drug or chemical induced diabetes mellitus with unspecified diabetic retinopathy with macular edema |  |  |  |  |  |  |  |  |  |
|  | E09319 | Drug or chemical induced diabetes mellitus with unspecified diabetic retinopathy without macular edema |  |  |  |  |  |  |  |  |  |
|  | E093211 | Drug or chemical induced diabetes mellitus with mild nonproliferative diabetic retinopathy with macular edema, right eye |  |  |  |  |  |  |  |  |  |
|  | E093212 | Drug or chemical induced diabetes mellitus with mild nonproliferative diabetic retinopathy with macular edema, left eye |  |  |  |  |  |  |  |  |  |
|  | E093213 | Drug or chemical induced diabetes mellitus with mild nonproliferative diabetic retinopathy with macular edema, bilateral |  |  |  |  |  |  |  |  |  |
|  | E093219 | Drug or chemical induced diabetes mellitus with mild nonproliferative diabetic retinopathy with macular edema, unspecified eye |  |  |  |  |  |  |  |  |  |
|  | E093291 | Drug or chemical induced diabetes mellitus with mild nonproliferative diabetic retinopathy without macular edema, right eye |  |  |  |  |  |  |  |  |  |
|  | E093292 | Drug or chemical induced diabetes mellitus with mild nonproliferative diabetic retinopathy without macular edema, left eye |  |  |  |  |  |  |  |  |  |
|  | E093293 | Drug or chemical induced diabetes mellitus with mild nonproliferative diabetic retinopathy without macular edema, bilateral |  |  |  |  |  |  |  |  |  |
|  | E093299 | Drug or chemical induced diabetes mellitus with mild nonproliferative diabetic retinopathy without macular edema, unspecified eye |  |  |  |  |  |  |  |  |  |
|  | E093311 | Drug or chemical induced diabetes mellitus with moderate nonproliferative diabetic retinopathy with macular edema, right eye |  |  |  |  |  |  |  |  |  |
|  | E093312 | Drug or chemical induced diabetes mellitus with moderate nonproliferative diabetic retinopathy with macular edema, left eye |  |  |  |  |  |  |  |  |  |
|  | E093313 | Drug or chemical induced diabetes mellitus with moderate nonproliferative diabetic retinopathy with macular edema, bilateral |  |  |  |  |  |  |  |  |  |
|  | E093319 | Drug or chemical induced diabetes mellitus with moderate nonproliferative diabetic retinopathy with macular edema, unspecified eye |  |  |  |  |  |  |  |  |  |
|  | E093391 | Drug or chemical induced diabetes mellitus with moderate nonproliferative diabetic retinopathy without macular edema, right eye |  |  |  |  |  |  |  |  |  |
|  | E093392 | Drug or chemical induced diabetes mellitus with moderate nonproliferative diabetic retinopathy without macular edema, left eye |  |  |  |  |  |  |  |  |  |
|  | E093393 | Drug or chemical induced diabetes mellitus with moderate nonproliferative diabetic retinopathy without macular edema, bilateral |  |  |  |  |  |  |  |  |  |
|  | E093399 | Drug or chemical induced diabetes mellitus with moderate nonproliferative diabetic retinopathy without macular edema, unspecified eye |  |  |  |  |  |  |  |  |  |
|  | E093411 | Drug or chemical induced diabetes mellitus with severe nonproliferative diabetic retinopathy with macular edema, right eye |  |  |  |  |  |  |  |  |  |
|  | E093412 | Drug or chemical induced diabetes mellitus with severe nonproliferative diabetic retinopathy with macular edema, left eye |  |  |  |  |  |  |  |  |  |
|  | E093413 | Drug or chemical induced diabetes mellitus with severe nonproliferative diabetic retinopathy with macular edema, bilateral |  |  |  |  |  |  |  |  |  |
|  | E093419 | Drug or chemical induced diabetes mellitus with severe nonproliferative diabetic retinopathy with macular edema, unspecified eye |  |  |  |  |  |  |  |  |  |
|  | E093491 | Drug or chemical induced diabetes mellitus with severe nonproliferative diabetic retinopathy without macular edema, right eye |  |  |  |  |  |  |  |  |  |
|  | E093492 | Drug or chemical induced diabetes mellitus with severe nonproliferative diabetic retinopathy without macular edema, left eye |  |  |  |  |  |  |  |  |  |
|  | E093493 | Drug or chemical induced diabetes mellitus with severe nonproliferative diabetic retinopathy without macular edema, bilateral |  |  |  |  |  |  |  |  |  |
|  | E093499 | Drug or chemical induced diabetes mellitus with severe nonproliferative diabetic retinopathy without macular edema, unspecified eye |  |  |  |  |  |  |  |  |  |
|  | E093511 | Drug or chemical induced diabetes mellitus with proliferative diabetic retinopathy with macular edema, right eye |  |  |  |  |  |  |  |  |  |
|  | E093512 | Drug or chemical induced diabetes mellitus with proliferative diabetic retinopathy with macular edema, left eye |  |  |  |  |  |  |  |  |  |
|  | E093513 | Drug or chemical induced diabetes mellitus with proliferative diabetic retinopathy with macular edema, bilateral |  |  |  |  |  |  |  |  |  |
|  | E093519 | Drug or chemical induced diabetes mellitus with proliferative diabetic retinopathy with macular edema, unspecified eye |  |  |  |  |  |  |  |  |  |
|  | E093521 | Drug or chemical induced diabetes mellitus with proliferative diabetic retinopathy with traction retinal detachment involving the macula, right eye |  |  |  |  |  |  |  |  |  |
|  | E093522 | Drug or chemical induced diabetes mellitus with proliferative diabetic retinopathy with traction retinal detachment involving the macula, left eye |  |  |  |  |  |  |  |  |  |
|  | E093523 | Drug or chemical induced diabetes mellitus with proliferative diabetic retinopathy with traction retinal detachment involving the macula, bilateral |  |  |  |  |  |  |  |  |  |
|  | E093529 | Drug or chemical induced diabetes mellitus with proliferative diabetic retinopathy with traction retinal detachment involving the macula, unspecified eye |  |  |  |  |  |  |  |  |  |
|  | E093531 | Drug or chemical induced diabetes mellitus with proliferative diabetic retinopathy with traction retinal detachment not involving the macula, right eye |  |  |  |  |  |  |  |  |  |
|  | E093532 | Drug or chemical induced diabetes mellitus with proliferative diabetic retinopathy with traction retinal detachment not involving the macula, left eye |  |  |  |  |  |  |  |  |  |
|  | E093533 | Drug or chemical induced diabetes mellitus with proliferative diabetic retinopathy with traction retinal detachment not involving the macula, bilateral |  |  |  |  |  |  |  |  |  |
|  | E093539 | Drug or chemical induced diabetes mellitus with proliferative diabetic retinopathy with traction retinal detachment not involving the macula, unspecified eye |  |  |  |  |  |  |  |  |  |
|  | E093541 | Drug or chemical induced diabetes mellitus with proliferative diabetic retinopathy with combined traction retinal detachment and rhegmatogenous retinal detachment, right eye |  |  |  |  |  |  |  |  |  |
|  | E093542 | Drug or chemical induced diabetes mellitus with proliferative diabetic retinopathy with combined traction retinal detachment and rhegmatogenous retinal detachment, left eye |  |  |  |  |  |  |  |  |  |
|  | E093543 | Drug or chemical induced diabetes mellitus with proliferative diabetic retinopathy with combined traction retinal detachment and rhegmatogenous retinal detachment, bilateral |  |  |  |  |  |  |  |  |  |
|  | E208 | Other hypoparathyroidism |  |  |  |  |  |  |  |  |  |
|  | E093549 | Drug or chemical induced diabetes mellitus with proliferative diabetic retinopathy with combined traction retinal detachment and rhegmatogenous retinal detachment, unspecified eye |  |  |  |  |  |  |  |  |  |
|  | E093551 | Drug or chemical induced diabetes mellitus with stable proliferative diabetic retinopathy, right eye |  |  |  |  |  |  |  |  |  |
|  | E093552 | Drug or chemical induced diabetes mellitus with stable proliferative diabetic retinopathy, left eye |  |  |  |  |  |  |  |  |  |
|  | E093553 | Drug or chemical induced diabetes mellitus with stable proliferative diabetic retinopathy, bilateral |  |  |  |  |  |  |  |  |  |
|  | E093559 | Drug or chemical induced diabetes mellitus with stable proliferative diabetic retinopathy, unspecified eye |  |  |  |  |  |  |  |  |  |
|  | E093591 | Drug or chemical induced diabetes mellitus with proliferative diabetic retinopathy without macular edema, right eye |  |  |  |  |  |  |  |  |  |
|  | E093592 | Drug or chemical induced diabetes mellitus with proliferative diabetic retinopathy without macular edema, left eye |  |  |  |  |  |  |  |  |  |
|  | E093593 | Drug or chemical induced diabetes mellitus with proliferative diabetic retinopathy without macular edema, bilateral |  |  |  |  |  |  |  |  |  |
|  | E093599 | Drug or chemical induced diabetes mellitus with proliferative diabetic retinopathy without macular edema, unspecified eye |  |  |  |  |  |  |  |  |  |
|  | E0936 | Drug or chemical induced diabetes mellitus with diabetic cataract |  |  |  |  |  |  |  |  |  |
|  | E0937X1 | Drug or chemical induced diabetes mellitus with diabetic macular edema, resolved following treatment, right eye |  |  |  |  |  |  |  |  |  |
|  | E0937X2 | Drug or chemical induced diabetes mellitus with diabetic macular edema, resolved following treatment, left eye |  |  |  |  |  |  |  |  |  |
|  | E0937X3 | Drug or chemical induced diabetes mellitus with diabetic macular edema, resolved following treatment, bilateral |  |  |  |  |  |  |  |  |  |
|  | E0937X9 | Drug or chemical induced diabetes mellitus with diabetic macular edema, resolved following treatment, unspecified eye |  |  |  |  |  |  |  |  |  |
|  | E0939 | Drug or chemical induced diabetes mellitus with other diabetic ophthalmic complication |  |  |  |  |  |  |  |  |  |
|  | E0940 | Drug or chemical induced diabetes mellitus with neurological complications with diabetic neuropathy, unspecified |  |  |  |  |  |  |  |  |  |
|  | E0941 | Drug or chemical induced diabetes mellitus with neurological complications with diabetic mononeuropathy |  |  |  |  |  |  |  |  |  |
|  | E0942 | Drug or chemical induced diabetes mellitus with neurological complications with diabetic polyneuropathy |  |  |  |  |  |  |  |  |  |
|  | E0943 | Drug or chemical induced diabetes mellitus with neurological complications with diabetic autonomic (poly)neuropathy |  |  |  |  |  |  |  |  |  |
|  | E0944 | Drug or chemical induced diabetes mellitus with neurological complications with diabetic amyotrophy |  |  |  |  |  |  |  |  |  |
|  | E0949 | Drug or chemical induced diabetes mellitus with neurological complications with other diabetic neurological complication |  |  |  |  |  |  |  |  |  |
|  | E0951 | Drug or chemical induced diabetes mellitus with diabetic peripheral angiopathy without gangrene |  |  |  |  |  |  |  |  |  |
|  | E0952 | Drug or chemical induced diabetes mellitus with diabetic peripheral angiopathy with gangrene |  |  |  |  |  |  |  |  |  |
|  | E0959 | Drug or chemical induced diabetes mellitus with other circulatory complications |  |  |  |  |  |  |  |  |  |
|  | E09610 | Drug or chemical induced diabetes mellitus with diabetic neuropathic arthropathy |  |  |  |  |  |  |  |  |  |
|  | E09618 | Drug or chemical induced diabetes mellitus with other diabetic arthropathy |  |  |  |  |  |  |  |  |  |
|  | E09620 | Drug or chemical induced diabetes mellitus with diabetic dermatitis |  |  |  |  |  |  |  |  |  |
|  | E09621 | Drug or chemical induced diabetes mellitus with foot ulcer |  |  |  |  |  |  |  |  |  |
|  | E09622 | Drug or chemical induced diabetes mellitus with other skin ulcer |  |  |  |  |  |  |  |  |  |
|  | E09628 | Drug or chemical induced diabetes mellitus with other skin complications |  |  |  |  |  |  |  |  |  |
|  | E09630 | Drug or chemical induced diabetes mellitus with periodontal disease |  |  |  |  |  |  |  |  |  |
|  | E09638 | Drug or chemical induced diabetes mellitus with other oral complications |  |  |  |  |  |  |  |  |  |
|  | E09641 | Drug or chemical induced diabetes mellitus with hypoglycemia with coma |  |  |  |  |  |  |  |  |  |
|  | E09649 | Drug or chemical induced diabetes mellitus with hypoglycemia without coma |  |  |  |  |  |  |  |  |  |
|  | E0965 | Drug or chemical induced diabetes mellitus with hyperglycemia |  |  |  |  |  |  |  |  |  |
|  | E0969 | Drug or chemical induced diabetes mellitus with other specified complication |  |  |  |  |  |  |  |  |  |
|  | E098 | Drug or chemical induced diabetes mellitus with unspecified complications |  |  |  |  |  |  |  |  |  |
|  | E099 | Drug or chemical induced diabetes mellitus without complications |  |  |  |  |  |  |  |  |  |
|  | E1010 | Type 1 diabetes mellitus with ketoacidosis without coma |  |  |  |  |  |  |  |  |  |
|  | E1011 | Type 1 diabetes mellitus with ketoacidosis with coma |  |  |  |  |  |  |  |  |  |
|  | E1021 | Type 1 diabetes mellitus with diabetic nephropathy |  |  |  |  |  |  |  |  |  |
|  | E1022 | Type 1 diabetes mellitus with diabetic chronic kidney disease |  |  |  |  |  |  |  |  |  |
|  | E1029 | Type 1 diabetes mellitus with other diabetic kidney complication |  |  |  |  |  |  |  |  |  |
|  | E10311 | Type 1 diabetes mellitus with unspecified diabetic retinopathy with macular edema |  |  |  |  |  |  |  |  |  |
|  | E10319 | Type 1 diabetes mellitus with unspecified diabetic retinopathy without macular edema |  |  |  |  |  |  |  |  |  |
|  | E103211 | Type 1 diabetes mellitus with mild nonproliferative diabetic retinopathy with macular edema, right eye |  |  |  |  |  |  |  |  |  |
|  | E103212 | Type 1 diabetes mellitus with mild nonproliferative diabetic retinopathy with macular edema, left eye |  |  |  |  |  |  |  |  |  |
|  | E103213 | Type 1 diabetes mellitus with mild nonproliferative diabetic retinopathy with macular edema, bilateral |  |  |  |  |  |  |  |  |  |
|  | E103219 | Type 1 diabetes mellitus with mild nonproliferative diabetic retinopathy with macular edema, unspecified eye |  |  |  |  |  |  |  |  |  |
|  | E103291 | Type 1 diabetes mellitus with mild nonproliferative diabetic retinopathy without macular edema, right eye |  |  |  |  |  |  |  |  |  |
|  | E103292 | Type 1 diabetes mellitus with mild nonproliferative diabetic retinopathy without macular edema, left eye |  |  |  |  |  |  |  |  |  |
|  | E103293 | Type 1 diabetes mellitus with mild nonproliferative diabetic retinopathy without macular edema, bilateral |  |  |  |  |  |  |  |  |  |
|  | E103299 | Type 1 diabetes mellitus with mild nonproliferative diabetic retinopathy without macular edema, unspecified eye |  |  |  |  |  |  |  |  |  |
|  | E103311 | Type 1 diabetes mellitus with moderate nonproliferative diabetic retinopathy with macular edema, right eye |  |  |  |  |  |  |  |  |  |
|  | E103312 | Type 1 diabetes mellitus with moderate nonproliferative diabetic retinopathy with macular edema, left eye |  |  |  |  |  |  |  |  |  |
|  | E103313 | Type 1 diabetes mellitus with moderate nonproliferative diabetic retinopathy with macular edema, bilateral |  |  |  |  |  |  |  |  |  |
|  | E103319 | Type 1 diabetes mellitus with moderate nonproliferative diabetic retinopathy with macular edema, unspecified eye |  |  |  |  |  |  |  |  |  |
|  | E103391 | Type 1 diabetes mellitus with moderate nonproliferative diabetic retinopathy without macular edema, right eye |  |  |  |  |  |  |  |  |  |
|  | E103392 | Type 1 diabetes mellitus with moderate nonproliferative diabetic retinopathy without macular edema, left eye |  |  |  |  |  |  |  |  |  |
|  | E103393 | Type 1 diabetes mellitus with moderate nonproliferative diabetic retinopathy without macular edema, bilateral |  |  |  |  |  |  |  |  |  |
|  | E103399 | Type 1 diabetes mellitus with moderate nonproliferative diabetic retinopathy without macular edema, unspecified eye |  |  |  |  |  |  |  |  |  |
|  | E103411 | Type 1 diabetes mellitus with severe nonproliferative diabetic retinopathy with macular edema, right eye |  |  |  |  |  |  |  |  |  |
|  | E103412 | Type 1 diabetes mellitus with severe nonproliferative diabetic retinopathy with macular edema, left eye |  |  |  |  |  |  |  |  |  |
|  | E103413 | Type 1 diabetes mellitus with severe nonproliferative diabetic retinopathy with macular edema, bilateral |  |  |  |  |  |  |  |  |  |
|  | E103419 | Type 1 diabetes mellitus with severe nonproliferative diabetic retinopathy with macular edema, unspecified eye |  |  |  |  |  |  |  |  |  |
|  | E103491 | Type 1 diabetes mellitus with severe nonproliferative diabetic retinopathy without macular edema, right eye |  |  |  |  |  |  |  |  |  |
|  | E103492 | Type 1 diabetes mellitus with severe nonproliferative diabetic retinopathy without macular edema, left eye |  |  |  |  |  |  |  |  |  |
|  | E103493 | Type 1 diabetes mellitus with severe nonproliferative diabetic retinopathy without macular edema, bilateral |  |  |  |  |  |  |  |  |  |
|  | E103499 | Type 1 diabetes mellitus with severe nonproliferative diabetic retinopathy without macular edema, unspecified eye |  |  |  |  |  |  |  |  |  |
|  | E103511 | Type 1 diabetes mellitus with proliferative diabetic retinopathy with macular edema, right eye |  |  |  |  |  |  |  |  |  |
|  | E103512 | Type 1 diabetes mellitus with proliferative diabetic retinopathy with macular edema, left eye |  |  |  |  |  |  |  |  |  |
|  | E103513 | Type 1 diabetes mellitus with proliferative diabetic retinopathy with macular edema, bilateral |  |  |  |  |  |  |  |  |  |
|  | E103519 | Type 1 diabetes mellitus with proliferative diabetic retinopathy with macular edema, unspecified eye |  |  |  |  |  |  |  |  |  |
|  | E103521 | Type 1 diabetes mellitus with proliferative diabetic retinopathy with traction retinal detachment involving the macula, right eye |  |  |  |  |  |  |  |  |  |
|  | E103522 | Type 1 diabetes mellitus with proliferative diabetic retinopathy with traction retinal detachment involving the macula, left eye |  |  |  |  |  |  |  |  |  |
|  | E103523 | Type 1 diabetes mellitus with proliferative diabetic retinopathy with traction retinal detachment involving the macula, bilateral |  |  |  |  |  |  |  |  |  |
|  | E103529 | Type 1 diabetes mellitus with proliferative diabetic retinopathy with traction retinal detachment involving the macula, unspecified eye |  |  |  |  |  |  |  |  |  |
|  | E103531 | Type 1 diabetes mellitus with proliferative diabetic retinopathy with traction retinal detachment not involving the macula, right eye |  |  |  |  |  |  |  |  |  |
|  | E103532 | Type 1 diabetes mellitus with proliferative diabetic retinopathy with traction retinal detachment not involving the macula, left eye |  |  |  |  |  |  |  |  |  |
|  | E103533 | Type 1 diabetes mellitus with proliferative diabetic retinopathy with traction retinal detachment not involving the macula, bilateral |  |  |  |  |  |  |  |  |  |
|  | E103539 | Type 1 diabetes mellitus with proliferative diabetic retinopathy with traction retinal detachment not involving the macula, unspecified eye |  |  |  |  |  |  |  |  |  |
|  | E103541 | Type 1 diabetes mellitus with proliferative diabetic retinopathy with combined traction retinal detachment and rhegmatogenous retinal detachment, right eye |  |  |  |  |  |  |  |  |  |
|  | E103542 | Type 1 diabetes mellitus with proliferative diabetic retinopathy with combined traction retinal detachment and rhegmatogenous retinal detachment, left eye |  |  |  |  |  |  |  |  |  |
|  | E103543 | Type 1 diabetes mellitus with proliferative diabetic retinopathy with combined traction retinal detachment and rhegmatogenous retinal detachment, bilateral |  |  |  |  |  |  |  |  |  |
|  | E103549 | Type 1 diabetes mellitus with proliferative diabetic retinopathy with combined traction retinal detachment and rhegmatogenous retinal detachment, unspecified eye |  |  |  |  |  |  |  |  |  |
|  | E103551 | Type 1 diabetes mellitus with stable proliferative diabetic retinopathy, right eye |  |  |  |  |  |  |  |  |  |
|  | E103552 | Type 1 diabetes mellitus with stable proliferative diabetic retinopathy, left eye |  |  |  |  |  |  |  |  |  |
|  | E103553 | Type 1 diabetes mellitus with stable proliferative diabetic retinopathy, bilateral |  |  |  |  |  |  |  |  |  |
|  | E103559 | Type 1 diabetes mellitus with stable proliferative diabetic retinopathy, unspecified eye |  |  |  |  |  |  |  |  |  |
|  | E103591 | Type 1 diabetes mellitus with proliferative diabetic retinopathy without macular edema, right eye |  |  |  |  |  |  |  |  |  |
|  | E103592 | Type 1 diabetes mellitus with proliferative diabetic retinopathy without macular edema, left eye |  |  |  |  |  |  |  |  |  |
|  | E103593 | Type 1 diabetes mellitus with proliferative diabetic retinopathy without macular edema, bilateral |  |  |  |  |  |  |  |  |  |
|  | E103599 | Type 1 diabetes mellitus with proliferative diabetic retinopathy without macular edema, unspecified eye |  |  |  |  |  |  |  |  |  |
|  | E1036 | Type 1 diabetes mellitus with diabetic cataract |  |  |  |  |  |  |  |  |  |
|  | E1037X1 | Type 1 diabetes mellitus with diabetic macular edema, resolved following treatment, right eye |  |  |  |  |  |  |  |  |  |
|  | E1037X2 | Type 1 diabetes mellitus with diabetic macular edema, resolved following treatment, left eye |  |  |  |  |  |  |  |  |  |
|  | E1037X3 | Type 1 diabetes mellitus with diabetic macular edema, resolved following treatment, bilateral |  |  |  |  |  |  |  |  |  |
|  | E1037X9 | Type 1 diabetes mellitus with diabetic macular edema, resolved following treatment, unspecified eye |  |  |  |  |  |  |  |  |  |
|  | E1039 | Type 1 diabetes mellitus with other diabetic ophthalmic complication |  |  |  |  |  |  |  |  |  |
|  | E1040 | Type 1 diabetes mellitus with diabetic neuropathy, unspecified |  |  |  |  |  |  |  |  |  |
|  | E1041 | Type 1 diabetes mellitus with diabetic mononeuropathy |  |  |  |  |  |  |  |  |  |
|  | E1042 | Type 1 diabetes mellitus with diabetic polyneuropathy |  |  |  |  |  |  |  |  |  |
|  | E1043 | Type 1 diabetes mellitus with diabetic autonomic (poly)neuropathy |  |  |  |  |  |  |  |  |  |
|  | E1044 | Type 1 diabetes mellitus with diabetic amyotrophy |  |  |  |  |  |  |  |  |  |
|  | E1049 | Type 1 diabetes mellitus with other diabetic neurological complication |  |  |  |  |  |  |  |  |  |
|  | E1051 | Type 1 diabetes mellitus with diabetic peripheral angiopathy without gangrene |  |  |  |  |  |  |  |  |  |
|  | E1052 | Type 1 diabetes mellitus with diabetic peripheral angiopathy with gangrene |  |  |  |  |  |  |  |  |  |
|  | E1059 | Type 1 diabetes mellitus with other circulatory complications |  |  |  |  |  |  |  |  |  |
|  | E10610 | Type 1 diabetes mellitus with diabetic neuropathic arthropathy |  |  |  |  |  |  |  |  |  |
|  | E10618 | Type 1 diabetes mellitus with other diabetic arthropathy |  |  |  |  |  |  |  |  |  |
|  | E10620 | Type 1 diabetes mellitus with diabetic dermatitis |  |  |  |  |  |  |  |  |  |
|  | E10621 | Type 1 diabetes mellitus with foot ulcer |  |  |  |  |  |  |  |  |  |
|  | E10622 | Type 1 diabetes mellitus with other skin ulcer |  |  |  |  |  |  |  |  |  |
|  | E10628 | Type 1 diabetes mellitus with other skin complications |  |  |  |  |  |  |  |  |  |
|  | E10630 | Type 1 diabetes mellitus with periodontal disease |  |  |  |  |  |  |  |  |  |
|  | E10638 | Type 1 diabetes mellitus with other oral complications |  |  |  |  |  |  |  |  |  |
|  | E10641 | Type 1 diabetes mellitus with hypoglycemia with coma |  |  |  |  |  |  |  |  |  |
|  | E10649 | Type 1 diabetes mellitus with hypoglycemia without coma |  |  |  |  |  |  |  |  |  |
|  | E1065 | Type 1 diabetes mellitus with hyperglycemia |  |  |  |  |  |  |  |  |  |
|  | E1069 | Type 1 diabetes mellitus with other specified complication |  |  |  |  |  |  |  |  |  |
|  | E108 | Type 1 diabetes mellitus with unspecified complications |  |  |  |  |  |  |  |  |  |
|  | E1100 | Type 2 diabetes mellitus with hyperosmolarity without nonketotic hyperglycemic-hyperosmolar coma (NKHHC) |  |  |  |  |  |  |  |  |  |
|  | E1101 | Type 2 diabetes mellitus with hyperosmolarity with coma |  |  |  |  |  |  |  |  |  |
|  | E1110 | Type 2 diabetes mellitus with ketoacidosis without coma |  |  |  |  |  |  |  |  |  |
|  | E1111 | Type 2 diabetes mellitus with ketoacidosis with coma |  |  |  |  |  |  |  |  |  |
|  | E1121 | Type 2 diabetes mellitus with diabetic nephropathy |  |  |  |  |  |  |  |  |  |
|  | E1122 | Type 2 diabetes mellitus with diabetic chronic kidney disease |  |  |  |  |  |  |  |  |  |
|  | E1129 | Type 2 diabetes mellitus with other diabetic kidney complication |  |  |  |  |  |  |  |  |  |
|  | E11311 | Type 2 diabetes mellitus with unspecified diabetic retinopathy with macular edema |  |  |  |  |  |  |  |  |  |
|  | E11319 | Type 2 diabetes mellitus with unspecified diabetic retinopathy without macular edema |  |  |  |  |  |  |  |  |  |
|  | E113211 | Type 2 diabetes mellitus with mild nonproliferative diabetic retinopathy with macular edema, right eye |  |  |  |  |  |  |  |  |  |
|  | E113212 | Type 2 diabetes mellitus with mild nonproliferative diabetic retinopathy with macular edema, left eye |  |  |  |  |  |  |  |  |  |
|  | E113213 | Type 2 diabetes mellitus with mild nonproliferative diabetic retinopathy with macular edema, bilateral |  |  |  |  |  |  |  |  |  |
|  | E113219 | Type 2 diabetes mellitus with mild nonproliferative diabetic retinopathy with macular edema, unspecified eye |  |  |  |  |  |  |  |  |  |
|  | E113291 | Type 2 diabetes mellitus with mild nonproliferative diabetic retinopathy without macular edema, right eye |  |  |  |  |  |  |  |  |  |
|  | E113292 | Type 2 diabetes mellitus with mild nonproliferative diabetic retinopathy without macular edema, left eye |  |  |  |  |  |  |  |  |  |
|  | E113293 | Type 2 diabetes mellitus with mild nonproliferative diabetic retinopathy without macular edema, bilateral |  |  |  |  |  |  |  |  |  |
|  | E113299 | Type 2 diabetes mellitus with mild nonproliferative diabetic retinopathy without macular edema, unspecified eye |  |  |  |  |  |  |  |  |  |
|  | E113311 | Type 2 diabetes mellitus with moderate nonproliferative diabetic retinopathy with macular edema, right eye |  |  |  |  |  |  |  |  |  |
|  | E113312 | Type 2 diabetes mellitus with moderate nonproliferative diabetic retinopathy with macular edema, left eye |  |  |  |  |  |  |  |  |  |
|  | E113313 | Type 2 diabetes mellitus with moderate nonproliferative diabetic retinopathy with macular edema, bilateral |  |  |  |  |  |  |  |  |  |
|  | E113319 | Type 2 diabetes mellitus with moderate nonproliferative diabetic retinopathy with macular edema, unspecified eye |  |  |  |  |  |  |  |  |  |
|  | E113391 | Type 2 diabetes mellitus with moderate nonproliferative diabetic retinopathy without macular edema, right eye |  |  |  |  |  |  |  |  |  |
|  | E113392 | Type 2 diabetes mellitus with moderate nonproliferative diabetic retinopathy without macular edema, left eye |  |  |  |  |  |  |  |  |  |
|  | E113393 | Type 2 diabetes mellitus with moderate nonproliferative diabetic retinopathy without macular edema, bilateral |  |  |  |  |  |  |  |  |  |
|  | E113399 | Type 2 diabetes mellitus with moderate nonproliferative diabetic retinopathy without macular edema, unspecified eye |  |  |  |  |  |  |  |  |  |
|  | E113411 | Type 2 diabetes mellitus with severe nonproliferative diabetic retinopathy with macular edema, right eye |  |  |  |  |  |  |  |  |  |
|  | E113412 | Type 2 diabetes mellitus with severe nonproliferative diabetic retinopathy with macular edema, left eye |  |  |  |  |  |  |  |  |  |
|  | E113413 | Type 2 diabetes mellitus with severe nonproliferative diabetic retinopathy with macular edema, bilateral |  |  |  |  |  |  |  |  |  |
|  | E113419 | Type 2 diabetes mellitus with severe nonproliferative diabetic retinopathy with macular edema, unspecified eye |  |  |  |  |  |  |  |  |  |
|  | E113491 | Type 2 diabetes mellitus with severe nonproliferative diabetic retinopathy without macular edema, right eye |  |  |  |  |  |  |  |  |  |
|  | E113492 | Type 2 diabetes mellitus with severe nonproliferative diabetic retinopathy without macular edema, left eye |  |  |  |  |  |  |  |  |  |
|  | E113493 | Type 2 diabetes mellitus with severe nonproliferative diabetic retinopathy without macular edema, bilateral |  |  |  |  |  |  |  |  |  |
|  | E113499 | Type 2 diabetes mellitus with severe nonproliferative diabetic retinopathy without macular edema, unspecified eye |  |  |  |  |  |  |  |  |  |
|  | E113511 | Type 2 diabetes mellitus with proliferative diabetic retinopathy with macular edema, right eye |  |  |  |  |  |  |  |  |  |
|  | E113512 | Type 2 diabetes mellitus with proliferative diabetic retinopathy with macular edema, left eye |  |  |  |  |  |  |  |  |  |
|  | E113513 | Type 2 diabetes mellitus with proliferative diabetic retinopathy with macular edema, bilateral |  |  |  |  |  |  |  |  |  |
|  | E113519 | Type 2 diabetes mellitus with proliferative diabetic retinopathy with macular edema, unspecified eye |  |  |  |  |  |  |  |  |  |
|  | E113521 | Type 2 diabetes mellitus with proliferative diabetic retinopathy with traction retinal detachment involving the macula, right eye |  |  |  |  |  |  |  |  |  |
|  | E113522 | Type 2 diabetes mellitus with proliferative diabetic retinopathy with traction retinal detachment involving the macula, left eye |  |  |  |  |  |  |  |  |  |
|  | E113523 | Type 2 diabetes mellitus with proliferative diabetic retinopathy with traction retinal detachment involving the macula, bilateral |  |  |  |  |  |  |  |  |  |
|  | E113529 | Type 2 diabetes mellitus with proliferative diabetic retinopathy with traction retinal detachment involving the macula, unspecified eye |  |  |  |  |  |  |  |  |  |
|  | E113531 | Type 2 diabetes mellitus with proliferative diabetic retinopathy with traction retinal detachment not involving the macula, right eye |  |  |  |  |  |  |  |  |  |
|  | E113532 | Type 2 diabetes mellitus with proliferative diabetic retinopathy with traction retinal detachment not involving the macula, left eye |  |  |  |  |  |  |  |  |  |
|  | E113533 | Type 2 diabetes mellitus with proliferative diabetic retinopathy with traction retinal detachment not involving the macula, bilateral |  |  |  |  |  |  |  |  |  |
|  | E113539 | Type 2 diabetes mellitus with proliferative diabetic retinopathy with traction retinal detachment not involving the macula, unspecified eye |  |  |  |  |  |  |  |  |  |
|  | E113541 | Type 2 diabetes mellitus with proliferative diabetic retinopathy with combined traction retinal detachment and rhegmatogenous retinal detachment, right eye |  |  |  |  |  |  |  |  |  |
|  | E113542 | Type 2 diabetes mellitus with proliferative diabetic retinopathy with combined traction retinal detachment and rhegmatogenous retinal detachment, left eye |  |  |  |  |  |  |  |  |  |
|  | E113543 | Type 2 diabetes mellitus with proliferative diabetic retinopathy with combined traction retinal detachment and rhegmatogenous retinal detachment, bilateral |  |  |  |  |  |  |  |  |  |
|  | E113549 | Type 2 diabetes mellitus with proliferative diabetic retinopathy with combined traction retinal detachment and rhegmatogenous retinal detachment, unspecified eye |  |  |  |  |  |  |  |  |  |
|  | E113551 | Type 2 diabetes mellitus with stable proliferative diabetic retinopathy, right eye |  |  |  |  |  |  |  |  |  |
|  | E113552 | Type 2 diabetes mellitus with stable proliferative diabetic retinopathy, left eye |  |  |  |  |  |  |  |  |  |
|  | E113553 | Type 2 diabetes mellitus with stable proliferative diabetic retinopathy, bilateral |  |  |  |  |  |  |  |  |  |
|  | E113559 | Type 2 diabetes mellitus with stable proliferative diabetic retinopathy, unspecified eye |  |  |  |  |  |  |  |  |  |
|  | E113591 | Type 2 diabetes mellitus with proliferative diabetic retinopathy without macular edema, right eye |  |  |  |  |  |  |  |  |  |
|  | E169 | Disorder of pancreatic internal secretion, unspecified |  |  |  |  |  |  |  |  |  |
|  | E113592 | Type 2 diabetes mellitus with proliferative diabetic retinopathy without macular edema, left eye |  |  |  |  |  |  |  |  |  |
|  | E113593 | Type 2 diabetes mellitus with proliferative diabetic retinopathy without macular edema, bilateral |  |  |  |  |  |  |  |  |  |
|  | E113599 | Type 2 diabetes mellitus with proliferative diabetic retinopathy without macular edema, unspecified eye |  |  |  |  |  |  |  |  |  |
|  | E1136 | Type 2 diabetes mellitus with diabetic cataract |  |  |  |  |  |  |  |  |  |
|  | E1137X1 | Type 2 diabetes mellitus with diabetic macular edema, resolved following treatment, right eye |  |  |  |  |  |  |  |  |  |
|  | E1137X2 | Type 2 diabetes mellitus with diabetic macular edema, resolved following treatment, left eye |  |  |  |  |  |  |  |  |  |
|  | E1137X3 | Type 2 diabetes mellitus with diabetic macular edema, resolved following treatment, bilateral |  |  |  |  |  |  |  |  |  |
|  | E1137X9 | Type 2 diabetes mellitus with diabetic macular edema, resolved following treatment, unspecified eye |  |  |  |  |  |  |  |  |  |
|  | E1139 | Type 2 diabetes mellitus with other diabetic ophthalmic complication |  |  |  |  |  |  |  |  |  |
|  | E1140 | Type 2 diabetes mellitus with diabetic neuropathy, unspecified |  |  |  |  |  |  |  |  |  |
|  | E1141 | Type 2 diabetes mellitus with diabetic mononeuropathy |  |  |  |  |  |  |  |  |  |
|  | E1142 | Type 2 diabetes mellitus with diabetic polyneuropathy |  |  |  |  |  |  |  |  |  |
|  | E1143 | Type 2 diabetes mellitus with diabetic autonomic (poly)neuropathy |  |  |  |  |  |  |  |  |  |
|  | E1144 | Type 2 diabetes mellitus with diabetic amyotrophy |  |  |  |  |  |  |  |  |  |
|  | E1149 | Type 2 diabetes mellitus with other diabetic neurological complication |  |  |  |  |  |  |  |  |  |
|  | E1151 | Type 2 diabetes mellitus with diabetic peripheral angiopathy without gangrene |  |  |  |  |  |  |  |  |  |
|  | E1152 | Type 2 diabetes mellitus with diabetic peripheral angiopathy with gangrene |  |  |  |  |  |  |  |  |  |
|  | E1159 | Type 2 diabetes mellitus with other circulatory complications |  |  |  |  |  |  |  |  |  |
|  | E11610 | Type 2 diabetes mellitus with diabetic neuropathic arthropathy |  |  |  |  |  |  |  |  |  |
|  | E11618 | Type 2 diabetes mellitus with other diabetic arthropathy |  |  |  |  |  |  |  |  |  |
|  | E11620 | Type 2 diabetes mellitus with diabetic dermatitis |  |  |  |  |  |  |  |  |  |
|  | E11621 | Type 2 diabetes mellitus with foot ulcer |  |  |  |  |  |  |  |  |  |
|  | E11622 | Type 2 diabetes mellitus with other skin ulcer |  |  |  |  |  |  |  |  |  |
|  | E11628 | Type 2 diabetes mellitus with other skin complications |  |  |  |  |  |  |  |  |  |
|  | E11630 | Type 2 diabetes mellitus with periodontal disease |  |  |  |  |  |  |  |  |  |
|  | E11638 | Type 2 diabetes mellitus with other oral complications |  |  |  |  |  |  |  |  |  |
|  | E11641 | Type 2 diabetes mellitus with hypoglycemia with coma |  |  |  |  |  |  |  |  |  |
|  | E11649 | Type 2 diabetes mellitus with hypoglycemia without coma |  |  |  |  |  |  |  |  |  |
|  | E1165 | Type 2 diabetes mellitus with hyperglycemia |  |  |  |  |  |  |  |  |  |
|  | E1169 | Type 2 diabetes mellitus with other specified complication |  |  |  |  |  |  |  |  |  |
|  | E118 | Type 2 diabetes mellitus with unspecified complications |  |  |  |  |  |  |  |  |  |
|  | E119 | Type 2 diabetes mellitus without complications |  |  |  |  |  |  |  |  |  |
|  | E1300 | Other specified diabetes mellitus with hyperosmolarity without nonketotic hyperglycemic-hyperosmolar coma (NKHHC) |  |  |  |  |  |  |  |  |  |
|  | E1301 | Other specified diabetes mellitus with hyperosmolarity with coma |  |  |  |  |  |  |  |  |  |
|  | E1310 | Other specified diabetes mellitus with ketoacidosis without coma |  |  |  |  |  |  |  |  |  |
|  | E1311 | Other specified diabetes mellitus with ketoacidosis with coma |  |  |  |  |  |  |  |  |  |
|  | E1321 | Other specified diabetes mellitus with diabetic nephropathy |  |  |  |  |  |  |  |  |  |
|  | E1322 | Other specified diabetes mellitus with diabetic chronic kidney disease |  |  |  |  |  |  |  |  |  |
|  | E1329 | Other specified diabetes mellitus with other diabetic kidney complication |  |  |  |  |  |  |  |  |  |
|  | E13311 | Other specified diabetes mellitus with unspecified diabetic retinopathy with macular edema |  |  |  |  |  |  |  |  |  |
|  | E13319 | Other specified diabetes mellitus with unspecified diabetic retinopathy without macular edema |  |  |  |  |  |  |  |  |  |
|  | E133211 | Other specified diabetes mellitus with mild nonproliferative diabetic retinopathy with macular edema, right eye |  |  |  |  |  |  |  |  |  |
|  | E133212 | Other specified diabetes mellitus with mild nonproliferative diabetic retinopathy with macular edema, left eye |  |  |  |  |  |  |  |  |  |
|  | E133213 | Other specified diabetes mellitus with mild nonproliferative diabetic retinopathy with macular edema, bilateral |  |  |  |  |  |  |  |  |  |
|  | E133219 | Other specified diabetes mellitus with mild nonproliferative diabetic retinopathy with macular edema, unspecified eye |  |  |  |  |  |  |  |  |  |
|  | E133291 | Other specified diabetes mellitus with mild nonproliferative diabetic retinopathy without macular edema, right eye |  |  |  |  |  |  |  |  |  |
|  | E133292 | Other specified diabetes mellitus with mild nonproliferative diabetic retinopathy without macular edema, left eye |  |  |  |  |  |  |  |  |  |
|  | E133293 | Other specified diabetes mellitus with mild nonproliferative diabetic retinopathy without macular edema, bilateral |  |  |  |  |  |  |  |  |  |
|  | E133299 | Other specified diabetes mellitus with mild nonproliferative diabetic retinopathy without macular edema, unspecified eye |  |  |  |  |  |  |  |  |  |
|  | E133311 | Other specified diabetes mellitus with moderate nonproliferative diabetic retinopathy with macular edema, right eye |  |  |  |  |  |  |  |  |  |
|  | E133312 | Other specified diabetes mellitus with moderate nonproliferative diabetic retinopathy with macular edema, left eye |  |  |  |  |  |  |  |  |  |
|  | E133313 | Other specified diabetes mellitus with moderate nonproliferative diabetic retinopathy with macular edema, bilateral |  |  |  |  |  |  |  |  |  |
|  | E133319 | Other specified diabetes mellitus with moderate nonproliferative diabetic retinopathy with macular edema, unspecified eye |  |  |  |  |  |  |  |  |  |
|  | E133391 | Other specified diabetes mellitus with moderate nonproliferative diabetic retinopathy without macular edema, right eye |  |  |  |  |  |  |  |  |  |
|  | E133392 | Other specified diabetes mellitus with moderate nonproliferative diabetic retinopathy without macular edema, left eye |  |  |  |  |  |  |  |  |  |
|  | E133393 | Other specified diabetes mellitus with moderate nonproliferative diabetic retinopathy without macular edema, bilateral |  |  |  |  |  |  |  |  |  |
|  | E133399 | Other specified diabetes mellitus with moderate nonproliferative diabetic retinopathy without macular edema, unspecified eye |  |  |  |  |  |  |  |  |  |
|  | E133411 | Other specified diabetes mellitus with severe nonproliferative diabetic retinopathy with macular edema, right eye |  |  |  |  |  |  |  |  |  |
|  | E133412 | Other specified diabetes mellitus with severe nonproliferative diabetic retinopathy with macular edema, left eye |  |  |  |  |  |  |  |  |  |
|  | E133413 | Other specified diabetes mellitus with severe nonproliferative diabetic retinopathy with macular edema, bilateral |  |  |  |  |  |  |  |  |  |
|  | E133419 | Other specified diabetes mellitus with severe nonproliferative diabetic retinopathy with macular edema, unspecified eye |  |  |  |  |  |  |  |  |  |
|  | E133491 | Other specified diabetes mellitus with severe nonproliferative diabetic retinopathy without macular edema, right eye |  |  |  |  |  |  |  |  |  |
|  | E133492 | Other specified diabetes mellitus with severe nonproliferative diabetic retinopathy without macular edema, left eye |  |  |  |  |  |  |  |  |  |
|  | E133493 | Other specified diabetes mellitus with severe nonproliferative diabetic retinopathy without macular edema, bilateral |  |  |  |  |  |  |  |  |  |
|  | E133499 | Other specified diabetes mellitus with severe nonproliferative diabetic retinopathy without macular edema, unspecified eye |  |  |  |  |  |  |  |  |  |
|  | E133511 | Other specified diabetes mellitus with proliferative diabetic retinopathy with macular edema, right eye |  |  |  |  |  |  |  |  |  |
|  | E133512 | Other specified diabetes mellitus with proliferative diabetic retinopathy with macular edema, left eye |  |  |  |  |  |  |  |  |  |
|  | E133513 | Other specified diabetes mellitus with proliferative diabetic retinopathy with macular edema, bilateral |  |  |  |  |  |  |  |  |  |
|  | E133519 | Other specified diabetes mellitus with proliferative diabetic retinopathy with macular edema, unspecified eye |  |  |  |  |  |  |  |  |  |
|  | E133521 | Other specified diabetes mellitus with proliferative diabetic retinopathy with traction retinal detachment involving the macula, right eye |  |  |  |  |  |  |  |  |  |
|  | E133522 | Other specified diabetes mellitus with proliferative diabetic retinopathy with traction retinal detachment involving the macula, left eye |  |  |  |  |  |  |  |  |  |
|  | E133523 | Other specified diabetes mellitus with proliferative diabetic retinopathy with traction retinal detachment involving the macula, bilateral |  |  |  |  |  |  |  |  |  |
|  | E133529 | Other specified diabetes mellitus with proliferative diabetic retinopathy with traction retinal detachment involving the macula, unspecified eye |  |  |  |  |  |  |  |  |  |
|  | E133531 | Other specified diabetes mellitus with proliferative diabetic retinopathy with traction retinal detachment not involving the macula, right eye |  |  |  |  |  |  |  |  |  |
|  | E133532 | Other specified diabetes mellitus with proliferative diabetic retinopathy with traction retinal detachment not involving the macula, left eye |  |  |  |  |  |  |  |  |  |
|  | E133533 | Other specified diabetes mellitus with proliferative diabetic retinopathy with traction retinal detachment not involving the macula, bilateral |  |  |  |  |  |  |  |  |  |
|  | E133539 | Other specified diabetes mellitus with proliferative diabetic retinopathy with traction retinal detachment not involving the macula, unspecified eye |  |  |  |  |  |  |  |  |  |
|  | E133541 | Other specified diabetes mellitus with proliferative diabetic retinopathy with combined traction retinal detachment and rhegmatogenous retinal detachment, right eye |  |  |  |  |  |  |  |  |  |
|  | E133542 | Other specified diabetes mellitus with proliferative diabetic retinopathy with combined traction retinal detachment and rhegmatogenous retinal detachment, left eye |  |  |  |  |  |  |  |  |  |
|  | E133543 | Other specified diabetes mellitus with proliferative diabetic retinopathy with combined traction retinal detachment and rhegmatogenous retinal detachment, bilateral |  |  |  |  |  |  |  |  |  |
|  | E133549 | Other specified diabetes mellitus with proliferative diabetic retinopathy with combined traction retinal detachment and rhegmatogenous retinal detachment, unspecified eye |  |  |  |  |  |  |  |  |  |
|  | E133551 | Other specified diabetes mellitus with stable proliferative diabetic retinopathy, right eye |  |  |  |  |  |  |  |  |  |
|  | E133552 | Other specified diabetes mellitus with stable proliferative diabetic retinopathy, left eye |  |  |  |  |  |  |  |  |  |
|  | E133553 | Other specified diabetes mellitus with stable proliferative diabetic retinopathy, bilateral |  |  |  |  |  |  |  |  |  |
|  | E133559 | Other specified diabetes mellitus with stable proliferative diabetic retinopathy, unspecified eye |  |  |  |  |  |  |  |  |  |
|  | E133591 | Other specified diabetes mellitus with proliferative diabetic retinopathy without macular edema, right eye |  |  |  |  |  |  |  |  |  |
|  | E133592 | Other specified diabetes mellitus with proliferative diabetic retinopathy without macular edema, left eye |  |  |  |  |  |  |  |  |  |
|  | E133593 | Other specified diabetes mellitus with proliferative diabetic retinopathy without macular edema, bilateral |  |  |  |  |  |  |  |  |  |
|  | E133599 | Other specified diabetes mellitus with proliferative diabetic retinopathy without macular edema, unspecified eye |  |  |  |  |  |  |  |  |  |
|  | E1336 | Other specified diabetes mellitus with diabetic cataract |  |  |  |  |  |  |  |  |  |
|  | E1337X1 | Other specified diabetes mellitus with diabetic macular edema, resolved following treatment, right eye |  |  |  |  |  |  |  |  |  |
|  | E1337X2 | Other specified diabetes mellitus with diabetic macular edema, resolved following treatment, left eye |  |  |  |  |  |  |  |  |  |
|  | E1337X3 | Other specified diabetes mellitus with diabetic macular edema, resolved following treatment, bilateral |  |  |  |  |  |  |  |  |  |
|  | E1337X9 | Other specified diabetes mellitus with diabetic macular edema, resolved following treatment, unspecified eye |  |  |  |  |  |  |  |  |  |
|  | E1339 | Other specified diabetes mellitus with other diabetic ophthalmic complication |  |  |  |  |  |  |  |  |  |
|  | E1340 | Other specified diabetes mellitus with diabetic neuropathy, unspecified |  |  |  |  |  |  |  |  |  |
|  | E1341 | Other specified diabetes mellitus with diabetic mononeuropathy |  |  |  |  |  |  |  |  |  |
|  | E1342 | Other specified diabetes mellitus with diabetic polyneuropathy |  |  |  |  |  |  |  |  |  |
|  | E1343 | Other specified diabetes mellitus with diabetic autonomic (poly)neuropathy |  |  |  |  |  |  |  |  |  |
|  | E1344 | Other specified diabetes mellitus with diabetic amyotrophy |  |  |  |  |  |  |  |  |  |
|  | E1349 | Other specified diabetes mellitus with other diabetic neurological complication |  |  |  |  |  |  |  |  |  |
|  | E1351 | Other specified diabetes mellitus with diabetic peripheral angiopathy without gangrene |  |  |  |  |  |  |  |  |  |
|  | E1352 | Other specified diabetes mellitus with diabetic peripheral angiopathy with gangrene |  |  |  |  |  |  |  |  |  |
|  | E1359 | Other specified diabetes mellitus with other circulatory complications |  |  |  |  |  |  |  |  |  |
|  | E13610 | Other specified diabetes mellitus with diabetic neuropathic arthropathy |  |  |  |  |  |  |  |  |  |
|  | E13618 | Other specified diabetes mellitus with other diabetic arthropathy |  |  |  |  |  |  |  |  |  |
|  | E13620 | Other specified diabetes mellitus with diabetic dermatitis |  |  |  |  |  |  |  |  |  |
|  | E13621 | Other specified diabetes mellitus with foot ulcer |  |  |  |  |  |  |  |  |  |
|  | E13622 | Other specified diabetes mellitus with other skin ulcer |  |  |  |  |  |  |  |  |  |
|  | E13628 | Other specified diabetes mellitus with other skin complications |  |  |  |  |  |  |  |  |  |
|  | E13630 | Other specified diabetes mellitus with periodontal disease |  |  |  |  |  |  |  |  |  |
|  | E13638 | Other specified diabetes mellitus with other oral complications |  |  |  |  |  |  |  |  |  |
|  | E13641 | Other specified diabetes mellitus with hypoglycemia with coma |  |  |  |  |  |  |  |  |  |
|  | E13649 | Other specified diabetes mellitus with hypoglycemia without coma |  |  |  |  |  |  |  |  |  |
|  | E1365 | Other specified diabetes mellitus with hyperglycemia |  |  |  |  |  |  |  |  |  |
|  | E1369 | Other specified diabetes mellitus with other specified complication |  |  |  |  |  |  |  |  |  |
|  | E138 | Other specified diabetes mellitus with unspecified complications |  |  |  |  |  |  |  |  |  |
|  |  |  |  |  |  |  |  |  |  |  |  |
